# A click-based modular approach to introduction of peroxides onto molecules and nanostructures†

**DOI:** 10.1039/d0ra09088c

**Published:** 2020-12-16

**Authors:** Alissa Horn, Patrick H. Dussault

**Affiliations:** Department of Chemistry, University of Nebraska-Lincoln Lincoln NE 68588-0304 USA pdussault1@unl.edu

## Abstract

Copper-promoted azide/alkyne cycloadditions (CuAAC) are explored as a tool for modular introduction of peroxides onto molecules and nanomaterials. Dialkyl peroxide-substituted alkynes undergo Cu(i)-promoted reaction with azides in either organic or biphasic media to furnish peroxide-substituted 1,2,3-triazoles. Heterolytic fragmentation of the peroxide to an aldehyde, a side reaction that appears to be related to the formation of the triazole, can be suppressed by use of excess alkyne, the presence of triethylsilane, or by use of iodoalkyne substrates. Complementary reactions of simple alkynes with azido-substituted peroxides are much less efficient. Click reactions of alkynyl peroxyacetals are also reported; reductive fragmentation can be minimized by increasing the distance between the peroxyacetal and the alkyne. The strategy enables modular introduction of dialkyl peroxides and peroxyacetals onto gold nanoparticles, the first such process to be reported.

## Abstract 2

Copper-promoted azide/alkyne cycloadditions (CuAAC) are explored as a tool for modular introduction of peroxides onto molecules and nanomaterials.

## Graphical abstract

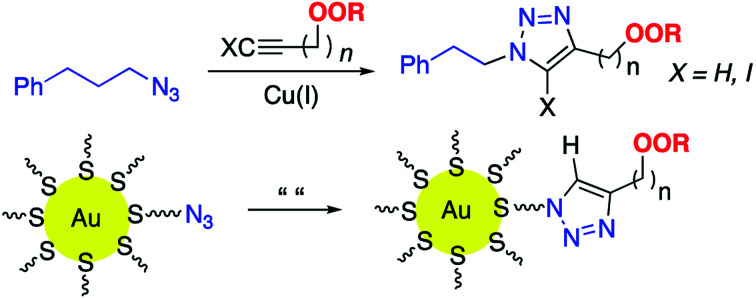

## Introduction

Organic peroxides are capable of a wide range of reaction pathways,^1^ and have been applied as oxidants,^2^ radical initiators,^3^ pharmacophores,^4^ enzyme inhibitors,^5^ and synthons for electrophilic transfer of alkoxide.^6^ Peroxides are also of interest as intermediates and products of oxidative degradation and sources of reactive oxygen species.^7^ This rich chemistry contrasts with the limited number of reports describing reactivity or application of peroxides at surfaces.^8^ This discontinuity cannot be completely attributed to concerns for stability or safety. Peroxides are compatible with a surprising variety of synthetic transformations,^9^ and many peroxides may be safely employed by following established precautions.^10^ A more significant barrier to broader use of peroxides is the lack of effective methods for their modular introduction under mild conditions.

“Click chemistry”, a family of strategies based upon rapid and specific pairwise reactions of matched functional groups, has become an indispensable tool for modular approaches to molecules, supramolecules, and functionalized surfaces and nanoparticles.^11^ The most widely applied of these transformations, is the copper-assisted azide/alkyne cycloaddition (CuAAC), now a workhorse reaction for ligation and modification of molecules and supramolecules.^12^ CuAAC chemistry has been employed to generate derivatives of the peroxide antimalarial artemisinin,^13^ and we became interested in the potential for modular introduction of peroxides *via* click reactions. We now describe explorations of the scope of CuAAC chemistry reaction in terms of the nature of the peroxide group, whether the peroxide is linked to the alkyne or azide partner, the distance of the peroxide from the reaction center, and the influence of reaction conditions. The fragmentation of the peroxides observed in some of the click reactions of peroxyacetals demonstrates a potential for controlled generation of reactive oxygen species. We also demonstrate successful application of these ligations to modular introduction of peroxides on the surface of functionalized Au nanoparticles (AuNP), a nanomaterial platform widely applied for sensing, imaging, and biointeraction applications.^14^

## Results

**Fig. 1:**
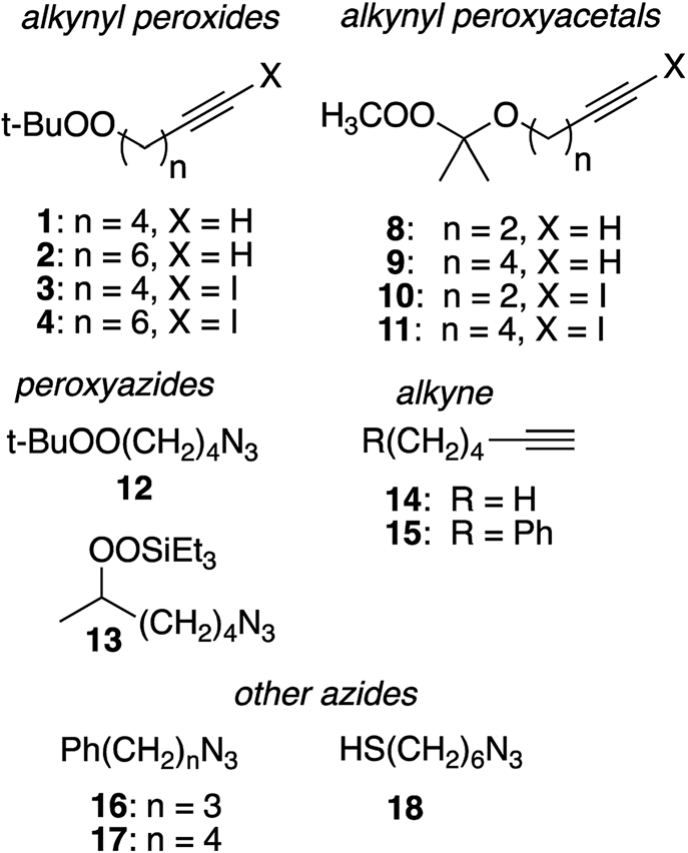
Substrates used for CuAAC chemistry studies.

Substrates employed in CuAAC chemistry are illustrated in Fig. 1.

**Scheme 1:**
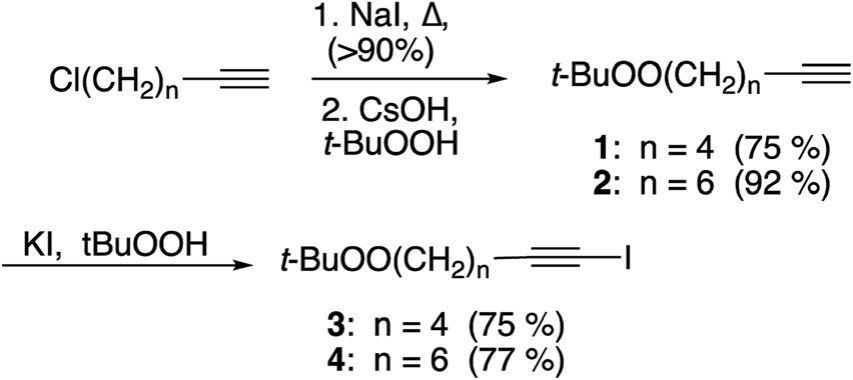
Synthesis of alkynyl and iodoalkynyl peroxides.

Unfunctionalized peroxyalkynes 1 and 2 were easily assembled (Scheme 1) *via* Finkelstein exchange on the corresponding chloroalkyne,^15^ followed by nucleophilic displacement of the iodide with *tert*-butyl hydroperoxide in the presence of CsOH.^16^ Iodination of the terminal alkyne furnished the corresponding iodoalkynes (3 and 4) in good yield.^17^

**Scheme 2:**
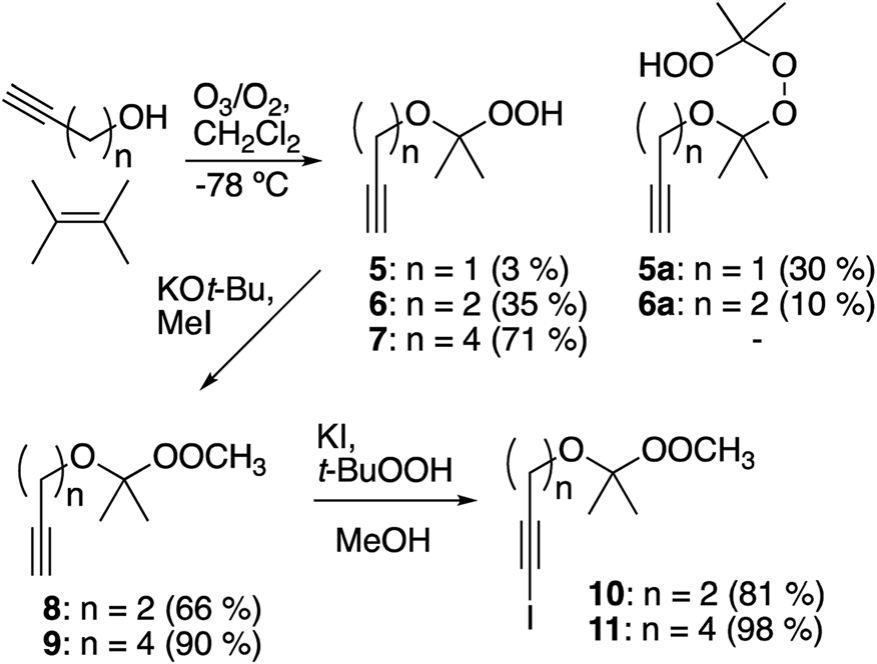
Synthesis of alkynyl- and iodoalkynyl peroxyacetals.

Preparation of alkynyl peroxyacetals was based upon trapping of an ozonolysis-derived carbonyl oxide with an alkynol (Scheme 2).^18^ This strategy, although rooted in the successful preparation of unsaturated hydroperoxyacetals from allyl alcohols,^19^ proved to be strongly dependent upon the structure of the alkynol. Ozonolysis of 2,3-dimethyl-2-butene in the presence of propargyl alcohol mainly afforded hydroperoxyperoxide 5a, which arises through reaction of the desired hydroperoxyacetal (5) with additional carbonyl oxide. The dimeric product predominates even in the presence of excess propargyl alcohol, presumably reflecting limited alcohol nucleophilicity.^20^ In contrast, the corresponding reactions of 3-butyn-1-ol and 5-hexyn-1-ol gave moderate and good yields of hydroperoxyacetals 6 and 7, respectively. The hydroperoxyacetals were readily methylated to afford peroxyacetals 8 and 9.^21^ Iodination of the peroxyacetals furnished iodoalkynyl acetal 10 and 11.

**Scheme 3:**
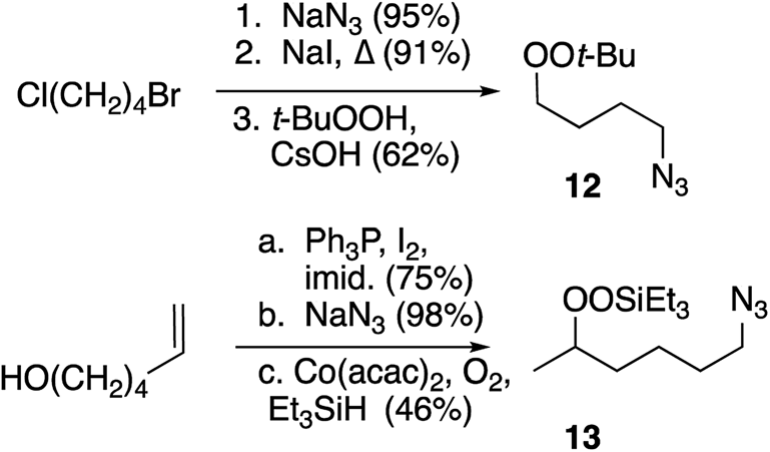
Synthesis of peroxy azides.

Preparation of peroxide-substituted azides is summarized in Scheme 3. Selective nucleophilic substitution of 1-bromo-4-chlorobutane afforded 1-chloro-4-azidobutane,^22^ which underwent CsOH-promoted substitution with *tert*-butyl hydroperoxide to generate azidoalkyl peroxide 12. The synthesis of a secondary peroxide/azide began with conversion of 5-hexene-2-ol to the corresponding iodide. Nucleophilic displacement with sodium azide was followed by Mukaiyama peroxidation to generate peroxy azide 13.^23^ As 12 and 13 both incorporate two energy-rich groups within a low molecular weight framework,^24^ initial syntheses were conducted on modest scales (∼1 mmol). Although both molecules proved to be stable to at least 100 °C (open chamber DSC, range limited by volatility), subsequent preparations were limited to ≤1 g and we avoided exposing either molecule to elevated temperatures.^10^ Preparations of 5-hexynyl benzene (15), azidoalkylbenzenes 16 and 17, and 6-azido hexanethiol (18) are detailed in the Experimental section.

### Copper(i) catalyzed click reaction with peroxy alkyne

Reaction of peroxyalkyne 1 with azidoalkylbenzenes 16 or 17 under either homogeneous (CuI and NEt_3_, in THF) or biphasic (CuSO_4_, sodium ascorbate, methylene chloride/water) conditions led to the rapid (≤15 min) appearance of triazoles 19 or 20 (Table 1);^12,25^ the presence of a peroxide was evident based upon a redox-sensitive TLC indicator.^26^ These initial reactions were stopped after brief reaction periods (1.5–3 h); longer reaction times led to consumption of starting material but also contamination with a nearly inseparable byproduct later established to be the tetrazole aldehyde (21, *vide infra*). A small amount of the corresponding 5-iodotriazole 20i, easily distinguished from 20 by the ^13^C signal for C_5_ (120.7 ppm for C–H *vs.* 78.0 ppm for C–I) was isolated from the reaction employing CuI.^27^

**Table 1:** Conditions for click chemistry with peroxy alkynes

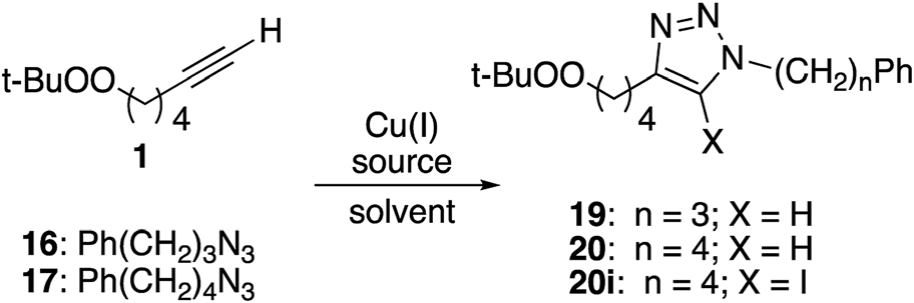					
Azide	Cu(i) source	Solv.	*t* (h)	Conv.	Prod. (yield, %)
16	CuCl, NEt_3_	THF	3	89%	19 (25%)
17	CuI, NEt_3_	THF	3	80%	20 (37%)
					20i (5%)
17	CuSO_4_, Na ascorbate	CH_2_Cl_2_/H_2_O	1.5	85%	20 (42%)

The relationship between reaction time and aldehyde formation was examined using the reaction of 1 and 17 (Table 2). Ratios of peroxide products (20, 20i) *vs.* aldehyde (21) were established by NMR; samples of pure aldehyde were also isolated for characterization. The near absence of aldehyde for reactions conducted in the presence of excess alkyne (entries 6 and 9) would emerge as an important tool (*vide infra*).

**Table 2:** Influence of reaction time on byproduct formation

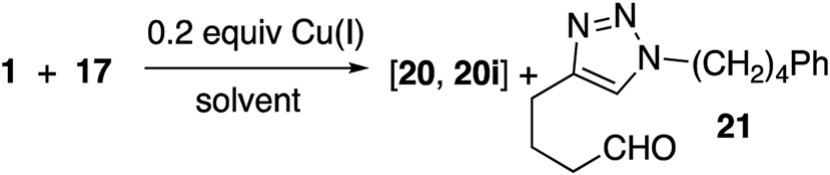				
Entry	Catalyst	Solvent	*t* (h)	21 (yield^a^, %)
1	CuI/Et_3_N	THF	1	6%
2	〃	〃	3	8%
3	〃	〃	8	13%
4	〃	〃	18	20%
5	〃	〃	27	30%
6^b^	〃	〃	3	<2%
7	CuSO_4_/sodium ascorbate	CH_2_Cl_2_/H_2_O	4	20%
8^c^	〃	〃	4	38%
9	〃	〃	18	57%
10^b^	〃	〃	1.5	5%
11^b^	〃	〃	4	22%

a Ratios assessed by ^1^H NMR.

b 1.2 equiv. 1.

c 0.4 equiv. Cu(i).

Resubjecting isolated triazole 20 to either set of reaction conditions led to formation of aldehyde 21 (eqn (1)); in the presence of CuSO_4_/ascorbate, the aldehyde became the major product.1
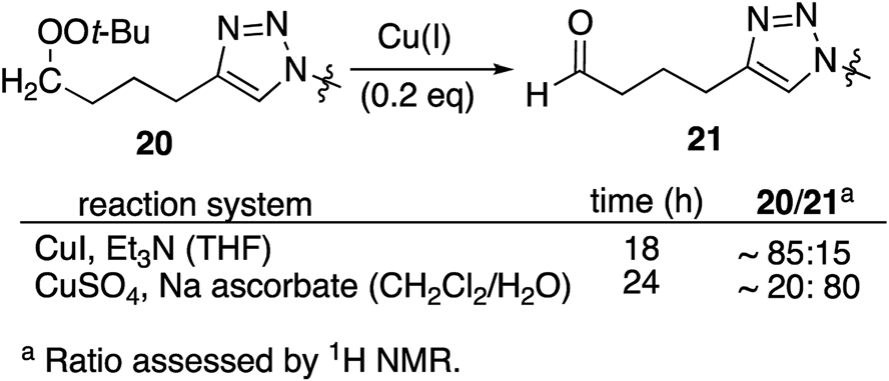


In an effort to better understand the factors leading to aldehyde formation, we probed the reactivity of triazole 20 towards reaction components (Table 3). No decomposition was observed in the presence of base or CuSO_4_ or ascorbate. However, treatment with CuI, CuI/triethylamine, or CuSO_4_/Na ascorbate generated significant amounts of aldehyde; the same was true for reaction with either Cu(i) source and azide. No aldehyde was observed in the presence of mixtures containing added alkyne (entries 5, 6, 11 and 12) which did not result in aldehyde formation. Interestingly, the 5-iodotriazole (20i) was unaffected by conditions which rapidly degraded 20 (entry 13).

**Table 3:** Stability of peroxide tetrazoles to reaction components

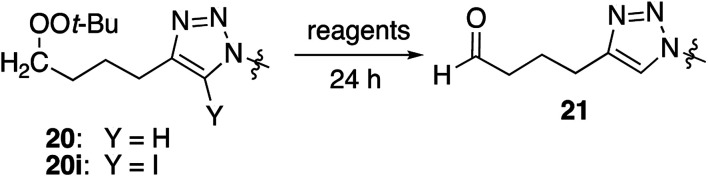					
Entry	S. mat	Reagents	Azide (equiv.)	Alkyne (equiv.)	21 (yield^a^, %)
1	20	Et_3_N	—	—	Nr
2	20	CuI	—	—	95%
3	20	CuI, Et_3_N	—	—	70%
4	20	〃	1.0	—	50%
5	20	〃	1.0	1.0	0%
6	20	〃	—	1.0	0%
7	20	Na ascorbate	—	—	0%
8	20	CuSO_4_, Na ascorbate	—	—	43%
9	20	CuSO_4_	—	—	0%
10	20	CuSO_4_, Na ascorbate	1.0	—	61%
11	20	CuSO_4_, Na ascorbate	1.0	1.0	0%
12	20	CuSO_4_, Na ascorbate		1.0	0%
13	20i	CuI, Et_3_N	—	—	0%

a NMR yields; isolated yield in parentheses.

The results in Table 3 suggest that the formation of aldehyde may be accelerated in the presence of the neighboring triazole. Supporting this hypothesis, alkynyl peroxide 1 proved inert towards reaction with copper iodide in THF (eqn (2)).2
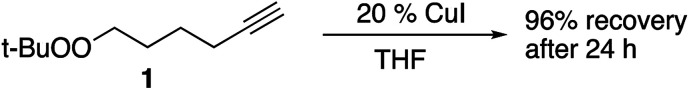


Consistent with literature reports,^28^ click reactions proceeded more rapidly in the presence of tris[(1-benzyl-1*H*-1,2,3-triazol-4-yl)methyl]amine (TBTA), a Cu(i) ligand (Table 4). However, aldehyde formation continuing to be a problem and the overall yields of clicked peroxide were no greater, and sometimes less, than for reactions in the absence of the ligand. In contrast, performing the CuI or CuSO_4_/ascorbate-catalyzed reactions in the presence of excess alkyne completely suppressed aldehyde formation. This led to the hypothesis that the lack of byproduct might reflect the ability of free alkyne to protonate the C_5_-cuprated triazole intermediate in CuAAC chemistry.^12,29^ We also investigated this reaction in the presence of a silane, a functionality also known to quench organocopper species,^30^ and again observed suppression of aldehyde formation. Finally, replacement of the terminal alkyne with an iodide furnished only the 5-iodoalkyl peroxide; no aldehyde was observed. These reactions, although allowed to proceed for 18–24 h, were typically complete (TLC) within 30 minutes.

**Table 4:** Influence of additives

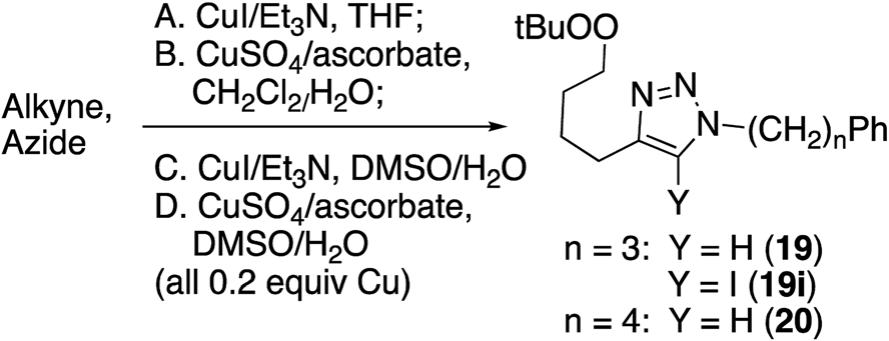					
Alkyne	Azide	Cond.	Additive (equiv.)	*t* (h)	Product (yield, %)
1	17	A	Alkyne 1 (1.2)	18	20 (55%)
1	17	B	Alkyne 1 (1.2)	18	20 (68%)
1	17	C	TBTA (1%)	3	20 (20%)
1	17	D	TBTA (1%)	3	20 (35%)
1	16	A	Et_3_SiH (1.2)	24	19 (62%)
3	16	A	—	24	19i (65%)

Iodoalkynes display enhanced reactivity relative to terminal alkynes in CuAAC reactions,^27^ and this is also observed in the reactions of the peroxide-substrates. A competition between structurally analogous alkyne derivatives led to preferential consumption of the iodoalkynyl peroxide 3.3
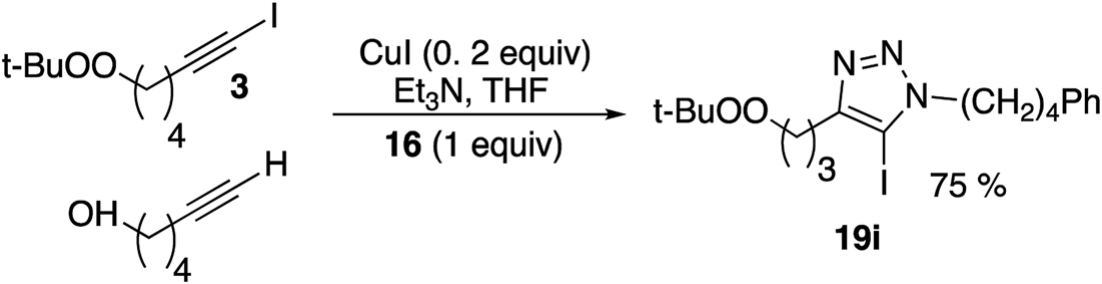


### Proximity of peroxide and alkyne

Click reactions of peroxyoctyne 2 and the corresponding iodoalkyne (4) produced a good yield of peroxy triazoles 22/23 and iodotetrazoles 22i/23i (Table 5). For reactions employing CuI as the copper source, formation of the aldehyde byproducts 24/25 was minimal (2) or did not occur at all (4). In the case of reactions using CuSO_4_, aldehyde formation was significant for reactions involving alkyne 2 and minimal for iodoalkyne 4.

**Table 5:** CuAAC reaction of longer dialkyl peroxide

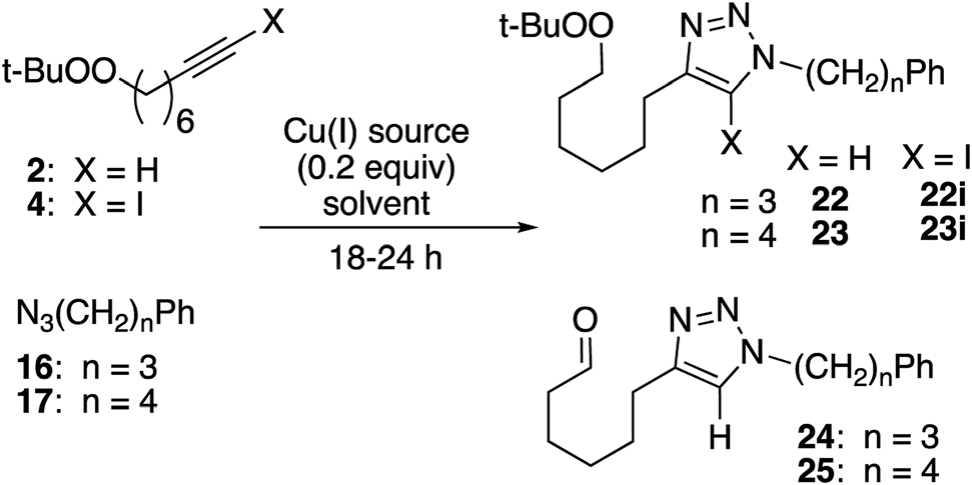					
Alkyne, azide	X	Cu(i)^a^	Conv. (%)	Peroxide (yield, %)	Aldehyde (yield, %)
2, 16	H	CuI	87.6	22 (68%)	24 (11%)
				22i (6%)	
2, 16	H	CuSO_4_	90	22 (41%)	24 (37%)
4, 16	I	CuI	94	22i (76%)^b^	—
2, 16	H	CuI^c^	∼10	22 (50%)	—
				22i (3%)	
2, 17	H	CuI	87	23 (55%)	25 (29%)
				23i (4%)	
2, 17	H	CuSO_4_	89	23 (39%)	25 (50%)

a CuI/Et_3_N (0.2 equiv.), THF; CuSO_4_/sodium ascorbate, DCM/H_2_O (biphasic).

b Significant amount of unidentified polar byproduct.

c Et_3_SiH (1.9 equiv.) also present.

### Alkynyl peroxyacetals

Cu(i)-promoted reaction of the butynyl peroxyacetal (8) with azides 16 or 17 gave poor results (eqn (4)). In some cases, we observed successful click reaction to form inseparable mixtures of the desired peroxyacetal and the acetate ester derived from peroxyacetal fragmentation. No improvement was observed based upon the presence of excess alkyne, TBTA, or triethylsilane. In the presence of promoters derived from Cu(ii), the reactions underwent a color change from dark green to light blue after 30–60 minutes. The color change could be temporarily reversed by the addition of more ascorbate but little or no clicked product was detected. Additionally, minimal progress was observed with the corresponding iodoalkynyl peroxyacetal (10).4
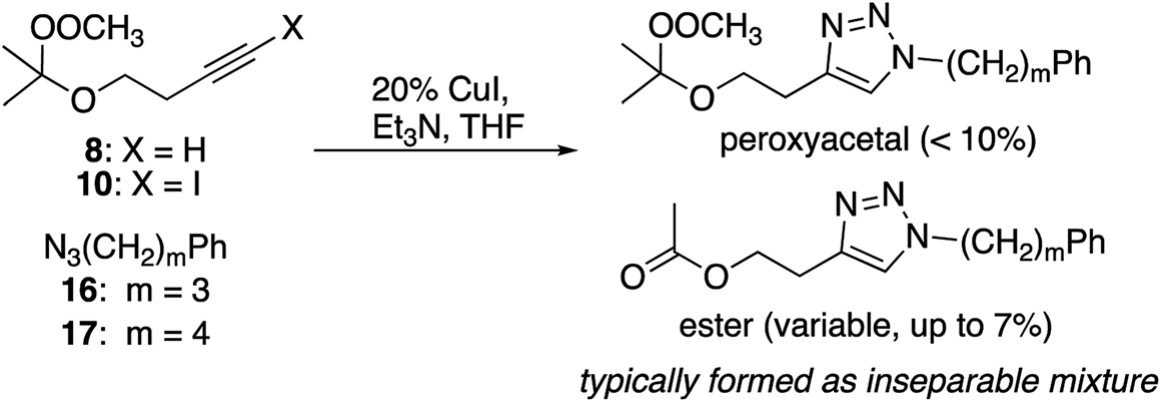


In contrast, the longer chain peroxyacetal (9) and the corresponding iodoalkyne (11) both reacted with azides in the presence of Cu(i) to give peroxy triazoles as the only major product (Table 6). Once again, no reaction was observed in the presence of a promoter derived from CuSO_4_/ascorbate.

**Table 6:** Click chemistry of hexynyl peroxyacetals

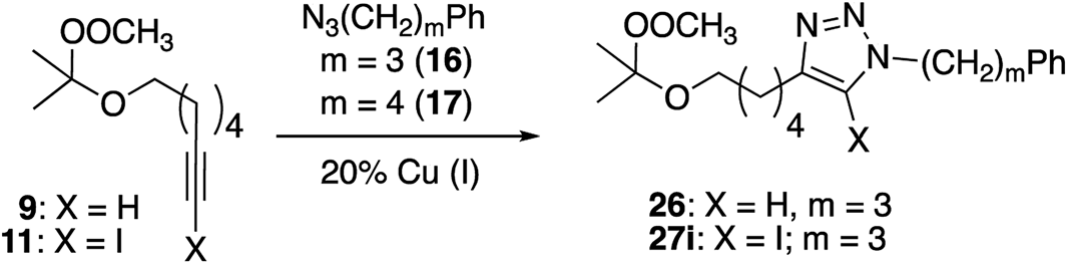				
Alkyne	Azide	Cu(i) source	*t* (h)	Product (yield^a^, %)
9	17	CuI, Et_3_N	3	26 (45%)
9	17	CuSO_4_, ascorbate	24	Nr
11	16	CuI, Et_3_N	18	27i (33%)

a Isolated.

A separate control experiment (eqn (5)) made obvious the stability of the alkynyl peroxyacetals towards Cu(i) in the absence of other reaction components.5
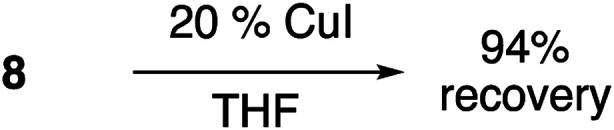


### CuAAC reactions of peroxyalkyl azides

Reactions of simple alkynes with peroxide-containing azide 12 proceeded in modest yield using CuI/Et_3_N (Table 7); the corresponding reactions with CuSO_4_/ascorbate provided lower yields and required substantially longer reaction times. In both series, significant amounts of the peroxyazide were recovered. Although analysis of these reactions was complicated by the presence of inseparable byproducts, we could verify that little or no aldehyde was generated under these conditions.

**Table 7:** CuAAC chemistry with peroxyalkyl azides

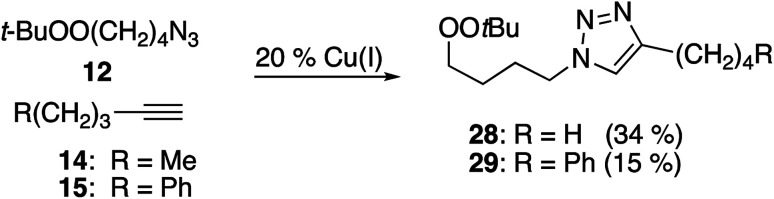				
Alkyne	Reagents	*t* (h)	Conv. (12)	Product (yield, %)
14	CuI/Et_3_N	3	71%	28 (34%)
14	CuSO_4_/Na ascorbate	24	80%	28 (29%)
15	CuI/Et_3_N	3	76%	29 (15%)
15	CuSO_4_/Na ascorbate	24	42%	29 (≤5%)

Secondary silyl peroxide 13 failed to give clicked products (eqn (6)); instead, producing a mixture of reduction and fragmentation products.^1*a*,31^ In the absence of alkyne, 13 was stable to both the CuI or CuSO_4_ reagent systems (not shown).6
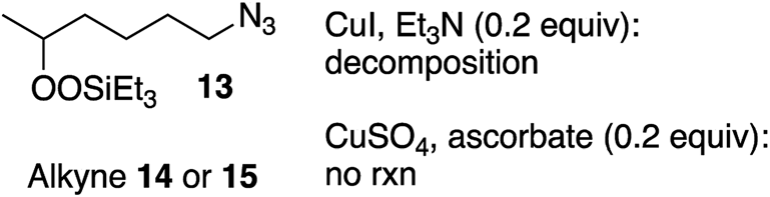


### Influence of peroxides on the CuAAC reaction

The lower reactivity and yields observed with the peroxy-substituted azides led us to investigate whether the presence of a peroxide was exerting a general dampening influence on the CuAAC reaction. The reaction of 1-hexyne (14) and phenylalkyl azide 17, which went to completion in just a few minutes under our typical reaction conditions, failed to proceed at all in the presence of a stoichiometric amount of peroxide 13 (eqn (7)).7
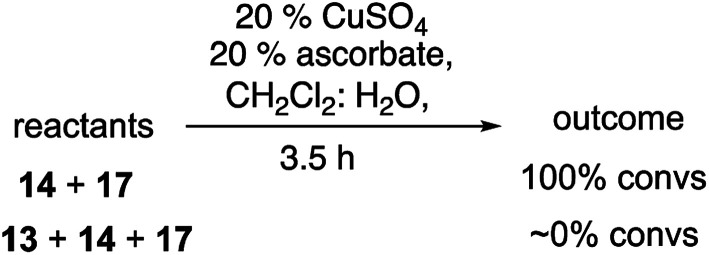


### Nanoparticle functionalization

Having identified useful conditions for CuAAC reactions of alkynyl peroxides and peroxyacetals, we became interested in applying the chemistry to functionalization of nanoparticles. CuAAC-based functionalization of AuNPs, which exploits the high affinity of thiols for gold and the resulting ability to create azide-functionalized nanoparticles, has been widely applied to a variety of applications in both organic and aqueous media,^11,14*a*,32–37^ and we focused our attention on this system.

**Scheme 4:**
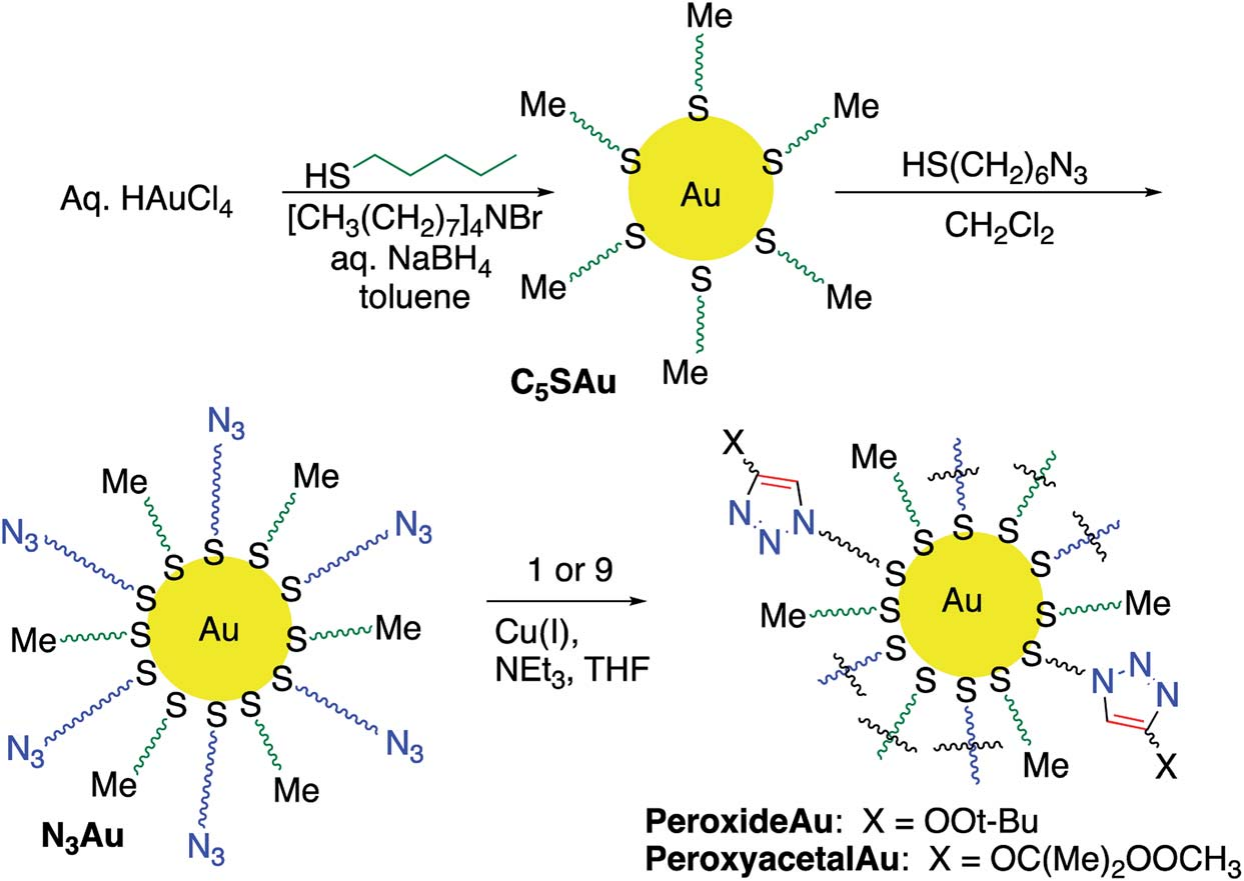
Synthesis of peroxide-functionalized nanoparticles.

**Fig. 2:**
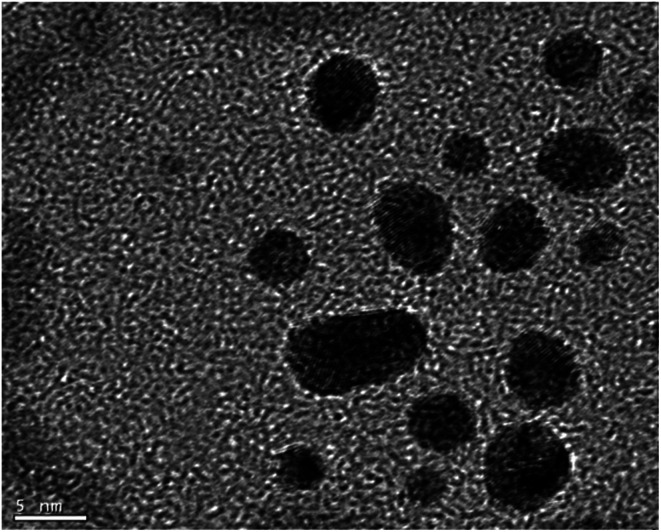
TEM of C5SAu. Scale bar = 5 nm.

**Fig. 3:**
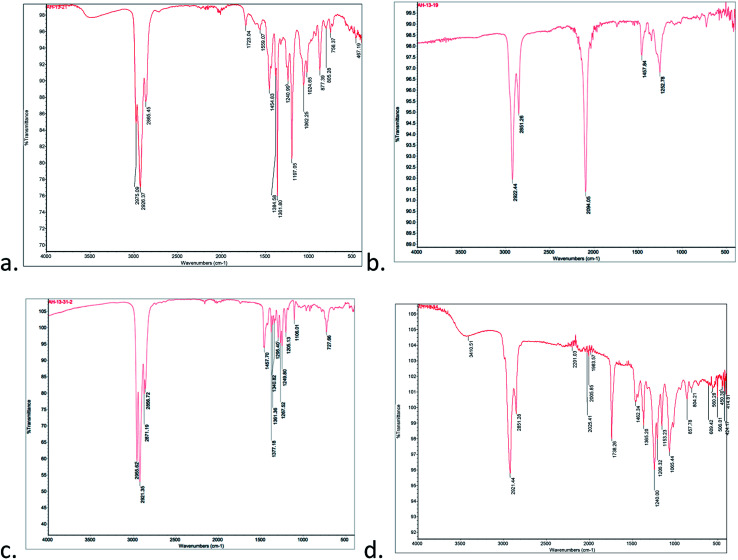
IR of nanoparticles: (a) C_5_SAu; (b) N_3_Au; (c) after click with peroxyalkyne 1; (d) after click with alkynyl peroxyacetal 9.

**Fig. 4:**
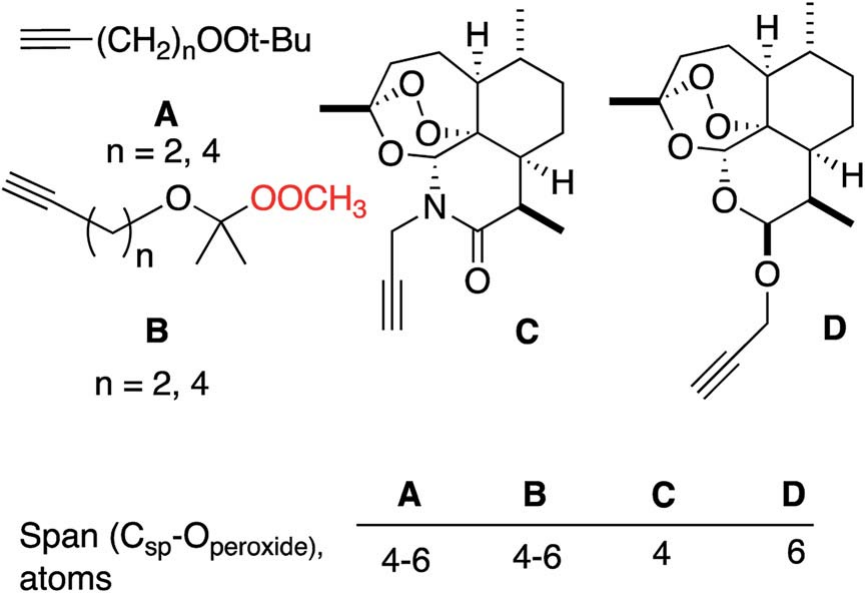
Comparison with artemisinin-derived “click” substrates.

Azide-functionalized nanoparticles (N_3_Au) were prepared using a variant of a reported procedure in which Au nanoparticles (AuNP) are subsequently reacted with a passivating and then a functionalized thiol (Scheme 4).^38^ Addition of a slight excess of pentanethiol to a biphasic mixture of HAuCl_3_ and tetraoctylammonium bromide resulted in a white suspension which reacted with freshly prepared aqueous sodium borohydride to generate a dark and opaque suspension of pentanethiolate-functionalized nanoparticles (C_5_SAu). The partially passivated nanoparticles were isolated by centrifugation.^11*c*^ TEM (Fig. 2) spectra confirmed the presence of 2–5 nm particles. The presence of the alkylthio chains was evident by the ^1^H signals for a terminal methyl group and the methylene groups at C_3_/C_4_ (ESI-Fig. 1†).^39^ XPS analysis revealed the surface region of the nanoparticles to consist of 50.1% Au, 43.9% C, and 6.0% S, suggesting that approximately 75% of the Au surface remained accessible for further functionalization (ESI-Fig. 2–4†).^40^ Repetition of the procedure using dodecanethiol, the preliminary passivating agent described in the original report, generated nanoparticles with similar properties (ESI-Fig. 5–8†).^11*c*^

Treatment of the C_5_SH-Au with excess 6-azidohexane thiol furnished the azide-modified nanoparticle (N_3_Au) which was isolated and purified using centrifugation and resuspension. The presence of the azide was evident from the strong IR absorbance at 2094 cm^−1^ (Fig. 3, compare panels a and b) and by the new ^1^H NMR signal at 3.31 ppm corresponding to *CH*_*2*_N_3_ (ESI-Fig. 9†); this signal was easily distinguished from signal for unbound azide (3.50 ppm), which was also observed in inadequately washed samples. The ∼2 : 3 ratio of the integrals for the signals corresponding to **CH**_**2**_N_3_*vs.* CH_2_**CH**_**3**_ suggests a similar extent of coverage. XPS suggested a surface composition of 54.0% Au, 34.2% C, 8.5% S, and 3.3% (ESI-Fig. 10–13†).

### Peroxide functionalization of nanoparticles

Addition of triethylamine and cat. copper iodide into a THF solution containing peroxyalkyne 1 and N_3_Au led to the immediate appearance of a new peroxide-active spot on TLC (see Experimental details).^26^ After being quenched with aq. NH_4_Cl, the reaction was concentrated and the residue purified away from residual reactant by repeated resuspension (MeOH) and centrifugation. The azide stretch was now absent in the IR spectrum (Fig. 3d), which instead showed weak tetrazole-related stretches at 1723 cm^−1^ and 1559 cm^−1^ and only minimal absorptions associated with an aldehyde (1705–1710 cm^−1^). Evidence for the click reaction could also be seen in the ^1^H NMR (ESI-Fig. 14†) as the loss of the *δ* 3.31 signal corresponding to the CH_2_N_3_ and new signals at 1.27 (*t*-Bu) and 3.99 (OO). XPS established a product composition of 26.4% Au and 73.6% pentanethiol and peroxide triazole (ESI-Fig. 15–19†).

We also investigated click functionalization of azidoNP with alkynyl peroxy acetal 9. Consumption of the azide was obvious in the lack of the ∼2100 cm^−1^ IR stretch and the observation of a new C–H stretch at 2921 cm^−1^ (Fig. 3d). Although ester formation was equally obvious as an IR stretch at 1738 cm^−1^, the limited magnitude of this peak, combined with the comparison in the ^1^H NMR (ESI-Fig. 20†) of signals for the MeOO (3.54 ppm) and the *gem*-dimethyl groups of the acetal (1.57 ppm) *vs.* the peak group at 3.86 ppm (CH_2_O) of both acetal and ester, suggested the majority of the functionalized chain remained as peroxyacetal. XPS (ESI-Fig. 21–25†) suggested a surface composition of 20.0% Au and 80.0% pentanethiol and peroxyacetal triazole.

## Discussion

Our results demonstrate that both peroxides and peroxyacetals can, under suitable conditions, serve as modular components for Cu-promoted CuAAC click reactions. The click reactions of the alkynyl peroxides and peroxyacetals proceed at rates in line with literature reports, with reactions times of 0.5–1 h for terminal alkynes and ≤0.75 h for 1-iodoalkynes.^12,27^ This is consistent with Zhu's observation that electron-poor alkynes are excellent substrates for CuAAC reactions.^41^ An “isomeric” set of click reactions involving simple alkynes and azidoalkyl peroxides is less efficient. The basis for inhibition of azide reactivity at a four-carbon distance from a peroxide remains unclear; however, we should note that we have observed a dampening influence of peroxides on S_N_2 reactions across a similar span.^42^

**Scheme 5:**
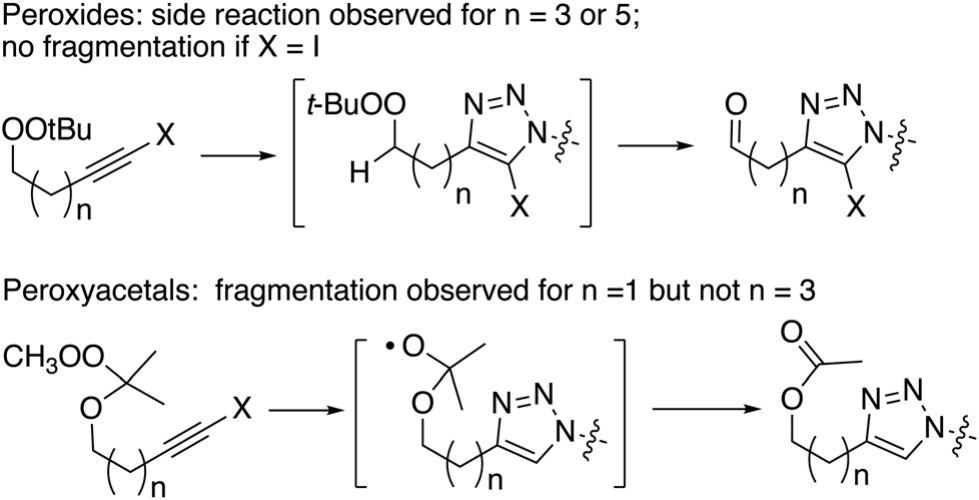
Overview of decomposition processes.

The use of CuI in organic solvents gives more consistent and often superior results compared to the use of CuSO_4_/ascorbate in biphasic media. The alkynyl peroxides and alkynyl peroxyacetal substrates, although stable towards Cu(i), are both prone to decomposition reactions in the presence of the reaction intermediates (Scheme 5). Interestingly, the efficiency of the reactions, the nature of the decomposition reactions, and the approaches to control of decomposition, are different for the two classes of substrates.

The dialkyl peroxides give modest to moderate yields under traditional CuAAC conditions due to the accumulation of a byproduct derived from fragmentation of the peroxide to an aldehyde. Aldehyde formation can be suppressed by use of an iodinated alkyne substrate, by performance of reactions in the presence of excess alkyne or added silane; under these conditions the yield of clicked peroxide is in the range of 62–68%. For the peroxyacetals, reactions proceed in lower yield, and, in the case of a shorter chain series (8), generate a significant amount of a byproduct in which the peroxyacetal has been fragmented to an acetoxy group. The formation of the ester is largely suppressed in a longer-chain peroxyacetal or for reactions of iodoalkynyl peroxyacetals. However, regardless of conditions, the yields obtained from peroxyacetals do not rival those obtained from the dialkyl peroxides.

**Scheme 6:**
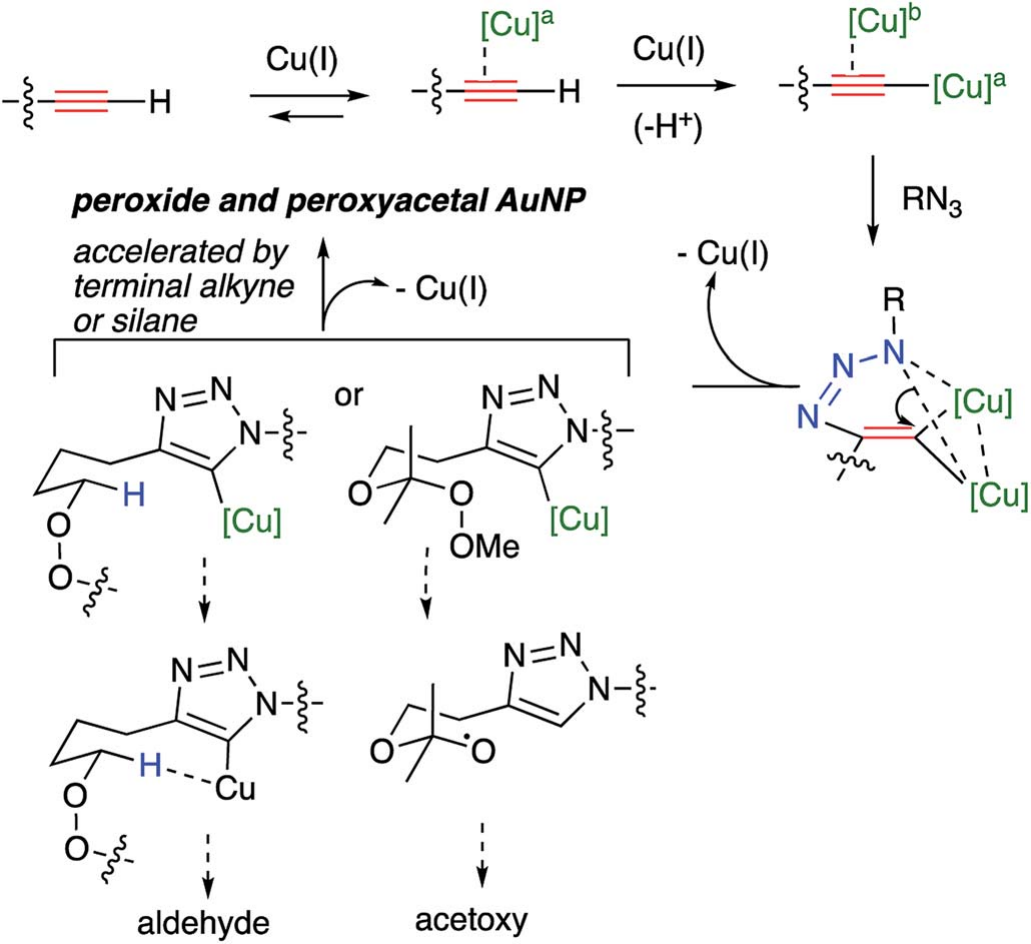
CuAAC mechanism and potential pathways for peroxide fragmentation.

An interpretation of our results in the context of the accepted mechanism of the CuAAC reaction (Scheme 6) suggests the decomposition reactions result from interaction of the peroxide or peroxyacetal with a heteroaryl organocopper intermediate generated at C_5_ of the developing triazole.^12^ Evidence in support of this hypothesis includes: the instability of the peroxyalkyne to any mixture containing Cu(i) and triazole; the lack of fragmentation observed for iodoalkynes; and the minimization of fragmentation the presence of excess alkyne or added triethylsilane, either of which would be expected to protonate the sp^2^-RCu.^30^ The clean formation of aldehyde, and the high barriers associated with electrochemical or chemical reduction of dialkyl peroxides,^43^ suggest the decomposition is a heterolytic process involving abstraction of the adjacent C–H.^1*a*^

The formation of an ester group (acetoxy) from fragmentations of the hydroperoxyacetals suggests the intermediacy of an alkoxy radical derived from cleavage of the peroxyacetal by an electron-donor; the likely reducing agent is the metalated triazole described above (Scheme 6).^44^ In contrast to the reactions of dialkyl peroxides, byproduct formation for the peroxyacetals is not suppressed by the presence of excess alkyne or added silane; it is suppressed by use of iodoalkynyl substrates or by increasing the peroxide/alkyne distance.

It is interesting to compare our results with reported CuAAC reactions of alkynyl derivatives of artemisinin-derived propargyl acetals and amides (Fig. 4); the approximate distance between the peroxide and the reaction site is similar in both systems.^13^ The heterolytic fragmentation we observed with dialkyl peroxides is not available in the artemisinin derivatives due to the lack of adjacent C–H groups. However, the fact that radical fragmentations are not observed in the artemisinin derivatives may indicate that the SET fragmentation requires close approach of the organocopper intermediates and the peroxyacetal, something that is possible in A and B and not possible in C or D.

CuAAC-based functionalization of AuNPs, which exploits the high affinity of thiols for gold has been widely applied to a variety of applications in both organic and aqueous media.^11*c*,14*b*,*c*,32–37^ At the outset of our studies, we were concerned by reports suggesting that CuAAC reactions on gold nanoparticles require longer reaction periods or use of microwave heating,^45,46^ and can be accompanied by nanoparticle aggregation and precipitation.^45,47^ Our results indicated that modular introduction of peroxides on nanoparticles *via* CuAAC suffers from no such limitations, and can be performed in less than eight hours and in the absence of microwave heating. Installation of dialkyl peroxides proceeds cleanly even in the absence of additives required in solution reactions, suggesting that the high local concentration of alkyne relative to the surface N_3_ groups suppresses aldehyde formation. Peroxyacetals, which are much more activated than dialkyl peroxides towards cleavage,^6,43^ also undergo successful click reactions on surfaces. However, in this case, the side reaction observed in solution phase chemistry continued to be observed in reactions on nanoparticles. This reaction, in contrast to some results on the extremely hindered peroxyacetal core of artemisinin, is suppressed by the use of a substrate containing a greater span between the peroxide and alkyne groups.

With the exception of functionalized SAMs displaying peroxides (*via* ozonolysis of alkene-terminated monolayers)^8^ or diacyl peroxides (*via* condensation of H_2_O_2_ with carboxylic acids),^48,49^ there are few examples of covalent introduction of peroxide on surfaces. Much of this is likely to relate to concerns about compatibility of the surface with the functionalization method; for example, ozone is known to attack the thiol/Au interface and is incompatible with many electron-rich groups.^18*b*,50^ Our work demonstrates that the CuAAC reaction can be used to install both dialkyl peroxides and the more reactive peroxyacetals on nanoparticle surfaces.

## Conclusions

Our work, the first systematic investigation of the factors influencing the use of click reactions for installing organic peroxides, demonstrates that the copper-assisted click offers a practical approach for modular introduction of dialkyl peroxides and peroxyacetals. The best results are obtained with dialkyl peroxides; a side reaction involving heterolytic fragmentation of the peroxide can be easily circumvented by choice of substrate or additive. Although installation of more reactive peroxyacetals can be complicated by a radical fragmentation, this process can be minimized by use of a greater span between the peroxide and the reaction site.

Our work also provides first example of the use of click chemistry for modular introduction of organic peroxides on surfaces. Reactions are rapid, proceed to high conversion of nanoparticle surface functionality, and can be accomplished under mild conditions and without nanoparticle aggregation. Peroxide-functionalized surfaces could provide the basis for reactive or antimicrobial coatings,^51^ as well as a platform for investigating spatially-constrained interactions or reactions of peroxides or derived reactive oxygen species.^52^ Along these lines, the peroxyacetals investigated here, in addition to offering a source of alkoxy radicals, are known to be easily hydrolyzed to generate free hydroperoxides,^16^ while the simple *t*-alkyl peroxides, although nearly inert to simple reducing agents and Fe(ii), are known to be activated photochemically^52,53^ or by iron/thiol complexes.^43^

## Experimental procedures

### General methods

All reagents and solvents were used as purchased except for CH_2_Cl_2_ (distilled from CaH_2_), DMF (vacuum distilled from CaH_2_), and THF (distilled from Na/Ph_2_CO). All nonaqueous reactions were conducted under an atmosphere of N_2_ in flame-dried glassware. Thin layer chromatography (TLC) was performed on 0.25 mm hard-layer silica plates. Developed plates were visualized by 254 nm UV lamp and/or by staining: 2.5% ammonium molybdate and 0.5% ceric sulfate in 10% aqueous sulfuric acid (general stain, after heating); 1% aq. potassium permanganate (alkynes); 1% *N*,*N*′-dimethyl-*p*-phenylenediamine in 1 : 20 : 100 acetic acid/water/methanol (specific for peroxides; dialkyl peroxides and peroxyacetals can be visualized as a reddish or reddish-green spot upon heating);^26^ or vanillin and sulfuric acid (3% each) in ethanol (general stain, after heating). Unless otherwise described, chromatography refers to silica flash chromatography.

NMR spectra were acquired in CDCl_3_ at 400 (^1^H) or 100 MHz (^13^C) unless otherwise noted. Chemical shifts are reported relative to residual chloroform (7.26 ppm, ^1^H; 77.36 ppm, ^13^C). ^1^H spectra are reported as chemical shift (multiplicity, *J* couplings in Hz, number of protons). IR spectra were recorded as neat films on a ZrSe crystal; selected absorbances are reported in cm^−1^. High resolution mass spectra (HRMS) were obtained at the Nebraska Center for Mass Spectrometry at UNL. XPS and TEM spectra were acquired in the Nebraska Center for Nanoscience and Materials on a Thermo Scientific K-Alpha X-ray photoelectron spectrometer using a monochromated Al Kα (1486.6 eV) X-ray source and a dual-beam flood source using a FEI Tecnai Osiris (scanning) transmission electron microscope.

CAUTION: Although we experienced no exotherms or explosions during the reported investigations, any work with peroxides of moderate or high active oxygen content, in particular any steps involving heating and/or concentration, should be conducted following standard precautions (adjusting scale to perceived hazard; concentration, and when necessary, reaction behind shields; some analysis of the thermal sensitivity of new products). Readers are directed towards a web-published overview of peroxide safety.^10*d*^

### Synthesis of peroxyalkynes

**6-(*tert*-Butylperoxy)-1-hexyne (** **1** **)** was prepared by an adaptation of published procedures.^54^ To a solution of 6-chloro-1-hexyne (4.66 g, 40.0 mmol) in acetone (150 mL) was added sodium iodide (32.98 g, 220.0 mmol, 5.5 equiv.). The reaction was stirred under reflux for 18 hours. The cooled reaction was then diluted with water (50 mL) and extracted with hexanes (50 mL × 3). The combined organic layers were dried with Na_2_SO_4_ and the residue concentrated under reduced pressure to yield 7.5307 g (91%) of 6-iodo-1-hexyne as a yellow oil which was used without purification. Spectral details matched those previously reported.^15,55^*R*_f_ = 0.42 (10% EA/Hex); ^1^H *δ* 3.20 (t, *J* = 8.0, 2H), 2.22 (td, *J* = 3.5, *J* = 9.3, 2H), 1.92 (t, *J* = 9.3, 1H), 1.63 (p, *J* = 6.4, 2H); ^13^C *δ* 83.7, 69.0, 32.3, 29.2, 17.5, 6.2.

To a solution of CsOH monohydrate (4.50 g, 30.0 mmol, 1.2 equiv.) in DMF (100 mL) at 0 °C was added dropwise TBHP as an ∼5.5 M solution in decane (6.81 mL, 37.5 mmol, 1.5 equiv.). The mixture was stirred for 30 min, whereupon 6-iodo-1-hexyne (5.20 g, 25.0 mmol) was added. The reaction was allowed to slowly warm to room temperature. After 5 h, the reaction was quenched with water (30 mL) and extracted with hexanes (30 mL × 3). The combined organic layers were dried with Na_2_SO_4_, concentrated under reduced pressure and the residue purified by column chromatography (5% EA/Hex) to yield 3.981 g (94%) of 6-(*tert*-butylperoxy)-1-hexyne as a colorless oil. Spectral details matched previous reports:^54*a*^*R*_f_ = 0.56 (10% EA/Hex) ^1^H *δ* 3.96 (t, *J* = 6.4, 2H), 2.22 (td, *J* = 7.0, *J* = 2.6, 2H), 1.94 (t, *J* = 2.6, 1H), 1.72 (m, 2H), 1.61 (m, 2H), 1.24 (s, 9H); ^13^C *δ* 84.3, 80.2, 74.5, 68.6, 27.2, 26.5, 25.4, 18.4; IR: 3314, 2924, 2119, 1362, 1198, 628; HRMS (ESI^+^, TOF) calcd for C_10_H_18_NaO_2_ [M + Na]^+^: 193.1204; found: 193.1205.

**8-(*tert*-Butylperoxy)-1-octyne (** **2** **)** was prepared by a similar procedure as described above. Reaction of 8-chloro-1-octyne (3.3914 g, 23.45 mmol) and sodium iodide (19.366 g, 129.2 mmol, 5.5 equiv.) furnish 5.0135 g (91%) of 8-iodo-1-octyne as a yellow oil which was used without further purification. Spectral details matched those previously reported:^56^*R*_f_: 0.43 (10% EA/Hex); ^1^H *δ* 3.19 (t, *J* = 7.0, 2H), 2.19 (td, *J* = 6.6, *J* = 2.2, 2H), 1.94 (t, *J* = 2.6, 1H), 1.83 (p, *J* = 6.7, 2H), 1.53 (m, 2H), 1.41–1.43 (m, 4H); ^13^C *δ* 84.5, 68.4, 33.5, 30.1, 28.3, 27.7, 18.4, 7.1.

By the same method as employed for synthesis of 1, reaction of the iodooctyne (3.8046 g, 16.50 mmol) with CsOH monohydrate (3.310 g, 22.1 mmol, 1.3 equiv.) and TBHP as an ∼5.5 M solution in decane (4.50 mL, 24.75 mmol, 1.5 equiv., ∼5.5 M solution in decane) in DMF, furnished 3.0118 g (92%) of 8-(*tert*-butylperoxy)-1-octyne (2) as a colorless oil: *R*_f_: 0.55 (10% EA/Hex); ^1^H *δ* 3.93 (t, *J* = 6.6, 2H), 2.18 (td, *J* = 6.9, *J* = 2.5, 2H), 1.93 (t, *J* = 2.6, 1H), 1.50–1.64 (m, 4H), 1.35–1.46 (m, 4H), 1.24 (s, 9H); ^13^C *δ* 84.7, 80.2, 75.1, 68.3, 28.7, 28.5, 27.9, 26.4, 25.8, 18.4 IR: 3312, 2945 (s), 2956, 1456, 1360 (s), 1198 (s); HRMS (ESI^+^, TOF) calcd for C_12_H_22_NaO_2_ [M + Na]^+^: 221.1517; found: 221.1510.

**1-Iodo-6-*tert*-butylperoxy hexyne (** **3** **)** was prepared using an adaptation of a published procedure.^17^ To a solution of 6-(*tert*-butylperoxy)-1-hexyne (850 mg, 4.99 mmol) in MeOH (20 mL) was added KI (1.02 g, 6.13 mmol, 1.2 equiv.), followed by TBHP as an ∼5.5 M solution in decane (1.36 mL, 7.48 mmol, 1.2 equiv.). The reaction was stirred at room temperature for 18 hours. The reaction was quenched with saturated aqueous Na_2_S_2_O_3_ (10 mL), washed with brine (10 mL), and extracted with EA (15 mL × 3). The combined organic layers were dried with Na_2_SO_4_, and the residue concentrated under reduced pressure to yield 1.1083 g (75%) of iodoalkynyl peroxide 3 as a light yellow oil: *R*_f_ = 0.55 (20% EA/Hex); ^1^H *δ* 3.95 (t, *J* = 6.3, 2H), 2.40 (t, *J* = 6.8, 2H), 1.70 (p, *J* = 6.1, 2H), 1.59 (p, *J* = 7.0, 2H), 1.24 (s, 9H) ^13^C *δ* 94.4, 80.3, 74.4, 27.2, 26.5, 25.4, 20.8, −6.8; IR 2925, 1362, 1196, 877; HRMS (ESI^+^, TOF) calcd for C_10_H_17_INaO_2_ [M + Na]^+^: 319.0171; found: 319.0174.

#### 1-Iodo-8-*tert*-butylperoxy octyne (4)

By a similar procedure as employed for synthesis of iodohexynyl peroxide 3, reaction of 8-(*tert*-butylperoxy)-1-octyne (714.9 mg, 3.60 mmol), KI (685.5 mg, 4.13 mmol, 1.2 equiv.), and TBHP (1.0 mL, 5.5 mmol, 1.5 equiv.) furnished, after column chromatography (10% EA/Hex), 901.1 mg (77%) of iodooctynyl peroxide 4 as a light yellow oil: *R*_f_: 0.57 (20% EA/Hex); ^1^H *δ* 3.95 (t, *J* = 6.7, 2H), 2.38 (t, *J* = 7.0, 2H), 1.62 (p, *J* = 7.1, 2H), 1.54 (p, *J* = 7.0, 2H), 1.26–1.36 (m, 4H), 1.26 (s, 9H); ^13^C *δ* 94.8, 80.2, 75.0, 28.7, 28.5, 27.9, 26.5, 25.8, 20.9, −7.4; IR: 2977, 2937, 2860, 1362, 1197; HRMS (ESI^+^, TOF) calcd for C_12_H_21_INaO_2_ [M + Na]^+^: 347.0484; found: 347.0485.

### Synthesis of alkynyl peroxyacetals

**3-((2-Hydroperoxypropan-2-yl)oxy)-1-propyne(** **5** **)** and **3-((2-((2-hydroperoxypropan-2-yl)peroxy)propan-2-yl)oxy)-1-propyne (5a)** were prepared using a procedure reported for unsaturated alcohols.^19^ To a solution of propargyl alcohol (0.97 mL, 16.9 mmol, 3 equiv.) in CH_2_Cl_2_ (50 mL) was added 2,3-dimethyl-2-butene (0.65 mL, 5.47 mmol). This mixture was cooled to −78 °C whereupon a gaseous stream of O_3_/O_2_ was admitted (pipette, ∼0.5–1 mmol O_3_/minute). When the reaction was complete (TLC) introduction of O_3_/O_2_ was halted and N_2_ was briefly bubbled through the reaction mixture. The solution was carefully concentrated under reduced pressure (CAUTION) and the residue purified by column chromatography (5% ether/pentane) to yield 210.8 mg (20%) of hydroperoxyperoxide 5a as a colorless oil, followed by traces of volatile hydroperoxyacetal 5. (CAUTION: the nearly 16% active oxygen content of hydroperoxyperoxide 5a suggests a moderate to high potential for exothermic decomposition.) As a result, this preparation was confined to <20 mmol scale and products were handled carefully (see general Discussion on safety, above).^10*d*^

5a: *R*_f_ = 0.46 (20% EA/Hex); ^1^H *δ* 9.61 (s, 1H), 4.31 (t, *J* = 2.4, 2H), 2.43 (t, *J* = 2.3, 1H), 1.51 (s, 6H), 1.42 (s, 6H); ^13^C *δ* 109.4, 106.8, 80.4, 74.3, 51.2, 23.5, 20.9; IR: 3362, 3286, 2999, 2946, 1369, 1176, 1034, 826, 620; HRMS (ESI^+^, TOF) calcd for C_9_H_16_NaO_5_ [M + Na]^+^: 227.0895; found: 227.0896.

5: (trace byproduct; incompletely characterized as a colorless oil): *R*_f_ = 0.43 (20% EA/Hex); ^1^H *δ* 9.88 (s, 1H), 4.20 (t, *J* = 2.4, 2H), 2.45 (t, *J* = 2.4, 1H), 1.41 (s, 6H); ^13^C *δ* 105.9, 81.3, 73.7, 49.9, 22.7.

**4-((2-Hydroperoxypropan-2-yl)oxy)-1-butyne** (6) and **4-((2-((2-hydroperoxypropan-2-yl)peroxy)propan-2-yl)oxy)-1-butyne** (6a) were prepared using the same procedure described above. To a solution of 3-butyn-1-ol (1.97 mL, 26.0 mmol, 2 equiv.) in CH_2_Cl_2_ (125 mL) was added 2,3-dimethyl-2-butene (1.56 mL, 13.1 mmol). This mixture was cooled to −78 °C whereupon a gaseous stream of O_3_/O_2_ was admitted (approximately 1 mmol O_3_/minute). When the reaction as judged complete, by TLC, introduction of O_3_/O_2_ was halted and N_2_ was briefly bubbled through the reaction mixture. The solution was carefully concentrated under reduced pressure and the residue (CAUTION) purified by column chromatography (5% ether/pentane) to yield 455.6 mg (24%) of 6 as an colorless oil, accompanied by 141.5 mg (5%) of 6a. CAUTION: The products shown here are hydroperoxides or peroxy hydroperoxides with active oxygen contents suggesting a moderate to high potential for exothermic decomposition. These reactions were deliberately run on a modest scale and products should be handled carefully.

6a: *R*_f_ = 0.46 (20% EA/Hex); ^1^H *δ* 9.75 (s, 1H), 3.80 (t, *J* = 9.6, 2H), 2.59 (td, *J* = 6.1, *J* = 3.6, 2H), 2.01 (t, *J* = 3.6, 1H), 1.53 (s, 6H), 1.47 (s, 6H); ^13^C *δ* 109.4, 106.8, 80.4, 74.3, 51.2, 23.5, 20.9; IR: 3293, 2997, 2946, 1367, 1196, 1177, 1141, 1045, 834, 636; HRMS (ESI^+^, TOF) calcd for C_9_H_16_NaO_5_ [M + Na]^+^: 241.1052; found: 241.1052.

6: *R*_f_ = 0.53 (20% EA/Hex); ^1^H *δ* 8.46 (s, 1H), 3.65 (t, *J* = 5.4, 2H), 2.50 (td, *J* = 6.0, *J* = 2.6, 2H), 2.09 (t, *J* = 2.6, 1H), 1.49 (s, 6H); ^13^C *δ* 105.7, 83.7, 69.8, 60.0, 22.6, 20.5; IR: 3427, 3287, 2995, 2947, 1367, 1201, 1158, 1051, 816, 639; HRMS (ESI^+^, TOF) calcd for C_7_H_12_NaO_3_ [M + Na]^+^: 167.0684; found: 167.0681.

#### 6-((2-Hydroperoxypropan-2-yl)oxy)-1-hexyne (7)

By a similar procedure as described above, a mixture of 5-hexyn-1-ol (0.88 mL, 7.98 mmol, 3 equiv.) in CH_2_Cl_2_ (30 mL) and 2,3-dimethyl-2-butene (0.32 mL, 2.69 mmol) were reacted with O_3_/O_2_ to generate, after careful concentration and chromatography 328.6 mg (71%) of hydroperoxyacetal 7 as a colorless oil: *R*_f_ = 0.41 (20% EA/Hex); ^1^H *δ* 7.89 (s, 1H), 3.51 (t, *J* = 6.5, 2H), 2.22 (td, *J* = 6.7, *J* = 2.6, 2H), 1.95 (t, *J* = 2.6, 1H), 1.73–1.57 (m, 4H), 1.39 (s, 6H); ^13^C *δ* 105.3, 84.5, 68.7, 61.2, 29.1, 25.3, 22.8, 18.3; IR: 3296, 2993, 2943, 2872, 2117, 1740, 1366, 1199, 1151, 1061, 866, 629; HRMS (ESI^+^, TOF) calcd for C_9_H_16_NaO_3_ [M + Na]^+^: 195.0997; found: 195.0995.

***4-((2-Methylperoxypropan-2-yl)oxy)-1-butyne (*** *8* ***)*** was prepared using an adaptation of a reported procedure.^21,57^ To a solution of hydroperoxyacetal 6 (359 mg, 2.49 mmol) in THF (10 mL) was added potassium *tert*-butoxide (350 mg, 3.12 mmol, 1.2 equiv.), followed by methyl iodide (0.19 mL, 3.05 mmol, 1.2 equiv.). After the reaction had stirred at room temperature for 5 minutes, it was quenched with water (10 mL) and the resulting mixture extracted with ether (15 mL × 3). The combined organic layers were dried with Na_2_SO_4_ and concentrated under reduced pressure. The residue was purified by column chromatography (10% ether/pentane) to yield 262.2 mg (66%) of peroxyacetal 8 as a colorless oil: *R*_f_ = 0.54 (20% EA/Hex); ^1^H *δ* 3.85 (s, 3H), 3.66 (t, *J* = 9.8, 2H), 2.47 (td, *J* = 9.8, *J* = 3.6, 2H), 1.97 (t, *J* = 3.6, 1H), 1.40 (s, 6H); ^13^C *δ* 104.9, 81.5, 69.4, 63.4, 60.2, 23.2, 20.3; IR: 2992, 2943, 2894, 1379, 1200, 1152, 1042, 864; HRMS (ESI^+^, TOF) calcd for C_8_H_14_NaO_3_ [M + Na]^+^: 181.0814; found: 181.0841.

#### 6-((2-Methylperoxypropan-2-yl)oxy)-1-hexyne (9)

By a similar procedure as described for above, a solution of hydroperoxyacetal 7 (146 mg, 0.85 mmol) in THF (5 mL) was reacted with potassium *tert*-butoxide (114 mg, 1.02 mmol, 1.2 equiv.) and methyl iodide (0.10 mL, 1 mmol, 1.2 equiv.), to furnish, after purification, 142.5 mg (90%) of peroxyacetal 9 as an colorless oil: *R*_f_ = 0.45 (20% EA/Hex); ^1^H *δ* 3.84 (s, 3H), 3.53 (t, *J* = 8.3, 2H), 2.23 (td, *J* = 9.3, *J* = 3.4, 2H), 1.93 (t, *J* = 3.1, 1H), 1.71–1.59 (m, 4H), 1.38 (s, 6H); ^13^C *δ* 104.7, 84.6, 68.4, 63.3, 61.0, 29.1, 25.4, 23.5, 23.2, 18.3; IR: 3294, 2943, 2117, 1365, 1205, 1153, 1067, 1015, 869, 629; HRMS (ESI^+^, TOF) calcd for C_10_H_18_NaO_3_ [M + Na]^+^: 209.1154; found: 209.1153.

#### 1-Iodo-4-((2-methylperoxypropan-2-yl)oxy)-1-butyne (10)

By a similar procedure as employed for synthesis of iodoalkynyl peroxide 3, reaction of peroxyacetal 8 (141 mg, 0.89 mmol) in MeOH (3 mL) with KI (183 mg, 1.10 mmol, 1.2 equiv.) followed by TBHP as an ∼5.5 M solution in decane (0.25 mL, 1.38 mmol, 1.2 equiv.) furnished, after concentration under reduced pressure. 206.7 mg (81%) of iodoalkynyl peroxyacetal 10 as a light yellow oil: *R*_f_ = 0.54 (10% EA/Hex); ^1^H *δ* 3.85 (s, 3H), 3.64 (t, *J* = 7.3, 2H), 2.64 (t, *J* = 7.4, 2H), 1.39 (s, 6H); ^13^C *δ* 104.9, 91.5, 63.4, 60.2, 23.2, 22.6, −5.4; IR: 2992, 2940, 2891, 1366, 1199, 1152, 1073, 1013, 862; HRMS (ESI^+^, TOF) calcd for C_8_H_13_INaO_2_ [M + Na]^+^: 306.9807; found: 306.9811.

#### 1-Iodo-6-((2-methylperoxypropan-2-yl)oxy)-1-hexyne (11)

By a similar procedure as employed for synthesis of 3, reaction of peroxyacetal 9 (242 mg, 1.30 mmol) in MeOH (10 mL) with KI (267 mg, 1.61 mmol, 1.2 equiv.) and TBHP as an ∼5.5 M solution in decane (0.36 mL, 1.98 mmol, 1.5 equiv.) furnished iodoalkynyl peroxyacetal 11 (400.8 mg, 98%) of as a light yellow oil: *R*_f_ = 0.49 (10% EA/Hex); ^1^H *δ* 3.85 (s, 3H), 3.52 (t, *J* = 6.5, 2H), 2.40 (t, *J* = 6.8, 2H), 1.69–1.54 (m, 4H), 1.38 (s, 6H); ^13^C *δ* 104.7, 94.7, 63.3, 61.0, 29.2, 25.4, 23.2, 20.8, −7.1; IR: 3299, 2940, 1466, 1378, 1365, 1204, 1152, 1013, 858, 632; HRMS (ESI^+^, TOF) calcd for C_10_H_17_INaO_2_ [M + Na]^+^: 335.0120; found: 335.0126.

### Synthesis of peroxy azides

#### 1-Azido-(4-(*tert*-butylperoxy))butane (12)

To a solution of 1-bromo-4-chlorobutane (2.57 g, 15.0 mmol) in DMF (40 mL) was added sodium azide (1.96 mg, 30.1 mmol, 2 equiv.). The reaction was stirred at room temperature for 24 hours. The reaction was diluted with (20 mL) water and extracted with hexanes (15 mL × 3). The combined organic layers were dried with Na_2_SO_4_ and the residue concentrated under reduced pressure to yield 1.8975 g (95%) of 1-azido-4-chlorobutane as a colorless oil which was used without further purification: *R*_f_ = 0.49 (10% EA/Hex). Spectral details matched those previously reported.^22^*R*_f_ = 0.49 (10% EA/Hex).

A solution of the 1-azido-4-chlorobutane (2.40 g, 18.0 mmol) and sodium iodide (15.0 g, 100 mmol, 5.5 equiv.) was refluxed in acetone (150 mL) for 18 hours. The reaction was then diluted with water (60 mL) and extracted with hexanes (50 mL × 3). The combined organic layers were dried with Na_2_SO_4_ and the residue concentrated under reduced pressure to yield 3.6943 g (91%) of 1-azido-4-iodobutane as an orange oil which was used without further purification. Spectral details matched those previously reported.^22^*R*_f_ = 0.5 (10% EA/Hex); ^1^H *δ* 3.32 (t, *J* = 6.6, 2H), 3.20 (t, *J* = 6.7, 2H), 1.91 (p, *J* = 7.4, 2H), 1.71 (p, *J* = 7.4, 2H); ^13^C *δ* 50.5, 30.6, 30.0, 7.2.

By a similar procedure as applied for synthesis of peroxide 1, reaction of CsOH monohydrate (127 mg, 0.85 mmol, 1.2 equiv.) in DMF (12 mL) at 0 °C, TBHP as an ∼5.5 M solution in decane (0.19 mL, 1.05 mmol, 1.5 equiv., ∼5.5 M solution in decane), and 1-azido-4-iodobutane (158 mg, 0.70 mmol) furnished, after purification by chromatography (5% EA/Hex), 80.9 mg (62%) of the azidobutyl *t*-butyl peroxide (12) as a colorless oil (CAUTION: low molecular weight azide/peroxide): *R*_f_ = 0.51 (10% EA/Hex); ^1^H *δ* 3.96 (m, 2H), 3.30 (t, *J* = 6.4, 2H), 1.71–1.66 (m, 4H), 1.24 (s, 9H); ^13^C *δ* 80.3, 74.7, 51.4, 26.5, 26.0, 25.4; IR: 2977, 2931, 2093, 1456, 1362, 1245, 1196, 881; HRMS (ESI^+^, TOF) calcd for C_8_H_17_N_3_NaO_2_ [M + Na]^+^: 210.1218; found: 210.1213.

#### ((6-Azidohexan-2-yl)peroxy)triethylsilane (13)

Into a RBF containing CH_2_Cl_2_ (300 mL), were added sequentially iodine (7.61 g, 30.0 mmol), PPh_3_ (7.87 g, 30.0 mmol) and imidazole (2.04 g, 30.0 mmol). The mixture was stirred for 15 minutes at 0 °C, followed by the addition of 5-hexenol (2.50 g, 25.0 mmol). Reaction was stirred at room temperature and filtered through silica with hexanes (600 mL). The filtrate was concentrated under reduced pressure to yield 3.8557 g (75%) of 6-iodohexene as a light orange oil which was used without further purification. Spectral details matched those previously reported.^58^*R*_f_ = 0.58 (10% EA/Hex); ^1^H *δ* 5.02 (dq, *J* = 17.0, *J* = 1.8, 1H), 4.96 (ddt, *J* = 10.0, 1.8, 0.9, 1H), 5.78 (dddd, *J* = 17.2, *J* = 10.5, *J* = 6.7, *J* = 6.7, 1H), 3.19 (t, *J* = 7.0, 2H), 2.08 (q, *J* = 7.0, 2H), 1.84 (p, *J* = 7.5, 2H), 1.50 (p, *J* = 7.5, 2H); ^13^C *δ* 138.2, 115.1, 33.0, 32.7, 29.8, 7.0.

To a solution of the iodohexene (1.20 g, 5.71 mmol) in DMF (20 mL) was added sodium azide (1.11 g, 17.1 mmol, 3 equiv.). The reaction was stirred at room temperature for 18 hours and then diluted with water (10 mL). The combined hexane extracts (3 × 15 mL) were dried with Na_2_SO_4_ and the residue concentrated under reduced pressure to yield 688.5 mg (98%) of 6-azido-hexene as a colorless oil which was used without further purification. Spectral details matched those previously reported:^59^*R*_f_ = 0.59 (10% EA/Hex); ^1^H *δ* 5.01 (dq, *J* = 17.2, *J* = 1.6, 1H), 4.97 (d, *J* = 10.2, 1H), 5.79 (dddd, *J* = 17.0, *J* = 10.5, *J* = 6.7, *J* = 6.7, 1H) 3.27 (t, *J* = 6.8, 2H), 2.09 (q, *J* = 7.1, 2H), 1.62 (p, *J* = 7.5, 2H), 1.48 (p, *J* = 7.6, 2H); ^13^C *δ* 138.3a 115.1, 51.5, 33.3, 28.4, 26.0.

To a solution of the azidohexene (113 mg, 0.90 mmol) in EtOH (10 mL) was added triethylsilane (0.29 mL, 1.82 mmol, 2 equiv.), followed by Co(acac)_2_ (25.7 mg, 0.10 mmol, 0.1 equiv.). The reaction was stirred at room temperature under O_2_ for 18 hours.^23^ The solution was concentrated under reduced pressure and the residue purified by column chromatography (5% EA/Hex) to yield 114.0 mg (46%) of azido peroxide 13 as a colorless oil: *R*_f_ = 0.5 (10% EA/Hex); ^1^H *δ* 4.01 (s, *J* = 5.8, 1H), 3.27 (t, *J* = 6.9, 2H), 1.61–1.40 (m, 6H), 1.20 (d, *J* = 6.2, 3H), 0.99 (t, *J* = 7.9, 9H), 0.69 (q, *J* = 8.0, 6H); ^13^C *δ* 81.2, 51.5, 34.0, 29.1, 22.8, 18.5, 6.9, 3.9; IR; 2938, 2877, 2093, 1459, 1242, 1006, 796, 727; HRMS (ESI^+^, TOF) calcd for C_12_H_27_N_3_NaO_2_Si [M + Na]^+^: 296.1770; found: 296.1772.

#### Hexyne (14) was used as received

##### Hex-5-yn-1-yl benzene (15) (100848-88-2)

Alkyne 15 was prepared using a variant of a reported procedure.^60^ To a solution of ethynyl trimethylsilane (998.3 mg, 3.84 mmol) in THF (10 mL) was added *n*-BuLi (2.7 mL, 4.32 mmol, 1.1 equiv.) at −78 °C, followed by 4-(iodobutyl)benzene (386.2 mg, 3.93 mmol, 1.02 equiv.). The reaction was allowed to warm to room temperature and stirred for 4 hours. The reaction was quenched with water (30 mL) and the combined ethyl acetate extracts (3 × 30 mL) were dried with Na_2_SO_4_. Concentration under reduced pressure yielded 825.2 mg (85%) of trimethyl(6-phenylhex-1-yn-1-yl)silane (2253948-27-3) as a colorless oil which was used directly in the following reaction. *R*_f_ = 0.60 (10% EA/Hex). Spectra details matched those in a literature report.^61^

To a solution of the crude alkynyl silane (743.2 mg, 3.09 mmol) in methanol (25 mL) was added potassium hydroxide (304.0 mg, 5.42 mmol, 1.75 equiv.). The reaction was stirred at room temperature for 2 hours.^62^ The reaction was quenched with water (40 mL) and extracted with hexanes (30 mL × 3). The combined organic layers were dried with Na_2_SO_4_, concentrated under reduced pressure and the residue purified by column chromatography (5% EA/Hex) to yield 499.0 mg (89%) of the hex-5-yn-1-yl benzene as a colorless oil. Spectral details matched those previously reported:^61,63^*R*_f_ = 0.65 (10% EA/Hex); ^1^H *δ* 7.31 (m, 2H), 7.24–7.21 (m, 3H), 2.67 (t, *J* = 7.9, 2H), 2.25 (td, *J* = 2.6, *J* = 7.1, 2H), 1.98 (t, *J* = 2.6, 1H), 1.78 (p, *J* = 7.8, 2H), 1.61 (p, 7.5, 2H); ^13^C *δ* 142.4, 128.5, 128.4, 125.9, 84.5, 68.4, 35.5, 30.5, 28.1, 18.4; IR: 3305, 2927, 2854, 1495, 1453, 744, 696.

#### 3-Azidopropylbenzene (16)

Iodine (11.74 g, 46.3 mmol), PPh_3_ (11.89 g, 45.3 mmol) and imidazole (3.16 g, 46.4 mmol) were sequentially added to a solution of CH_2_Cl_2_ (300 mL). The mixture was stirred for 15 minutes at 0 °C, whereupon 3-phenyl-1-propanol (5.07 g, 37.2 mmol) was added. The reaction was stirred at room temperature for 5 hours and filtered through a ∼3′′ pad of silica with hexanes (600 mL). The filtrate was concentrated under reduced pressure to yield 9.05 g (98%) of 3-iodopropylbenzene as a light orange oil which was used without further purification. Spectral details matched those previously reported.^64^*R*_f_: 0.50 (10% EA/Hex); ^1^H *δ* 7.33 (t, *J* = 7.4, 2H), 7.26 (t, *J* = 8.1, 1H), 7.24 (d, *J* = 6.6, 2H), 3.21 (t, *J* = 6.8, 2H), 2.77 (t, *J* = 7.3, 2H), 2.17 (p, *J* = 7.2, 2H); ^13^C *δ* 140.5, 128.7, 128.6, 126.3, 36.4, 35.0, 6.5.

To a solution of the iodopropyl benzene (1.10 g, 4.48 mmol) in DMF (20 mL) was added sodium azide (875.6 g, 13.4 mmol, 3 equiv.). The reaction was stirred at room temperature for 18 hours. The reaction was diluted with water (10 mL) and extracted with CH_2_Cl_2_ (15 mL × 3). The combined organic layers were dried with Na_2_SO_4_ and the residue concentrated under reduced pressure to yield 694.1 mg (96%) of 3-azidopropylbenzene as a colorless oil. Spectral details matched those previously reported.^65^*R*_f_: 0.52 (10% EA/Hex); ^1^H *δ* 7.34 (t, *J* = 7.4, 2H), 7.26 (t, *J* = 7.4, 1H), 7.22 (d, *J* = 7.3, 2H), 3.32 (t, *J* = 6.8, 2H), 2.75 (t, *J* = 7.5, 2H), 1.95 (p, *J* = 7.2, 2H); ^13^C *δ* 141.0, 128.6, 128.6, 126.3, 50.8, 32.9, 30.6.

#### 4-Azidobutylbenzene (17)

Using a similar procedure as described above, reaction of a solution of iodine (7.18 g, 28.3 mmol), PPh_3_ (7.40 g, 28.2 mmol), imidazole (1.91 g, 28.1 mmol) and 4-phenyl-1-butanol (3.47 g, 23.1 mmol) in CH_2_Cl_2_ (300 mL) furnished 4.6282 g (77%) of 4-iodobutylbenzene as a light orange oil. Spectral details matched those previously reported.^66^*R*_f_ = 0.51 (10% EA/Hex); ^1^H *δ* 7.32 (dt, *J* = 5.4, *J* = 1.5, 2H), 7.24 (dt, *J* = 8.4, 1.2, 1H), 7.21 (d, *J* = 6.8, 2H), 3.23 (t, *J* = 6.8, 2H), 2.67 (t, *J* = 7.6, 2H), 1.89 (m, 2H), 1.77 (m, 2H); ^13^C *δ* 141.9, 128.5, 126.0, 34.9, 33.1, 32.3, 6.9.

Using a similar procedure as described above, reaction of the 4-iodobutylbenzene (1.25 g, 4.81 mmol) with sodium azide (969 mg, 14.9 mmol, 3 equiv.) in DMF (20 mL) furnished 811.7 mg (97%) of 4-azidobutylbenzene (17) as a colorless oil. Spectral details matched those previously reported.^66^*R*_f_ = 0.53 (10% EA/Hex); ^1^H *δ* 7.32 (t, *J* = 7.3, 2H), 7.22 (t, *J* = 7.3, 1H), 7.21 (d, *J* = 7.3, 2H), 3.31 (t, *J* = 6.5, 2H), 2.68 (t, *J* = 7.6, 2H), 1.75 (m, 2H), 1.67 (m, 2H); ^13^C *δ* 142.0, 128.5, 128.5, 126.0, 51.5, 35.5, 28.6.

#### 6-Azidohexane-1-thiol (18)

This molecule, which is commercially available, has been previously described without characterization.^67^

Synthesis of *S*-(6-hydroxyhexyl)ethanethioate was adapted from a reported procedure.^67^ To a solution of 6-bromohexanol (3.07 g, 17.0 mmol) in acetone (175 mL) was added potassium thioacetate (3.92 g, 34.3 mmol, 2 equiv.). The reaction was stirred at 40 °C for 18 hours. The reaction was then quenched with 1 M NaHCO_3_ (50 mL) and the mixture extracted with ether (50 mL × 3). The combined organic layers were dried with Na_2_SO_4_, concentrated under reduced pressure, and the residue was purified by column chromatography (5–20% ether/pentane) to yield 1.89 g (63%) of the thioester as a red oil. Spectral details matched those previously reported.^68^*R*_f_ = 0.30 (30% EA/Hex); ^1^H *δ* 3.62 (t, *J* = 6.4, 2H), 2.86 (t, *J* = 6.8, 2H), 2.31 (s, 3H), 1.53–1.62 (m, 4H), 1.35–1.43 (m, 4H); ^13^C *δ* 196.2, 62.9, 32.7, 30.8, 29.6, 29.1, 28.6, 25.3; IR: 3292, 2944, 1380, 1205, 1157, 1064, 831, 630.

The following reagents were sequentially added to CH_2_Cl_2_ (300 mL): iodine (2.86 g, 11.3 mmol, 1.2 equiv.), PPh_3_ (2.96 g, 11.3 mmol, 1.2 equiv.) and imidazole (769.3 mg, 11.3 mmol, 1.2 equiv.). The mixture was stirred for 15 minutes at 0 °C, whereupon *S*-(6-hydroxyhexyl)ethanethioate (1.66 g, 9.42 mmol) was added. The reaction was stirred at room temperature for 5 hours and filtered through a 7.5 cm pad of silica with hexanes (600 mL). The filtrate was concentrated under reduced pressure to yield 1.98 g (73%) of *S*-(6-iodohexyl)ethanethioate as a brown oil which was used directly for the next reaction: *R*_f_ = 0.40 (5% EA/Hex); ^1^H *δ* 3.20 (t, *J* = 7.0, 2H), 2.89 (t, *J* = 7.3, 2H), 2.35 (s, 3H), 1.84 (p, *J* = 6.8, 2H), 1.61 (p, *J* = 6.9, 2H), 1.37–1.47 (m, 4H); ^13^C *δ* 196.1, 33.4, 30.8, 30.1, 29.4, 29.1, 27.8, 7.0; IR: 2929, 1686, 1132, 954, 623; HRMS (ESI^+^, TOF) calcd for C_8_H_15_IOS [M]^+^: 285.9888; found: 285.9898.

To a solution of *S*-(6-azidohexyl)ethanethioate (1.16 g, 4.05 mmol) in DMF (20 mL) was added sodium azide (1.45 g, 22.3 mmol, 5.5 equiv.). The reaction was stirred at room temperature for 18 hours. The reaction was diluted with water (10 mL) and extracted with CH_2_Cl_2_ (15 mL × 3). The combined organic layers were dried with Na_2_SO_4_ and the residue concentrated under reduced pressure to yield 762.6 mg (94%) of azido/ethioester as an orange oil which was used without further purification. Spectral details matched those previously reported.^69^*R*_f_ = 0.32 (5% EA/Hex); ^1^H *δ* 3.25 (t, *J* = 7.1, 2H), 2.86 (t, *J* = 7.1, 2H), 2.32 (s, 3H), 1.54–1.61 (m, 4H), 1.37–1.40 (m, 4H); ^13^C *δ* 196.0, 51.5, 30.8, 29.5, 29.0, 28.8, 28.4, 26.3; IR: 2933, 2090, 1688, 1256, 1132, 951, 624.

The deblocking of the thioacetate employed a procedure reported for a nine-carbon homolog.^70^ To a solution of *S*-(6-azidohexyl)ethanethioate (762.6 g, 3.79 mmol) in MeOH (70 mL) was added conc. aq. HCl (4.0 mL). The reaction was stirred at reflux for 3 hours and then allowed to cool. The reaction was diluted with water (20 mL) and extracted with diethyl ether (25 mL × 3). The combined organic layers were dried with Na_2_SO_4_ and the residue concentrated under reduced pressure to yield 559.8 mg (93%) of 6-azidohexane-1-thiol as a strong-smelling orange oil. *R*_f_ = 0.35 (5% EA/Hex); ^1^H *δ* 3.27 (t, *J* = 6.6, 2H), 2.53 (q, *J* = 7.2, 2H), 1.57–1.64 (m, 4H), 1.37–1.43 (m, 4H); IR: 3930, 3857, 2400 (w); 2086 (s); 1454, 1253; ^13^C *δ* 51.5, 33.9, 28.9, 28.0, 26.3, 24.6 IR: 2930, 2087, 1254; HRMS (ESI^+^, TOF) calcd for C_6_H_13_N_3_S [M]^+^: 159.0830; found: 159.0867, 316.1512 (disulfide).

### General procedure for click reactions

Click reactions were conducted based upon adaptations of a reported procedure.^25*b*^

#### General procedure for click reactions with CuI

To a solution of alkyne (1 equiv.) and azide (1 equiv.) in THF was added CuI (0.2 equiv.), followed by triethylamine (3 equiv.). The reaction was stirred at room temperature for 3 hours, then diluted with water and extracted with ethyl acetate (×3). The combined organic layers were dried with Na_2_SO_4_, concentrated under reduced pressure and the residue purified by column chromatography.

#### General procedure for click reactions with CuSO_4_

To a solution of alkyne (1 equiv.) and azide 16 (1 equiv.) in CH_2_Cl_2_ was added an aqueous solution containing CuSO_4_ (0.2 equiv.) and sodium ascorbate (0.2 equiv.). After stirring at rt for 3 h, the reaction was diluted with water and extracted with ethyl acetate (×3). The combined organic layers were dried with Na_2_SO_4_, concentrated under reduced pressure and the residue purified by column chromatography.

#### 4-(4-(*tert*-Butylperoxy)butyl)-1-(3-phenylpropyl)-1*H*-1,2,3-triazole (19)

To a solution of peroxyhexyne 1 (203 mg, 1.19 mmol) and azide 16 (193 mg, 1.20 mmol) in THF (5 mL) was added CuI (47.6 mg, 0.25 mmol, 0.2 equiv.),^25*b*^ followed by triethylamine (0.50 mL, 3.6 mmol, 3 equiv.) and triethylsilane (0.21 mL, 1.3 mmol, 1.1 equiv.). The reaction was stirred at room temperature for 24 hours. The reaction was diluted with water (10 mL) and extracted with EA (15 mL × 3). The combined organic layers were dried with Na_2_SO_4_, concentrated under reduced pressure and the residue purified by column chromatography (10% EA/Hex) to yield 156.2 mg (38%) of triazole 19 as a yellow oil: *R*_f_ = 0.28 (40% EA/Hex); ^1^H *δ* 7.29 (t, *J* = 7.4, 2H), 7.26 (s, 1H), 7.23 (t, *J* = 7.4, 1H), 7.19 (d, *J* = 7.1, 2H), 4.33 (t, *J* = 7.1, 2H), 3.99 (t, *J* = 6.3, 2H), 2.77 (t, *J* = 7.4, 2H), 2.67 (t, *J* = 7.5, 2H), 2.25 (p, *J* = 7.3, 2H), 1.78 (m, 2H), 1.69 (m, 2H), 1.25 (s, 9H); ^13^C *δ* 148.0, 140.4, 128.7, 128.5, 126.4, 120.7, 80.2, 74.8, 49.5, 32.7, 31.8, 27.6, 26.5, 26.2, 25.6; IR: 2976, 2932, 2866, 1497, 1454, 1362, 1196, 1046, 878, 746, 670, 493; HRMS (ESI^+^, TOF) calcd for C_19_H_29_N_3_NaO_2_ [M + Na]^+^: 354.2157; found: 354.2155.

#### 4-(4-(*tert*-Butylperoxy)butyl)-1-(4-phenylbutyl)-1*H*-1,2,3-triazole (20) and 4-(4-(*tert*-butylperoxy)butyl)-5-iodo-1-(4-phenylbutyl)-1*H*-1,2,3-triazole (20i)

To a solution of 6-(*tert*-butylperoxy)-1-hexyne (238 mg, 1.40 mmol) and 4-azidobutylbenzene (209 mg, 1.19 mmol) in THF (5 mL) was added CuI (47.6 mg, 0.25 mmol, 0.2 equiv.), followed by triethylamine (0.60 mL, 4.29 mmol, 3 equiv.).^46^ The reaction was stirred at room temperature for 1 hour. The reaction was diluted with water (10 mL) and extracted with EA (15 mL × 3). The combined organic layers were dried with Na_2_SO_4_, concentrated under reduced pressure and the residue purified by column chromatography (10% EA/Hex) to yield 154.4 mg (37%) of triazole 20 as a yellow oil. Reactions conducted in the presence of CuI often contained traces of the corresponding 5-iodotriazole (20i).

20: *R*_f_ = 0.27 (40% EA/Hex); ^1^H *δ* 7.29 (t, *J* = 7.4, 2H), 7.24 (s, 1H), 7.21 (t, *J* = 7.4, 1H), 7.16 (d, *J* = 7.2, 2H), 4.33 (t, *J* = 7.2, 2H), 3.98 (t, *J* = 6.1, 2H), 2.75 (t, *J* = 7.2, 2H), 2.66 (t, *J* = 7.5, 2H), 1.93 (p, *J* = 7.5, 2H), 1.80–1.62 (m, 6H), 1.25 (s, 9H); ^13^C *δ* 150.0, 141.6, 128.5, 128.5, 126.1, 120.6, 80.2, 74.8, 50.1, 35.3, 29.9, 28.3, 27.6, 26.4, 26.2, 25.6 ppm; IR: 3026, 2934, 1454, 1362, 1196, 1044, 747, 699; HRMS (ESI^+^, TOF) calcd for C_20_H_31_N_3_NaO_2_ [M + Na]^+^: 368.2314; found: 368.2312.

20i: *R*_f_ = 0.31 (40% EA/Hex); ^1^H *δ* 7.21 (t, *J* = 7.2, 2H), 7.18 (m, 3H), 4.37 (t, *J* = 7.2, 2H), 3.99 (t, *J* = 6.4, 2H), 2.70 (t, *J* = 7.2, 2H), 2.68 (t, *J* ∼ 7, 2H), 1.95 (p, *J* = 7.2, 2H), 1.80 (m, 2H), 1.76 (m, 4H), 1.25 (s, 9H); ^13^C *δ* 151.60, 141.7, 128.58, 128.55, 126.1, 120.6, 80.3, 78.1, 74.8, 50.6, 35.3, 29.5, 28.2, 27.6, 26.4, 26.1, 25.8 ppm; IR: 2976, 2936, 1454, 1362, 1197, 1044, 743, 699; HRMS (ESI^+^, TOF) calcd for C_20_H_30_IN_3_NaO_2_ [M + Na]^+^: 494.1280; found: 494.1276.

#### 4-(1-(4-Phenylbutyl)-1*H*-1,2,3-triazol-4-yl)butanal (21)

This colorless oil, which coelutes with the parent peroxide, was a minor byproduct in many of the click reactions but became a major byproduct for reactions conducted for long periods or when the peroxy triazole products were resubjected to reaction conditions: *R*_f_ = 0.27 (40% EA/Hex); ^1^H *δ* 9.79 (t, *J* = 0.7, 1H), 7.32–7.16 (m, 6H), 4.34 (t, *J* = 7.2, 2H), 2.78 (t, *J* = 7.4, 2H), 2.68 (t, *J* = 7.5, 2H), 2.55 (td, *J* = 1.4, *J* = 7.3, 2H), 2.04 (p, *J* = 7.3, 2H), 1.94 (p, *J* = 7.6, 2H), 1.67 (m, 2H); ^13^C (176 MHz) *δ* 202.2, 147.2, 141.6, 128.6, 128.5, 126.2, 120.8, 50.2, 43.3, 35.3, 32.1, 28.3, 27.2, 21.9; IR: 3135, 3025, 2925, 2723, 1722, 1454, 1275, 748, 701; HRMS (ESI^+^, TOF) calcd for C_20_H_31_N_3_NaO_2_ [M + Na]^+^: 294.1582; found: 294.1588.

### Stability of peroxyalkyne towards CuI

To 6-(*tert*-butylperoxy)-1-hexyne 1 (170 mg, 1.0 mmol) in THF (5 mL) was added CuI (38 mg, 0.20 mmol, 0.2 equiv.). The reaction was stirred at room temperature for 24 hours. The reaction was quenched with water and extracted with ethyl acetate (15 mL × 3). The combined organic layers were dried with Na_2_SO_4_, concentrated under reduced pressure to yield 153.4 mg (90%) of recovered starting material as a colorless oil: *R*_f_ = 0.55 (10% EA/Hex).

### Reactions in the presence of TBTA

#### CuSO_4_

To 6-(*tert*-butylperoxy)-1-hexyne 1 (272 mg, 1.6 mmol) and 4-phenyl-butyl-azide 17 (228 mg, 1.3 mmol) in 1 : 1 DMSO/water (10 mL) was added CuSO_4_ (25.5 mg, 0.16 mmol, 0.1 equiv.), sodium ascorbate (47.5 mg, 0.24 mmol, 0.15 equiv.), and TBTA (8.4 mg, 0.016 mmol, 0.01 equiv.). The reaction was stirred at room temperature for 1.5 hours. The reaction was quenched with water and extracted with ethyl acetate (15 mL × 3). The combined organic layers were dried with Na_2_SO_4_, concentrated under reduced pressure and purified by column chromatography (10% EA/Hex) to yield 89.9 mg (20%) of triazole 20 as a yellow oil: *R*_f_ = 0.28 (40% EA/Hex).

#### CuI

To 6-(*tert*-butylperoxy)-1-hexyne 1 (238 mg, 1.4 mmol) and azide 17 (245 mg, 1.4 mmol) in DMSO : water (2.5 mL : 2.5 mL) was added CuI (57 mg, 0.30 mmol, 0.2 equiv.) and triethylamine (0.6 mL, 4.2 mmol, 3 equiv.), followed by TBTA (7.4 mg, 0.014 mmol, 0.01 equiv.). The reaction was stirred at room temperature for 3 hours. The reaction was quenched with water and extracted with ethyl acetate (15 mL × 3). The combined organic layers were dried with Na_2_SO_4_, concentrated under reduced pressure and purified by column chromatography (10% EA/Hex) to yield 166.3 mg (35%) of triazole 20 as a yellow oil: *R*_f_ = 0.28 (40% EA/Hex).

### Reaction of iodoalkyne

#### 4-(4-(*tert*-Butylperoxy)butyl)-5-iodo-1-(3-phenylpropyl)-1*H*-1,2,3-triazole (19i)

To a solution of 1-iodo-6-*tert*-butylperoxy hexyne 3 (118 mg, 0.40 mmol) and 3-azidopropylbenzene 16 (64.5 mg, 0.41 mmol) in THF (3.5 mL) was added CuI (7.6 mg, 0.04 mmol, 0.1 equiv.), followed by triethylamine (0.12 mL, 0.86 mmol, 2 equiv.).^12^ The reaction was stirred at room temperature for 24 hours. The reaction was diluted with water (10 mL) and extracted with EA (15 mL × 3). The combined organic layers were dried with Na_2_SO_4_, concentrated under reduced pressure and the residue purified by column chromatography (10% EA/Hex) to yield 116.4 mg (64%) of iodotetrazole 19i as a yellow oil: *R*_f_ = 0.36 (20% EA/Hex); ^1^H *δ* 7.32 (t, *J* = 7.5, 2H), 7.24 (t, *J* = 7.5, 1H), 7.22 (d, *J* = 7.6, 2H), 4.37 (t, *J* = 7.3, 2H), 3.99 (t, *J* = 6.4, 2H), 2.70 (td, *J* = 7.5, *J* = 3.4, 4H), 2.25 (p, *J* = 7.4, 2H), 1.80 (p, *J* = 7.6, 2H), 1.68 (p, *J* = 6.3, 2H), 1.25 (s, 9H); ^13^C *δ* 151.6, 140.4, 128.7, 128.6, 126.4, 80.2, 78.1, 74.7, 50.1, 32.6, 31.3, 27.6, 26.5, 26.0, 25.8; IR: 3031, 2936, 2098, 1461, 1361, 1196 1022, 880, 752, 699, 509; HRMS (ESI^+^, TOF) calcd for C_19_H_28_IN_3_NaO_2_ [M + Na]^+^: 480.1124; found: 480.1126.

### Competition of iodoalkynyl peroxide with simple alkyne

To a solution of 6-(*tert*-butylperoxy)-1-iodohexyne 3 (0.5 mmol), 5-hexyn-1-ol (0.5 mmol) and 3-phenylpropyl azide 16 (0.5 mmol) in THF (5 mL) was added CuI (0.050 mmol, 0.1 equiv.), followed by triethylamine (1.0 mmol, 2 equiv.). The reaction was stirred at room temperature for 18 hours. The reaction was quenched with water and extracted with ethyl acetate (15 mL × 3). The combined organic layers were dried with Na_2_SO_4_, concentrated under reduced pressure and purified by column chromatography (10% EA/Hex) to yield 64.5 mg (29%) of 4-(4-(*tert*-butylperoxy)butyl)-5-iodo-1-(3-phenylpropyl)-1*H*-1,2,3-triazole (19i) as a yellow oil.

#### 4-(6-(*tert*-Butylperoxy)hexyl)-1-(3-phenylpropyl)-1*H*-1,2,3-triazole (6/3) (22) and 4-(6-(*tert*-butylperoxy)hexyl)-5-iodo-1-(3-phenylpropyl)-1*H*-1,2,3-triazole and 6-(1-(3-phenylpropyl)-1*H*-1,2,3-triazol-4-yl)hexanal (24)

To a solution of 8-(*tert*-butylperoxy)-1-octyne 2 (304.1 mg, 1.53 mmol) and 3-azidopropylbenzene 16 (268.4 mg, 1.66 mmol, 1.1 equiv.) in THF (6 mL) was added CuI (64.8 mg, 0.25 mmol, 0.2 equiv.), followed by triethylamine (0.65 mL, 4.66 mmol, 3 equiv.).^3^ The reaction was stirred at room temperature for 3 hours. The reaction was diluted with water (10 mL) and extracted with EA (15 mL × 3). The combined organic layers were dried with Na_2_SO_4_, concentrated under reduced pressure and the residue purified by column chromatography (10% EA/Hex) to yield 374.3 mg (68%) of peroxy triazole 22, 45.9 mg (6%) of iodotriazole 22i and 47.1 mg (11%) of aldehyde 24.

22: *R*_f_: 0.28 (40% EA/Hex); ^1^H *δ* 7.31 (t, *J* = 7.5, 2H), 7.24 (s, 1H), 7.22 (t, *J* = 7.6, 1H), 7.19 (d, *J* = 7.4, 2H), 4.33 (t, *J* = 7.1, 2H), 3.94 (t, *J* = 6.5, 2H), 2.73 (t, *J* = 7.6, 2H), 2.66 (t, *J* = 7.5, 2H), 2.25 (p, *J* = 7.3, 2H), 1.69 (p, *J* = 7.4, 2H), 1.61 (p, *J* = 6.8, 2H), 1.36–1.45 (m, 4H), 1.25 (s, 9H); ^13^C *δ* 148.4, 140.4, 128.7, 128.5, 126.4, 120.6, 80.2, 75.1, 49.4, 32.7, 31.8, 29.5, 29.2, 27.9, 26.5, 26.1, 25.7; IR: 2976, 2933, 2859, 1454, 1361, 1197, 1047; HRMS (ESI^+^, TOF) calcd for C_21_H_33_N_3_NaO_2_ [M + Na]^+^: 382.2470; found: 382.2467.

22i: *R*_f_: 0.32 (40% EA/Hex); ^1^H *δ* 7.31 (t, *J* = 7.3, 2H), 7.21–7.24 (m, 3H), 4.36 (t, *J* = 7.2, 2H), 3.94 (t, *J* = 6.6, 2H), 2.68 (app p, *J* = 8.1, *J* = 7.6, 4H), 2.25 (p, *J* = 7.2, 2H), 1.71 (p, *J* = 7.1, 2H), 1.61 (p, *J* = 6.6, 2H), 1.40–1.41 (m, 4H), 1.25 (s, 9H); ^13^C *δ* 151.9, 140.4, 128.7, 128.6, 126.4, 80.1, 78.0, 75.1, 50.1, 32.6, 31.3, 29.1, 29.0, 27.9, 26.5, 26.1, 26.1; IR: 2974, 2934, 2861, 1453, 1362; HRMS (ESI^+^, TOF) calcd for C_21_H_32_IN_3_NaO_2_ [M + Na]^+^: 508.1437; found: 508.1423.

24: *R*_f_: 0.27 (40% EA/Hex); ^1^H *δ* 9.76 (t, *J* = 1.69, 1H), 7.30 (t, *J* = 7.4, 2H), 7.25 (s, 1H), 7.22 (t, *J* = 7.2, 1H), 7.18 (d, *J* = 7.3, 2H), 4.32 (t, *J* = 7.2, 2H), 2.73 (t, *J* = 7.6, 2H), 2.65 (t, *J* = 7.6, 2H), 2.44 (td, *J* = 1.7, *J* = 7.3, 2H), 2.24 (p, *J* = 7.6, 2H), 1.69 (m, 2H), 1.58 (p, *J* = 6.8, 2H) 1.42 (m, 2H); ^13^C (176 MHz) *δ* 202.7, 148.4, 140.4, 128.7, 128.5, 126.4, 120.6, 62.9, 49.5, 43.9, 32.7, 31.8, 25.5, 21.9, 14.3; IR: 2928, 2856, 1720, 1453, 1212, 1047, 1029 cm^−1^; HRMS (ESI^+^, TOF) calcd for C_17_H_23_N_3_NaO [M + Na]^+^: 308.1739; found: 308.1734.

#### 4-(6-(*tert*-Butylperoxy)hexyl)-1-(3-phenylpropyl)-1*H*-1,2,3-triazole (22) *via* CuSO_4_

To a solution of 8-(*tert*-butylperoxy)-1-octyne 2 (301.6 mg, 1.52 mmol) and 3-azidopropylbenzene 16 (260.5 mg, 1.62 mmol, 1.1 equiv.) in CH_2_Cl_2_ (4 mL) was added 2 mL of an aqueous solution containing CuSO_4_ (50.7 mg, 0.32 mmol, 0.2 equiv.) and sodium ascorbate (65.4 mg, 0.33 mmol, 0.2 equiv.).^12,29^ After stirring at rt for 3 h, the reaction was diluted with water (10 mL) and extracted with EA (15 mL × 3). The combined organic layers were dried with Na_2_SO_4_, concentrated under reduced pressure and the residue purified by column chromatography (10% EA/Hex) to yield 223.9 mg (41%) of 4-(6-(*tert*-butylperoxy)hexyl)-1-(3-phenylpropyl)-1*H*-1,2,3-triazole (22) accompanied by 139.0 mg (37%) of aldehyde 24.

#### 4-(6-(*tert*-Butylperoxy)hexyl)-1-(3-phenylpropyl)-1*H*-1,2,3-triazole (22) in presence of silane

To a solution of 8-(*tert*-butylperoxy)-1-octyne 2 (263.8 mg, 1.33 mmol) and 3-azidopropylbenzene 16 (261.0 mg, 1.34 mmol, 1.0 equiv.) in THF (5 mL) was added CuI (53.1 mg, 0.28 mmol, 0.2 equiv.),^12^ followed by triethylamine (0.60 mL, 4.3 mmol, 3 equiv.) and triethylsilane (0.45 mL, 2.50 mmol, 1.9 equiv.). The reaction was stirred at room temperature for 24 hours. The reaction was diluted with water (10 mL) and extracted with EA (15 mL × 3). The combined organic layers were dried with Na_2_SO_4_, concentrated under reduced pressure and the residue purified by column chromatography (10% EA/Hex) to yield 239.0 mg (50%) of 4-(6-(*tert*-butylperoxy)hexyl)-1-(3-phenylpropyl)-1*H*-1,2,3-triazole as a yellow oil (spectra described previously).

#### 4-(6-(*tert*-Butylperoxy)hexyl)-5-iodo-1-(3-phenylpropyl)-1*H*-1,2,3-triazole (22i) from iodoalkyne 3

To a solution of 1-iodo-8-*tert*-butylperoxy octyne 4 (297.2 mg, 0.917 mmol) and 3-azidopropylbenzene 16 (159.0 mg, 0.99 mmol, 1.1 equiv.) in THF (6 mL) was added CuI (28.3 mg, 0.15 mmol, 0.2 equiv.), followed by triethylamine (0.30 mL, 2.15 mmol, 2 equiv.).^27*c*^ The reaction was stirred at room temperature for 24 hours. The reaction was diluted with water (10 mL) and extracted with EA (15 mL × 3). The combined organic layers were dried with Na_2_SO_4_, concentrated under reduced pressure and the residue purified by column chromatography (10% EA/Hex) to yield 338.4 mg (76%) of iodotriazole 22i as a yellow oil (described previously).

#### 4-(6-(*tert*-Butylperoxy)hexyl)-1-(4-phenylbutyl)-1*H*-1,2,3-triazole (23), 4-(6-(*tert*-butylperoxy)hexyl)-5-iodo-1-(4-phenylbutyl)-1*H*-1,2,3-triazole (23i) and 6-(1-(4-phenylbutyl)-1*H*-1,2,3-triazol-4-yl)hexanal (25)

To a solution of 8-(*tert*-butylperoxy)-1-octyne 2 (305.4 mg, 1.54 mmol) and 4-azidobutylbenzene 17 (277.0 mg, 1.58 mmol, 1.0 equiv.) in THF (6 mL) was added CuI (64.2 mg, 0.34 mmol, 0.2 equiv.), followed by triethylamine (0.65 mL, 4.66 mmol, 3 equiv.). The reaction was stirred at room temperature for 24 hours. The reaction was diluted with water (10 mL) and extracted with EA (15 mL × 3). The combined organic layers were dried with Na_2_SO_4_, concentrated under reduced pressure and the residue purified by column chromatography (10% EA/Hex) to yield 315.7 mg (55%) of 29.8 mg (4%) of iodotriazole 23i, 315.7 mg (55%) of triazole 23 and 134.6 mg (45%) of aldehyde 25.

23: *R*_f_: 0.28 (40% EA/Hex): ^1^H *δ* 7.30 (t, *J* = 7.3, 2H), 7.22 (s, 1H), 7.21 (t, *J* = 7.5, 1H), 7.16 (d, *J* = 7.4, 2H), 4.33 (t, *J* = 7.2, 2H), 3.94 (t, *J* = 6.6, 2H), 2.72 (t, *J* = 7.7, 2H), 2.67 (t, *J* = 7.5, 2H), 1.94 (p, *J* = 7.6, 2H), 1.58–1.72 (m, 6H), 1.40–1.41 (m, 4H), 1.26 (s, 9H); ^13^C *δ* 148.4, 141.6, 128.5, 128.5, 126.1, 120.5, 80.2, 75.1, 50.1, 35.3, 29.9, 29.5, 29.2, 28.3, 27.9, 26.5, 26.1, 25.7; IR: 2977, 2920, 2860, 1454, 1362; HRMS (ESI^+^, TOF) calcd for C_22_H_35_N_3_NaO_2_ [M + Na]^+^: 396.2627; found: 396.2612.

23i:^27^*R*_f_: 0.32 (40% EA/Hex) ^1^H *δ* 7.30 (t, *J* = 6.8, 2H), 7.22 (t, *J* = 7.2, 1H), 7.18 (d, *J* = 7.0, 2H), 4.37 (t, *J* = 7.3, 2H), 3.95 (t, *J* = 6.3, 2H), 2.64–2.70 (m, 4H), 1.96 (p, *J* = 7.0, 2H), 1.68–1.73 (m, 4H), 1.62 (p, *J* = 6.5, 2H), 1.38–1.46 (m, 4H), 1.26 (s, 9H); ^13^C *δ* 151.9, 141.6, 128.5, 128.5, 126.1, 80.2, 77.9, 75.1, 50.6, 35.2, 29.8, 29.5, 29.1, 29.0, 28.1, 27.9, 26.5, 26.1; IR: 2088, 2920, 2858, 1454, 1361; HRMS (ESI^+^, TOF) calcd for C_22_H_34_IN_3_NaO_2_ [M + Na]^+^: 522.1593; found: 522.1581.

25: *R*_f_: 0.27 (40% EA/Hex); ^1^H *δ* 9.77 (t, *J* = 1.7, 1H), 7.30 (t, *J* = 7.4, 2H), 7.22 (s, 1H), 7.21 (t, *J* = 7.4, 1H), 7.16 (d, *J* = 7.4, 2H), 4.33 (t, *J* = 7.2, 2H), 2.73 (t, *J* = 7.6, 2H), 2.67 (t, *J* = 7.6, 2H), 1.94 (p, *J* = 7.7, 2H), 1.63–1.73 (m, 4H), 1.59 (p, *J* = 6.6, 2H), 1.37–1.45 (m, 4H); ^13^C (176 MHz) *δ* 202.8, 148.4, 141.6, 128.5, 128.5, 126.1, 120.5, 62.9, 50.1, 43.9, 35.3, 32.7, 29.9, 28.3, 25.5, 21.9; IR: 2920, 2858, 1721, 1454; HRMS (ESI^+^, TOF) calcd for C_18_H_25_N_3_NaO [M + Na]^+^: 322.1895′; found: 322.1882.

#### 4-(6-(*tert*-Butylperoxy)hexyl)-1-(4-phenylbutyl)-1*H*-1,2,3-triazole (23) and 6-(1-(4-phenylbutyl)-1*H*-1,2,3-triazol-4-yl)hexanal (25)

To a solution of 8-(*tert*-butylperoxy)-1-octyne 2 (305.1 mg, 1.54 mmol) and 4-azidobutylbenzene 17 (272.3 mg, 1.55 mmol, 1.0 equiv.) in CH_2_Cl_2_ (4 mL) was added a solution of CuSO_4_ (59.2 mg, 0.37 mmol, 0.2 equiv.) and sodium ascorbate (75.5 mg, 0.38 mmol, 0.2 equiv.) in water (2 mL).^12^ The reaction was stirred at room temperature for 24 hours. The reaction was diluted with water (10 mL) and extracted with EA (15 mL × 3). The combined organic layers were dried with Na_2_SO_4_, concentrated under reduced pressure and the residue purified by column chromatography (10% EA/Hex) to yield 225.7 mg (39%) of peroxy triazole 23 as a yellow oil accompanied by 231.4 mg (50%) of aldehyde 25.

### Click reaction of peroxyacetals

To a solution of 4-((2-methylperoxypropan-2-yl)oxy)-1-butyne 8 (166 mg, 1.05 mmol) and 4-azidobutylbenzene 17 (193 mg, 1.10 mmol) in THF (6 mL) was added CuI (38.1 mg, 0.20 mmol, 0.2 equiv.), followed by triethylamine (0.28 mL, 2.01 mmol, 3 equiv.). The reaction was stirred at room temperature for 1.5 hours. The reaction was diluted with water (10 mL) and extracted with EA (15 mL × 3). The combined organic layers were dried with Na_2_SO_4_, concentrated under reduced pressure and the residue purified by column chromatography (10% EA/Hex) to yield a light yellow oil as a nearly inseparable mixture of 4-(2-((2-(methylperoxy)propan-2-yl)oxy)ethyl)-1-(4-phenylbutyl)-1*H*-1,2,3-triazole and 4-(2-acetoxyethyl)-1-(4-phenylbutyl)-1*H*-1,2,3-triazole. The peroxyacetal, typically formed in less than 10% yield, was contaminated with variable amounts of the ester.

#### 4-(2-((2-(Methylperoxy)propan-2-yl)oxy)ethyl)-1-(4-phenylbutyl)-1*H*-1,2,3-triazole

*R*_f_ = 0.34 (30% EA/Hex); ^1^H *δ* 7.32 (t, *J* = 7.1, 2H), 7.27 (s, 1H), 7.24 (t, *J* = 7.7, 1H), 7.23 (d, *J* = 7.5, 2H), 4.38 (t, *J* = 7.2, 2H), 3.86 (t, *J* = 7.6, 2H), 3.84 (s, 3H), 2.97 (t, *J* = 7.5, 2H), 2.71 (t, *J* = 7.3, 2H), 2.26 (p, *J* = 7.4, 2H), 1.28 (s, 6H); ^13^C *δ* 149.3, 140.4, 128.7, 128.6, 126.4, 104.9, 63.3, 60.7, 50.2, 32.6, 31.4, 29.8, 27.4, 23.2.

#### 4-(4-((2-(Methylperoxy)propan-2-yl)oxy)butyl)1-(3-phenylpropyl)-1*H*-1,2,3-triazole (26)

To a solution of 6-((2-methylperoxypropan-2-yl)oxy)-1-hexyne 10 (102 mg, 0.55 mmol) and 4-azidobutylbenzene 17 (98.1 mg, 0.56 mmol) in THF (5 mL) was added CuI (20.9 mg, 0.11 mmol, 0.2 equiv.), followed by triethylamine (0.23 mL, 1.7 mmol, 3 equiv.). The reaction was stirred at room temperature for 3 hours. The reaction was diluted with water (10 mL) and extracted with EA (15 mL × 3). The combined organic layers were dried with Na_2_SO_4_, concentrated under reduced pressure and the residue purified by column chromatography (10% EA/Hex) to yield 88.5 mg (45%) of the peroxyacetal/triazole 26 as a yellow oil: *R*_f_ = 0.28 (30% EA/Hex); ^1^H *δ* 7.32 (t, *J* = 7.3, 2H), 7.27 (s, 1H), 7.23 (t, *J* = 7.4, 1H), 7.19 (d, *J* = 7.2, 2H), 4.33 (t, *J* = 7.1, 2H), 3.85 (s, 3H), 3.56 (t, *J* = 6.5, 2H), 2.77 (t, *J* = 7.5, 2H), 2.67 (t, *J* = 7.5, 2H), 2.25 (p, *J* = 7.3, 2H), 1.82–1.56 (m, 4H), 1.40 (s, 6H); ^13^C *δ* 148.0, 140.4, 128.7, 128.6, 126.4, 120.7, 104.7, 63.3, 61.3, 49.5, 32.7, 31.8, 29.6, 26.2, 25.6, 23.2; IR: 3025, 2926, 2858, 2202, 1736, 1454, 1365, 1207, 1066, 748, 701; HRMS (ESI^+^, TOF) calcd for C_19_H_29_N_3_NaO_30_. [M + Na]^+^: 370.2107; found: 370.2103.

#### 5-Iodo-4-(4-((2-(methylperoxy)propan-2-yl)oxy)butyl)-1-(3-phenylpropyl)-1*H*-1,2,3-triazole (27i)

To a solution of 1-iodo-6-((2-methylperoxypropan-2-yl)oxy)-1-hexyne 11 (172 mg, 0.55 mmol) and 3-azidopropylbenzene 16 (96.7 mg, 0.60 mmol) in THF (5 mL) was added CuI (10.5 mg, 0.055 mmol, 0.1 equiv.), followed by triethylamine (0.15 mL, 1.08 mmol, 2 equiv.). The reaction was stirred at room temperature for 24 hours. The reaction was diluted with water (10 mL) and extracted with EA (15 mL × 3). The combined organic layers were dried with Na_2_SO_4_, concentrated under reduced pressure and the residue purified by column chromatography (10% EA/Hex) to yield 84.5 mg (33%) of peroxyacetal/iodotriazole 27i as a light yellow oil: *R*_f_ = 0.31 (30% EA/Hex); ^1^H *δ* 7.31 (t, *J* = 7.0, 2H), 7.23 (t, *J* = 7.0, 1H), 7.21 (d, *J* = 7.7), 4.36 (t, *J* = 7.2, 2H), 3.85 (s, 3H), 3.56 (t, *J* = 6.6, 2H), 2.70 (t, *J* = 7.6, 2H), 2.25 (p, *J* = 7.4, 2H), 1.84–1.62 (m, 4H), 1.40 (s, 6H); ^13^C *δ* 151.7, 140.4, 128.6, 128.5, 126.4, 104.7, 78.1, 63.3, 61.3, 50.1, 32.6, 31.3, 29.5, 26.0, 25.7, 23.2; IR: 3356, 3026, 2934, 2860, 1733, 1453, 1210, 1028, 745, 699; HRMS (ESI^+^, TOF) calcd for C_15_H_20_IN_3_NaO [(M − C_4_H_8_O_2_) + Na]^+^; elim. with loss of peroxyacetal]^+^: 408.0549; found: 408.0549.

### Click reaction with azidoalkyl peroxides

#### 4-Butyl-1-(4-(*tert*-butylperoxy)butyl)-1*H*-1,2,3-triazole (28)

To a solution of 1-azido-(4-(*tert*-butylperoxy)butane 12 (142 mg, 0.76 mmol) and 1-hexyne 14 (65.7 mg, 0.80 mmol) in THF (3.5 mL) was added CuI (30.5 mg, 0.16 mmol, 0.2 equiv.), followed by triethylamine (0.32 mL, 2.30 mmol, 3 equiv.). After stirring for 3.5 hours, the reaction was diluted with water (10 mL). The combined EA extracts (15 mL × 3) were dried with Na_2_SO_4_, and concentrated under reduced pressure. The residue was purified by column chromatography (10% EA/Hex) to yield 69.5 mg (34%) of peroxy triazole 28 as a light yellow oil: *R*_f_ = 0.25 (40% EA/Hex); ^1^H *δ* 7.26 (s, 1H), 4.34 (t, *J* = 7.2, 2H), 3.95 (t, *J* = 6.2, 2H), 2.70 (t, *J* = 7.7, 2H), 1.98 (p, *J* = 7.5, 2H), 1.64 (p, *J* = 7.4, 2H), 1.62 (p, *J* = 7.4, 2H), 1.37 (tt, *J* = 14.9, *J* = 7.4, 2H), 1.22 (s, 9H), 0.92 (t, *J* = 7.3, 3H); ^13^C *δ* 148.5, 120.6, 80.4, 74.2, 50.0, 31.7, 27.5, 26.4, 25.5, 25.1, 22.4, 14.0; IR: 3133, 2931, 2097, 1458, 1362, 1196, 1046, 880; HRMS (ESI^+^, TOF) calcd for C_14_H_27_N_3_NaO_2_ [M + Na]^+^: 292.2001; found: 292.2003.

#### 4-(4-(*tert*-Butylperoxy)butyl)-1-(4-phenylbutyl)-1*H*-1,2,3-triazole (29)

By a similar procedure as for synthesis of 28 (above), reaction of a THF solution (3.5 mL) of 1-azido-(4-(*tert*-butylperoxy)butane 12 (140 mg, 0.75 mmol) and 5-hexynyl benzene 15 (125 mg, 0.79 mmol), CuI (30.5 mg, 0.16 mmol, 0.2 equiv.), and triethylamine (0.32 mL, 2.30 mmol, 3 equiv.) furnished 38.5 mg (15%) of peroxy triazole 29 as a light yellow oil: *R*_f_ = 0.23 (40% EA/Hex); ^1^H *δ* 7.31–7.17 (m, 6H), 4.39 (t, *J* = 7.3, 2H), 3.99 (t, *J* = 6.2, 2H), 2.77–2.64 (m, 4H), 2.02 (m, 2H), 1.83–1.61 (m, 6H), 1.26 (s, 9H); ^13^C *δ* 152.1, 142.5, 128.6, 128.5, 128.4, 125.8, 80.3, 74.0, 50.6, 35.7, 31.0, 28.7, 27.0, 26.5, 26.1, 25.1; IR: 3026, 2925, 2155, 2032, 1454, 1362, 1196, 699; HRMS (ESI^+^, TOF) calcd for C_20_H_31_N_3_NaO_2_ [M + Na]^+^: 368.2314; found: 368.2313.

#### 4-Butyl-1-(4-(*tert*-butylperoxy)butyl)-1*H*-1,2,3-triazole (28, CuSO_4_ catalyst)

To a solution of azidoperoxide 12 (0.80 mmol) and 1-hexyne 14 (0.9 mmol) in CH_2_Cl_2_ (3.5 mL) was added a solution of CuSO_4_ (0.16 mmol, 0.2 equiv.) and sodium ascorbate (0.16 mmol, 0.2 equiv.) in water (1.5 mL). After stirring for 24 h, the reaction was diluted with water. The combined ethyl acetate extracts (3 × 15 mL) were dried with Na_2_SO_4_, concentrated under reduced pressure and purified by column chromatography (10% EA/Hex) to yield 61.7 mg (29%) of peroxy triazole 28 as a light yellow oil: *R*_f_ = 0.25 (40% EA/Hex).

#### 4-(4-(*tert*-Butylperoxy)butyl)-1-(4-phenylbutyl)-1*H*-1,2,3-triazole (29, CuSO_4_ catalyst)

By a similar procedure as described above for synthesis of 28, reaction of azidoperoxybutane 12 and hexynyl benzene (15) with CuSO_4_ and sodium ascorbate in CH_2_Cl_2_ water furnished 7.1 mg (5%) of peroxy triazole 29.

### Unsuccessful click reaction involving silylperoxy/azide 13

Reaction of peroxide 13 with alkynes 14 or 15 in the presence of 20% CuI/Et_3_N under conditions similar to those described earlier furnished a mixture of peroxide decomposition products. The same reaction, when conducted in the presence of 20% CuSO_4_/sodium ascorbate resulted in no detectable reaction (TLC) and extensive recovery of 13.

### Preparation of functionalized nanoparticles

Synthesis of pentanethiolate functionalized gold nanoparticles was adapted from a reported procedure.^38*a*^ An aqueous solution of hydrogen tetrachloroaurate (10 mL, 30 mmol, 30 mM) was vigorously stirred with a toluene solution of tetraoctylammonium bromide (27 mL, 50 mmol, 50 mM) until the tetrachloroaurate was transferred into the organic toluene layer. Pentanethiol (42 mg) was then added to the organic phase, followed by the addition of freshly prepared aqueous solution of sodium borohydride (8.33 mL, 3.3 mmol, 0.4 M). After the reaction had stirred for 3 hours at room temperature, the organic layer was separated and concentrated under reduced pressure to a volume of ∼1 mL. The concentrated layer was mixed with 125 mL of methanol and held at −18 C for 4 hours. The resulting solution was centrifuged. The nanoparticle-containing pellet was resuspended (methanol, 25 mL) and again centrifuged (3×) to yield (73.1 mg) a brown precipitate: ^1^H *δ* 1.32 (4H), 0.92 (3H);^39^ IR: 2956, 2921; elemental analysis: Au, 50.1; C, 43.9; S, 6.0%; XPS (binding energies): Au 4f_7/2_ (82.6 eV), Au 4f_5/2_ (86.4 eV), C 1s (284.3 eV), S 2p (161.2 eV). Solutions of the nanoparticles displayed a broad absorption in the following paragraph, a dodecanethiol-passivated set of nanoparticles prepared as a standard, and which did display the small 520 nm bump, otherwise exhibited identical properties to the C5 nanoparticles.^71^

#### Dodecanethiolate functionalized gold nanoparticles

Synthesis of dodecanethiolate functionalized gold nanoparticles were prepared following a reported procedure.^38*a*^ An aqueous solution of hydrogen tetrachloroaurate (10 mL, 30 mmol, 30 mM) was vigorously stirred with a toluene solution of tetraoctylammonium bromide (27 mL, 50 mmol, 50 mM) until the tetrachloroaurate was transferred into the organic toluene layer. Dodecanethiol (57 mg) was then added to the organic phase, followed by the addition of freshly prepared aqueous solution of sodium borohydride (8.33 mL, 3.3 mmol, 0.4 M). The reaction was stirred for 3 hours at room temperature. After 3 hours of stirring at room temperature, the organic layer was separate, and concentrated under reduced pressure to 1 mL, and mixed with 125 mL of methanol to remove excess thiol and kept at −18 C for 4 hours. The solution was centrifuged and washed with methanol (25 mL × 3) to yield (73.1 mg) a black precipitate: ^1^H *δ* 1.27 (10H), 0.91 (3H); IR: 2956, 2920; UV-vis: 520.65 nm (diagnostic signal; see ESI†); elemental analysis: Au, 78.4; C, 17.7; S, 3.9%; XPS (binding energies): Au 4f_7/2_ (82.9 eV), Au 4f_5/2_ (86.6 eV), C 1s (283.8 eV), S 2p (161.0 eV).

Azide functionalized gold nanoparticles (N_3_Au) were prepared *via* a modification of reported procedures.^36^ To a solution of C_5_SH-Au P (47.9 mg) in CH_2_Cl_2_ (5 mL) was added 6-azidohexane-1-thiol (142.5 mg). After stirring for 3 days at room temperature under nitrogen, the reaction solution was concentrated under reduced pressure and pelleted/resuspended (6 × 10 mL, methanol) to yield (62.3 mg) a brown precipitate: ^1^H *δ* 3.31 (2H), 1.45 (2H), 1.28 (4H), 0.87 (3H); IR: 2922, 2851, 2094; elemental analysis: Au, 54.0; C, 34.2; S, 8.5; N, 3.3%; XPS (binding energies): Au 4f_7/2_ (83.1 eV), Au 4f_5/2_ (86.9 eV), C 1s (283.9 eV), S 2p (162.0 eV), N 1s (398.3 eV).

Peroxide functionalized gold nanoparticles (PeroxideAu) were prepared *via* a modification of reported procedures.^37^ To a solution of 6-(*tert*-butylperoxy)-1-hexyne (182.9 mg, 1.07 mmol) and azidoalkylthiolate Au-NP (23.4 mg) in THF (5 mL) was added CuI (41.7 mg, 0.22 mmol, 0.2 equiv. per alkyne), followed by triethylamine (0.50 mL, 3.6 mmol, 3 equiv. per alkyne). The reaction was stirred at room temperature for 8 hours. The reaction was diluted with saturated aqueous solution of NH_4_Cl (10 mL) and the organic layer was washed with water (10 mL × 3). The organic layer was dried with Na_2_SO_4_, concentrated under reduced pressure and washed with methanol (10 mL × 6) to remove excess alkyne to yield (29.8 mg) a light brown precipitate: ^1^H *δ* 3.99 (2H), 2.47 (2H), 1.60–1.75 (8H), 1.26–1.30 (11H), 0.88 (1.5 H) IR: 2975, 2926, 2865, 1723, 1559, 1463, 1362, 1022, 803; elemental analysis: Au, 26.4; C, 49.9; S, 4.7; N, 5.1; O, 13.9%; XPS (binding energies): Au 4f_7/2_ (84.0 eV), Au 4f_5/2_ (87.8 eV), C 1s (284.3 eV), S 2p (162.9 eV), N 1s (399.6 eV), O 1s (531.5 eV).

#### Peroxyacetal functionalized gold nanoparticles (PeroxyacetalAu)

To a solution of 6-((2-peroxypropan-2-yl)oxy)-1-hexyne (197.9 mg, 1.06 mmol) and azidoalkylthiolate Au-NP (28.2 mg) in THF (5 mL) was added CuI (41.4 mg, 0.22 mmol, 0.2 equiv. per alkyne), followed by triethylamine (0.50 mL, 3.6 mmol, 3 equiv. per alkyne). The reaction was stirred at room temperature for 8 hours. The reaction was diluted with saturated aqueous solution of NH_4_Cl (10 mL) and the organic layer was washed with water (10 mL × 3). The organic layer was dried with Na_2_SO_4_, concentrated under reduced pressure and washed with methanol (10 mL × 6) to remove excess alkyne to yield (14.1 mg) a yellow precipitate: ^1^H *δ* 3.86 (2H), 2.05 (2H), 1.67 (2H), 1.57 (6H), 1.39 (5 H), 1.28 (4H), 1.12 (4H), 0.91 (5H); IR: 3411, 2921, 2851, 1738, 1240, 1065; elemental analysis: Au, 20.0; C, 52.6; S, 4.3; N, 4.6; O, 18.5%; XPS (binding energies): Au 4f_7/2_ (83.9 eV), Au 4f_5/2_ (87.6 eV), C 1s (284.0 eV), S 2p (163.2 eV), N 1s (399.3 eV), O 1s (531.5 eV).

## Abbreviations

Hexane(s): Hex
EA: Ethyl acetate
THF: Tetrahydrofuran
DMF: *N*,*N*-Dimethylformamide
DMSO: Dimethyl sulfoxide
EtOH: Ethanol
MeOH: Methanol
TEA: Triethylamine
Na ascorb.: Sodium ascorbate
TBHP: *tert*-Butyl hydroperoxide
TBTA: Tris[(1-benzyl-1*H*-1,2,3-triazol-4-yl)methyl]amine
Co(acac)_2_: Cobalt(ii) acetylacetonate

## Conflicts of interest

There are no conflicts of interest to declare.

## Supplementary Material

RA-010-D0RA09088C-s001
