# Supplementary material for: A click-based modular approach to introduction of peroxides onto molecules and nanostructures
Source: RSC Adv. 2020 Dec 16;10(72):44408–29. doi: 10.1039/d0ra09088c (PMC9058499; doi:10.1039/d0ra09088c)

Electronic Supplementary Material (ESI) for RSC Advances.
This journal is © The Royal Society of Chemistry 2020

SUPPORTING INFORMATION

A click-based approach to modular introduction of peroxides onto molecules and nanostructures. Horn, A.; Dussault, P. H.

Includes copies of ^{1}H and ^{13}C NMR spectra of molecules reported in the paper, as well as 1H, IR, and XPS characterization of functionalized Au nanoparticles (100 pages).

Corresponding author:

Pat Dussault
Department of Chemistry
University of Nebraska-Lincoln
Lincoln, NE 68588-0304
pdussault1@unl.edu

Contents

6-(*tert*-Butylperoxy)-1-hexyne (**1**)
^{1}H S6
^{13}C S7

8-(*tert*-Butylperoxy)-1-octyne (**2**)
^{1}H S8
^{13}C S9

1-Iodo-6-tert-butylperoxy hexyne (**3**)
^{1}H S10
^{13}C S11

1-Iodo-8-tert-butylperoxy octyne (**4**)
^{1}H S12
^{13}C S13

3-((2-hydroperoxypropan-2-yl)oxy)-1-propyne (**5**)
^{1}H S14
^{13}C S15

3-((2-((2-Hydroperoxypropan-2-yl)peroxy)propan-2-yl)oxy)-1-propyne (**5a**)
^{1}H S16
^{13}C S17

4-((2-Hydroperoxypropan-2-yl)oxy)-1-butyne (**6**)
^{1}H S18
^{13}C S19

4-((2-((2-Hydroperoxypropan-2-yl)peroxy)propan-2-yl)oxy)-1-butyne (**6a**)
^{1}H S20
^{13}C S21

6-((2-Hydroperoxypropan-2-yl)oxy)-1-hexyne (**7**)
^{1}H S22
^{13}C S23

4-((2-Methylperoxypropan-2-yl)oxy)-1-butyne (**8**)
^{1}H S24
^{13}C S25
6-((2-Methylperoxypropan-2-yl)oxy)-1-hexyne (**9**)
^{1}H S26
^{13}C S27

1-Iodo-4-((2-methylperoxypropan-2-yl)oxy)-1-butyne (**10**)
^{1}H S28
^{13}C S29

1-Iodo-6-((2-methylperoxypropan-2-yl)oxy)-1-hexyne (**11**)
^{1}H S30
^{13}C S31

1-Azido-(4-(*tert*-butylperoxy) butane (**12**)
^{1}H S32
^{13}C S33

(6-Azidohexan-2-yl)peroxy)triethylsilane (**13**)
^{1}H S34
^{13}C S35

Hex-5-yn-1-yl benzene (**15**)
^{1}H S36
^{13}C S37

3-Azidopropylbenzene (**16**)
^{1}H S38
^{13}C S39

4-Azidobutylbenzene (**17**)
^{1}H S40
^{13}C S41

6-Azidohexane-1-thiol (**18**)
^{1}H S42
^{13}C S43

4-(4-(*tert*-Butylperoxy)butyl)-1-(3-phenylpropyl)-1*H*-1,2,3-triazole (**19**)
^{1}H S44
^{13}C S45

4-(4-(*tert*-Butylperoxy)butyl)-5-iodo-1-(3-phenylpropyl)-1*H*-1,2,3-triazole (**19i**)
^{1}H S46
^{13}C S47

4-(4-(*tert*-Butylperoxy)butyl)-1-(4-phenylbutyl)-1*H*-1,2,3-triazole (**20**)
^{1}H S48
^{13}C S49

4-(4-(*tert*-Butylperoxy)butyl)-5-iodo-1-(4-phenylbutyl)-1*H*-1,2,3-triazole (**20i**)
^{1}H S50
^{13}C S51

4-(1-(4-Phenylbutyl)-)-1H-1,2,3-triazol-4-yl)butanal (**21**)
^{1}H S52
^{13}C S53

4-(6-(*tert*-Butylperoxy)hexyl)-1-(3-phenylpropyl)-1*H*-1,2,3-triazole (**22**)
^{1}H S54
^{13}C S55

4-(6-(*tert*-Butylperoxy)hexyl)-5-iodo-1-(3-phenylpropyl)-1*H*-1,2,3-triazole (**22i**)
^{1}H S56
^{13}C S57

4-(6-(*tert*-Butylperoxy)hexyl)-1-(4-phenylbutyl)-1*H*-1,2,3-triazole (**23**)
^{1}H S58
^{13}C S59

4-(6-(*tert*-butylperoxy)hexyl)-5-iodo-1-(4-phenylbutyl)-1*H*-1,2,3-triazole (**23i**)
^{1}H S60
^{13}C S61

6-(1-(3-Phenylpropyl)-1*H*-1,2,3-triazol-4-yl)hexanal (**24**)
^{1}H S62
^{13}C S63

6-(1-(4-phenylbutyl)-1*H*-1,2,3-triazol-4-yl)hexanal (**25**)
^{1}H S64
^{13}C S65

4-(4-((2-(Methylperoxy)propan-2-yl)oxy)butyl)1-(3-phenylpropyl)-1*H*-1,2,3-triazole (**26**)s
^{1}H S66
^{13}C S67

5-Iodo-4-(4-((2-(methylperoxy)propan-2-yl)oxy)butyl)-1-(3-phenylpropyl)-1*H*-1,2,3-triazole (**27i**)
^{1}H S68
^{13}C S69

4-Butyl-1-(4-(*tert*-butylperoxy)butyl)-1*H*-1,2,3-triazole (**28**)
^{1}H S70
^{13}C S71

4-(4-(*tert*-Butylperoxy)butyl)-1-(4-phenylbutyl)-1*H*-1,2,3-triazole (**29**)
^{1}H S72
^{13}C S73

Pentanethiolate Functionalized Gold Nanoparticles
^{1}H (**SI-Figure 1**) S76
XPS Au (**SI-Figure 2**) S77
XPS S (**SI-Figure 3**) S78
XPS C (**SI-Figure 4**) S79

Dodecanethiolate Functionalized Gold Nanoparticles
^{1}H (**SI-Figure 5**) S80

XPS Au (**SI-Figure 6**) S81
XPS S (**SI-Figure 7**) S82
XPS C (**SI-Figure 8**) S83

Azide Functionalized Gold Nanoparticles
^{1}H (**SI-Figure 9**) S84
XPS Au (**SI-Figure 10**) S85
XPS S (**SI-Figure 11**) S86
XPS C (**SI-Figure 12**) S87
XPS N (**SI-Figure 13)** S88

Peroxide Functionalized Gold Nanoparticles
^{1}H (**SI-Figure 14**) S89
XPS Au (**SI-Figure 15**) S90
XPS S (**SI-Figure 16**) S91
XPS C (**SI-Figure 17**) S92
XPS N (**SI-Figure 18**) S93
XPS O (**SI-Figure 19**) S94

Peroxyacetal Functionalized Gold Nanoparticles
^{1}H (**SI-**Figure 20) S95
XPS Au (**SI-**Figure 21) S96
XPS S (**SI-**Figure 22) S97
XPS C (**SI-**Figure 23) S98
XPS N (**SI-**Figure 24) S99
XPS O (**SI-**Figure 25) S100

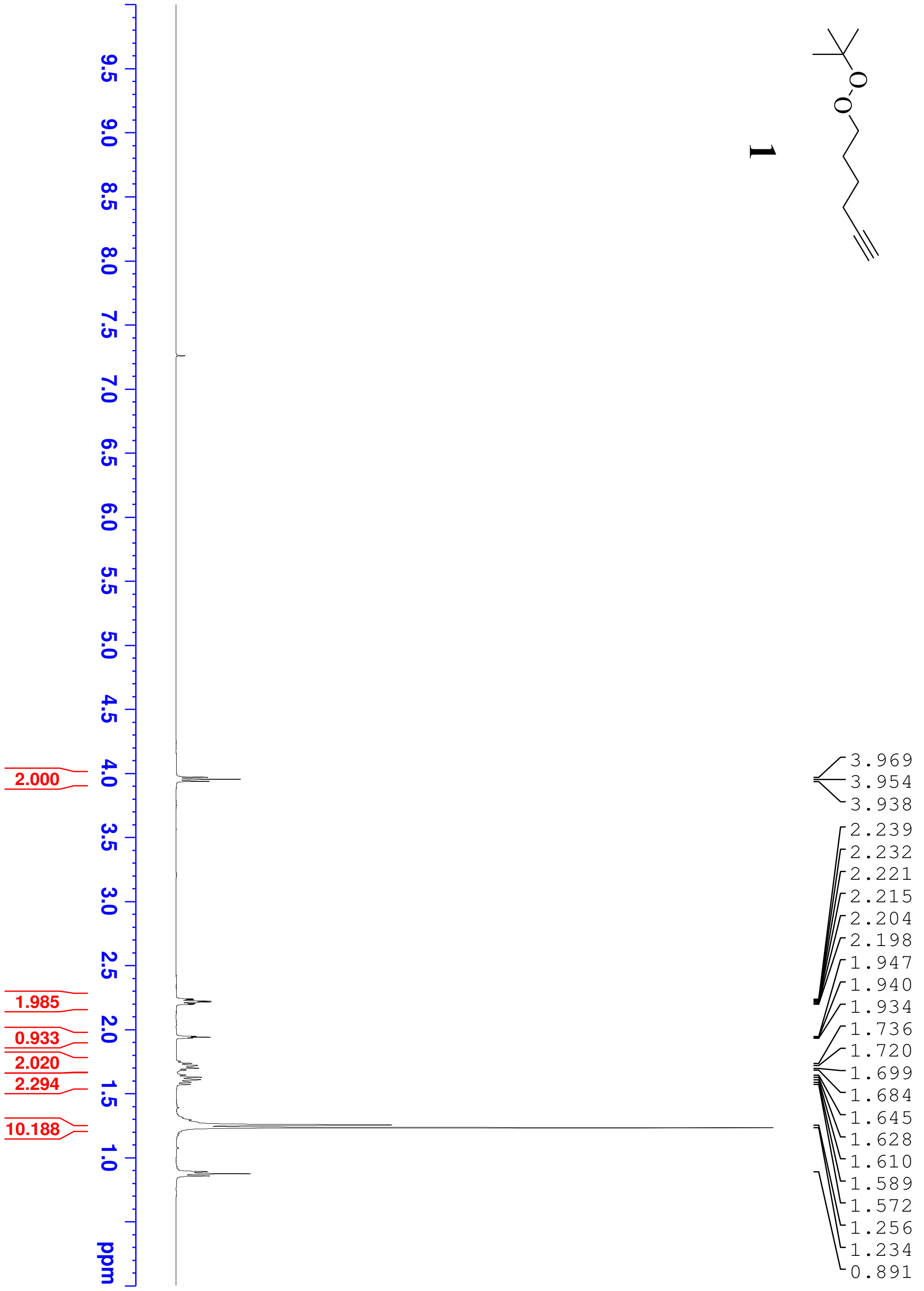
1
9.5
9.0
8.5
8.0
7.5
7.0
6.5
6.0
5.5
5.0
4.5
4.0
3.5
3.0
2.5
2.0
1.5
1.0
ppm
2.000
1.985
0.933
2.020
2.294
10.188
3.969
3.954
3.938
2.239
2.232
2.221
2.215
2.204
2.198
1.947
1.940
1.934
1.736
1.720
1.699
1.684
1.645
1.628
1.610
1.589
1.572
1.256
1.234
0.891

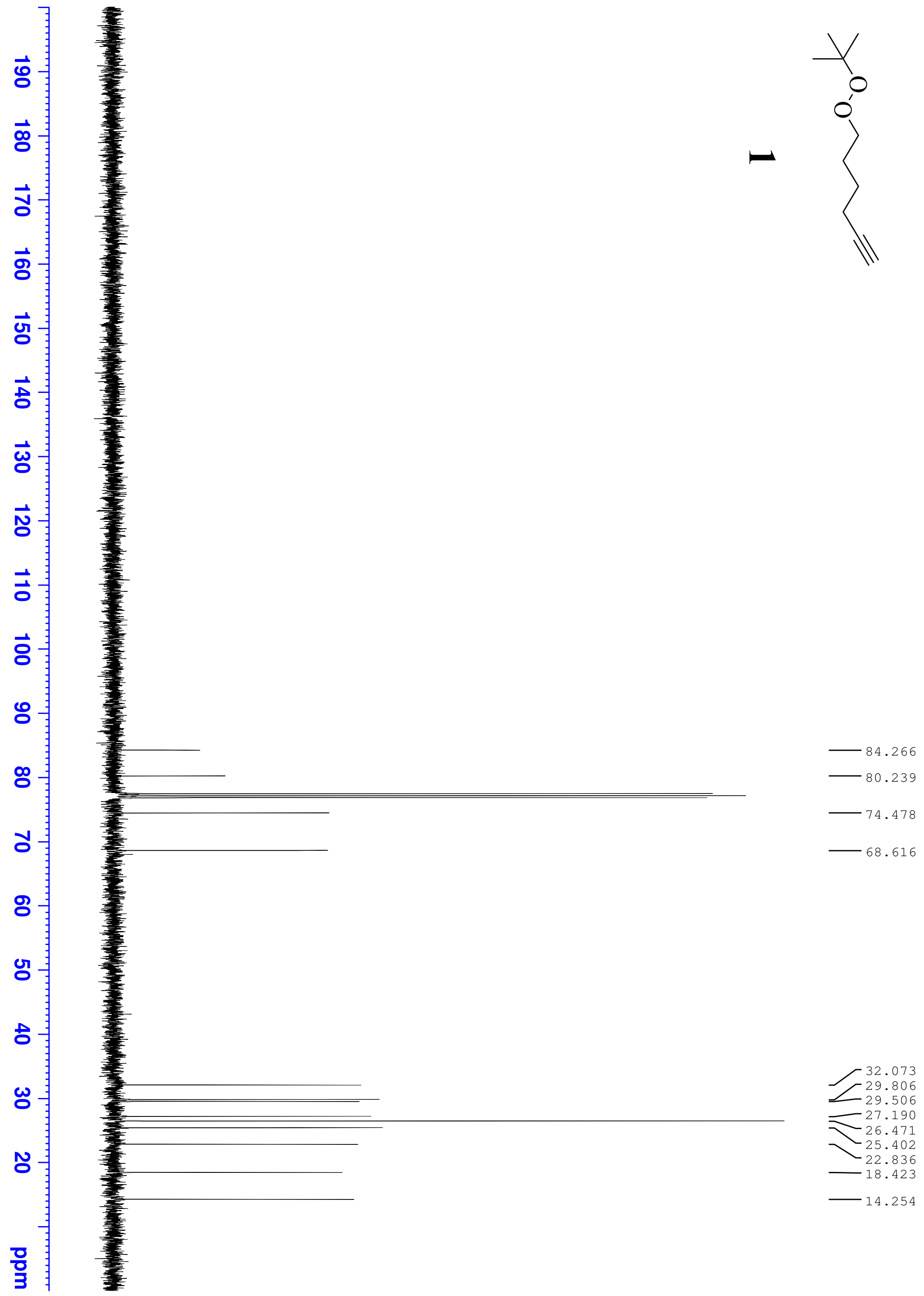
1
O
O
84.266
80.239
74.478
68.616
32.073
29.806
29.506
27.190
26.471
25.402
22.836
18.423
14.254
190
180
170
160
150
140
130
120
110
100
90
80
70
60
50
40
30
20
ppm

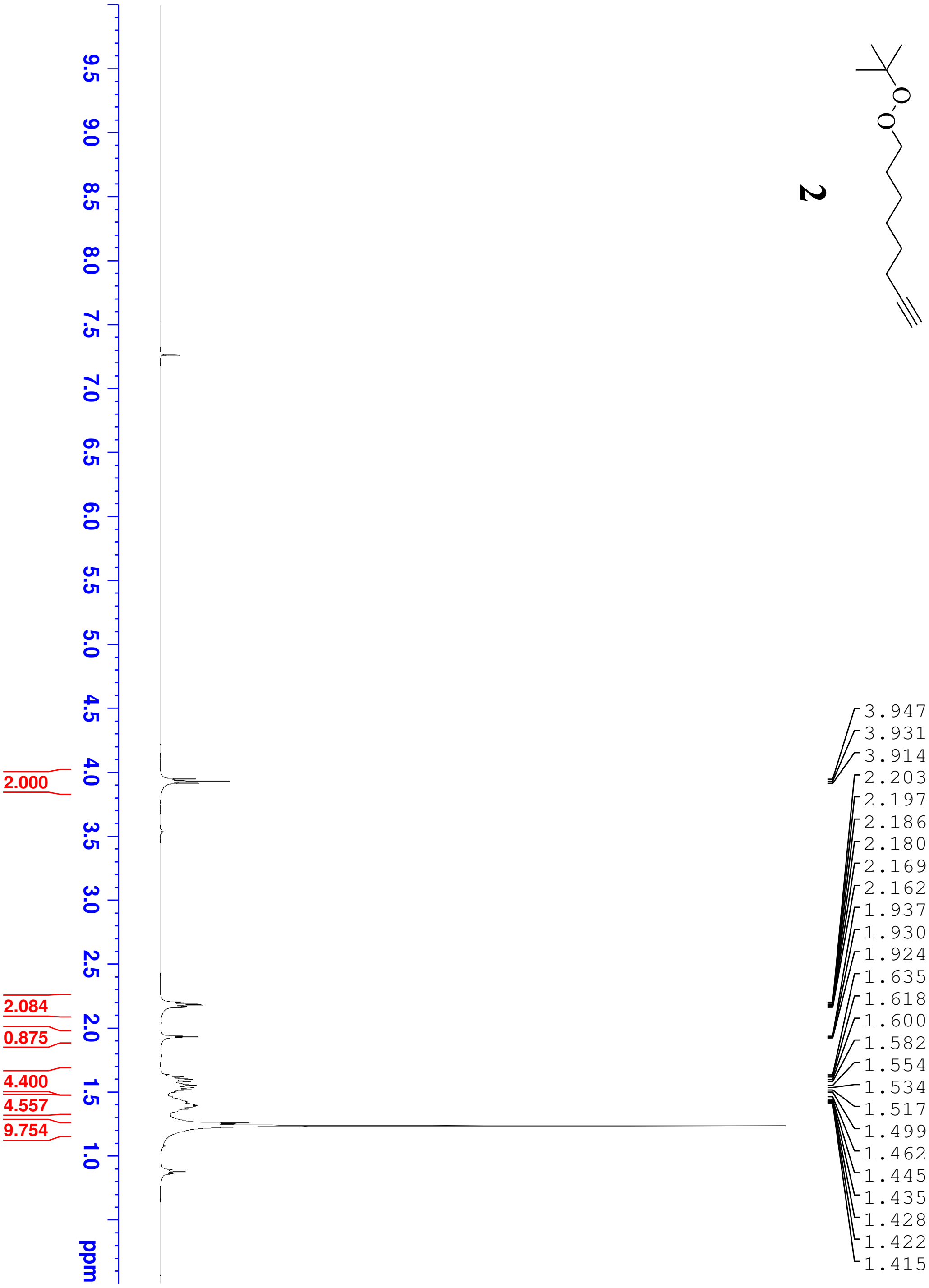
2
3.947
3.931
3.914
2.203
2.197
2.186
2.180
2.169
2.162
1.937
1.930
1.924
1.635
1.618
1.600
1.582
1.554
1.534
1.517
1.499
1.462
1.445
1.435
1.428
1.422
1.415
2.000
2.084
0.875
4.400
4.557
9.754
9.5
9.0
8.5
8.0
7.5
7.0
6.5
6.0
5.5
5.0
4.5
4.0
3.5
3.0
2.5
2.0
1.5
1.0
ppm

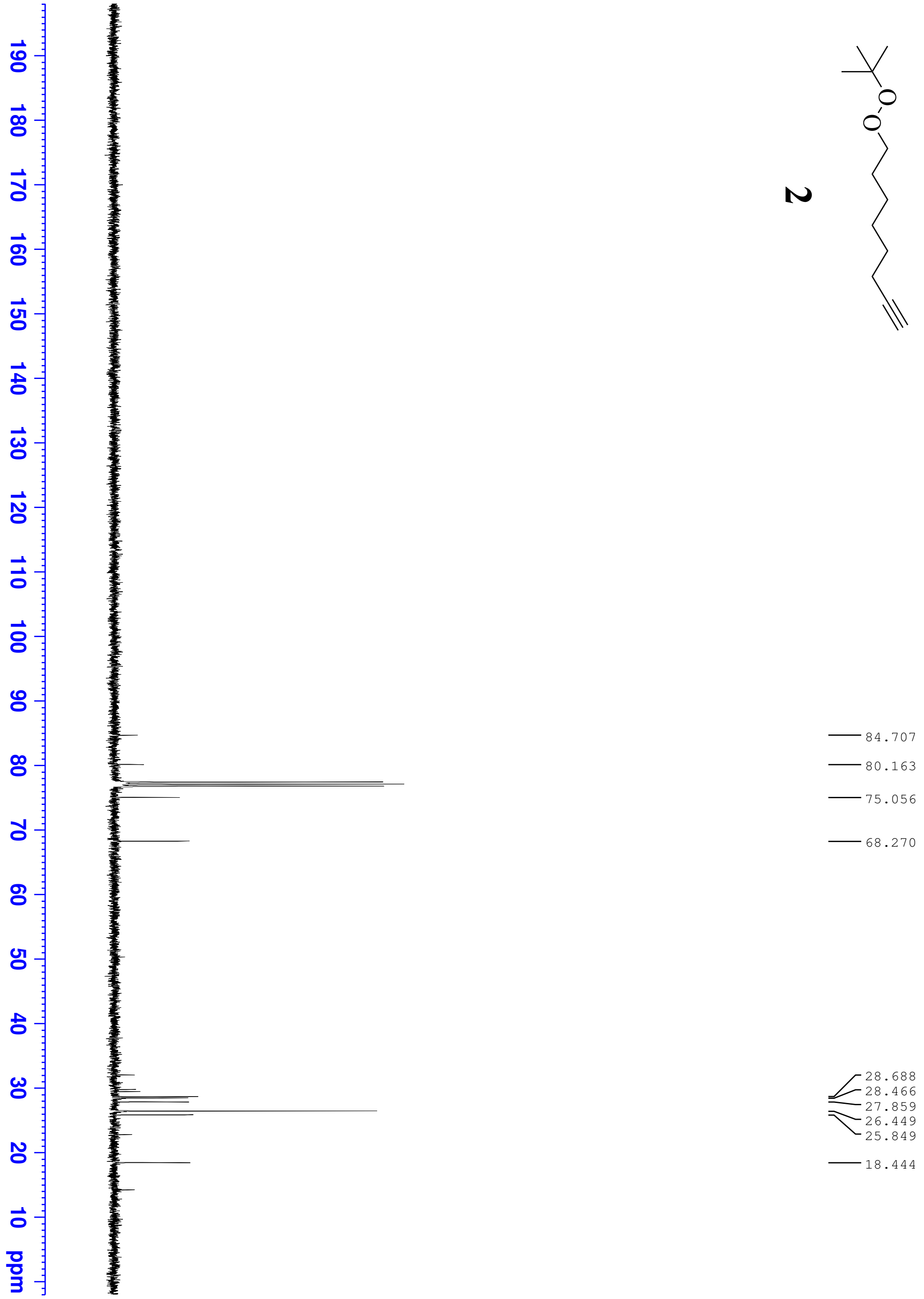
2
84.707
80.163
75.056
68.270
28.688
28.466
27.859
26.449
25.849
18.444
190
180
170
160
150
140
130
120
110
100
90
80
70
60
50
40
30
20
10
ppm

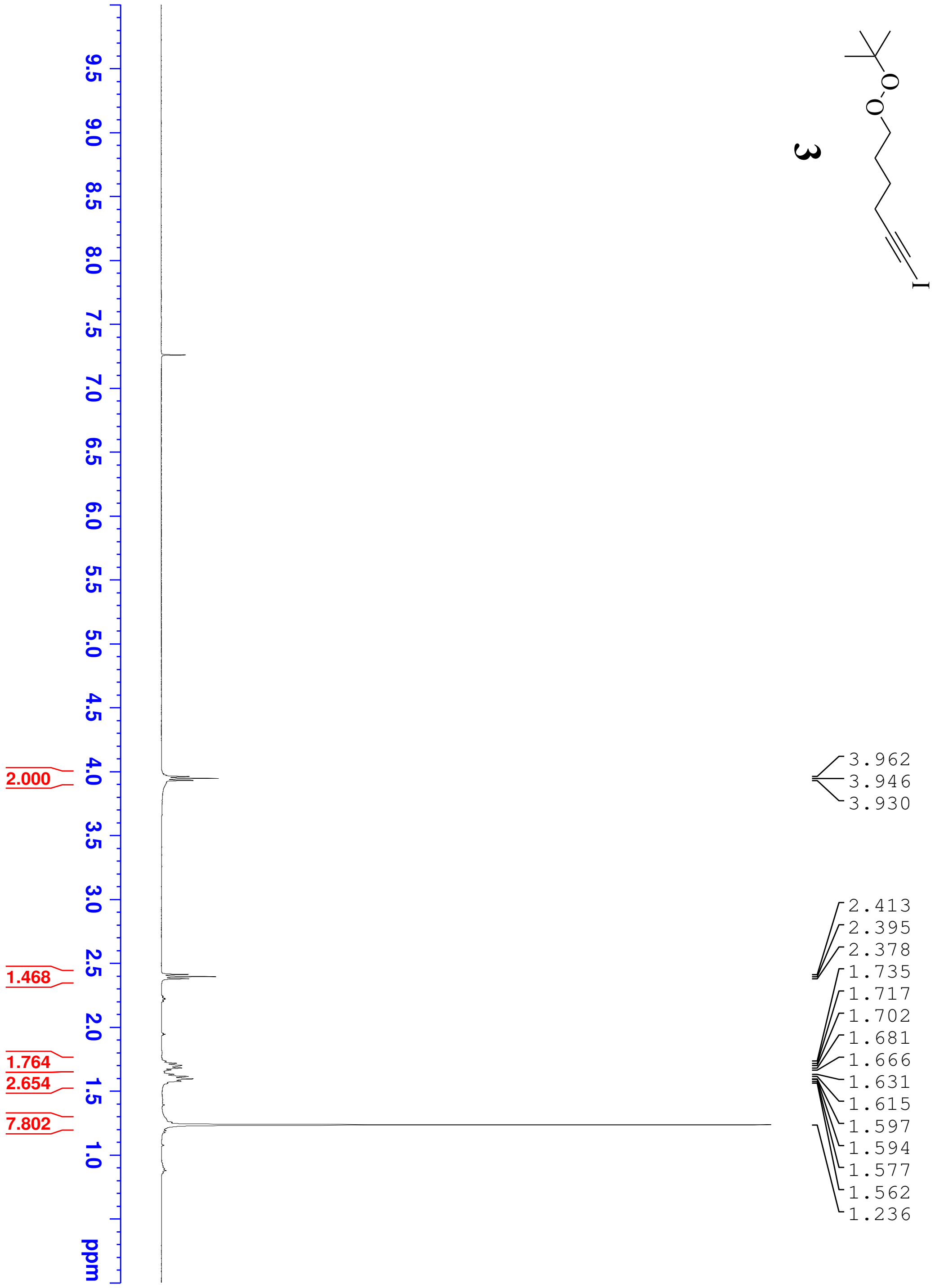
3
I
O
O
3.962
3.946
3.930
2.413
2.395
2.378
1.735
1.717
1.702
1.681
1.666
1.631
1.615
1.597
1.594
1.577
1.562
1.236
2.000
1.468
1.764
2.654
7.802
9.5
9.0
8.5
8.0
7.5
7.0
6.5
6.0
5.5
5.0
4.5
4.0
3.5
3.0
2.5
2.0
1.5
1.0
ppm

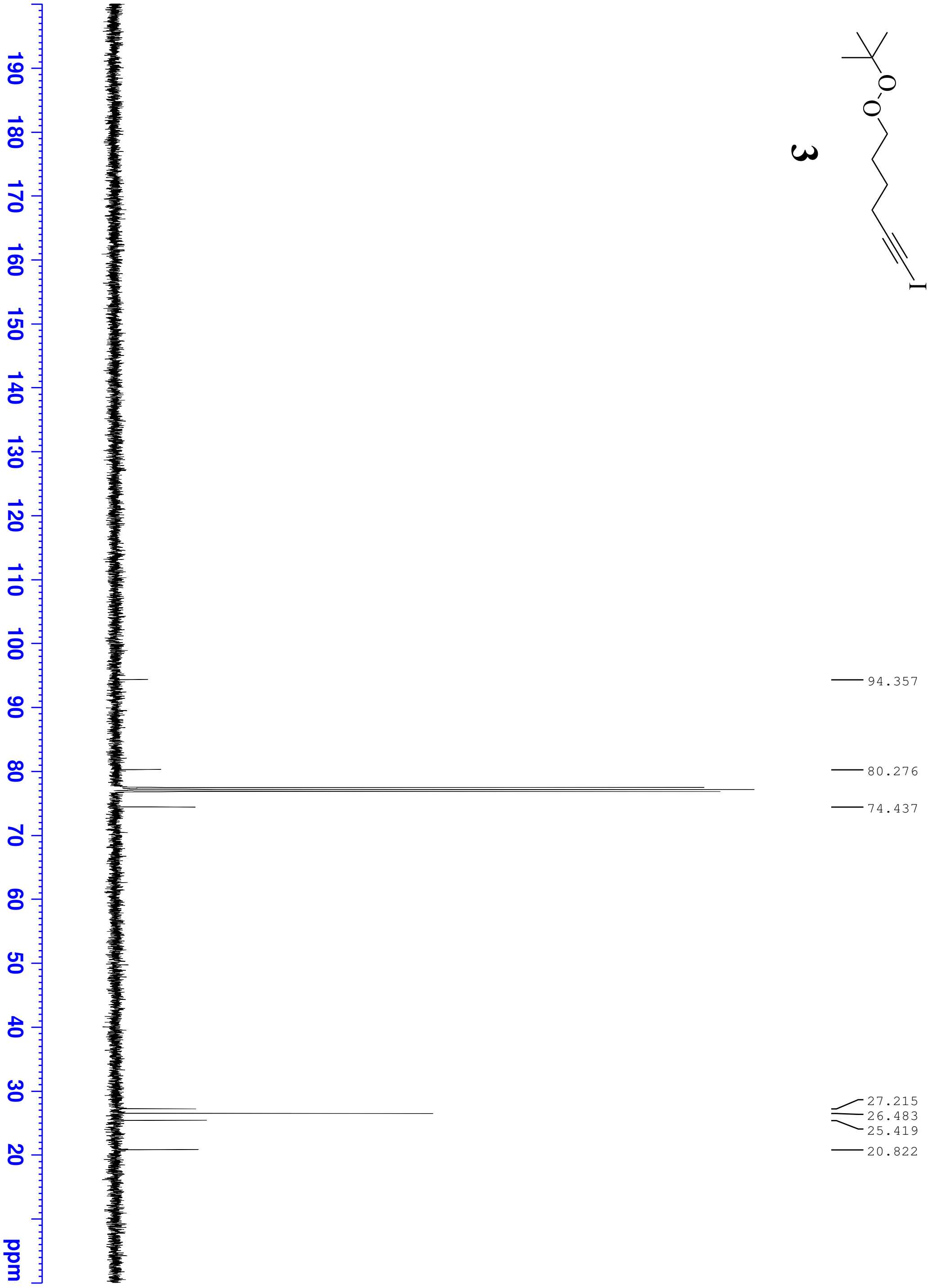
3
94.357
80.276
74.437
27.215
26.483
25.419
20.822
ppm

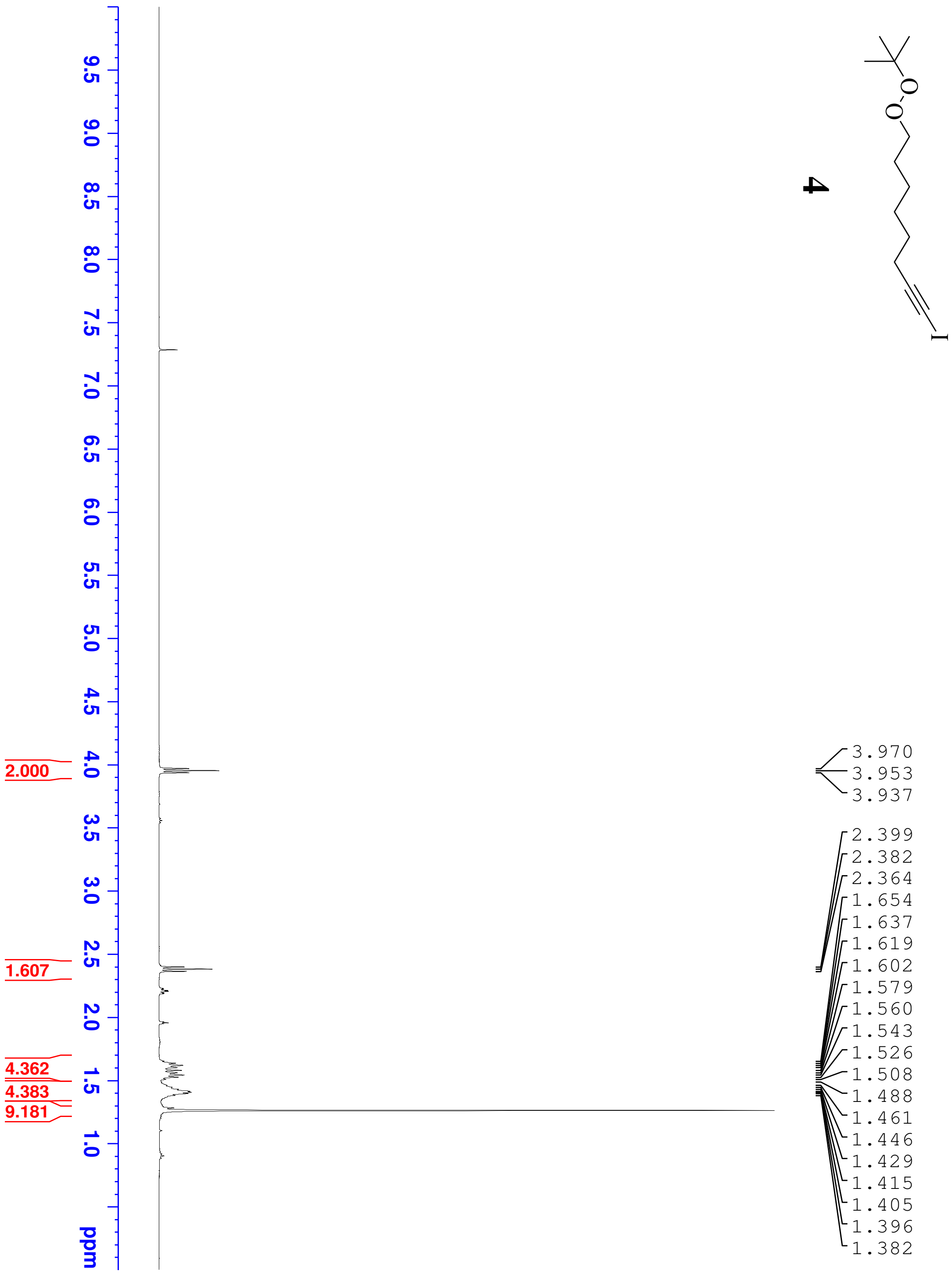

4
I
O
O
3.970
3.953
3.937
2.399
2.382
2.364
1.654
1.637
1.619
1.602
1.579
1.560
1.543
1.526
1.508
1.488
1.461
1.446
1.429
1.415
1.405
1.396
1.382
2.000
1.607
4.362
4.383
9.181
9.5
9.0
8.5
8.0
7.5
7.0
6.5
6.0
5.5
5.0
4.5
4.0
3.5
3.0
2.5
2.0
1.5
1.0
ppm

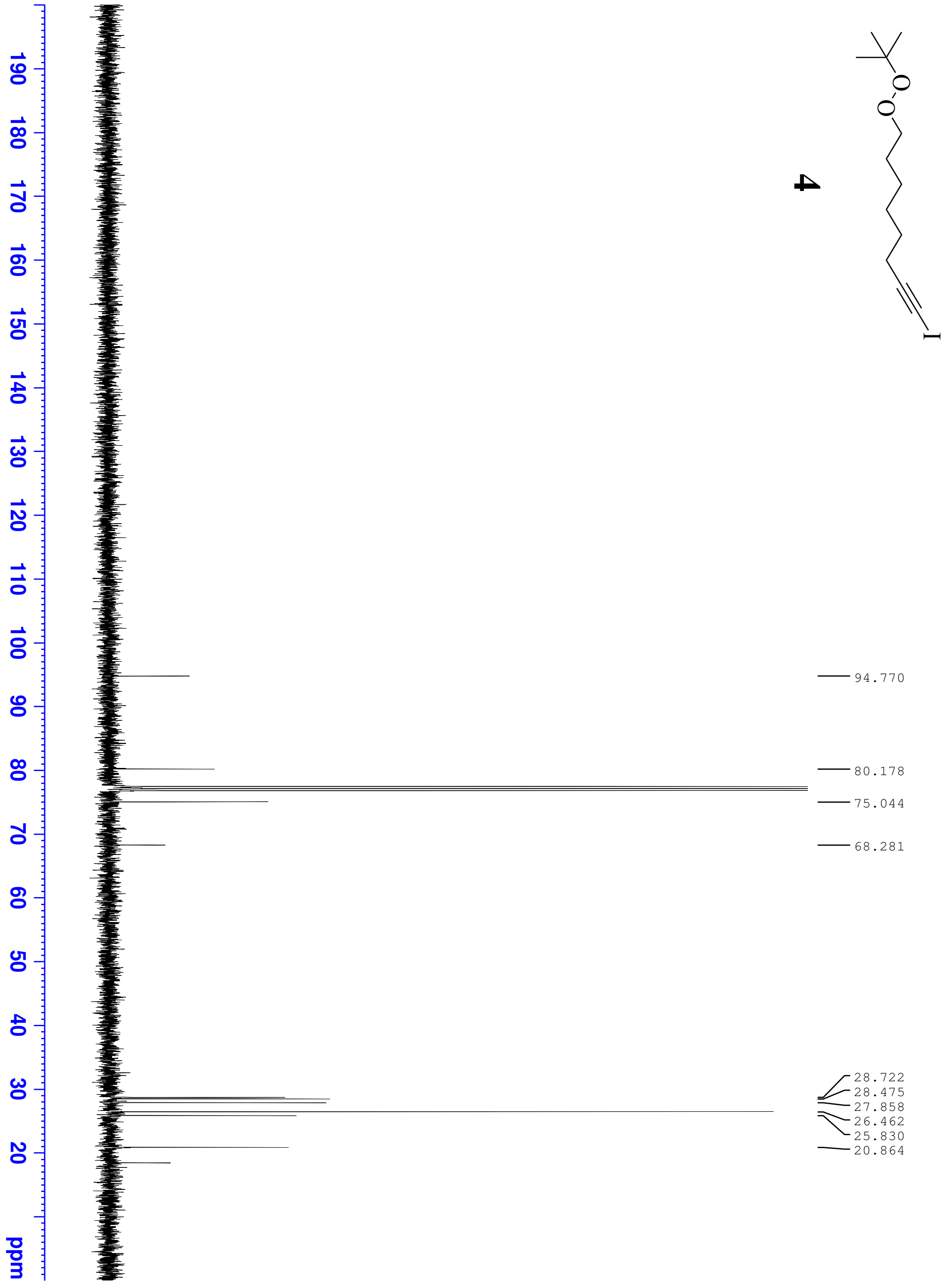
94.770
80.178
75.044
68.281
28.722
28.475
27.858
26.462
25.830
20.864

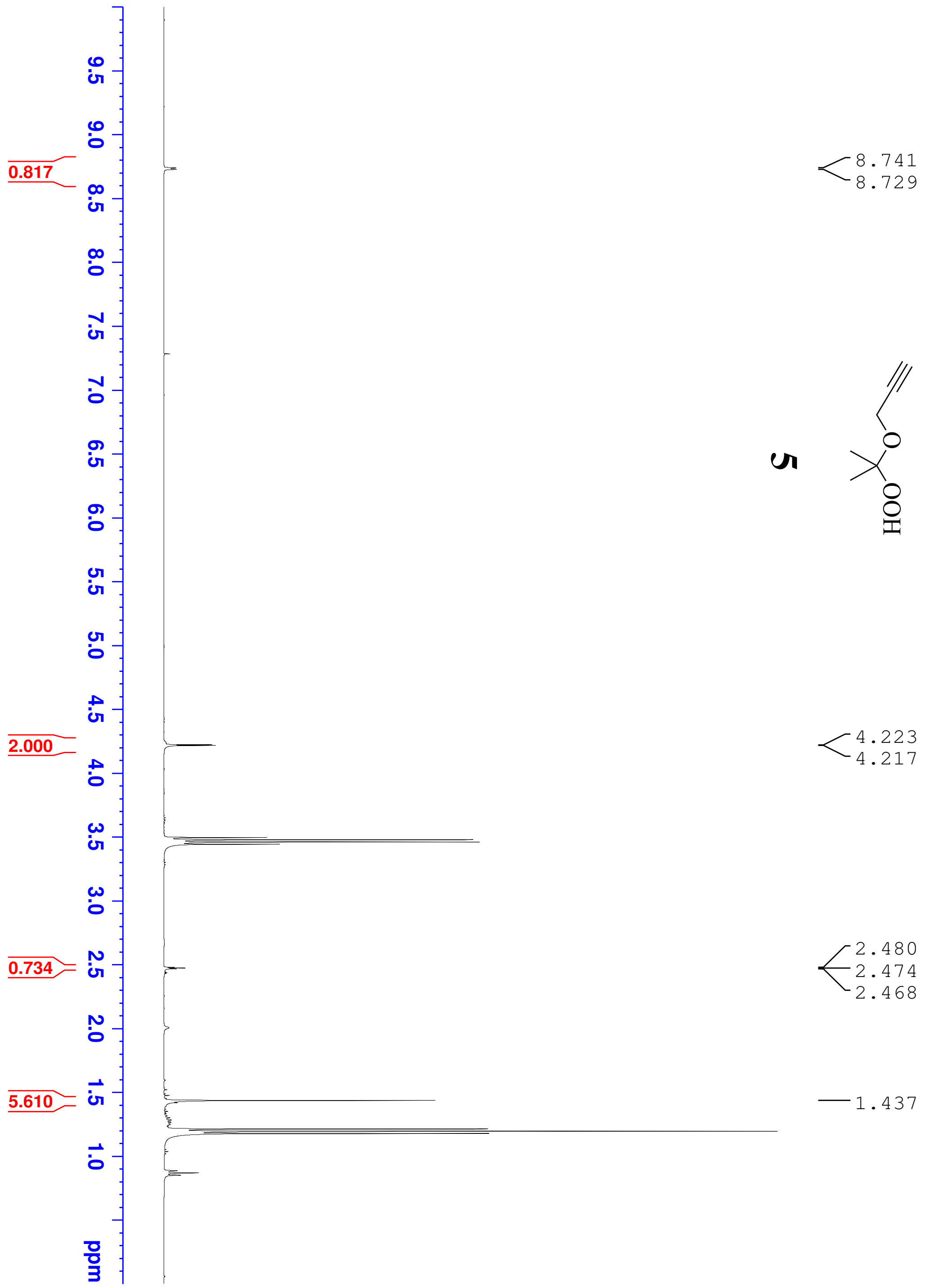
8.741
8.729
4.223
4.217
2.480
2.474
2.468
1.437
0.817
2.000
0.734
5.610
9.5
9.0
8.5
8.0
7.5
7.0
6.5
6.0
5.5
5.0
4.5
4.0
3.5
3.0
2.5
2.0
1.5
1.0
ppm
5
O
OOH

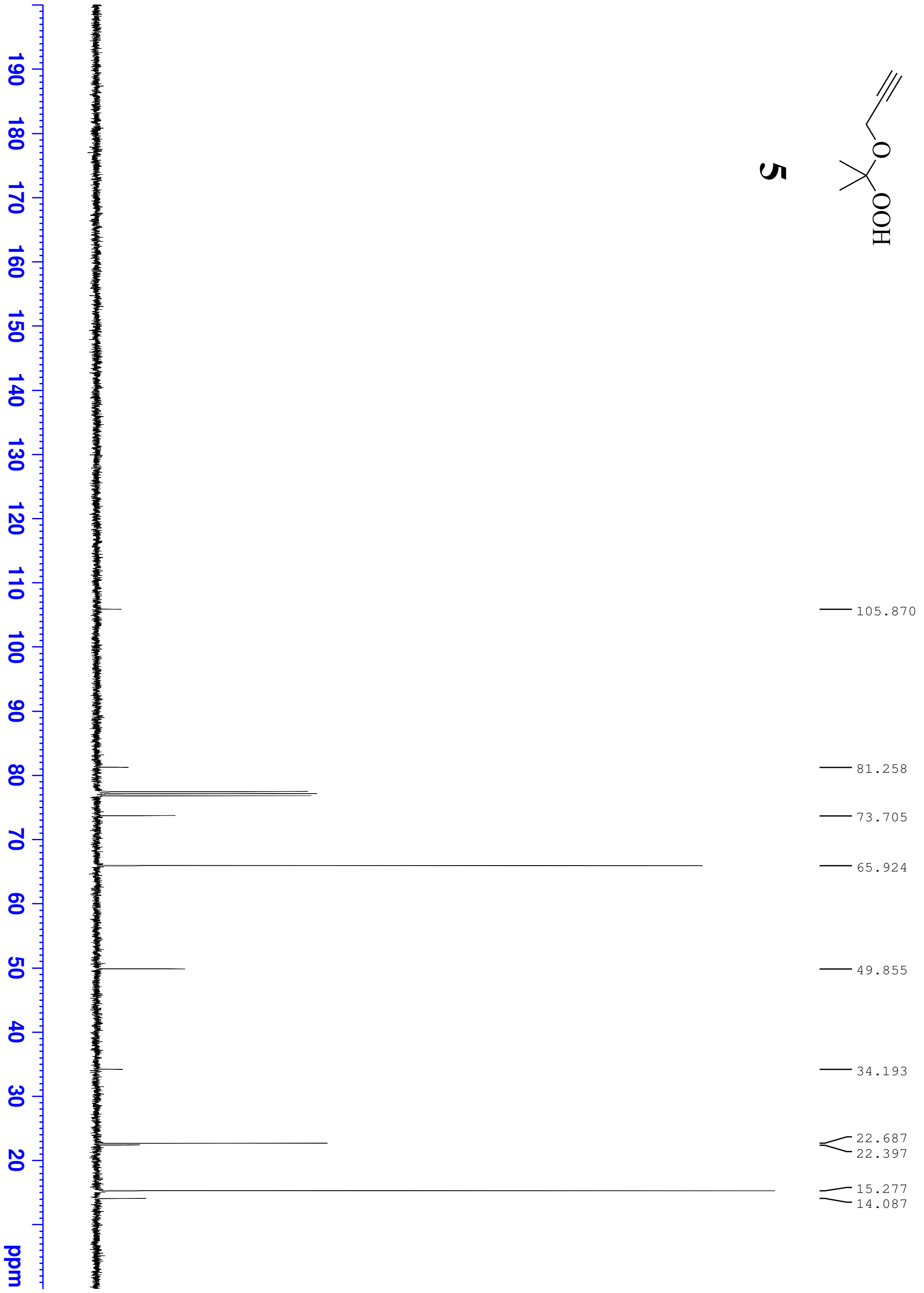

5
OOH
O
105.870
81.258
73.705
65.924
49.855
34.193
22.687
22.397
15.277
14.087
ppm

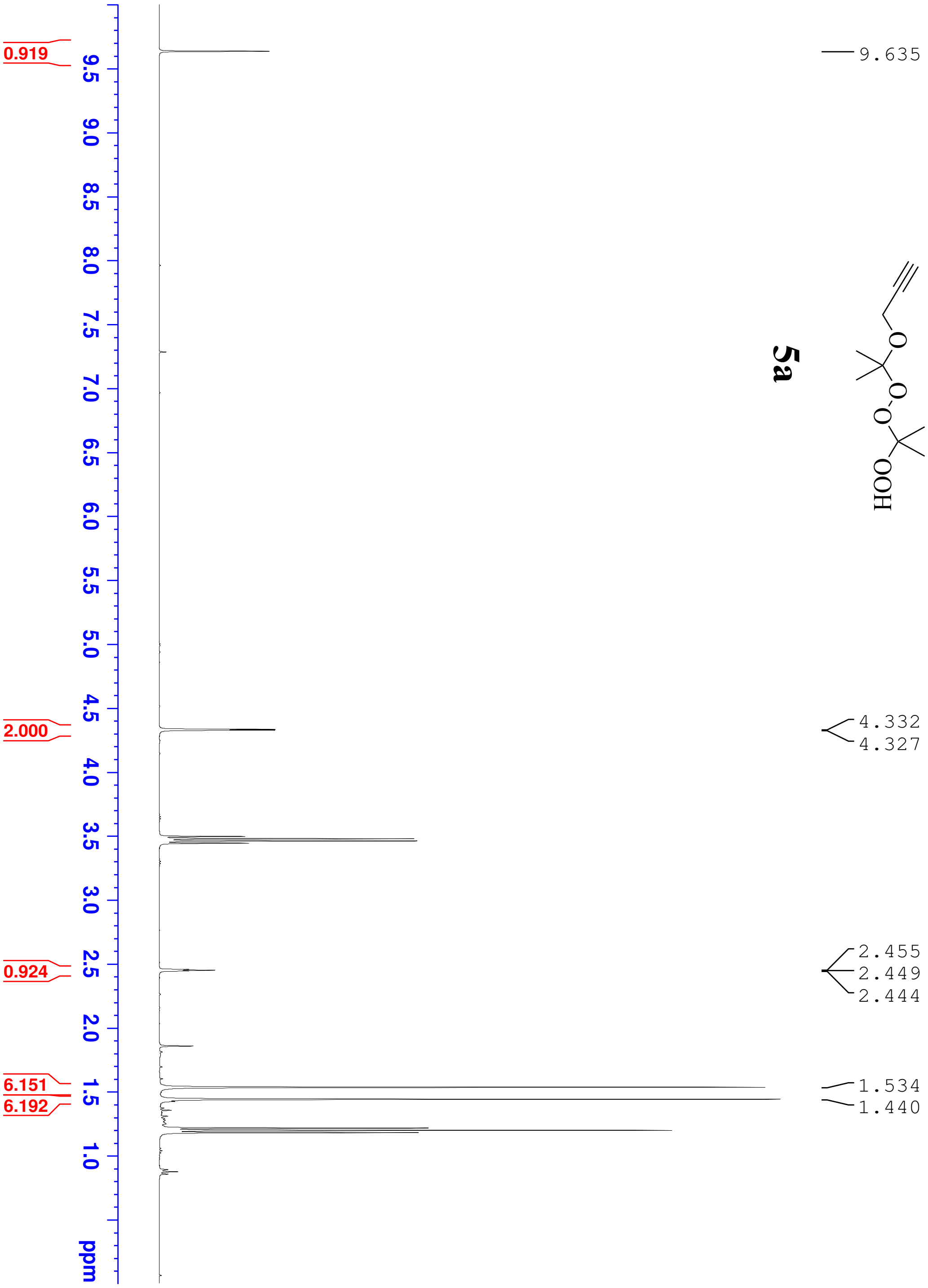
0.919
2.000
0.924
6.151
6.192
9.635
4.332
4.327
2.455
2.449
2.444
1.534
1.440
5a
OOH
ppm

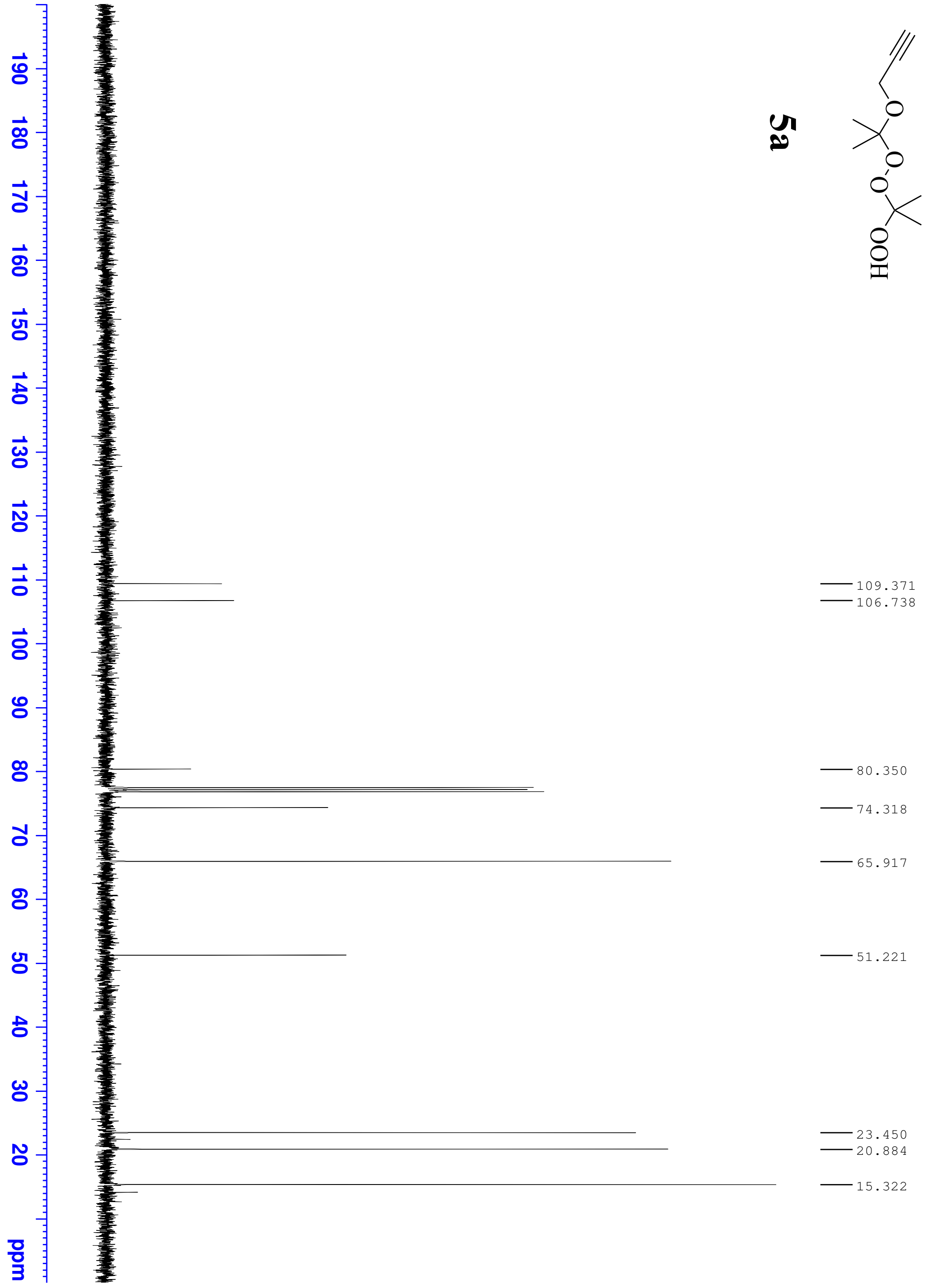
5a
OOH
O
O
O
109.371
106.738
80.350
74.318
65.917
51.221
23.450
20.884
15.322
ppm

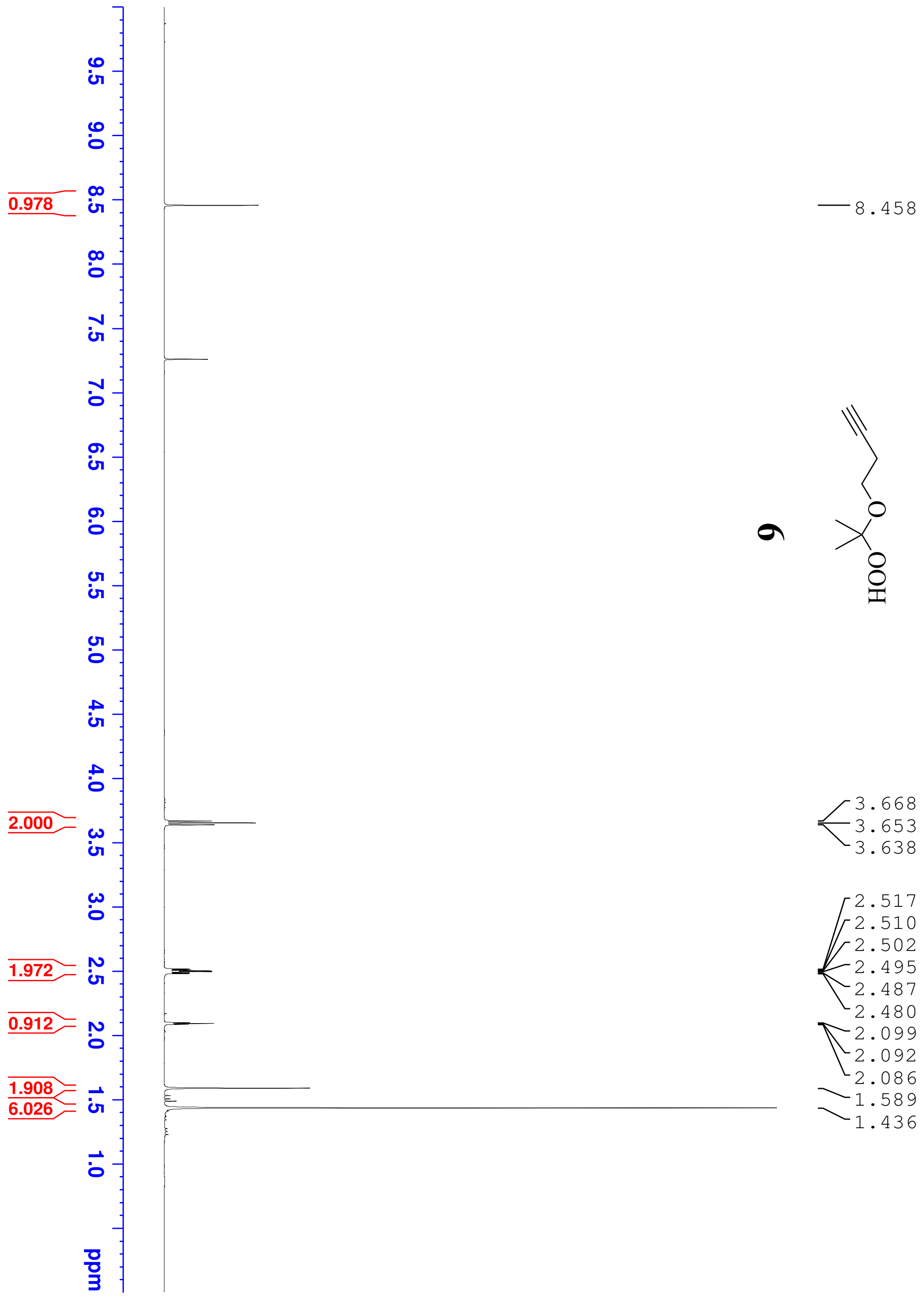
9.5
9.0
8.5
8.0
7.5
7.0
6.5
6.0
5.5
5.0
4.5
4.0
3.5
3.0
2.5
2.0
1.5
1.0
ppm
0.978
2.000
1.972
0.912
1.908
6.026
8.458
3.668
3.653
3.638
2.517
2.510
2.502
2.495
2.487
2.480
2.099
2.092
2.086
1.589
1.436
6
HOO
O

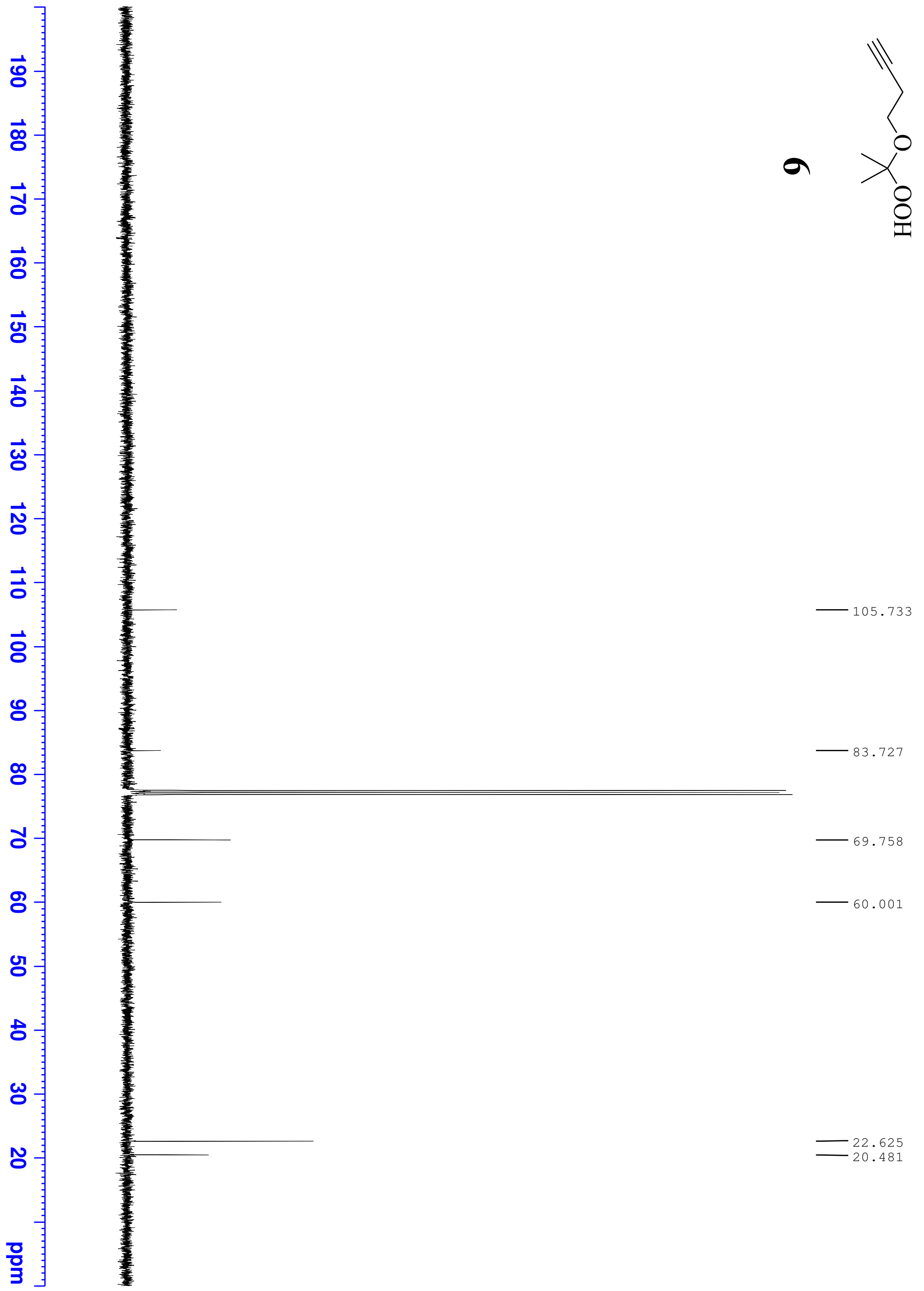
6
HOO
O
105.733
83.727
69.758
60.001
22.625
20.481
ppm

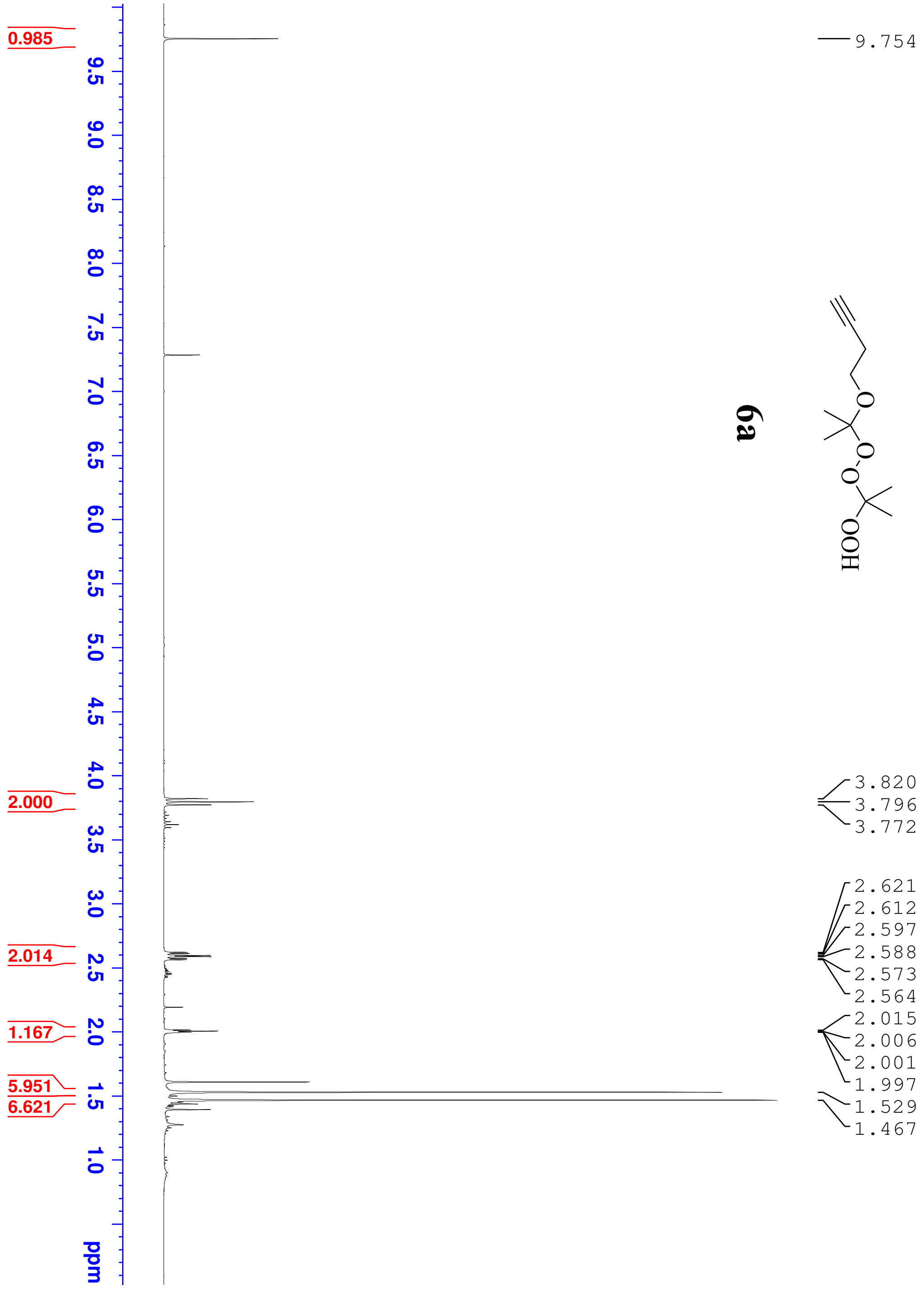
6a
HOO
O
O
O
9.754
3.820
3.796
3.772
2.621
2.612
2.597
2.588
2.573
2.564
2.015
2.006
2.001
1.997
1.529
1.467
0.985
2.000
2.014
1.167
5.951
6.621
9.5
9.0
8.5
8.0
7.5
7.0
6.5
6.0
5.5
5.0
4.5
4.0
3.5
3.0
2.5
2.0
1.5
1.0
ppm

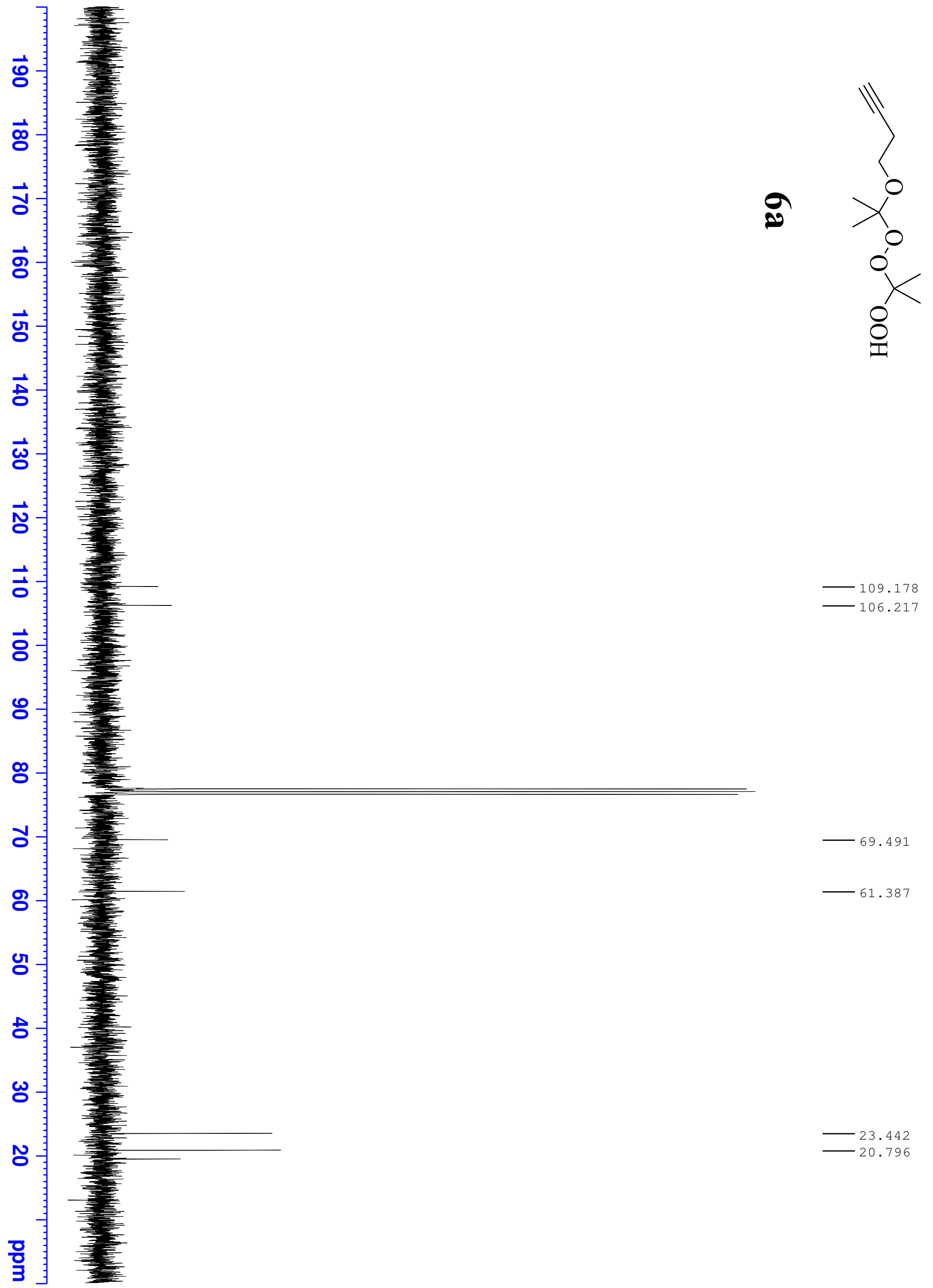
6a
109.178
106.217
69.491
61.387
23.442
20.796
ppm

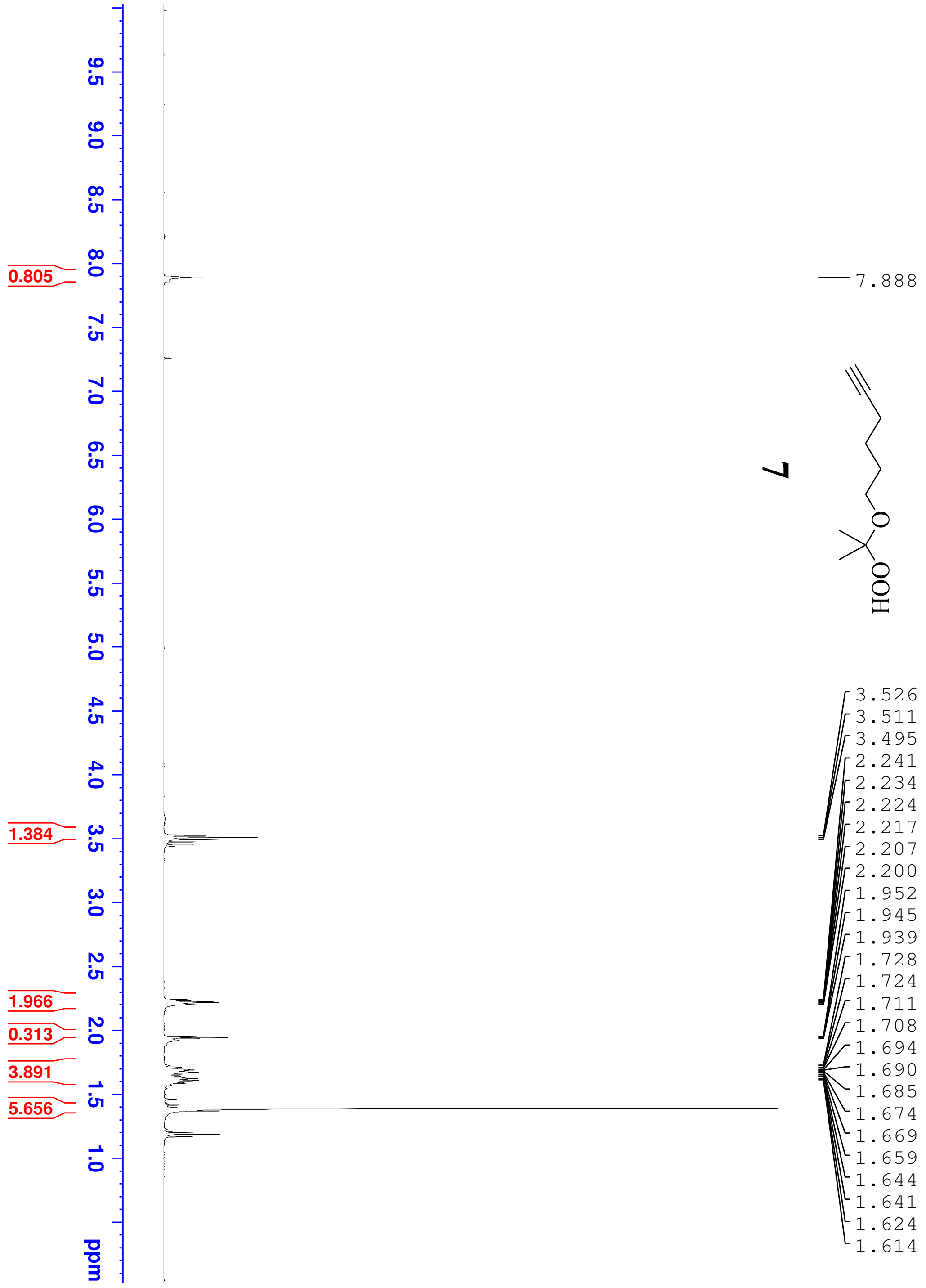
7
HOO
O
7.888
3.526
3.511
3.495
2.241
2.234
2.224
2.217
2.207
2.200
1.952
1.945
1.939
1.728
1.724
1.711
1.708
1.694
1.690
1.685
1.674
1.669
1.659
1.644
1.641
1.624
1.614
0.805
1.384
1.966
0.313
3.891
5.656
ppm

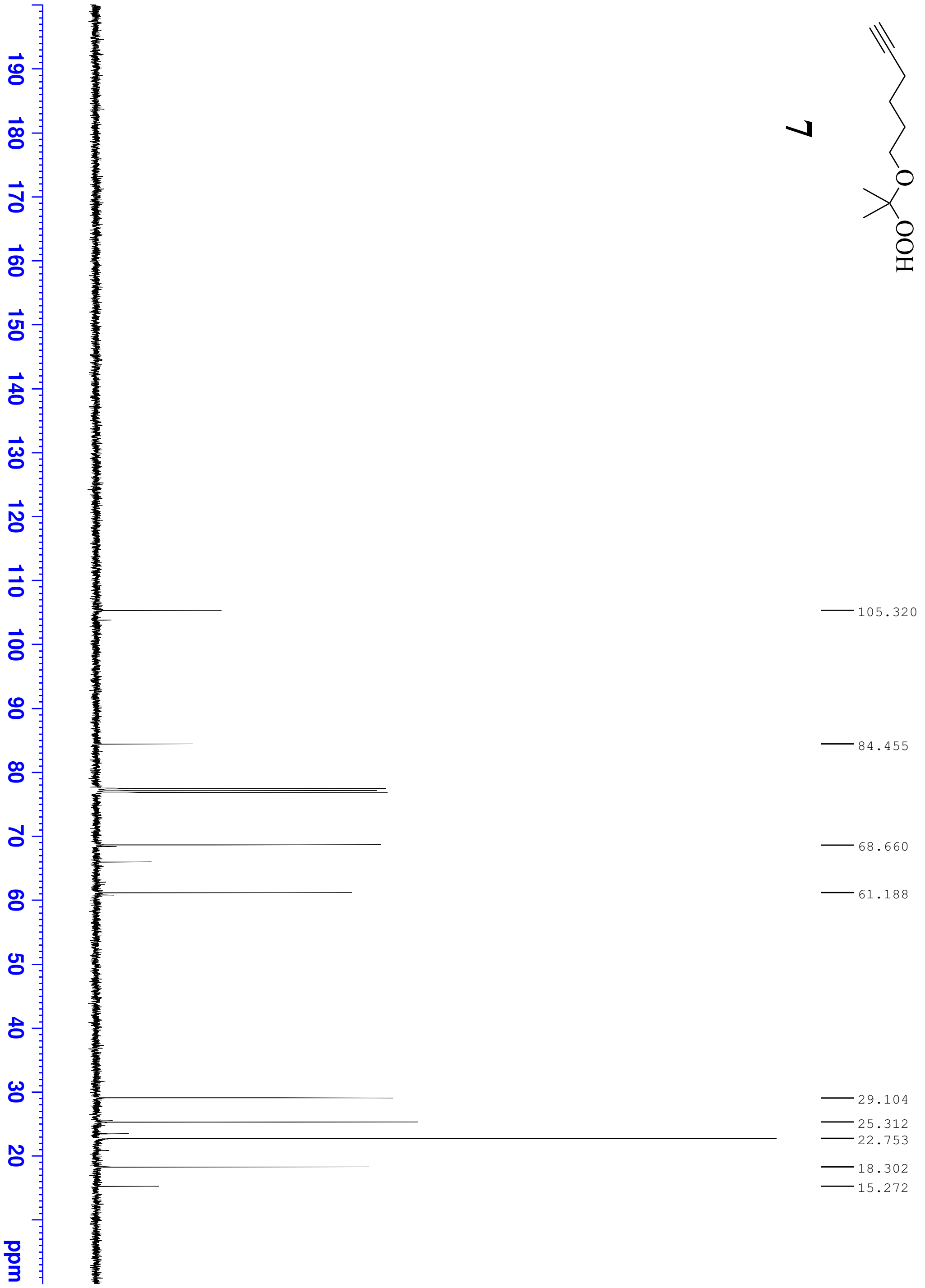

7
O
OOH
105.320
84.455
68.660
61.188
29.104
25.312
22.753
18.302
15.272
190
180
170
160
150
140
130
120
110
100
90
80
70
60
50
40
30
20
ppm

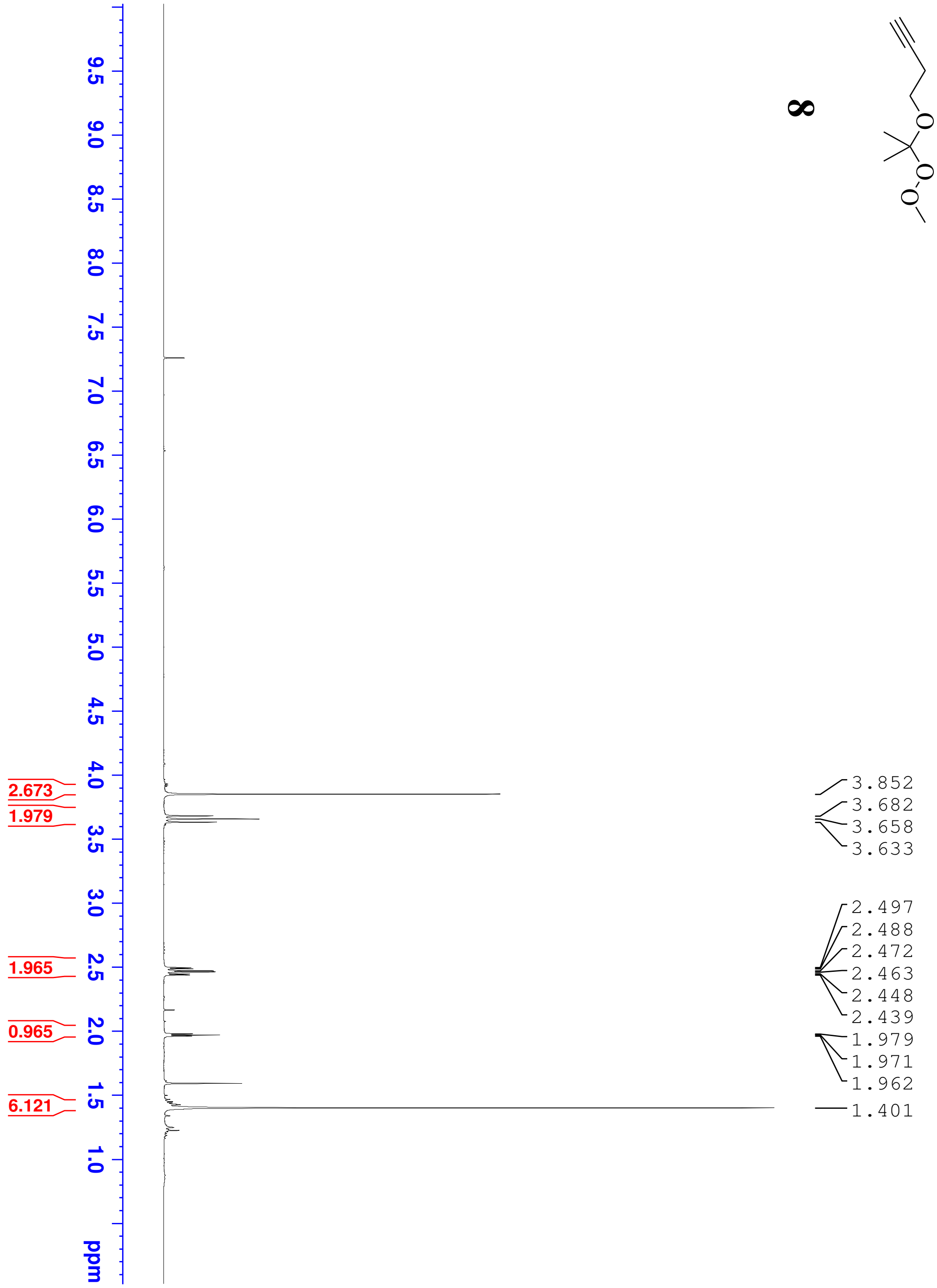
8
3.852
3.682
3.658
3.633
2.497
2.488
2.472
2.463
2.448
2.439
1.979
1.971
1.962
1.401
2.673
1.979
1.965
0.965
6.121
ppm

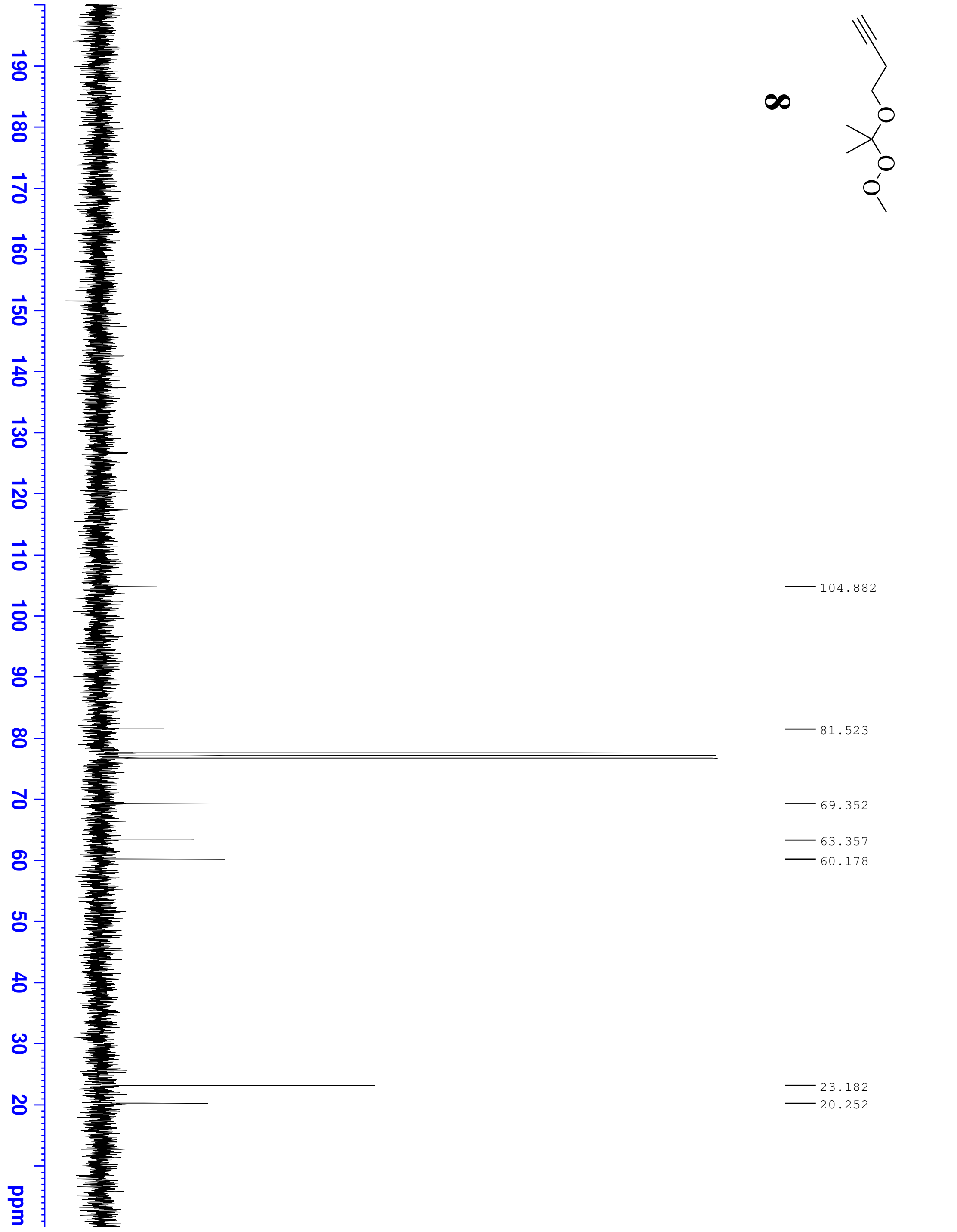
8
104.882
81.523
69.352
63.357
60.178
23.182
20.252
190
180
170
160
150
140
130
120
110
100
90
80
70
60
50
40
30
20
ppm

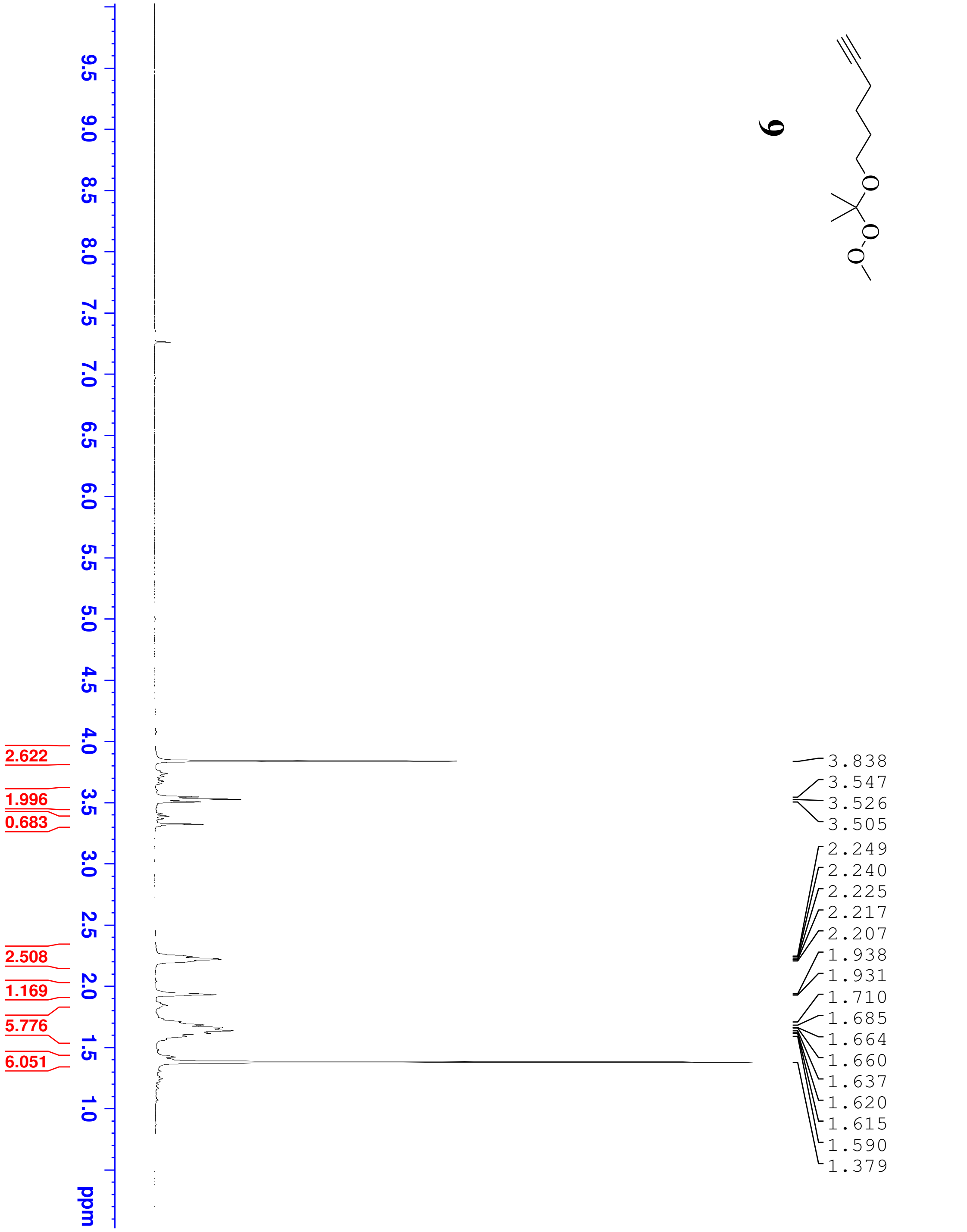
9
3.838
3.547
3.526
3.505
2.249
2.240
2.225
2.217
2.207
1.938
1.931
1.710
1.685
1.664
1.660
1.637
1.620
1.615
1.590
1.379
2.622
1.996
0.683
2.508
1.169
5.776
6.051
9.5
9.0
8.5
8.0
7.5
7.0
6.5
6.0
5.5
5.0
4.5
4.0
3.5
3.0
2.5
2.0
1.5
1.0
ppm

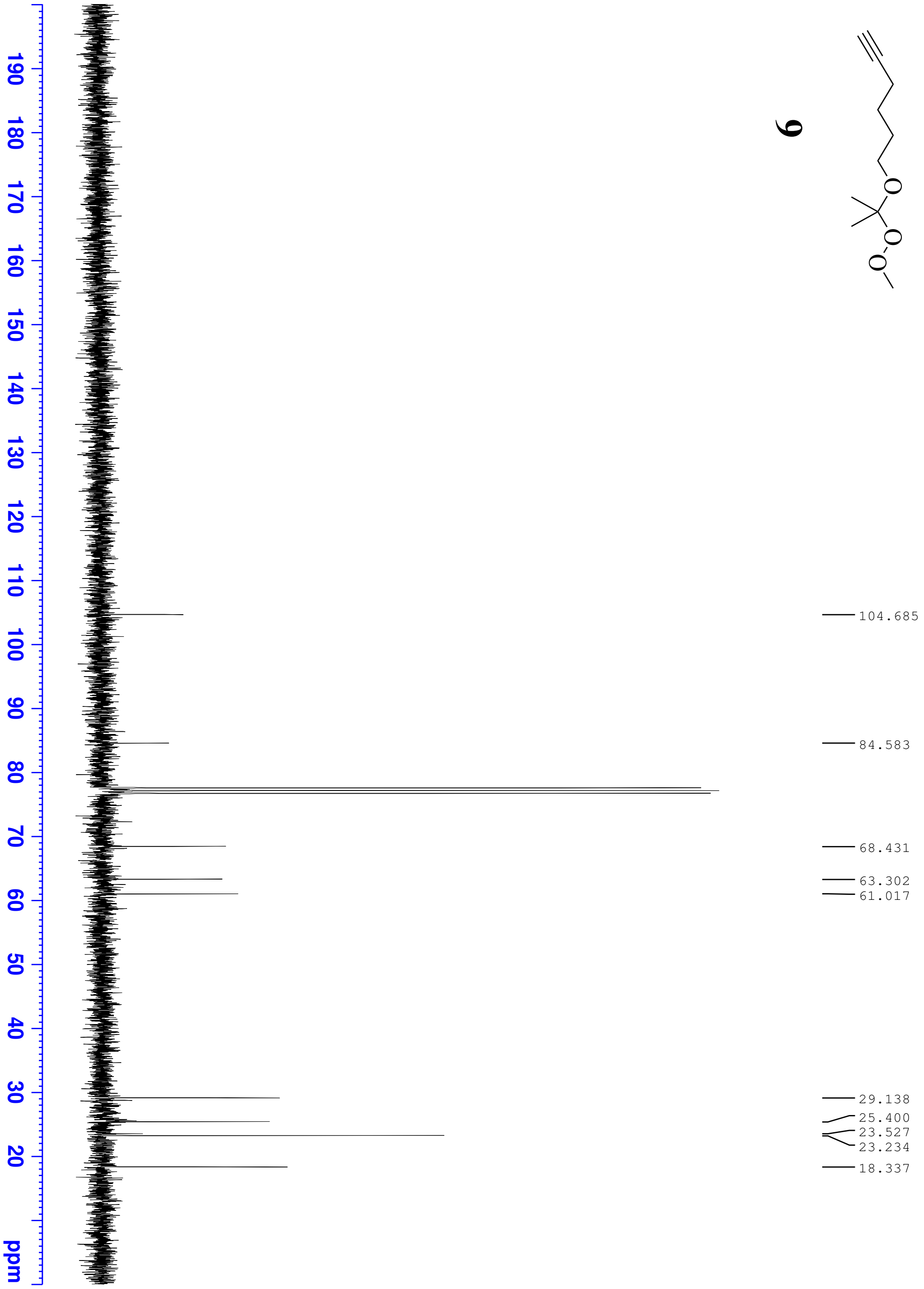
9
104.685
84.583
68.431
63.302
61.017
29.138
25.400
23.527
23.234
18.337
ppm

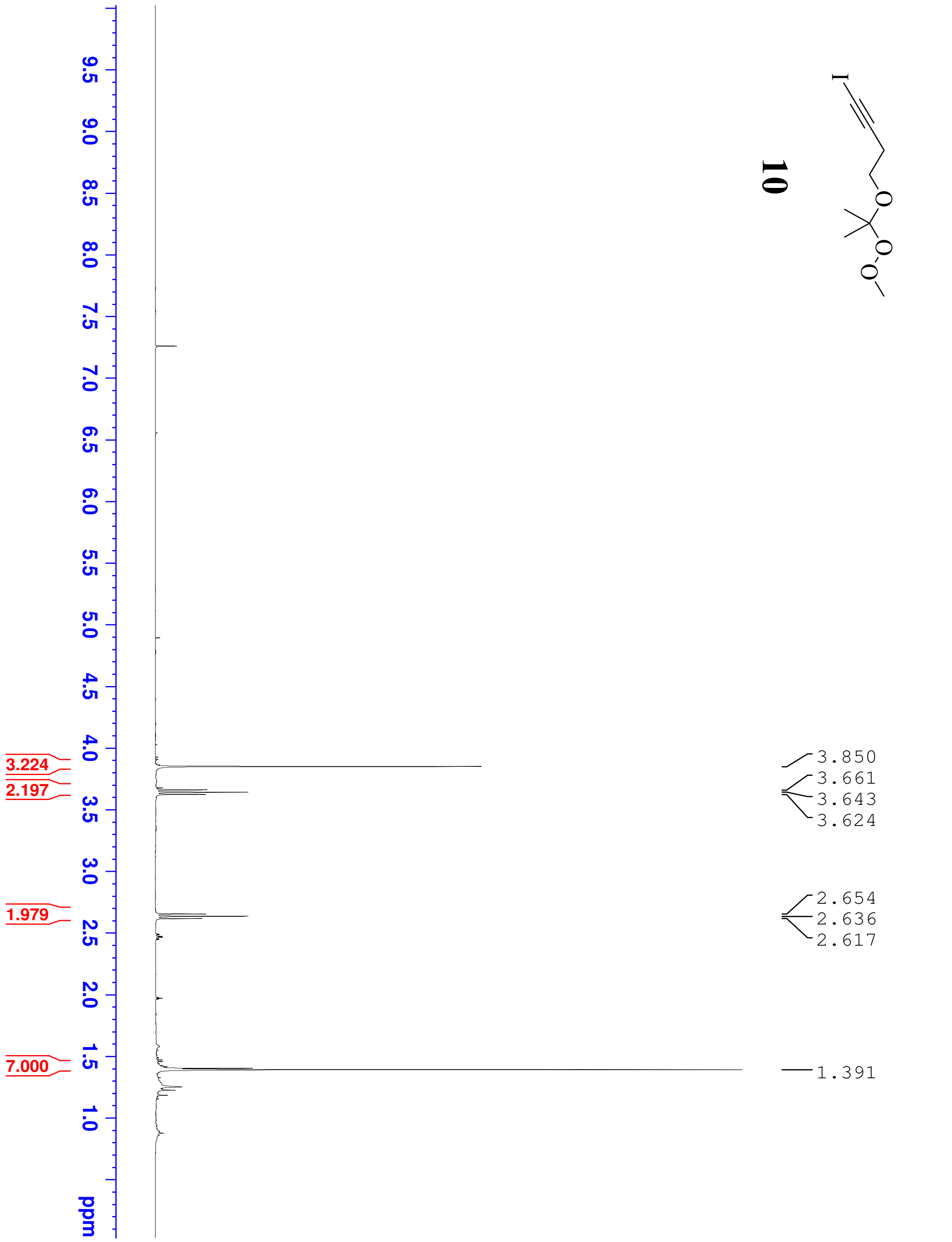
10
3.850
3.661
3.643
3.624
2.654
2.636
2.617
1.391
3.224
2.197
1.979
7.000
ppm

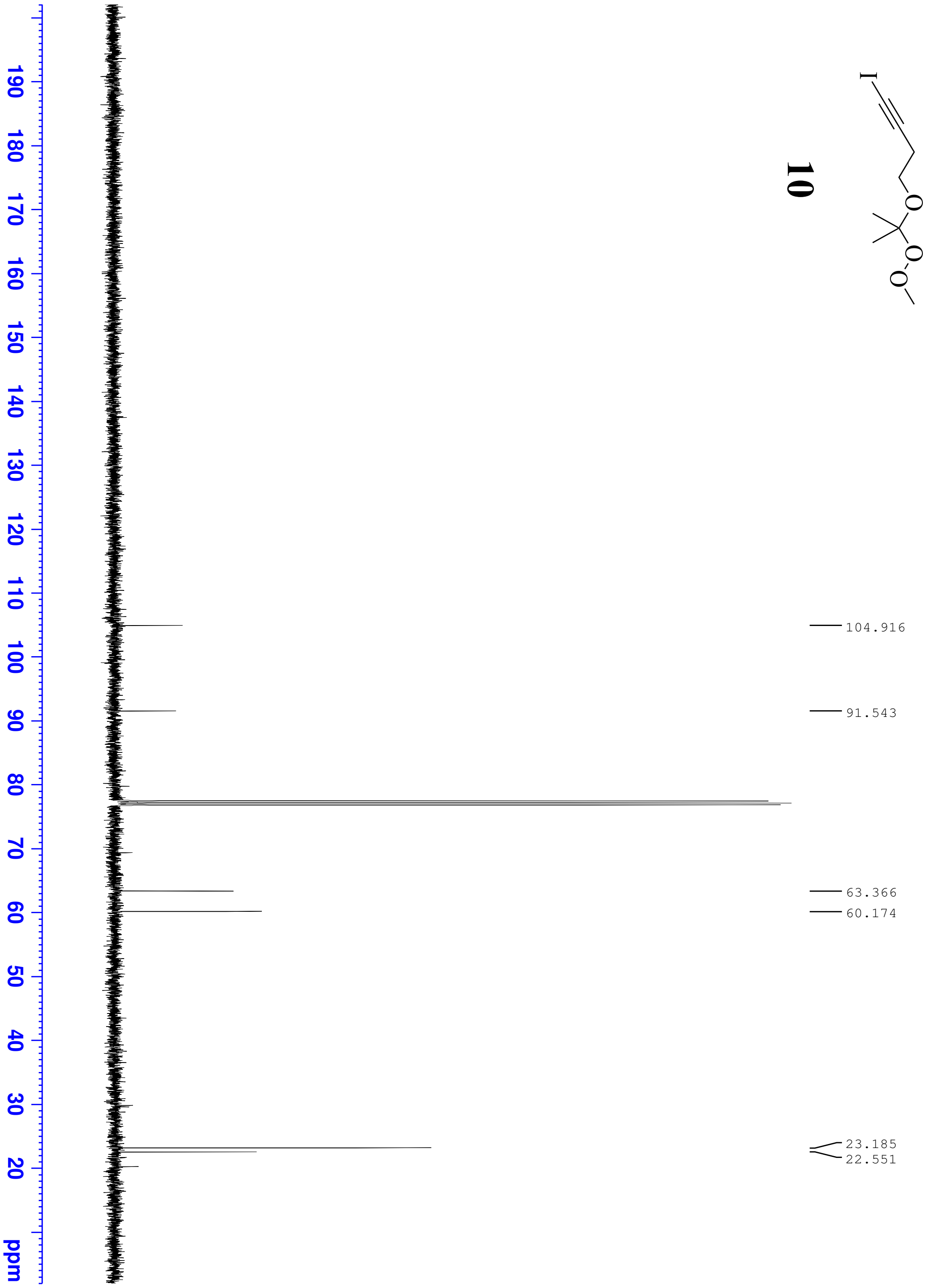

10

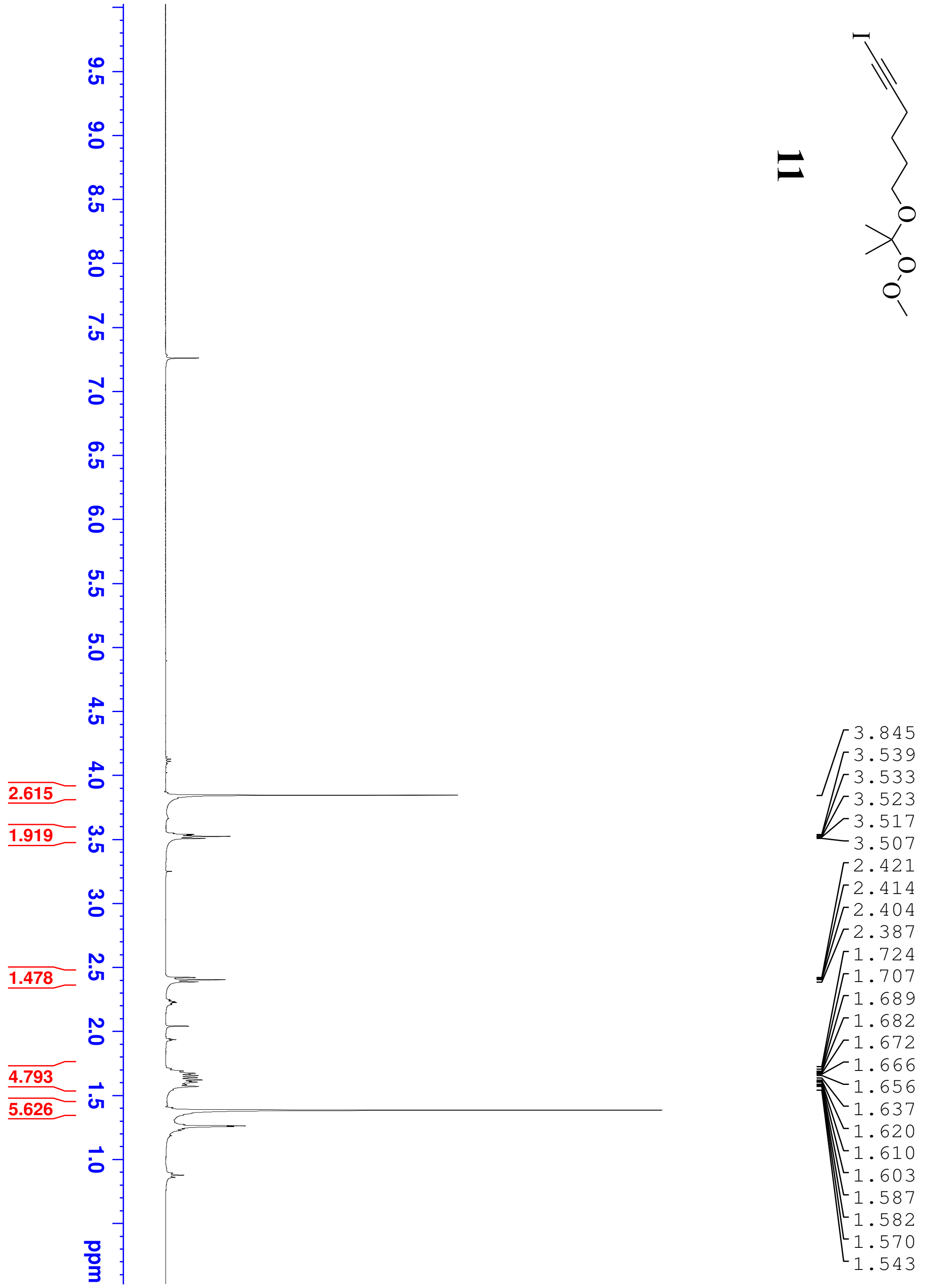
11
3.845
3.539
3.533
3.523
3.517
3.507
2.421
2.414
2.404
2.387
1.724
1.707
1.689
1.682
1.672
1.666
1.656
1.637
1.620
1.610
1.603
1.587
1.582
1.570
1.543
2.615
1.919
1.478
4.793
5.626
ppm

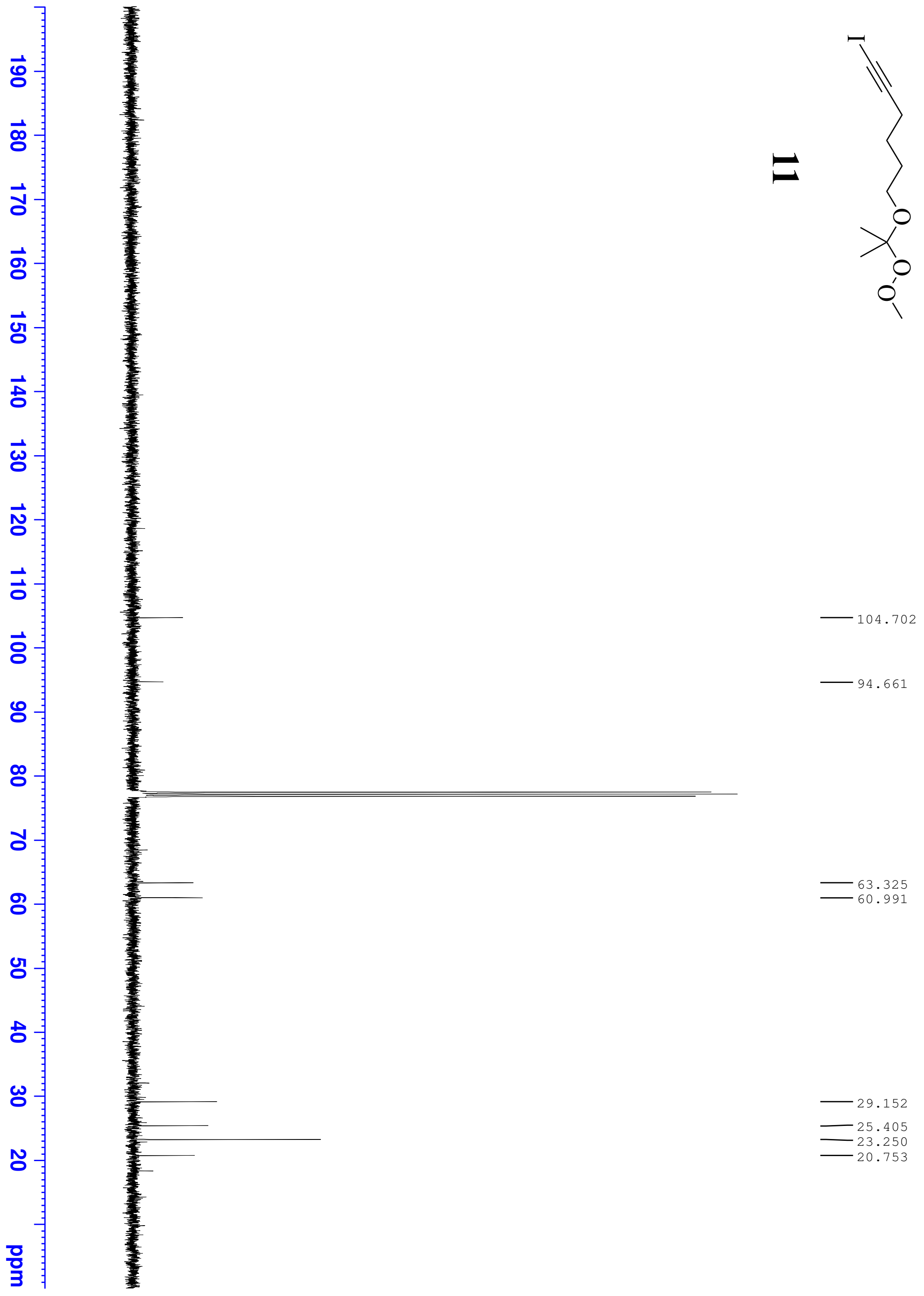
11
104.702
94.661
63.325
60.991
29.152
25.405
23.250
20.753
190
180
170
160
150
140
130
120
110
100
90
80
70
60
50
40
30
20
ppm

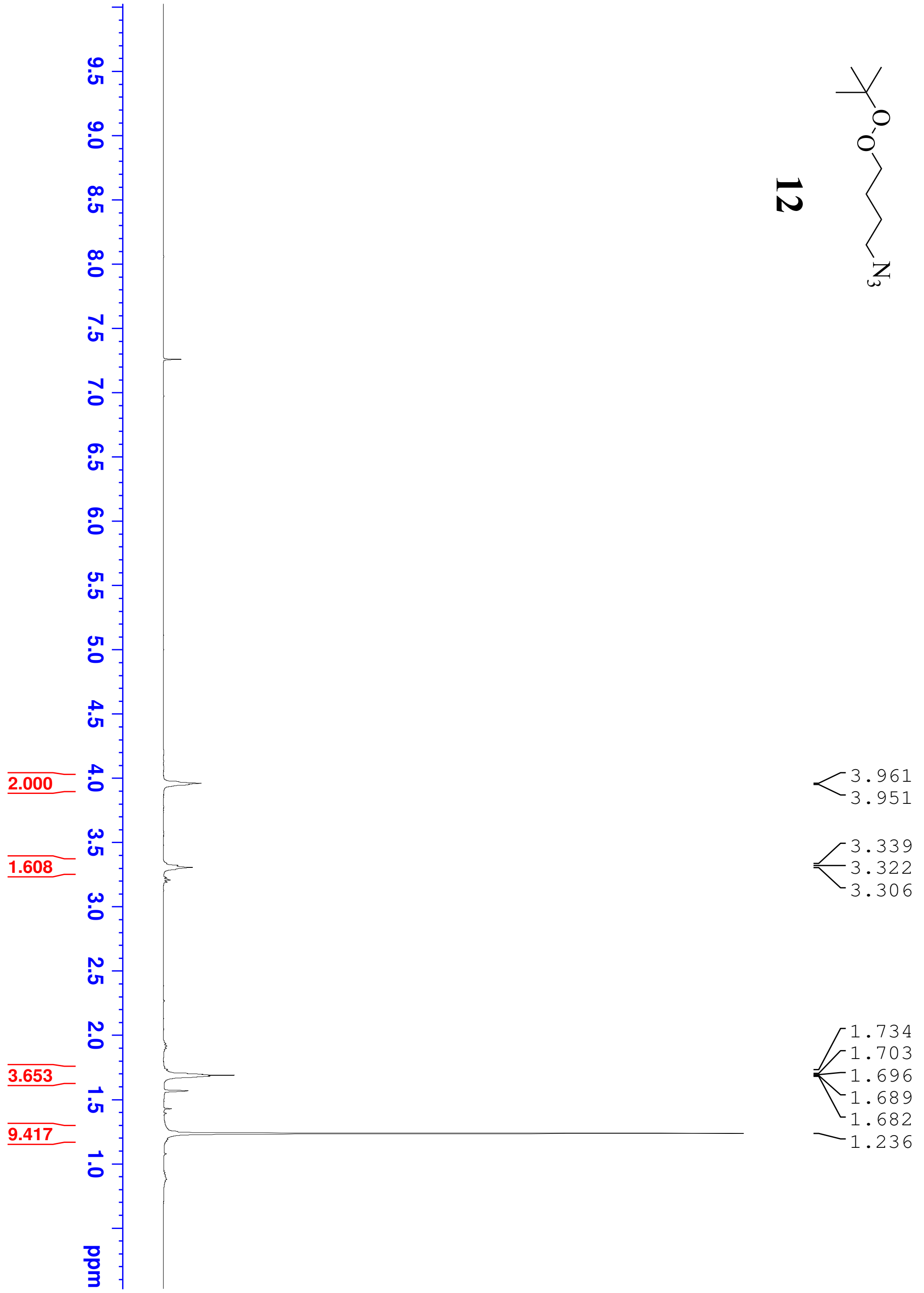
12
O
O
N3
3.961
3.951
3.339
3.322
3.306
1.734
1.703
1.696
1.689
1.682
1.236
2.000
1.608
3.653
9.417
9.5
9.0
8.5
8.0
7.5
7.0
6.5
6.0
5.5
5.0
4.5
4.0
3.5
3.0
2.5
2.0
1.5
1.0
ppm

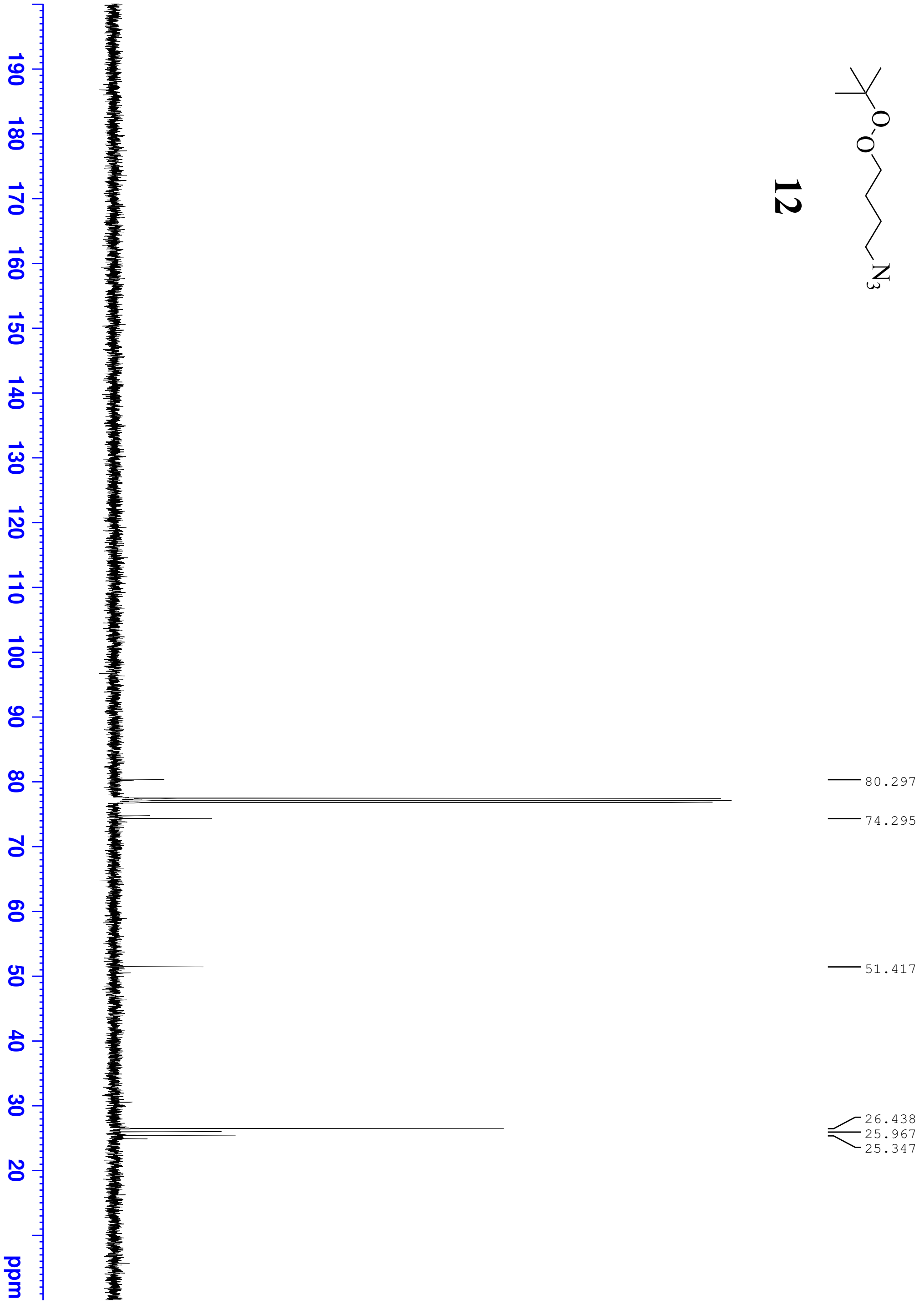
12
N_3
80.297
74.295
51.417
26.438
25.967
25.347
ppm
190
180
170
160
150
140
130
120
110
100
90
80
70
60
50
40
30
20

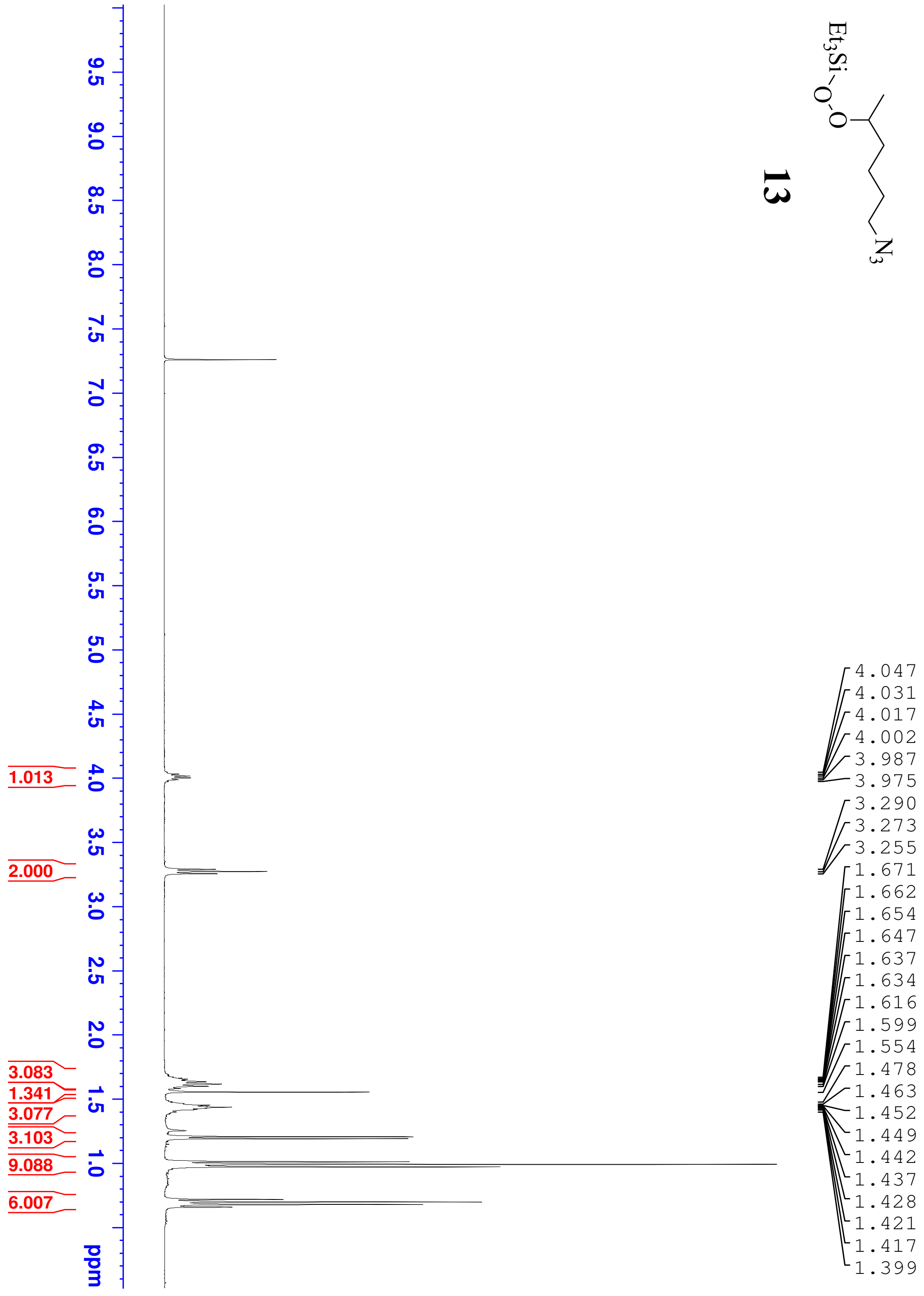

Et3Si
O
O
N3
13
4.047
4.031
4.017
4.002
3.987
3.975
3.290
3.273
3.255
1.671
1.662
1.654
1.647
1.637
1.634
1.616
1.599
1.554
1.478
1.463
1.452
1.449
1.442
1.437
1.428
1.421
1.417
1.399
1.013
2.000
3.083
1.341
3.077
3.103
9.088
6.007
9.5
9.0
8.5
8.0
7.5
7.0
6.5
6.0
5.5
5.0
4.5
4.0
3.5
3.0
2.5
2.0
1.5
1.0
ppm

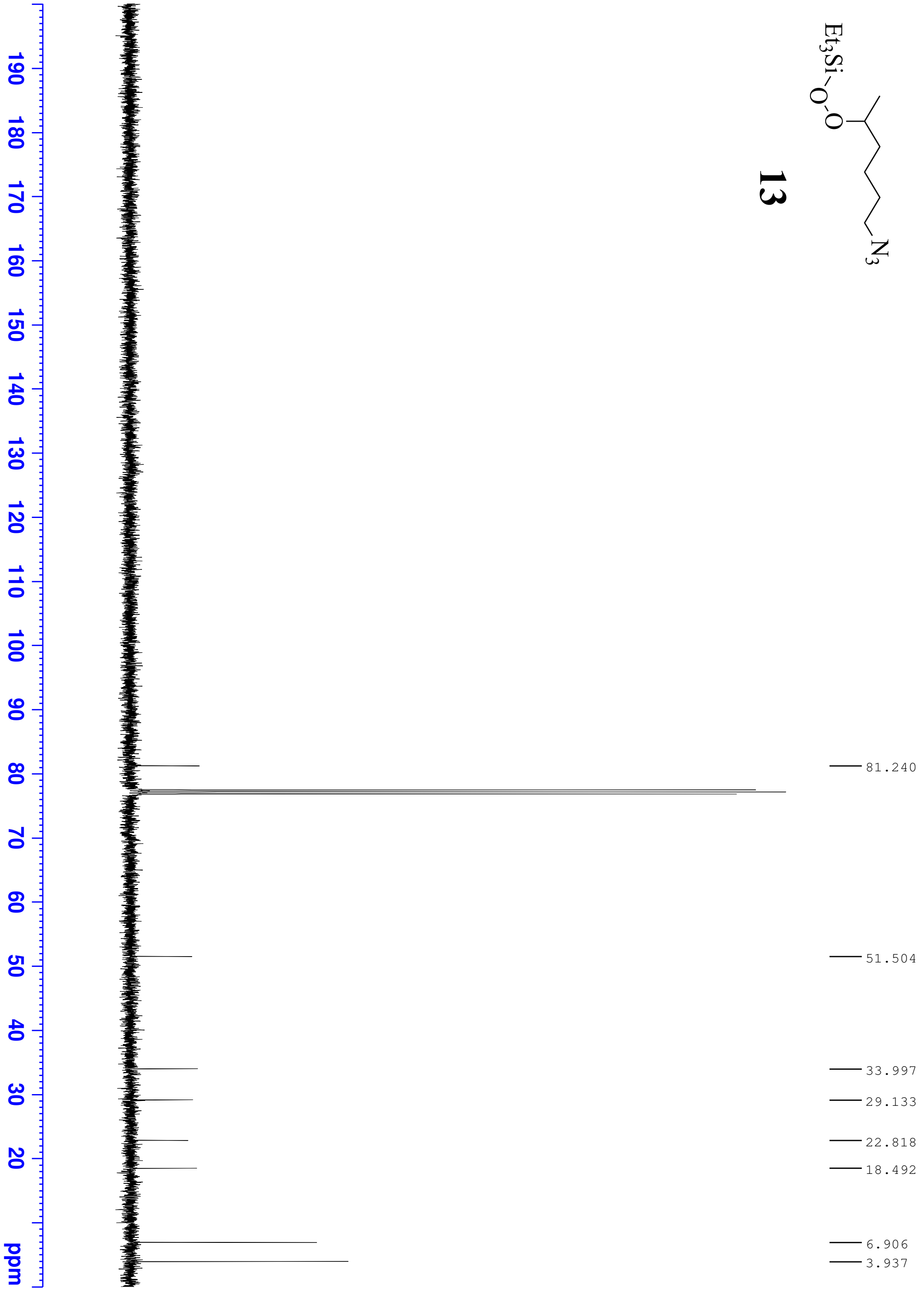

Et_3Si
O
O
N_3
13
81.240
51.504
33.997
29.133
22.818
18.492
6.906
3.937
190
180
170
160
150
140
130
120
110
100
90
80
70
60
50
40
30
20
ppm

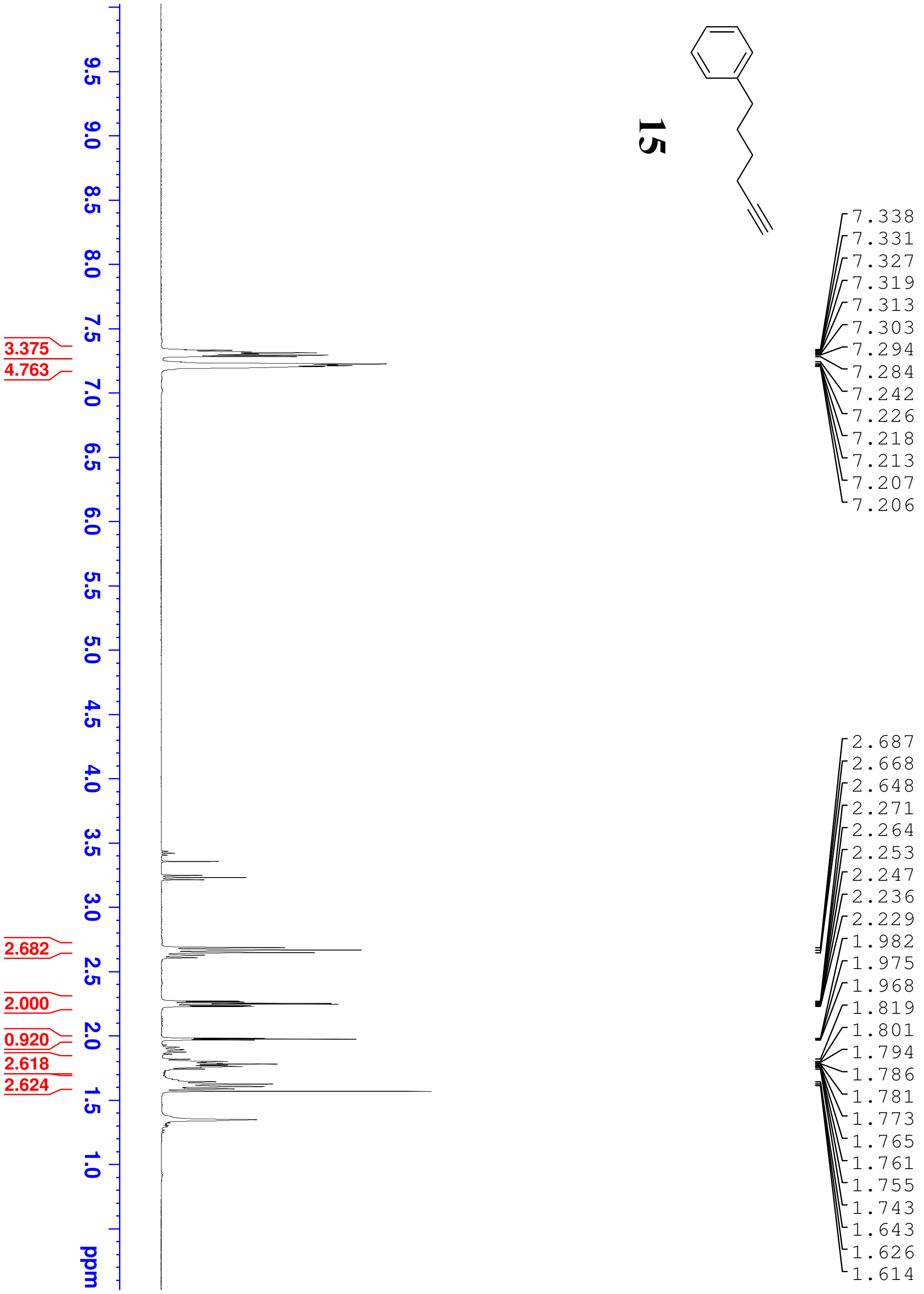
15
7.338
7.331
7.327
7.319
7.313
7.303
7.294
7.284
7.242
7.226
7.218
7.213
7.207
7.206
2.687
2.668
2.648
2.271
2.264
2.253
2.247
2.236
2.229
1.982
1.975
1.968
1.819
1.801
1.794
1.786
1.781
1.773
1.765
1.761
1.755
1.743
1.643
1.626
1.614
3.375
4.763
2.682
2.000
0.920
2.618
2.624
9.5
9.0
8.5
8.0
7.5
7.0
6.5
6.0
5.5
5.0
4.5
4.0
3.5
3.0
2.5
2.0
1.5
1.0
ppm

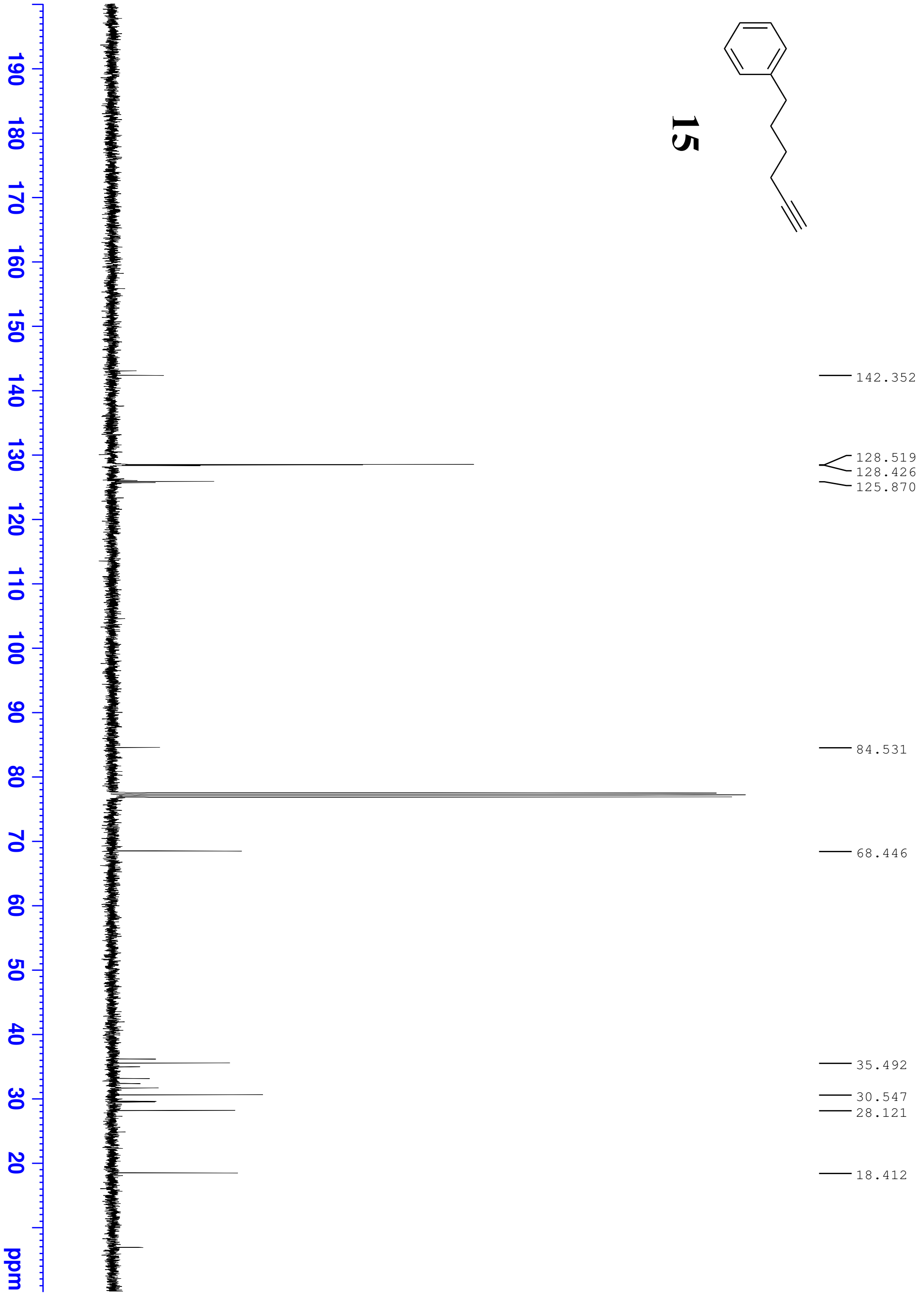
15
142.352
128.519
128.426
125.870
84.531
68.446
35.492
30.547
28.121
18.412
190
180
170
160
150
140
130
120
110
100
90
80
70
60
50
40
30
20
ppm

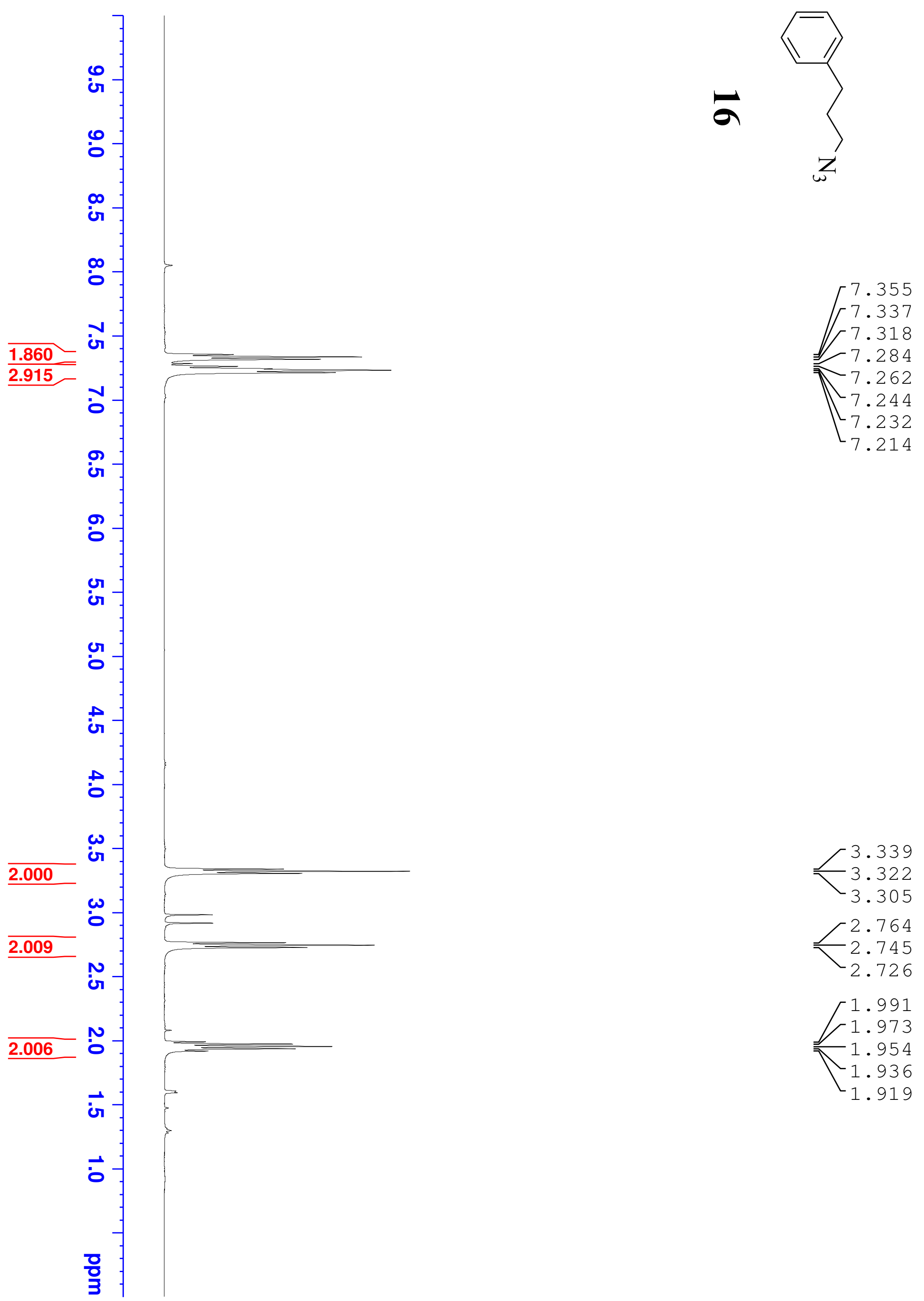
16
N_3
7.355
7.337
7.318
7.284
7.262
7.244
7.232
7.214
3.339
3.322
3.305
2.764
2.745
2.726
1.991
1.973
1.954
1.936
1.919
1.860
2.915
2.000
2.009
2.006
ppm

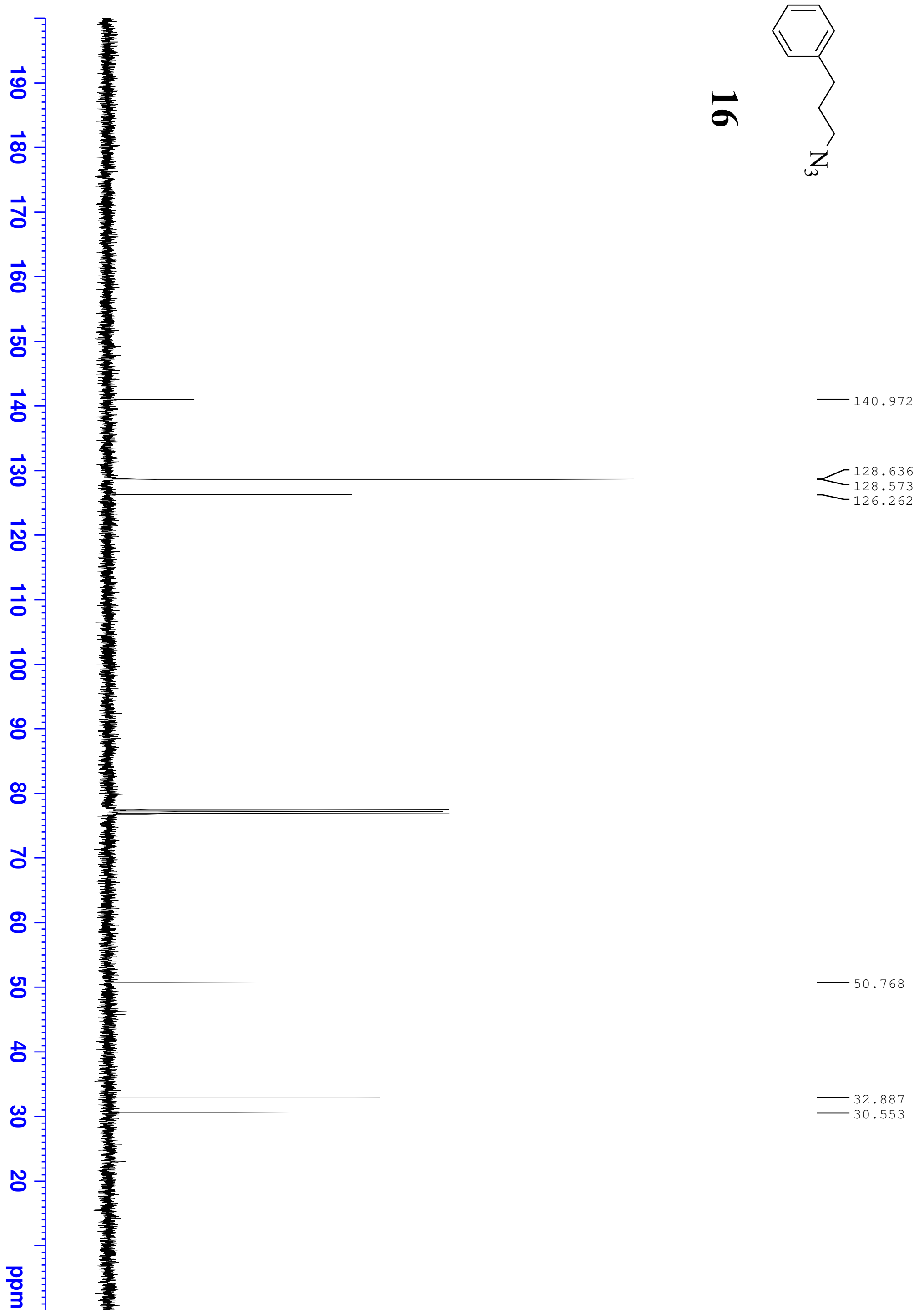
N3
140.972
128.636
128.573
126.262
50.768
32.887
30.553
190
180
170
160
150
140
130
120
110
100
90
80
70
60
50
40
30
20
ppm

16

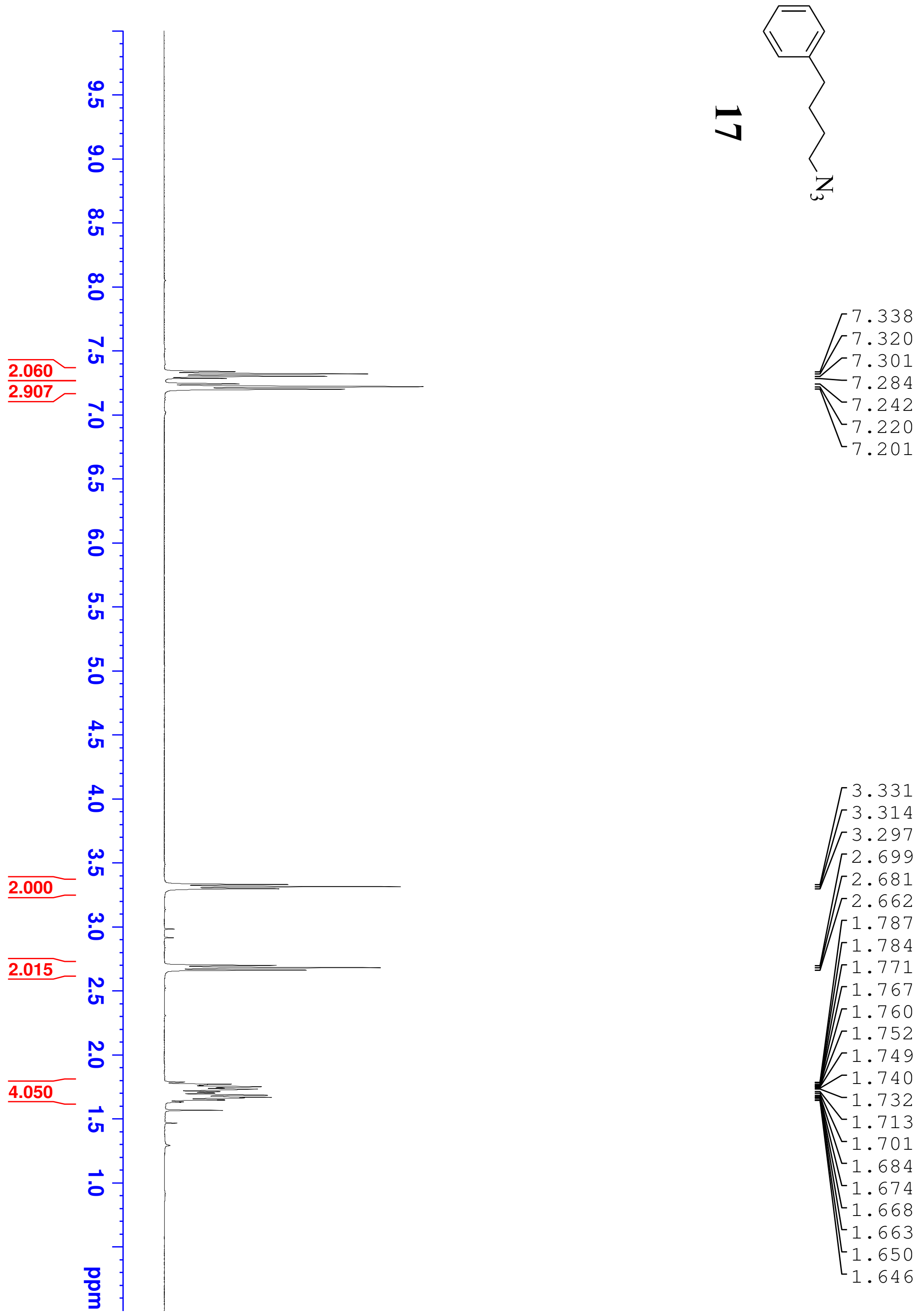
17
N3
7.338
7.320
7.301
7.284
7.242
7.220
7.201
3.331
3.314
3.297
2.699
2.681
2.662
1.787
1.784
1.771
1.767
1.760
1.752
1.749
1.740
1.732
1.713
1.701
1.684
1.674
1.668
1.663
1.650
1.646
2.060
2.907
2.000
2.015
4.050
ppm

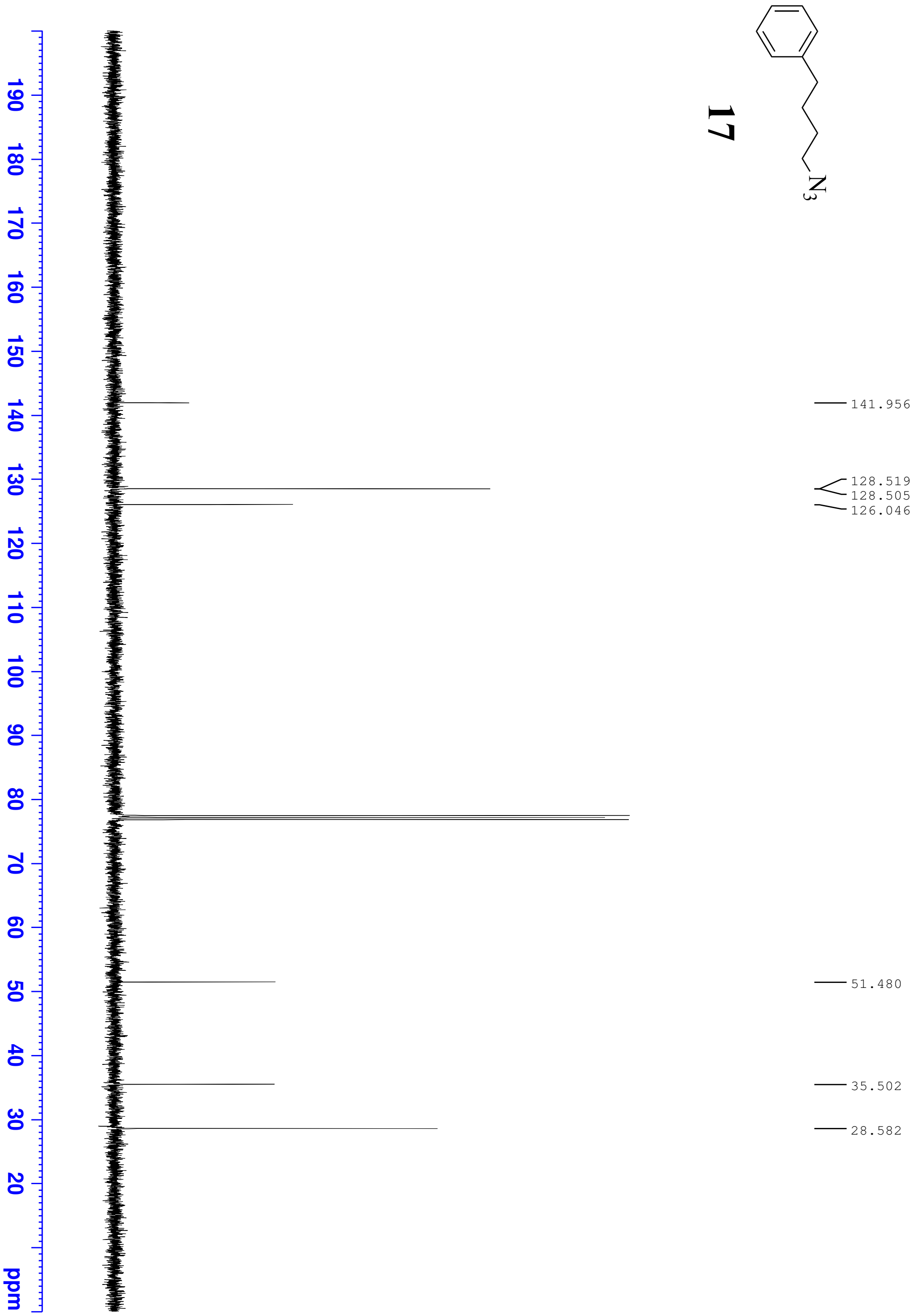
141.956
128.519
128.505
126.046
51.480
35.502
28.582
190
180
170
160
150
140
130
120
110
100
90
80
70
60
50
40
30
20
ppm
N3

17

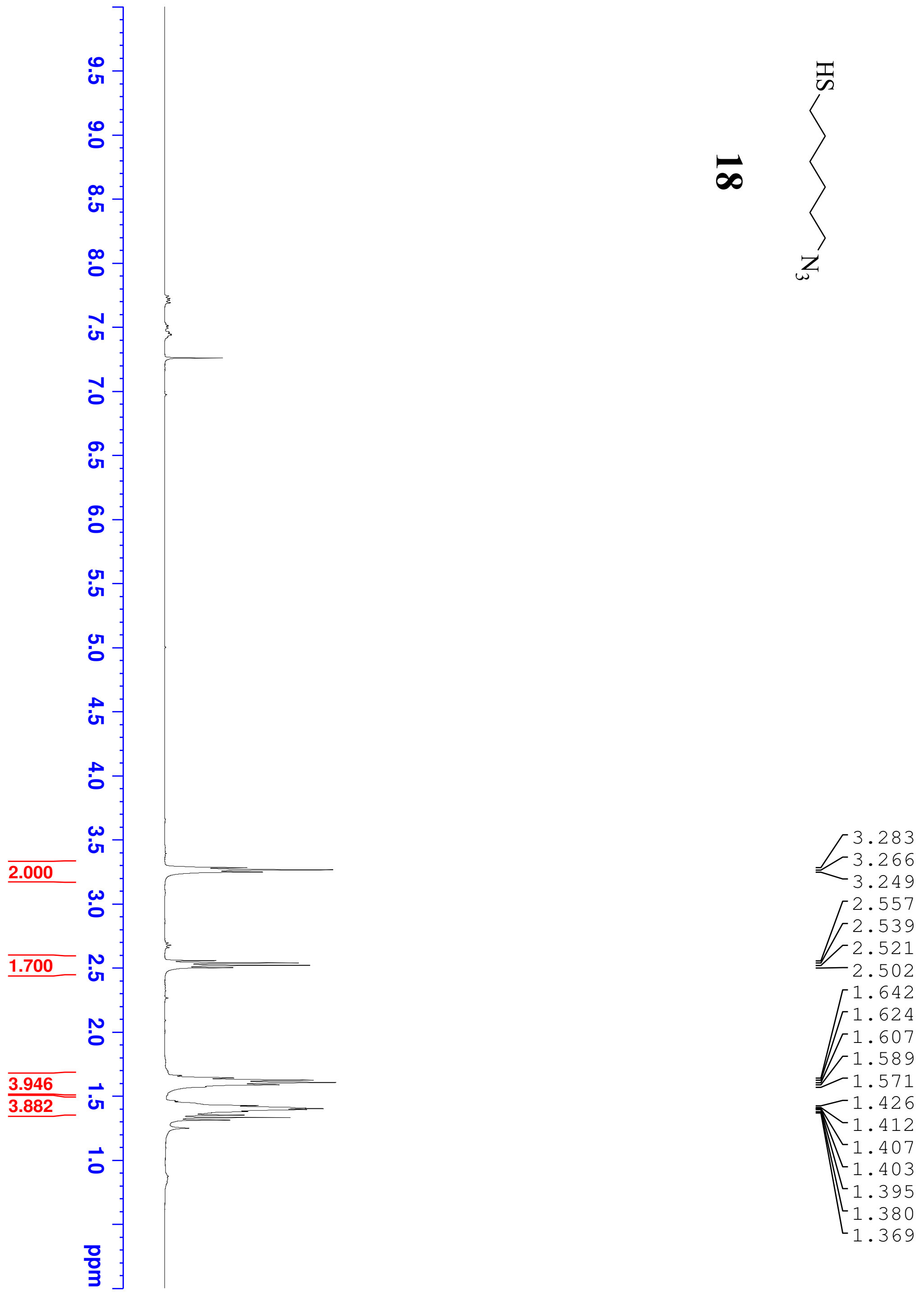
HS
N3
18
3.283
3.266
3.249
2.557
2.539
2.521
2.502
1.642
1.624
1.607
1.589
1.571
1.426
1.412
1.407
1.403
1.395
1.380
1.369
2.000
1.700
3.946
3.882
9.5
9.0
8.5
8.0
7.5
7.0
6.5
6.0
5.5
5.0
4.5
4.0
3.5
3.0
2.5
2.0
1.5
1.0
ppm

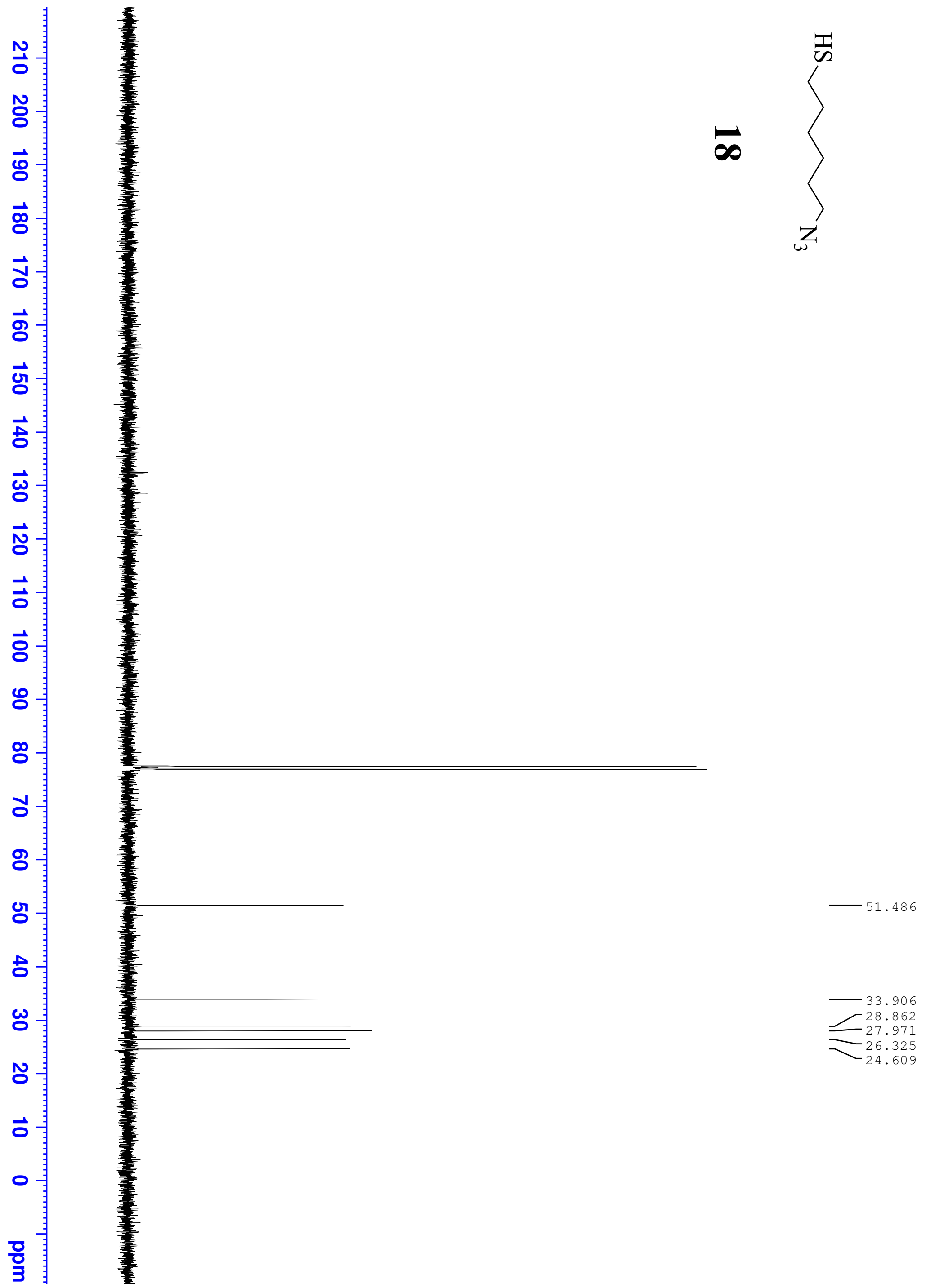
HS
N3
18
51.486
33.906
28.862
27.971
26.325
24.609
210
200
190
180
170
160
150
140
130
120
110
100
90
80
70
60
50
40
30
20
10
0
ppm

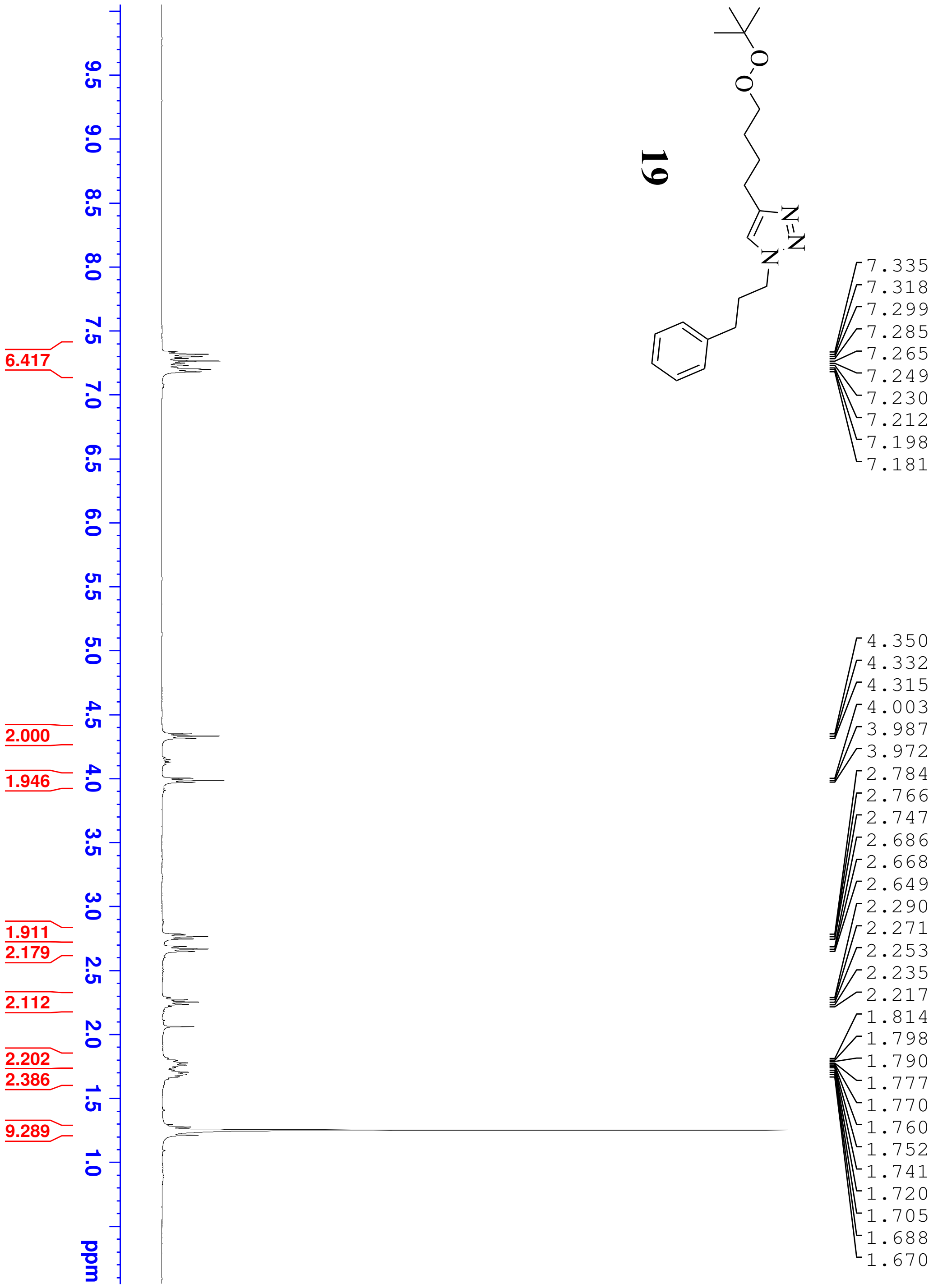
19
7.335
7.318
7.299
7.285
7.265
7.249
7.230
7.212
7.198
7.181
4.350
4.332
4.315
4.003
3.987
3.972
2.784
2.766
2.747
2.686
2.668
2.649
2.290
2.271
2.253
2.235
2.217
1.814
1.798
1.790
1.777
1.770
1.760
1.752
1.741
1.720
1.705
1.688
1.670
6.417
2.000
1.946
1.911
2.179
2.112
2.202
2.386
9.289
9.5
9.0
8.5
8.0
7.5
7.0
6.5
6.0
5.5
5.0
4.5
4.0
3.5
3.0
2.5
2.0
1.5
1.0
ppm

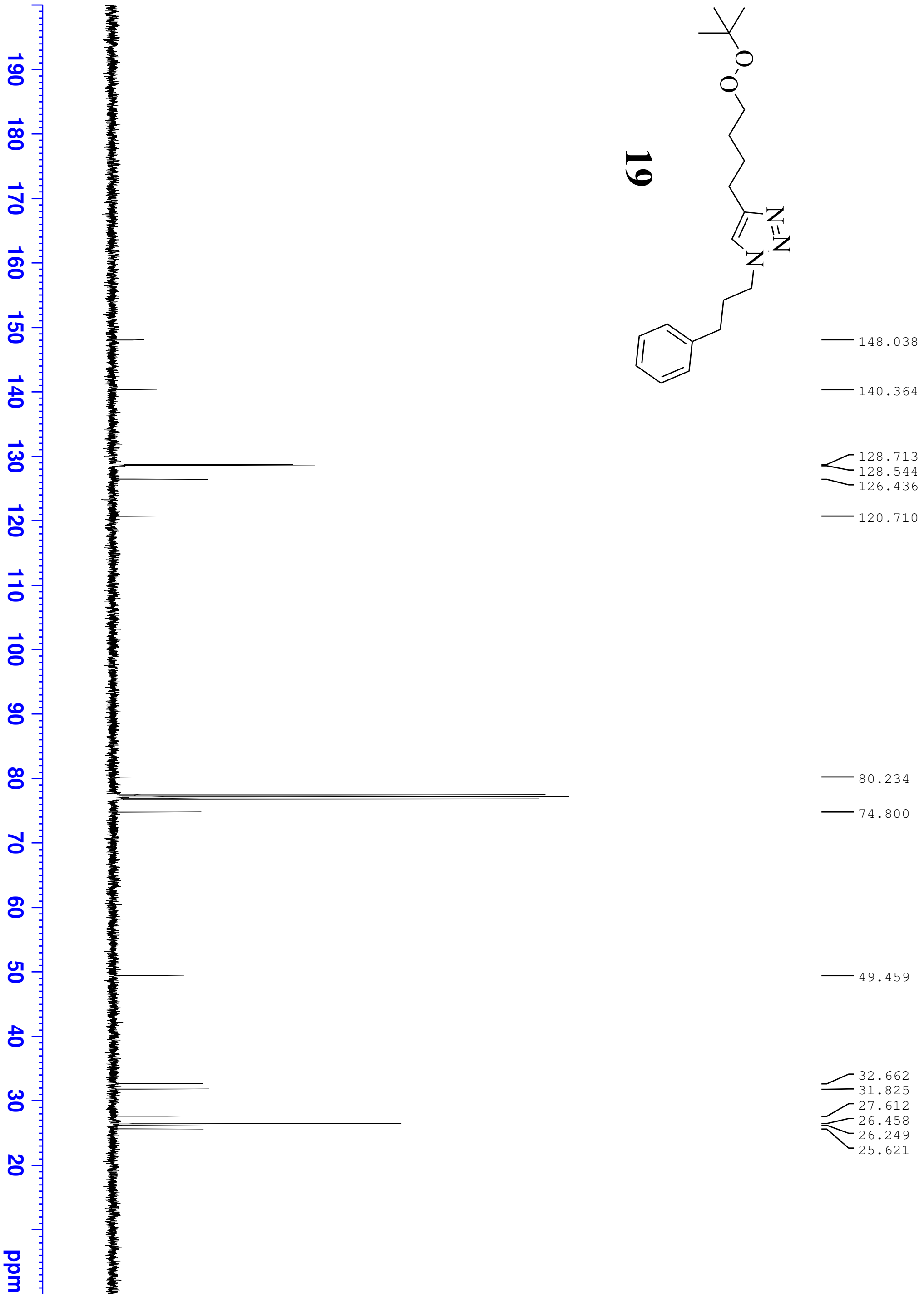
19
148.038
140.364
128.713
128.544
126.436
120.710
80.234
74.800
49.459
32.662
31.825
27.612
26.458
26.249
25.621
ppm

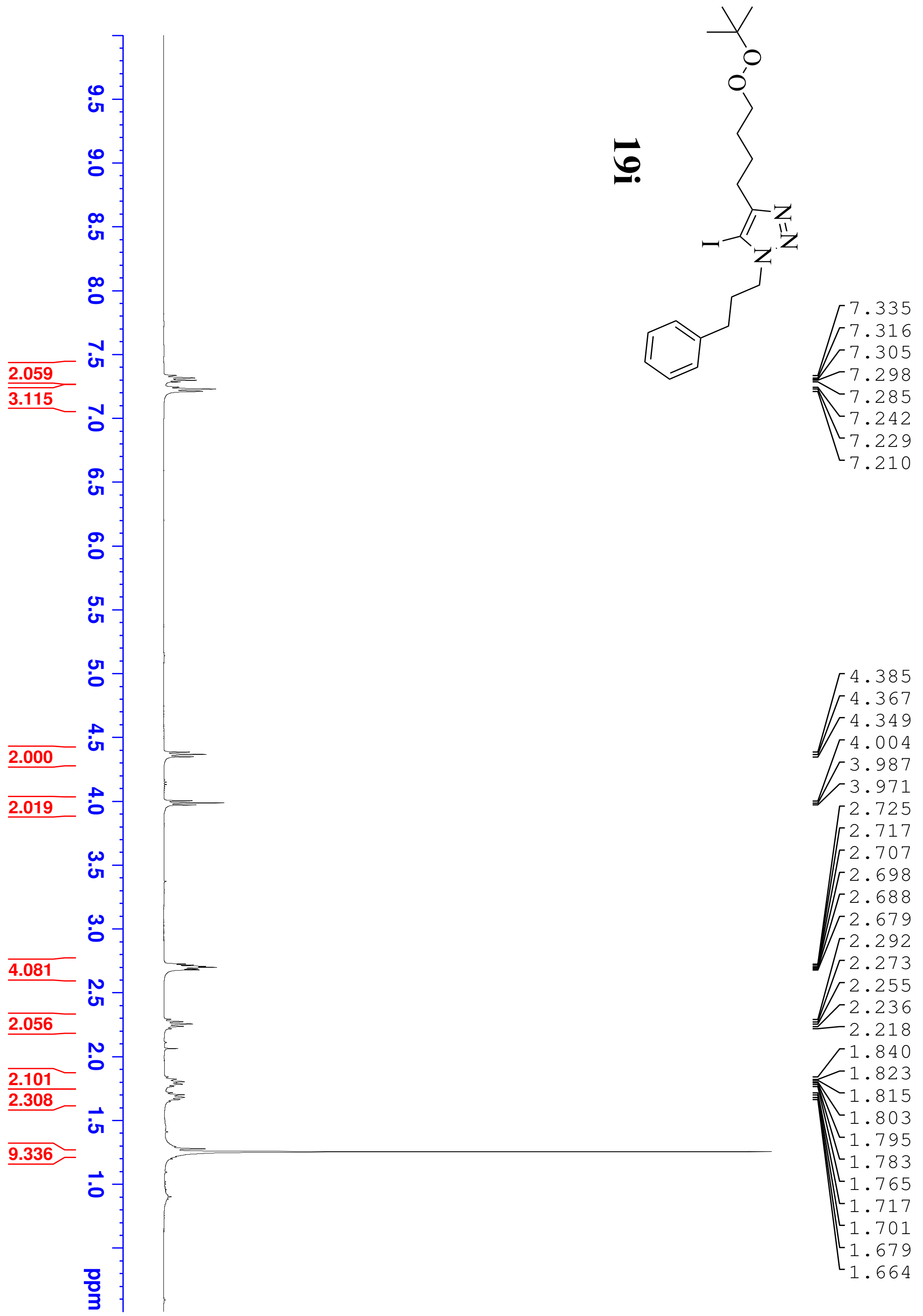
19i
I
N
N=N
O
O
7.335
7.316
7.305
7.298
7.285
7.242
7.229
7.210
4.385
4.367
4.349
4.004
3.987
3.971
2.725
2.717
2.707
2.698
2.688
2.679
2.292
2.273
2.255
2.236
2.218
1.840
1.823
1.815
1.803
1.795
1.783
1.765
1.717
1.701
1.679
1.664
2.059
3.115
2.000
2.019
4.081
2.056
2.101
2.308
9.336
9.5 9.0 8.5 8.0 7.5 7.0 6.5 6.0 5.5 5.0 4.5 4.0 3.5 3.0 2.5 2.0 1.5 1.0 ppm

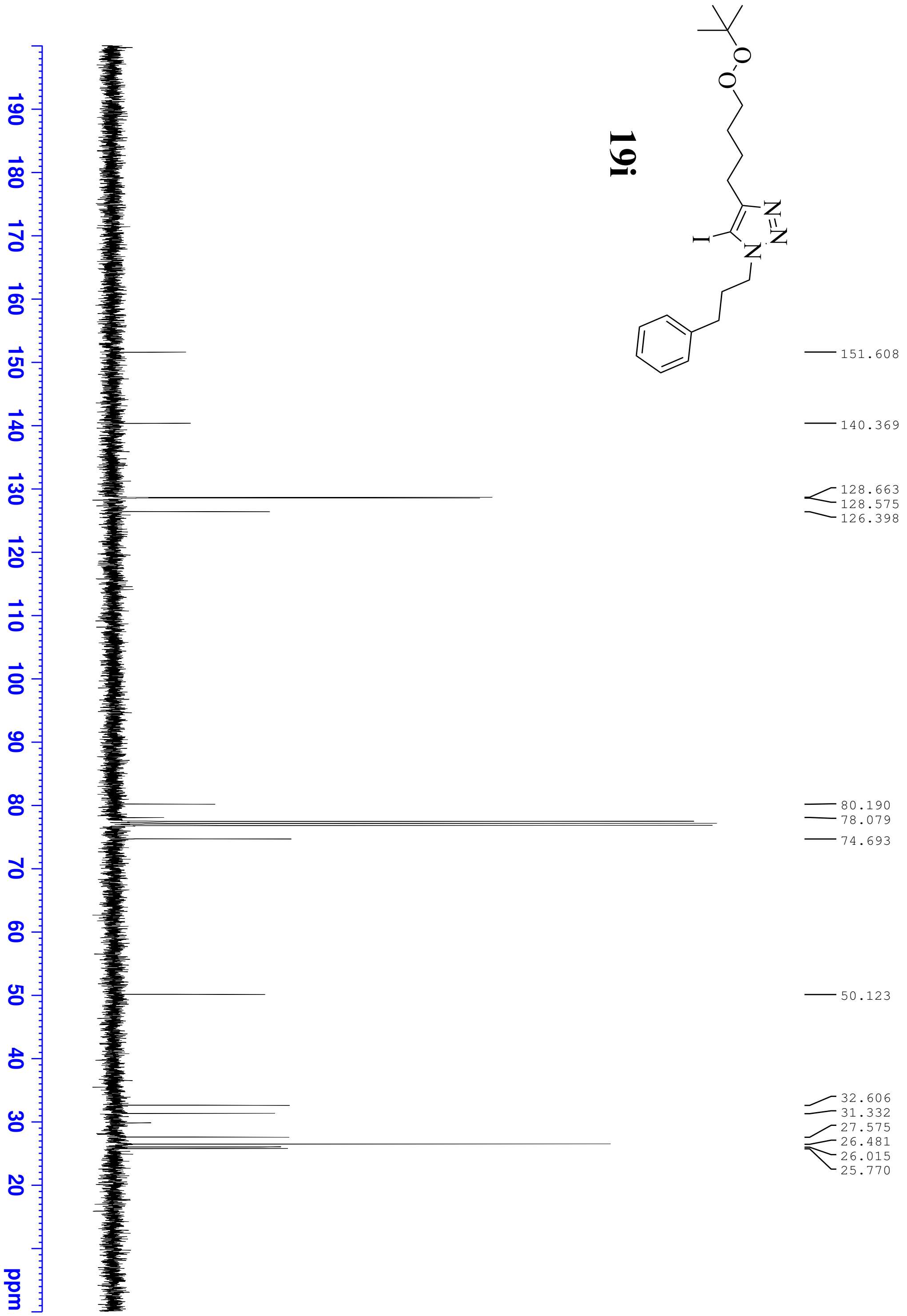
19i
151.608
140.369
128.663
128.575
126.398
80.190
78.079
74.693
50.123
32.606
31.332
27.575
26.481
26.015
25.770
190
180
170
160
150
140
130
120
110
100
90
80
70
60
50
40
30
20
ppm

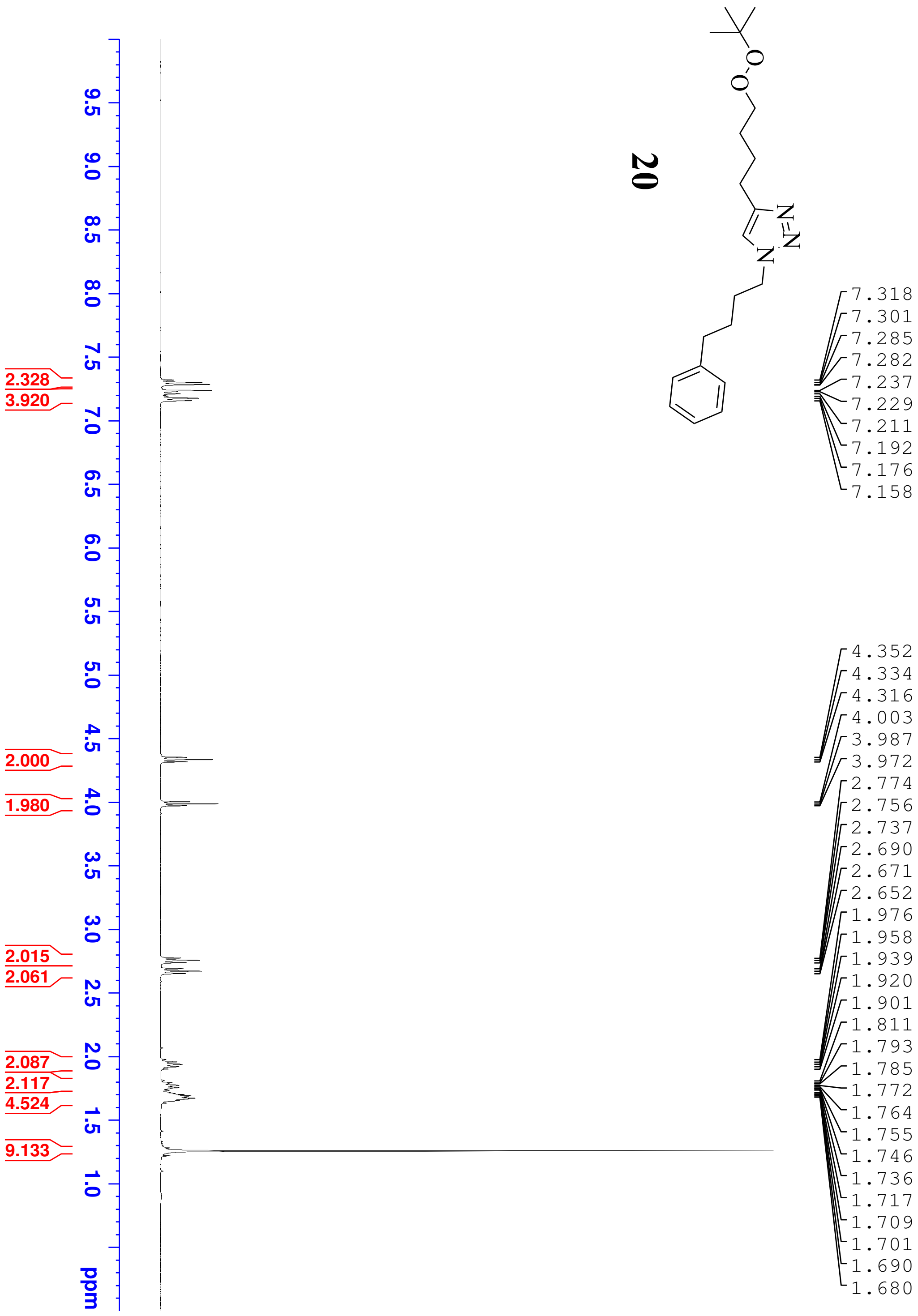
20
7.318
7.301
7.285
7.282
7.237
7.229
7.211
7.192
7.176
7.158
4.352
4.334
4.316
4.003
3.987
3.972
2.774
2.756
2.737
2.690
2.671
2.652
1.976
1.958
1.939
1.920
1.901
1.811
1.793
1.785
1.772
1.764
1.755
1.746
1.736
1.717
1.709
1.701
1.690
1.680
2.328
3.920
2.000
1.980
2.015
2.061
2.087
2.117
4.524
9.133
ppm

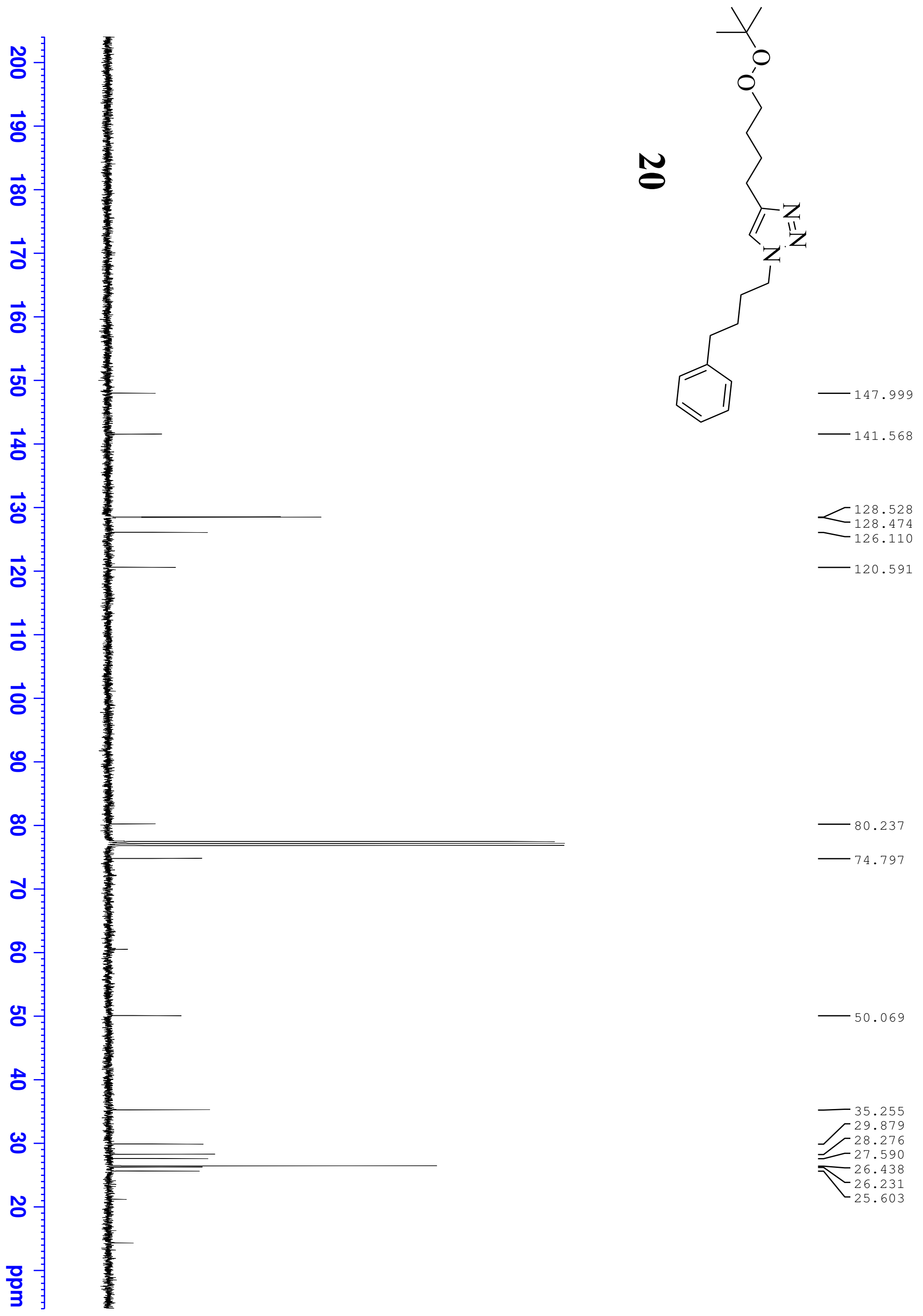
20
147.999
141.568
128.528
128.474
126.110
120.591
80.237
74.797
50.069
35.255
29.879
28.276
27.590
26.438
26.231
25.603
ppm

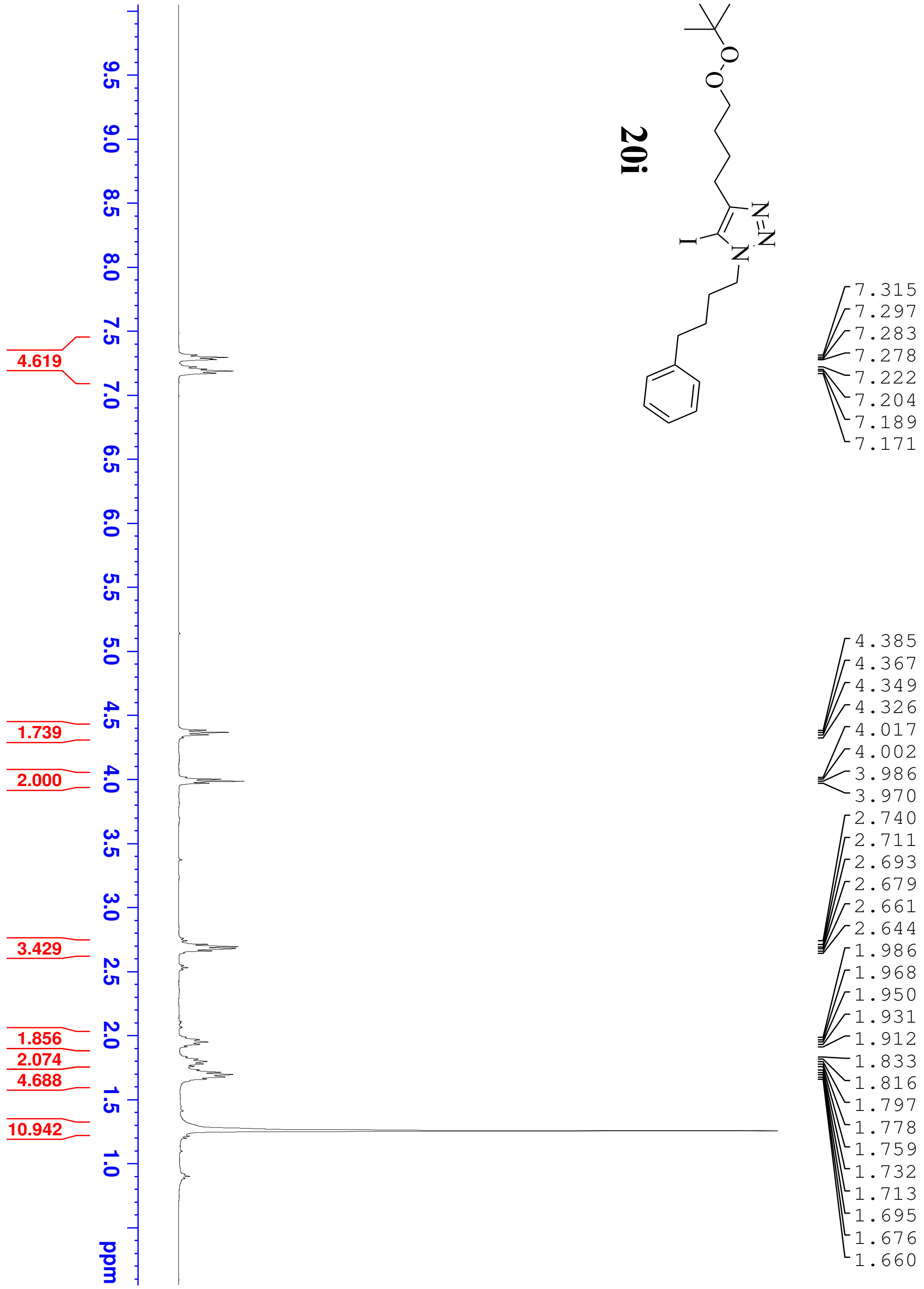
20i

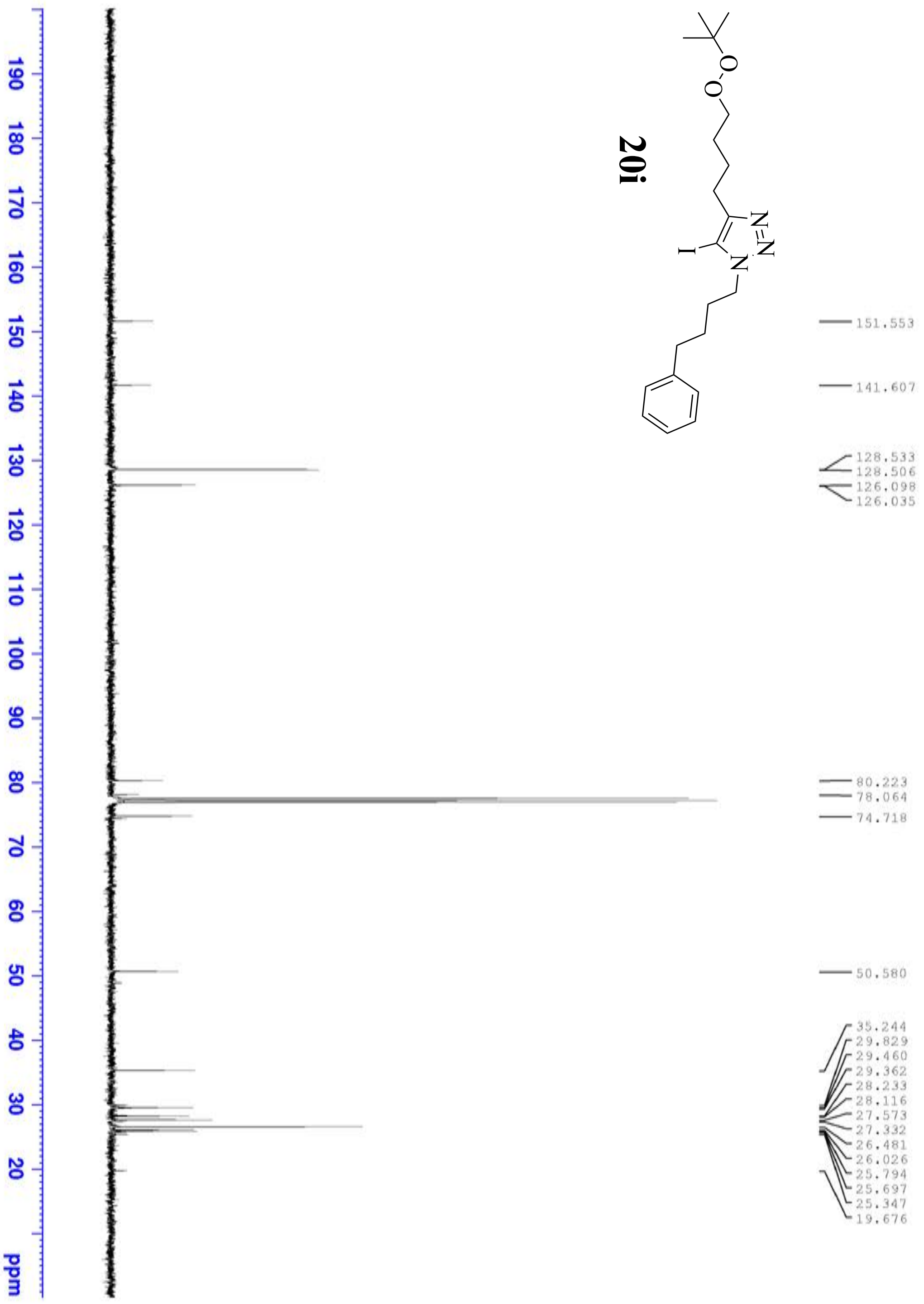
20i
151.553
141.607
128.533
128.506
126.098
126.035
80.223
78.064
74.718
50.580
35.244
29.829
29.460
29.362
28.233
28.116
27.573
27.332
26.481
26.026
25.794
25.697
25.347
19.676
ppm

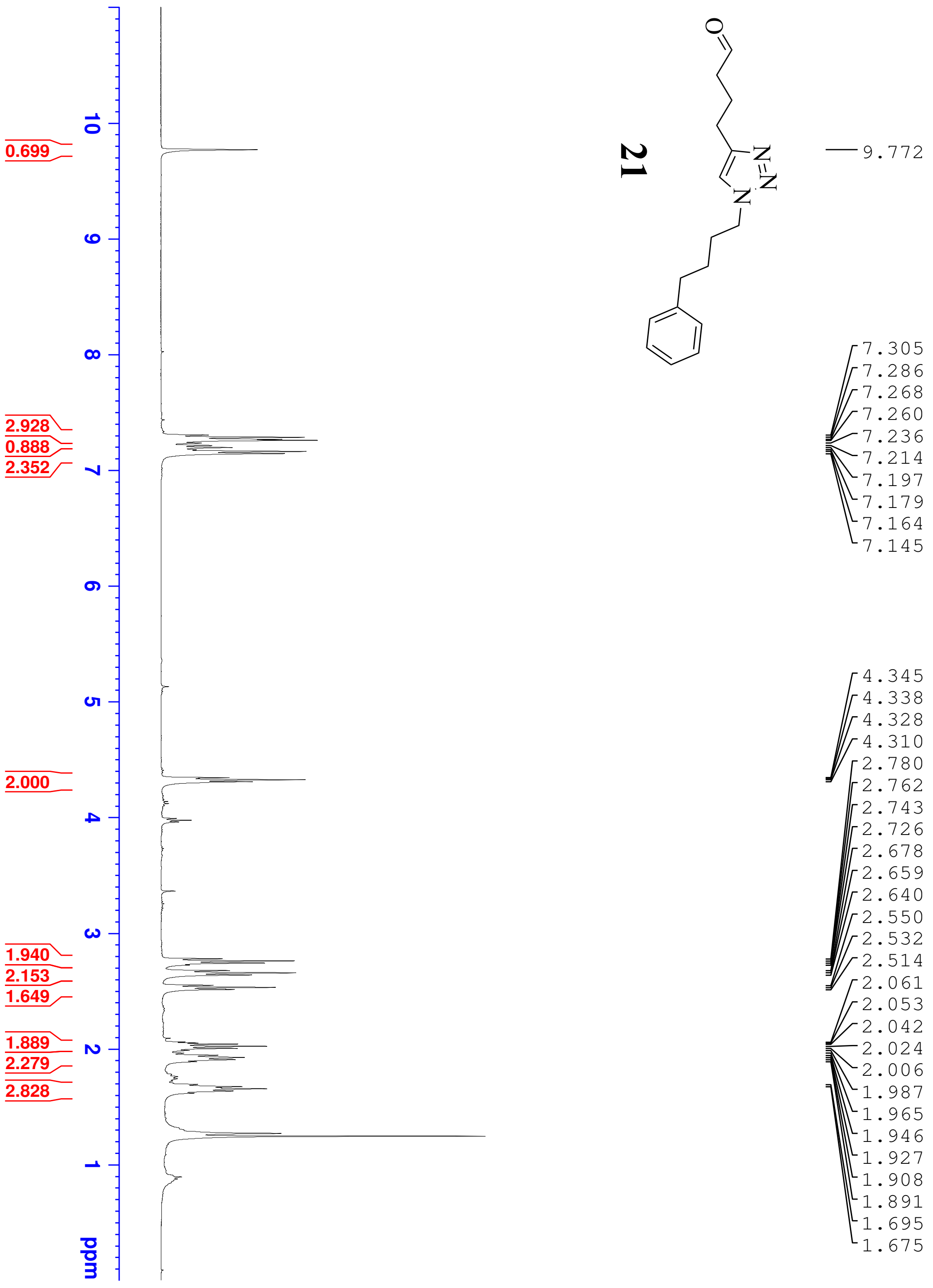
21
9.772
7.305
7.286
7.268
7.260
7.236
7.214
7.197
7.179
7.164
7.145
4.345
4.338
4.328
4.310
2.780
2.762
2.743
2.726
2.678
2.659
2.640
2.550
2.532
2.514
2.061
2.053
2.042
2.024
2.006
1.987
1.965
1.946
1.927
1.908
1.891
1.695
1.675
0.699
2.928
0.888
2.352
2.000
1.940
2.153
1.649
1.889
2.279
2.828
ppm

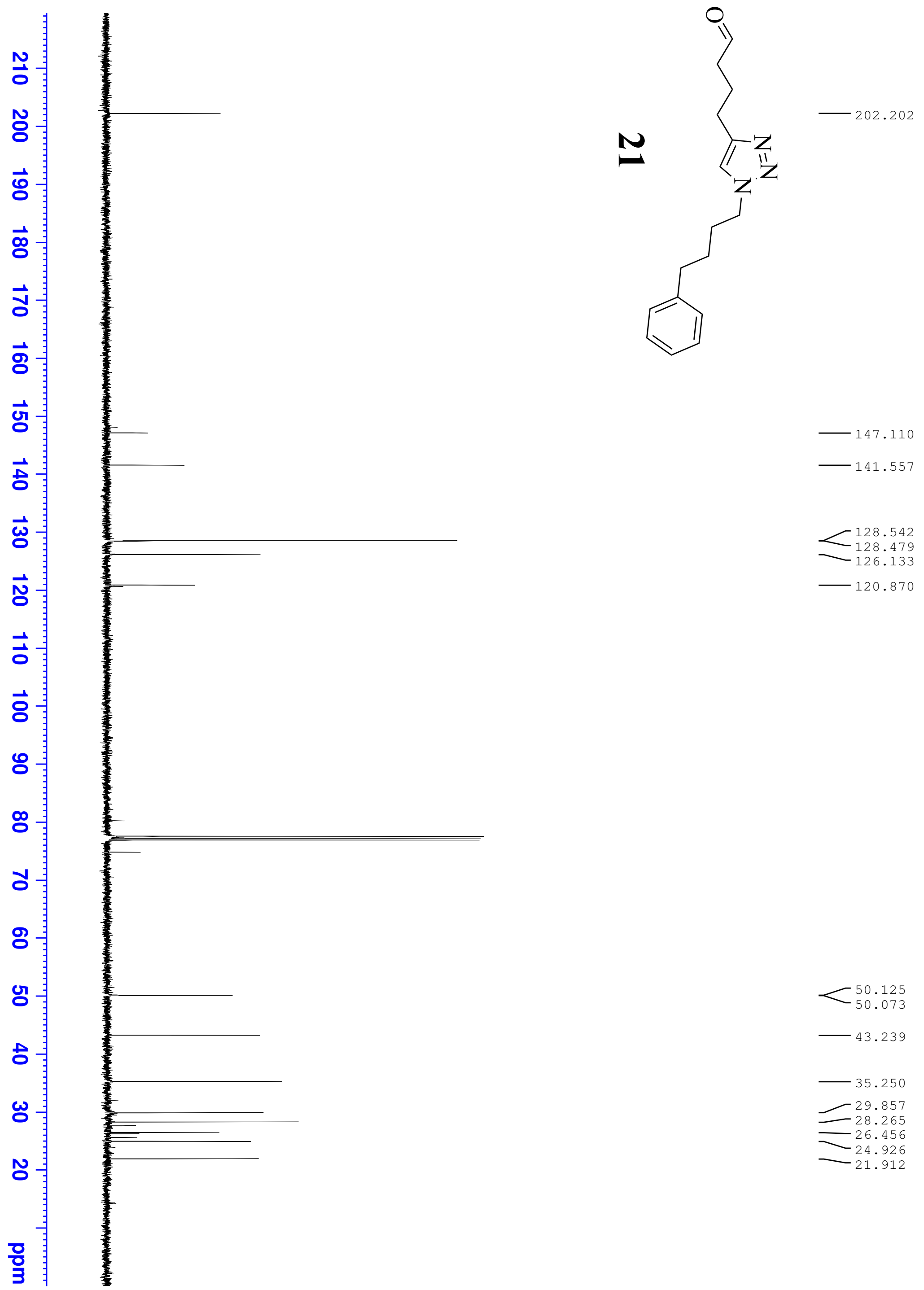
21
O
N=N
N
202.202
147.110
141.557
128.542
128.479
126.133
120.870
50.125
50.073
43.239
35.250
29.857
28.265
26.456
24.926
21.912
ppm

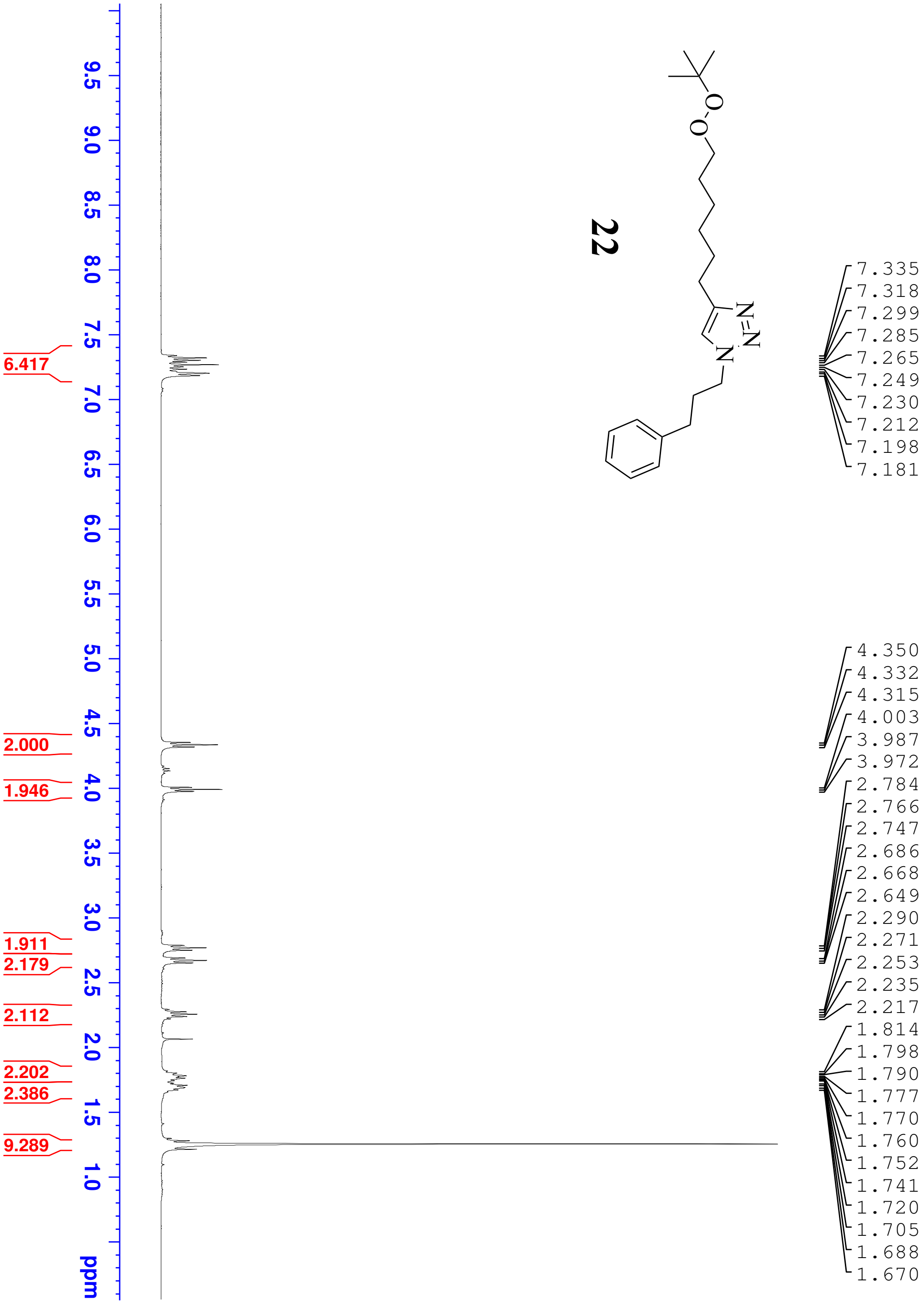
22
9.5
9.0
8.5
8.0
7.5
7.0
6.5
6.0
5.5
5.0
4.5
4.0
3.5
3.0
2.5
2.0
1.5
1.0
ppm
6.417
2.000
1.946
1.911
2.179
2.112
2.202
2.386
9.289
7.335
7.318
7.299
7.285
7.265
7.249
7.230
7.212
7.198
7.181
4.350
4.332
4.315
4.003
3.987
3.972
2.784
2.766
2.747
2.686
2.668
2.649
2.290
2.271
2.253
2.235
2.217
1.814
1.798
1.790
1.777
1.770
1.760
1.752
1.741
1.720
1.705
1.688
1.670

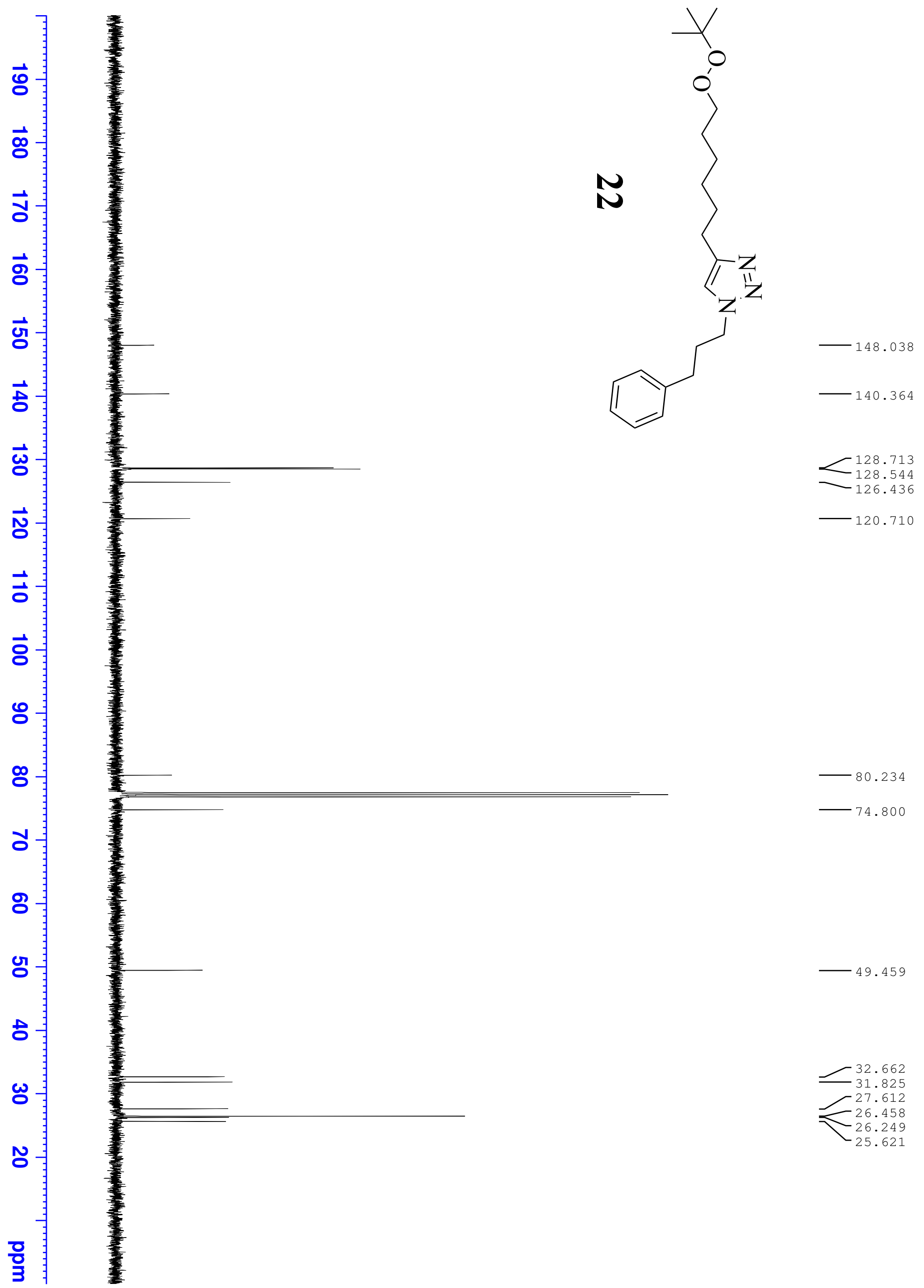
22
148.038
140.364
128.713
128.544
126.436
120.710
80.234
74.800
49.459
32.662
31.825
27.612
26.458
26.249
25.621
ppm

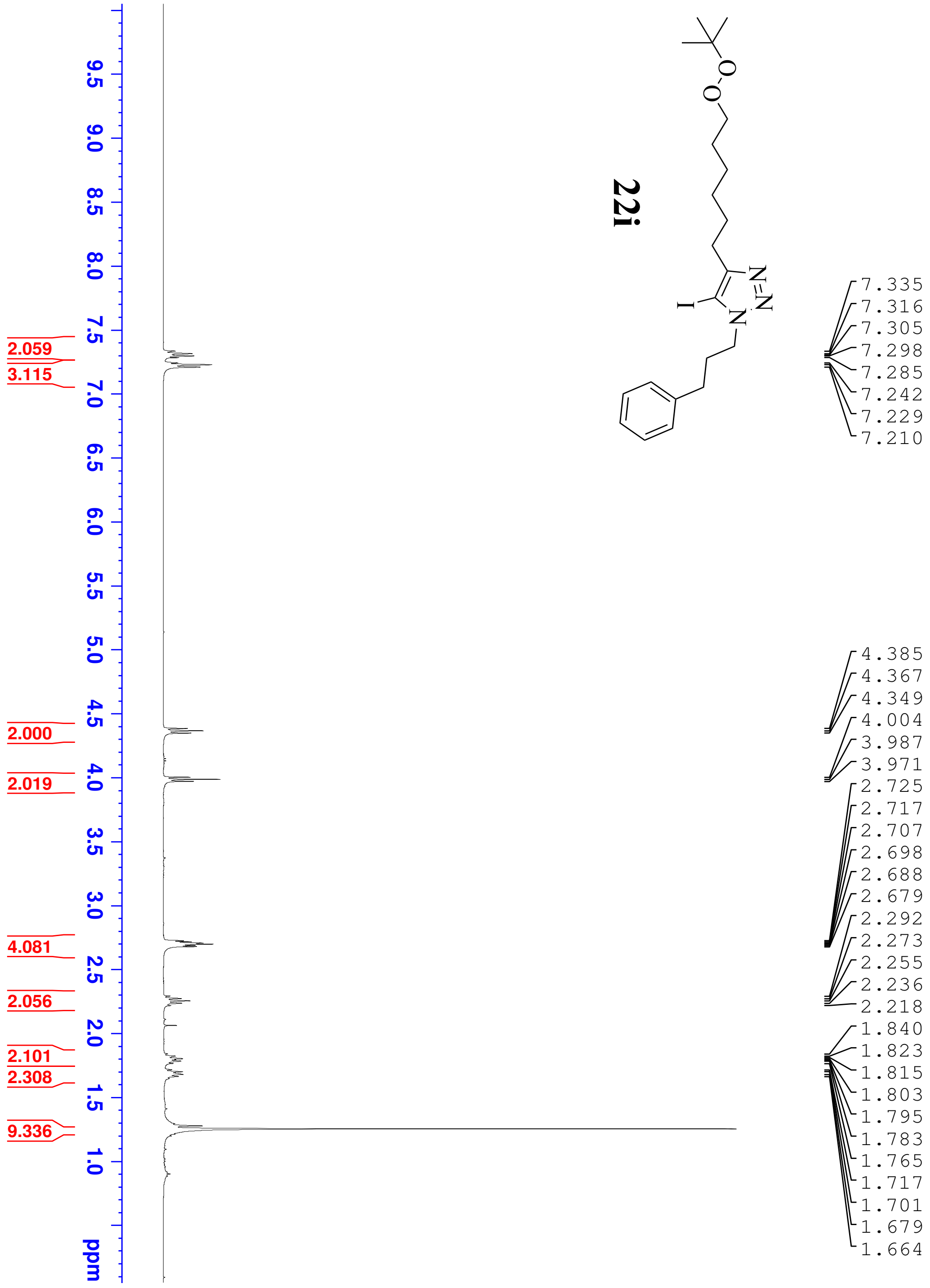

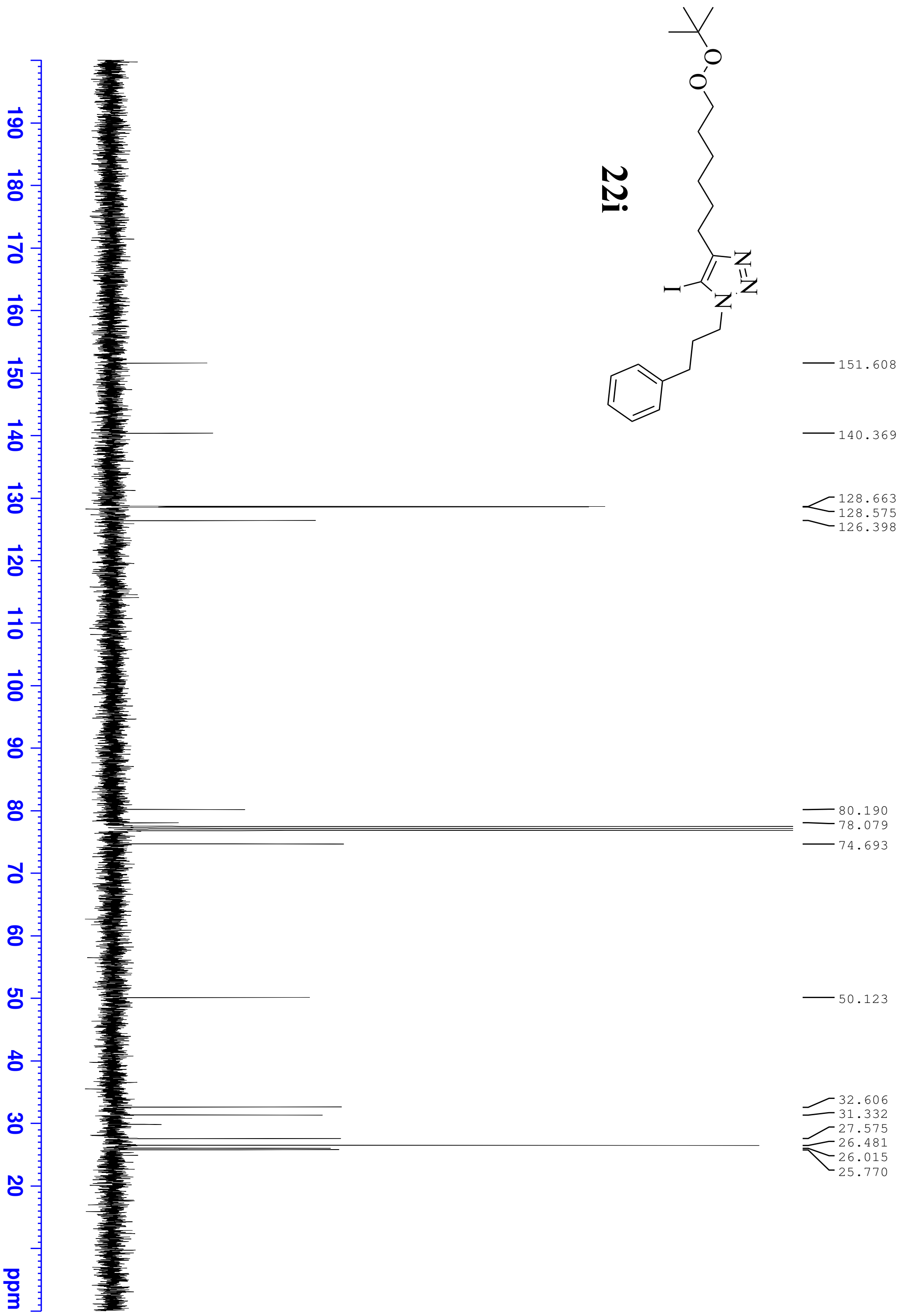
22i
I
N
N=N
O
O
151.608
140.369
128.663
128.575
126.398
80.190
78.079
74.693
50.123
32.606
31.332
27.575
26.481
26.015
25.770
190
180
170
160
150
140
130
120
110
100
90
80
70
60
50
40
30
20
ppm

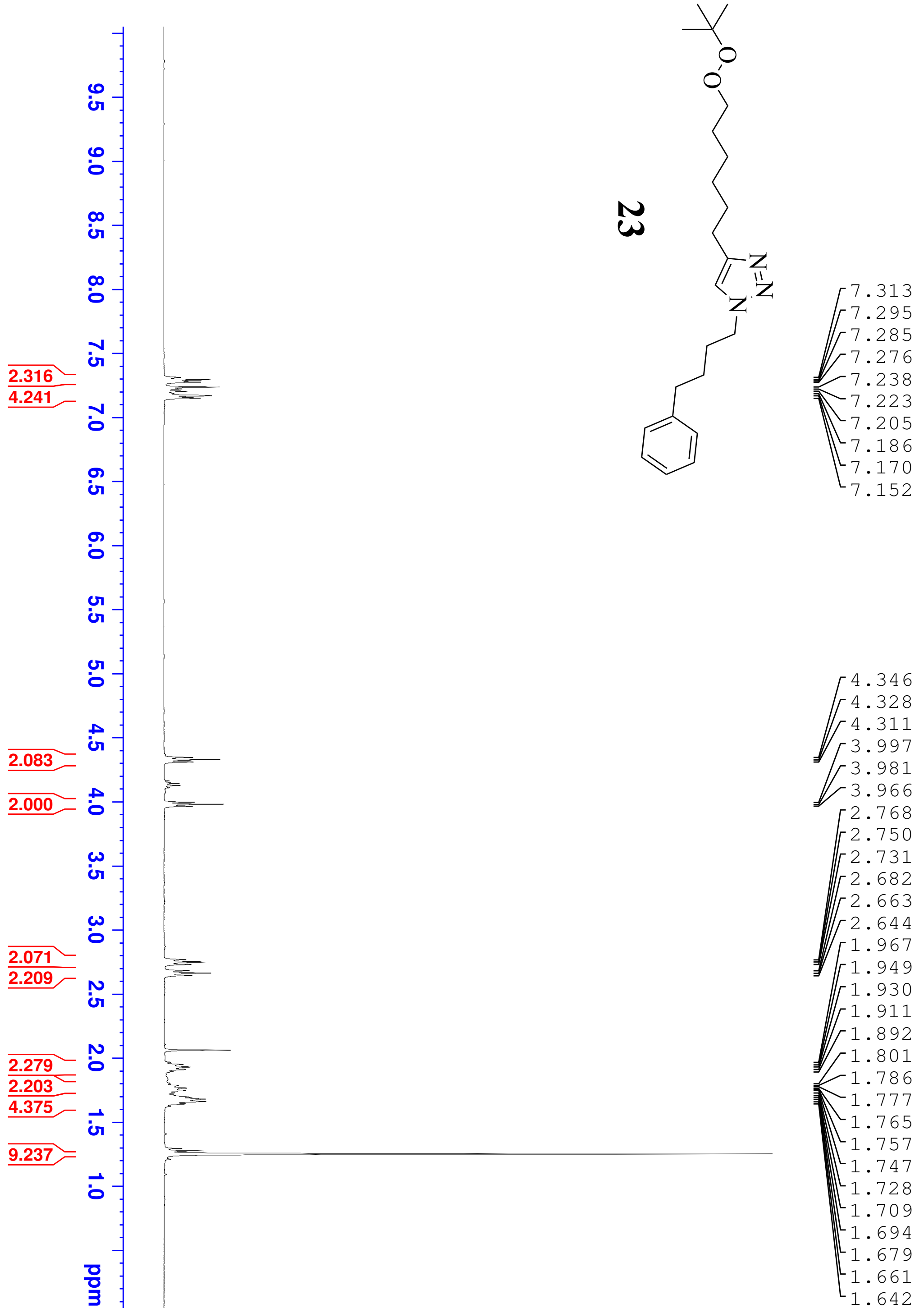
23
N=N
N
O
O
7.313
7.295
7.285
7.276
7.238
7.223
7.205
7.186
7.170
7.152
4.346
4.328
4.311
3.997
3.981
3.966
2.768
2.750
2.731
2.682
2.663
2.644
1.967
1.949
1.930
1.911
1.892
1.801
1.786
1.777
1.765
1.757
1.747
1.728
1.709
1.694
1.679
1.661
1.642
2.316
4.241
2.083
2.000
2.071
2.209
2.279
2.203
4.375
9.237
9.5
9.0
8.5
8.0
7.5
7.0
6.5
6.0
5.5
5.0
4.5
4.0
3.5
3.0
2.5
2.0
1.5
1.0
ppm

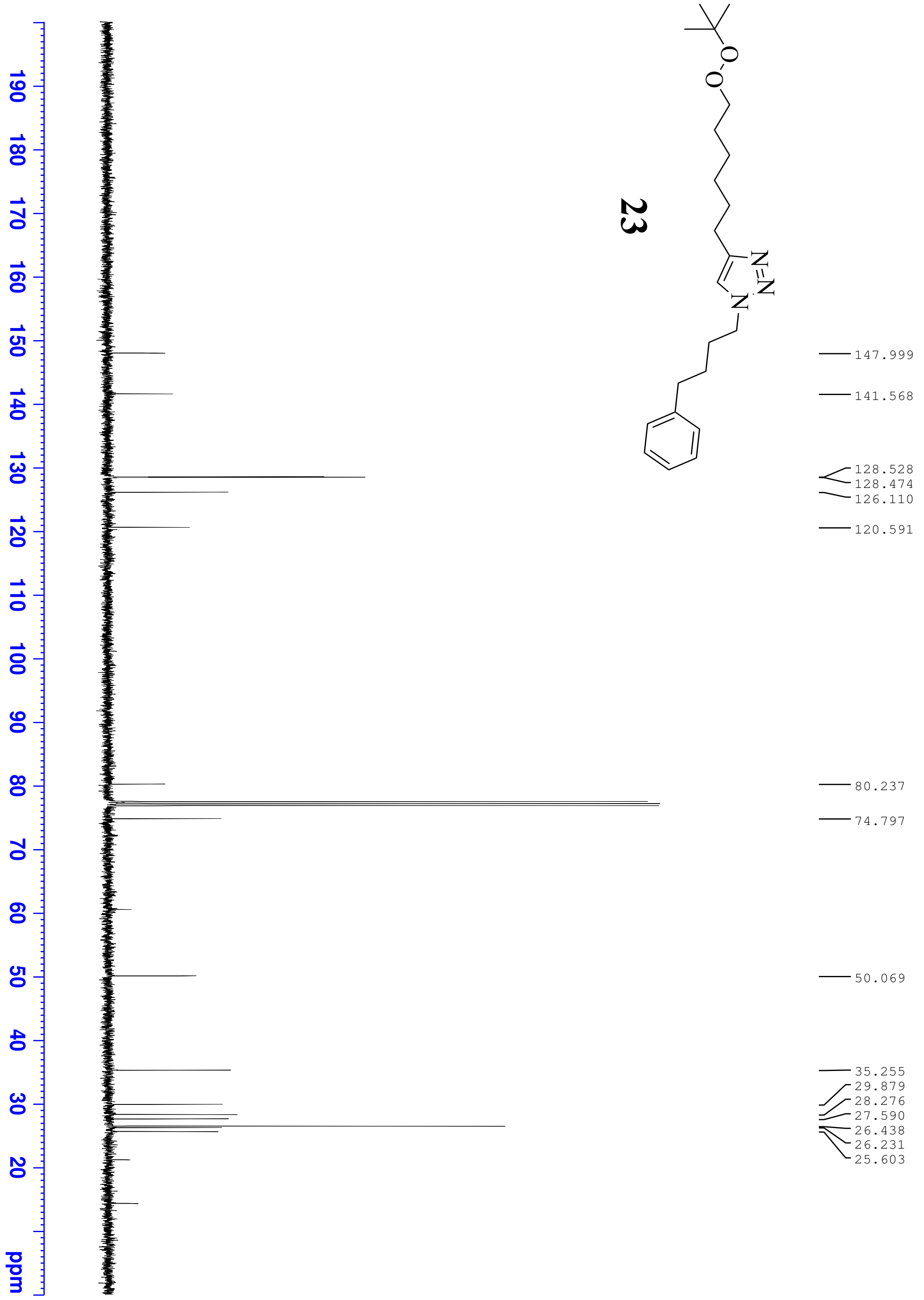
23
147.999
141.568
128.528
128.474
126.110
120.591
80.237
74.797
50.069
35.255
29.879
28.276
27.590
26.438
26.231
25.603
190 180 170 160 150 140 130 120 110 100 90 80 70 60 50 40 30 20
ppm

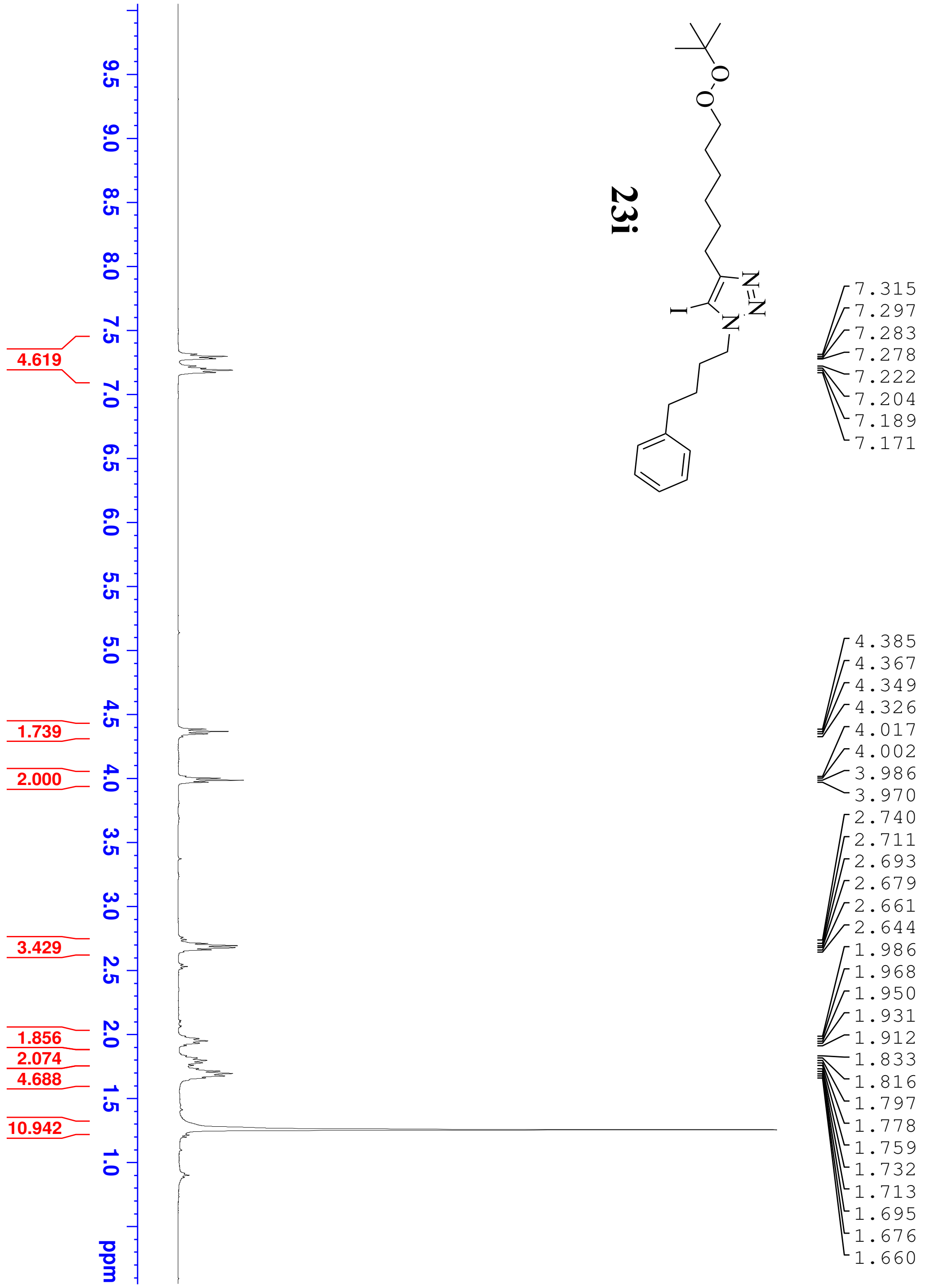
23i
7.315
7.297
7.283
7.278
7.222
7.204
7.189
7.171
4.385
4.367
4.349
4.326
4.017
4.002
3.986
3.970
2.740
2.711
2.693
2.679
2.661
2.644
1.986
1.968
1.950
1.931
1.912
1.833
1.816
1.797
1.778
1.759
1.732
1.713
1.695
1.676
1.660
4.619
1.739
2.000
3.429
1.856
2.074
4.688
10.942
ppm

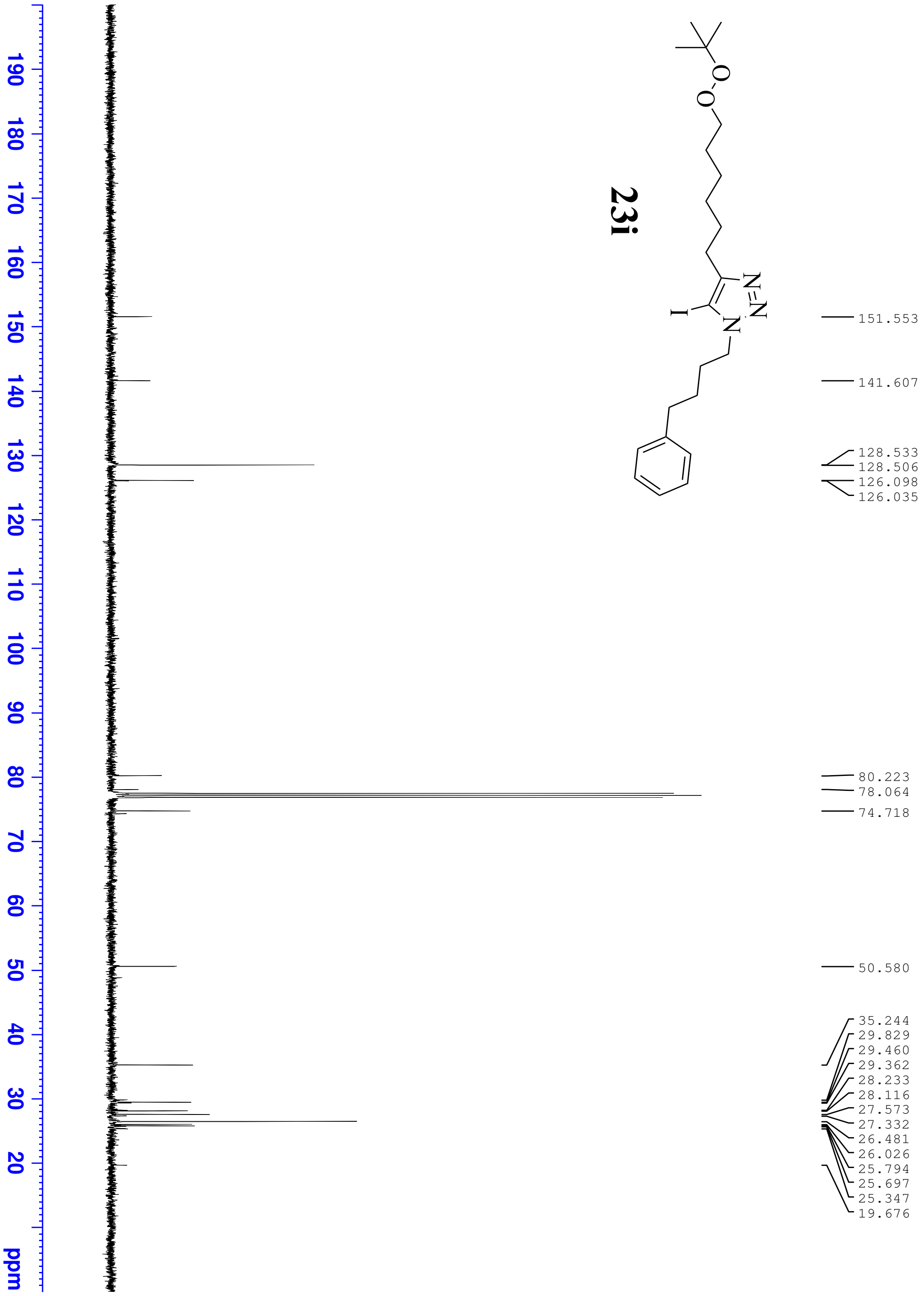
23i
151.553
141.607
128.533
128.506
126.098
126.035
80.223
78.064
74.718
50.580
35.244
29.829
29.460
29.362
28.233
28.116
27.573
27.332
26.481
26.026
25.794
25.697
25.347
19.676
ppm

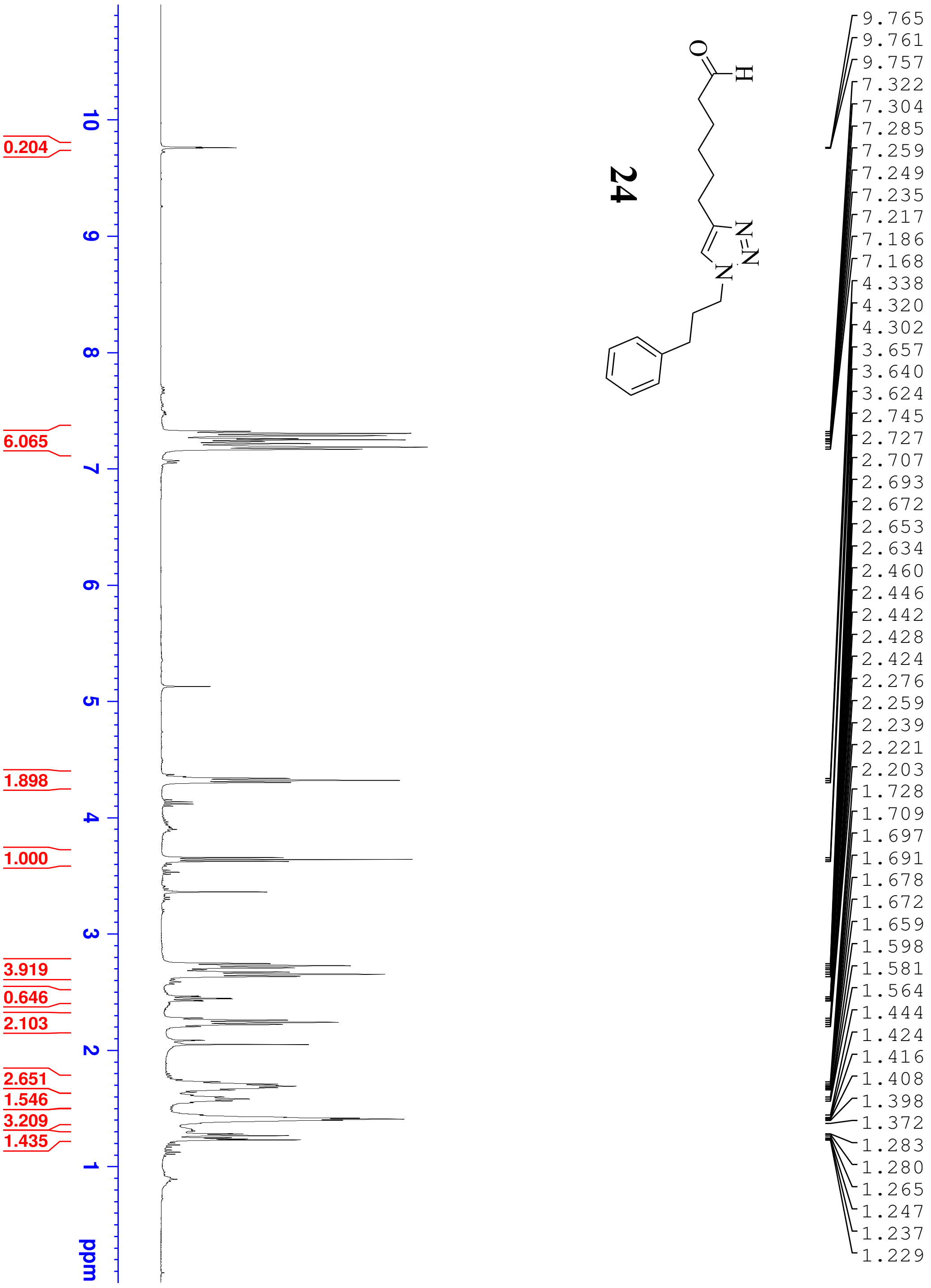
24
ppm
10
9
8
7
6
5
4
3
2
1
0.204
6.065
1.898
1.000
3.919
0.646
2.103
2.651
1.546
3.209
1.435
9.765
9.761
9.757
7.322
7.304
7.285
7.259
7.249
7.235
7.217
7.186
7.168
4.338
4.320
4.302
3.657
3.640
3.624
2.745
2.727
2.707
2.693
2.672
2.653
2.634
2.460
2.446
2.442
2.428
2.424
2.276
2.259
2.239
2.221
2.203
1.728
1.709
1.697
1.691
1.678
1.672
1.659
1.598
1.581
1.564
1.444
1.424
1.416
1.408
1.398
1.372
1.283
1.280
1.265
1.247
1.237
1.229

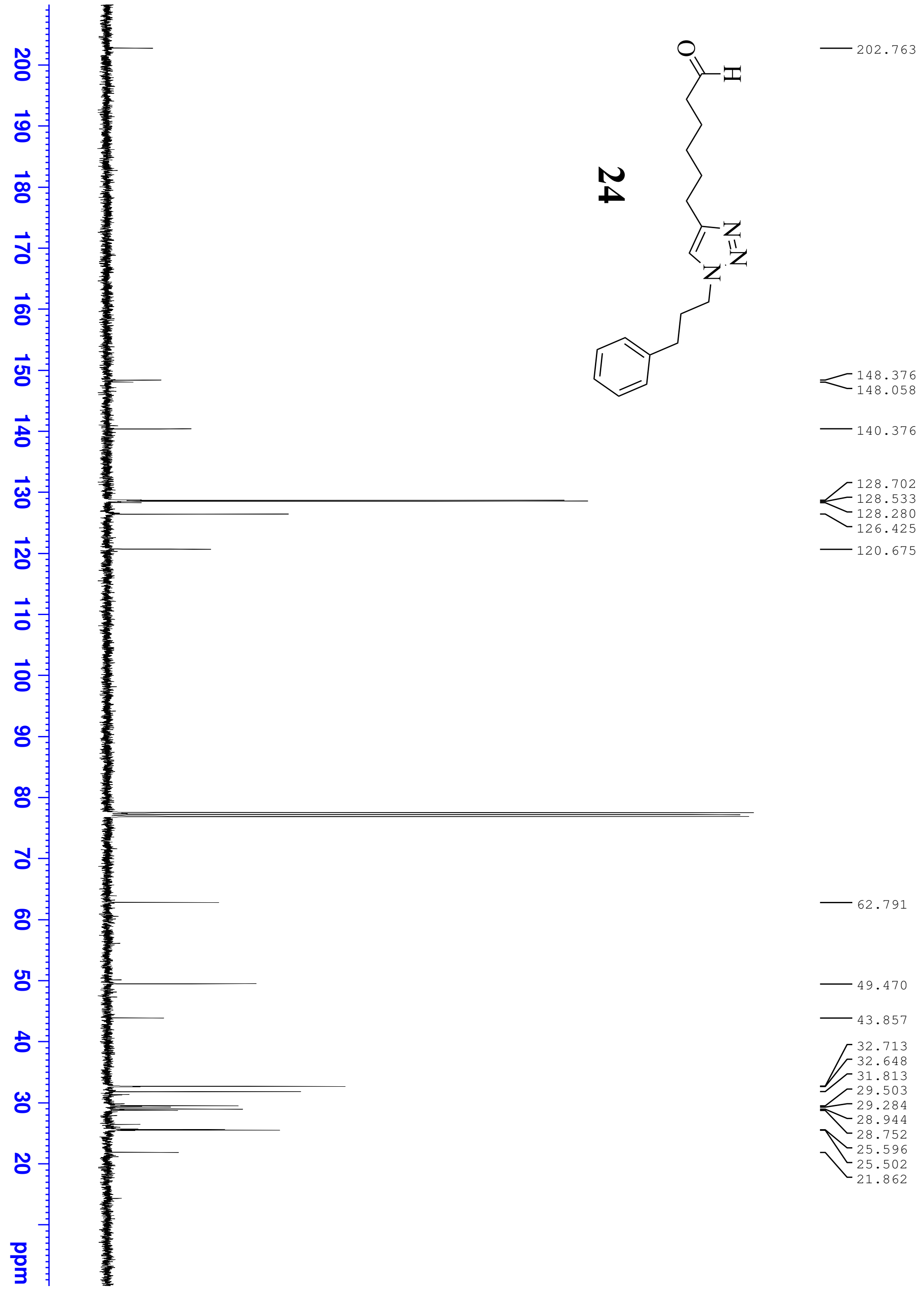
24
202.763
148.376
148.058
140.376
128.702
128.533
128.280
126.425
120.675
62.791
49.470
43.857
32.713
32.648
31.813
29.503
29.284
28.944
28.752
25.596
25.502
21.862
ppm

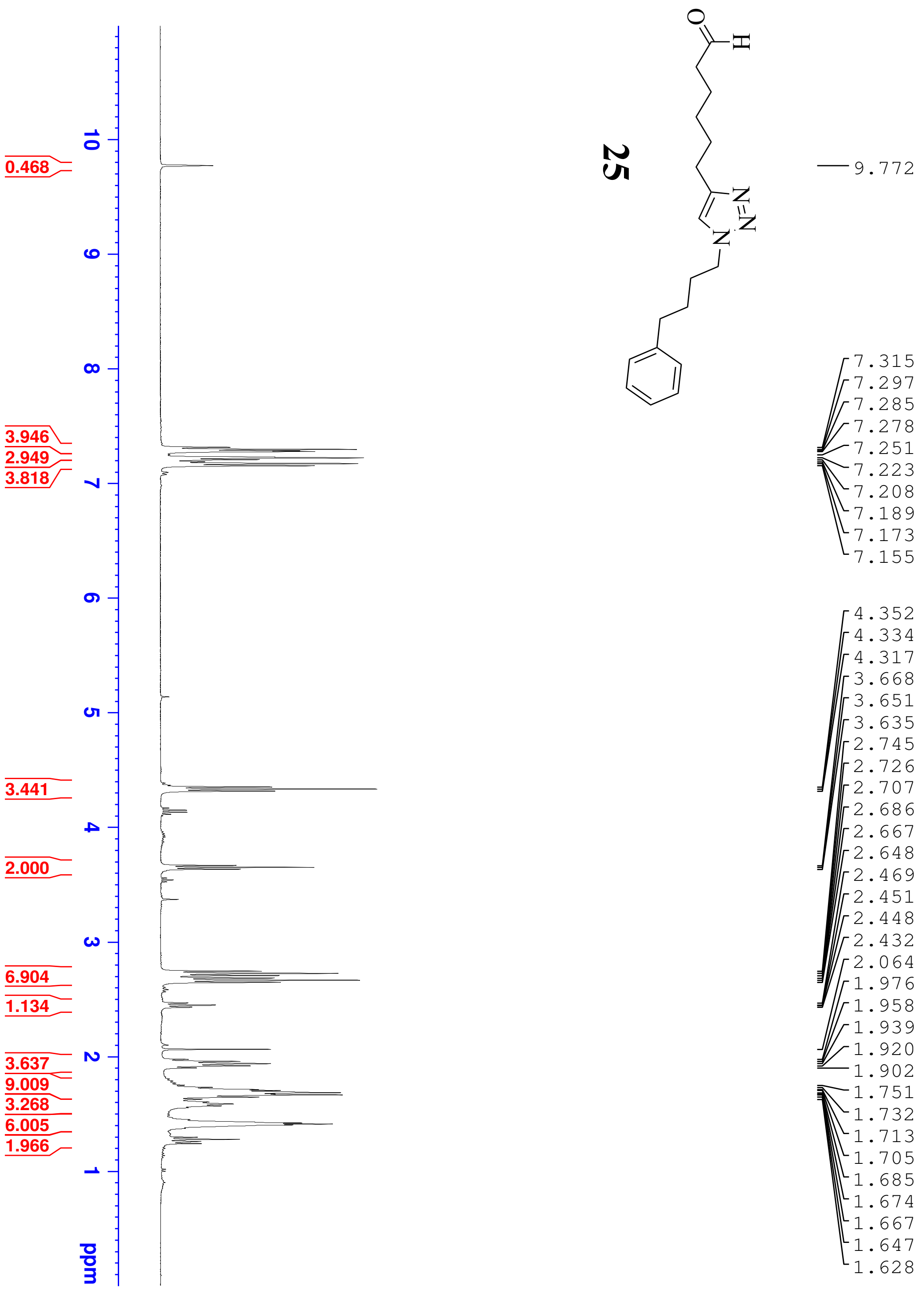
25
9.772
7.315
7.297
7.285
7.278
7.251
7.223
7.208
7.189
7.173
7.155
4.352
4.334
4.317
3.668
3.651
3.635
2.745
2.726
2.707
2.686
2.667
2.648
2.469
2.451
2.448
2.432
2.064
1.976
1.958
1.939
1.920
1.902
1.751
1.732
1.713
1.705
1.685
1.674
1.667
1.647
1.628
0.468
3.946
2.949
3.818
3.441
2.000
6.904
1.134
3.637
9.009
3.268
6.005
1.966
ppm

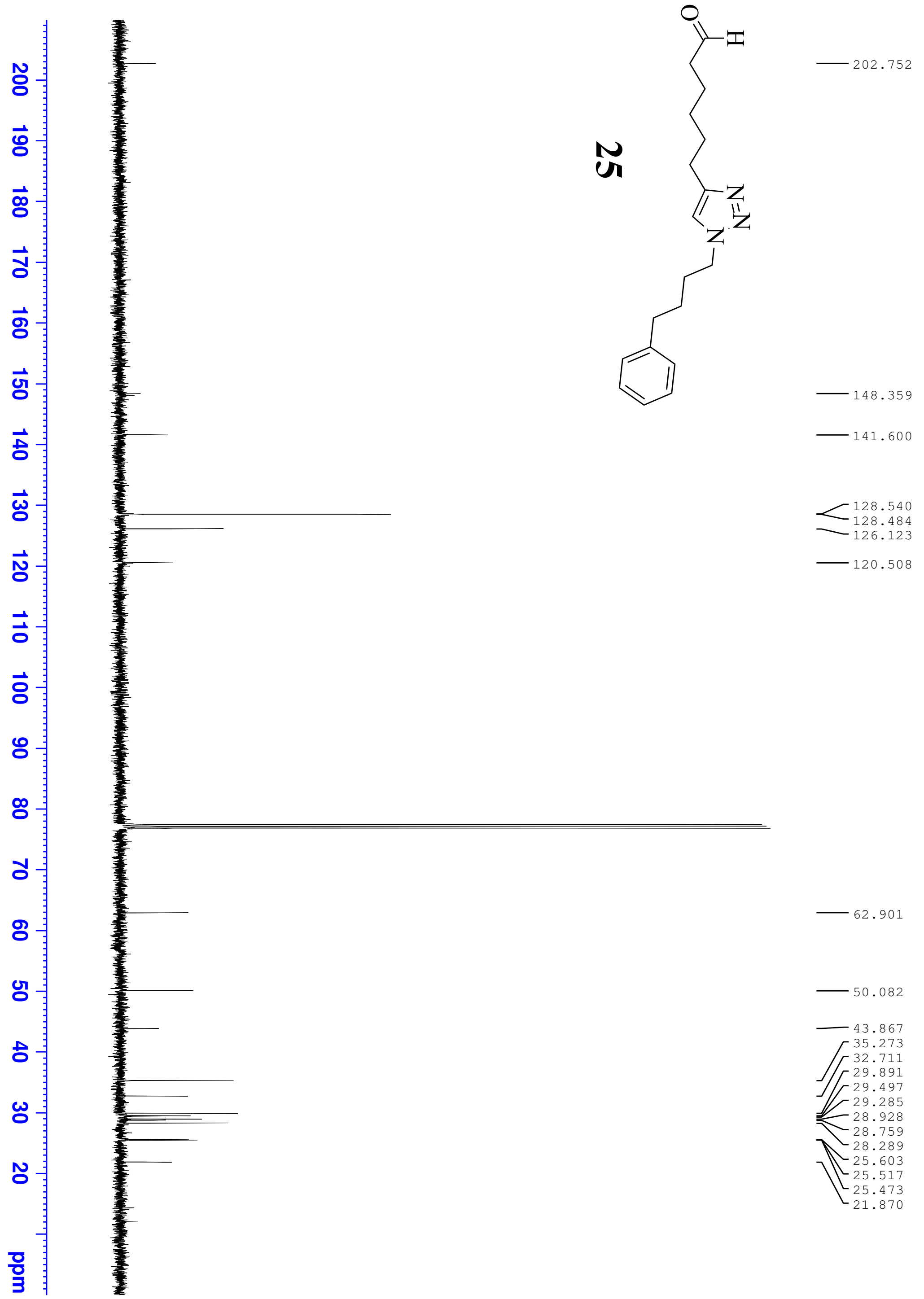
25
202.752
148.359
141.600
128.540
128.484
126.123
120.508
62.901
50.082
43.867
35.273
32.711
29.891
29.497
29.285
28.928
28.759
28.289
25.603
25.517
25.473
21.870
ppm

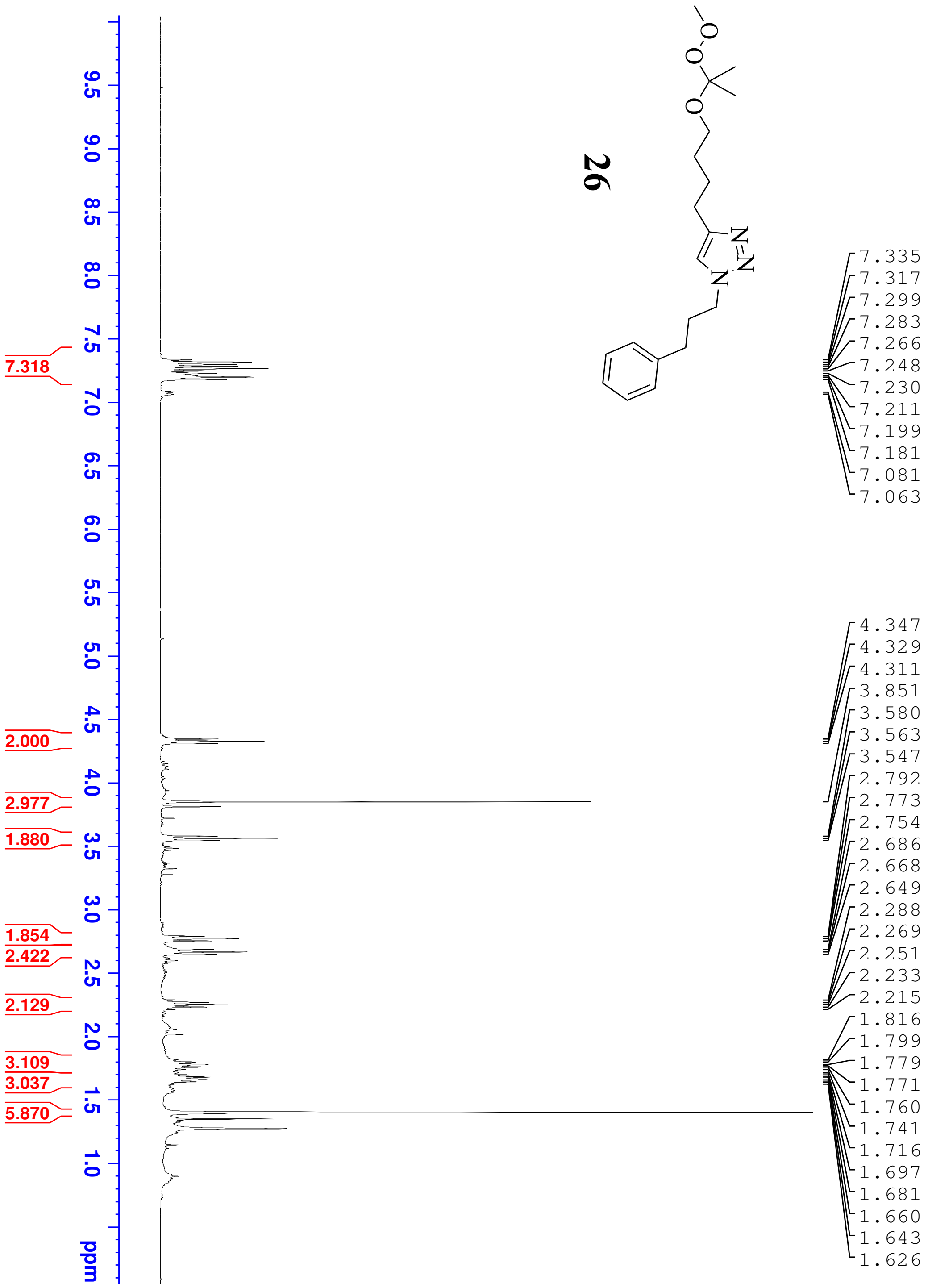

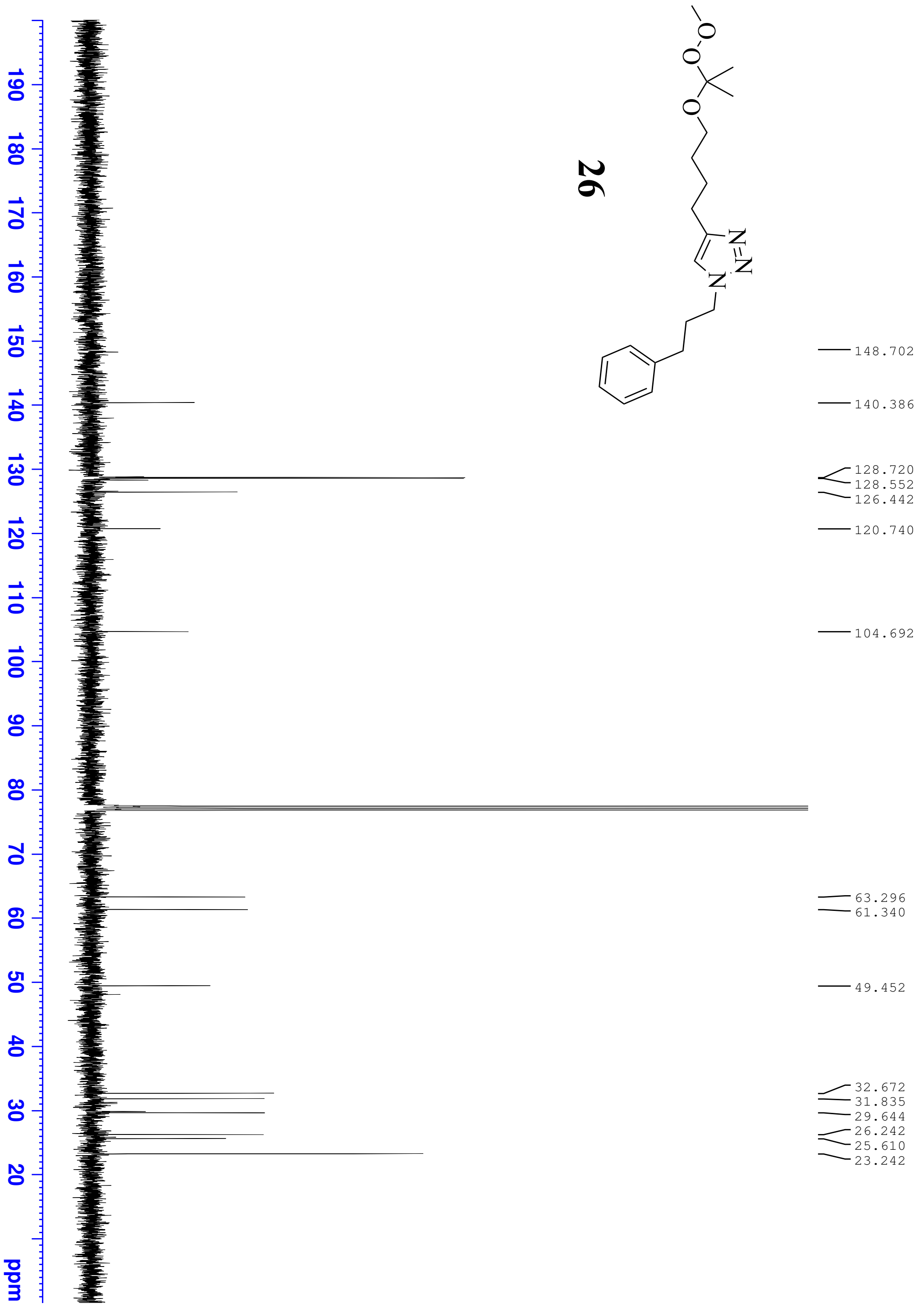
26
148.702
140.386
128.720
128.552
126.442
120.740
104.692
63.296
61.340
49.452
32.672
31.835
29.644
26.242
25.610
23.242
ppm

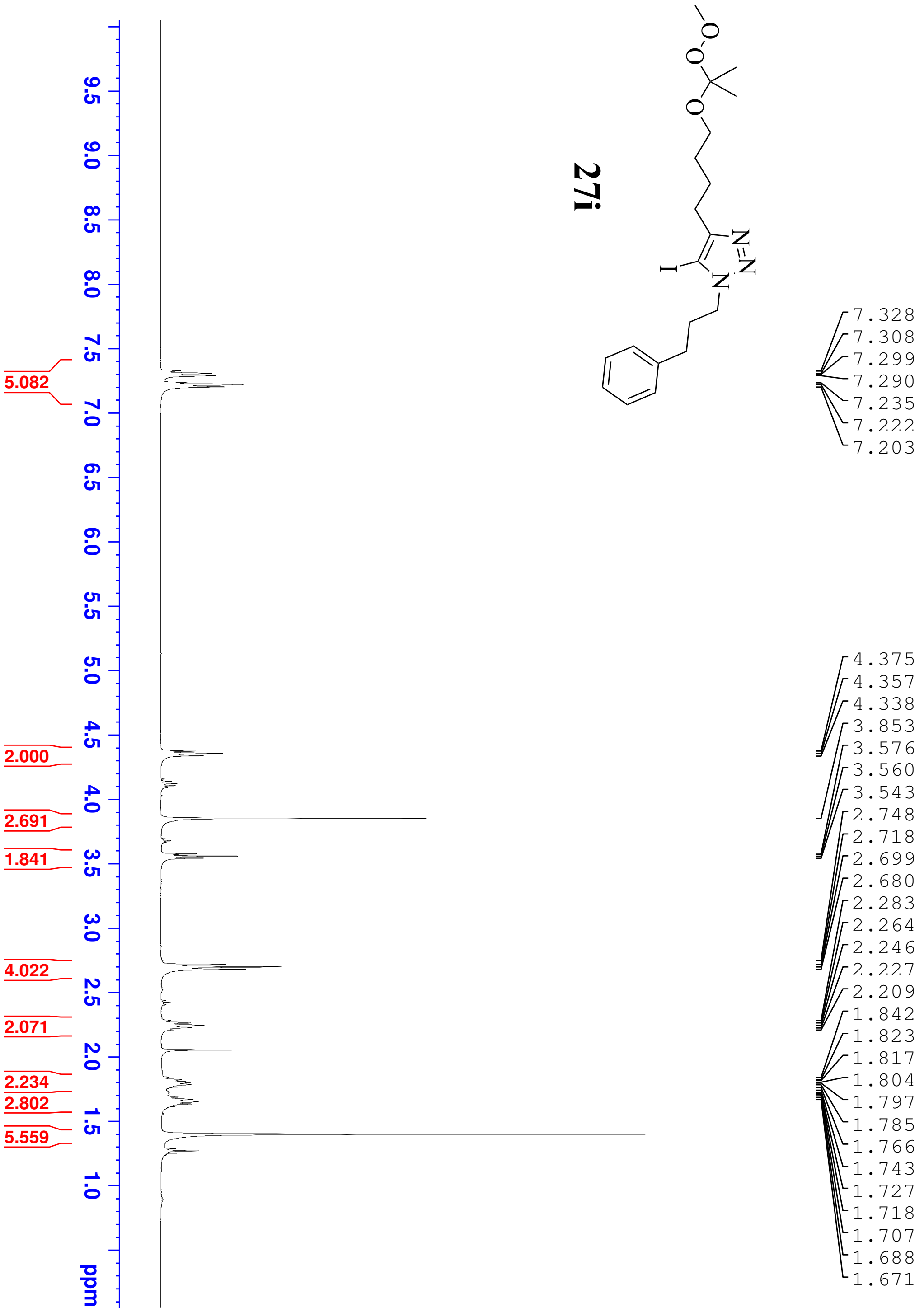
27i
I
N
N=N
O
O-O
7.328
7.308
7.299
7.290
7.235
7.222
7.203
4.375
4.357
4.338
3.853
3.576
3.560
3.543
2.748
2.718
2.699
2.680
2.283
2.264
2.246
2.227
2.209
1.842
1.823
1.817
1.804
1.797
1.785
1.766
1.743
1.727
1.718
1.707
1.688
1.671
5.082
2.000
2.691
1.841
4.022
2.071
2.234
2.802
5.559
9.5
9.0
8.5
8.0
7.5
7.0
6.5
6.0
5.5
5.0
4.5
4.0
3.5
3.0
2.5
2.0
1.5
1.0
ppm

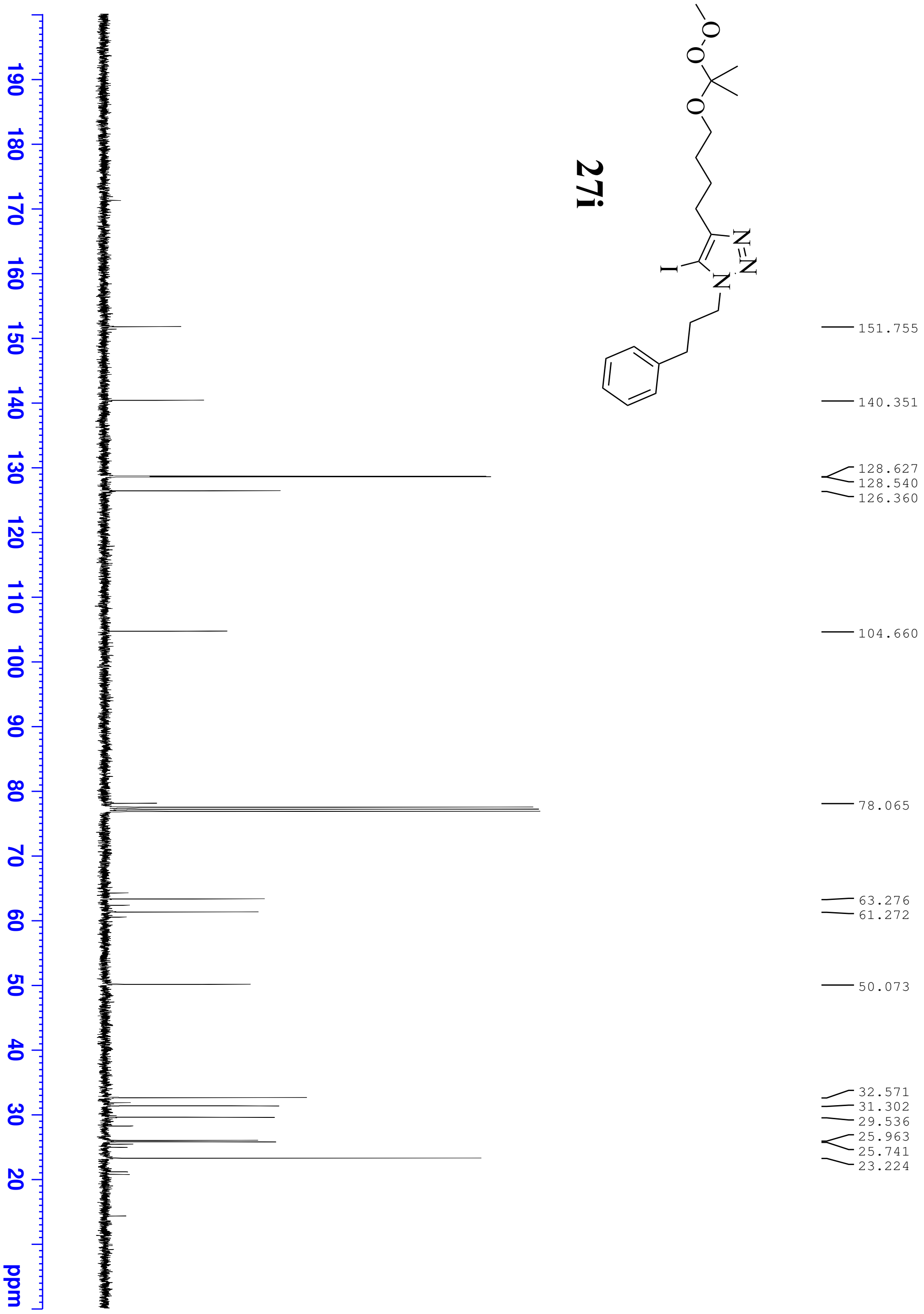
27i
I
N
N=N
O
O
O
151.755
140.351
128.627
128.540
126.360
104.660
78.065
63.276
61.272
50.073
32.571
31.302
29.536
25.963
25.741
23.224
190
180
170
160
150
140
130
120
110
100
90
80
70
60
50
40
30
20
ppm

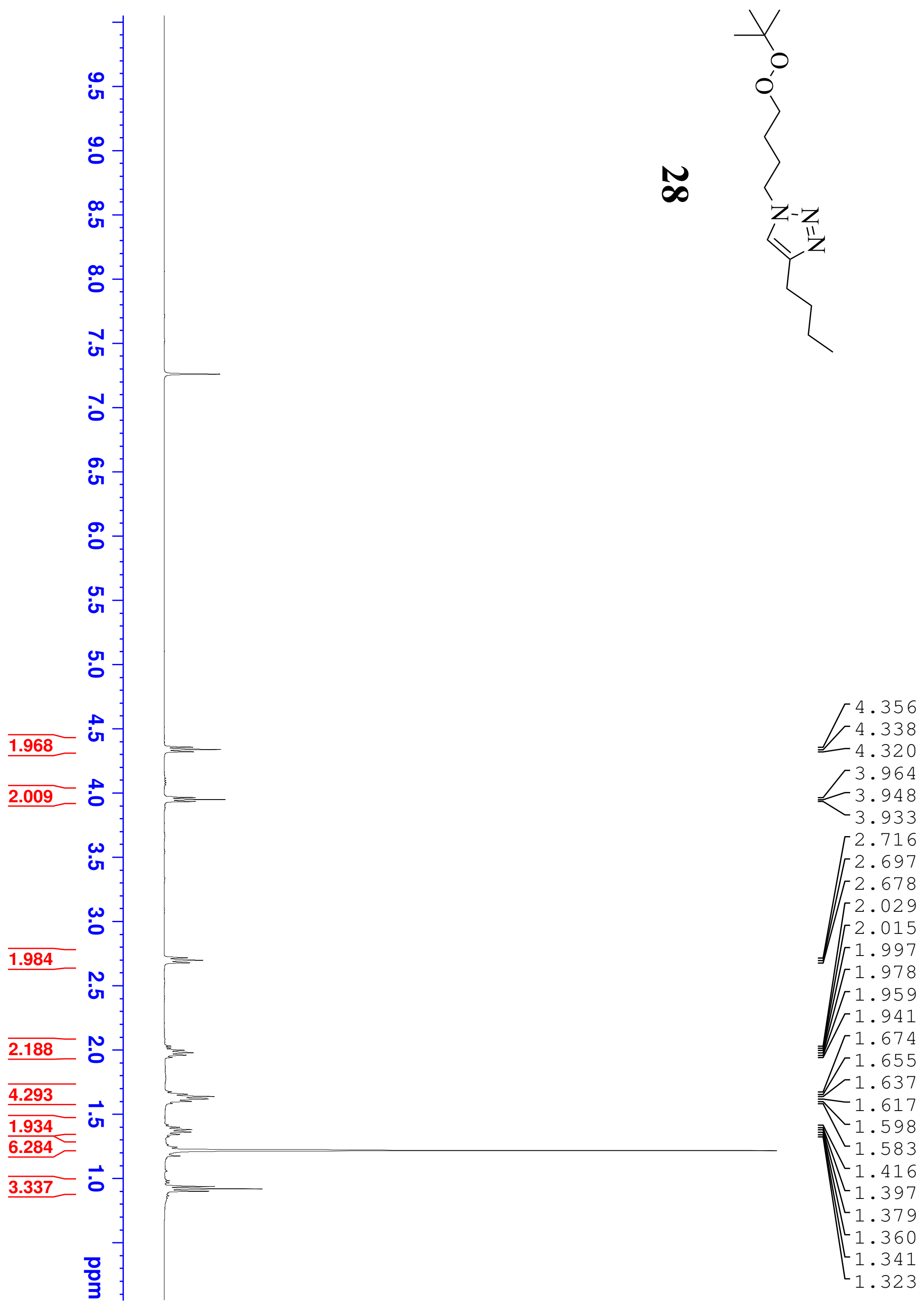
28
N=N
N
O-O
ppm
9.5
9.0
8.5
8.0
7.5
7.0
6.5
6.0
5.5
5.0
4.5
4.0
3.5
3.0
2.5
2.0
1.5
1.0
1.968
2.009
1.984
2.188
4.293
1.934
6.284
3.337
4.356
4.338
4.320
3.964
3.948
3.933
2.716
2.697
2.678
2.029
2.015
1.997
1.978
1.959
1.941
1.674
1.655
1.637
1.617
1.598
1.583
1.416
1.397
1.379
1.360
1.341
1.323

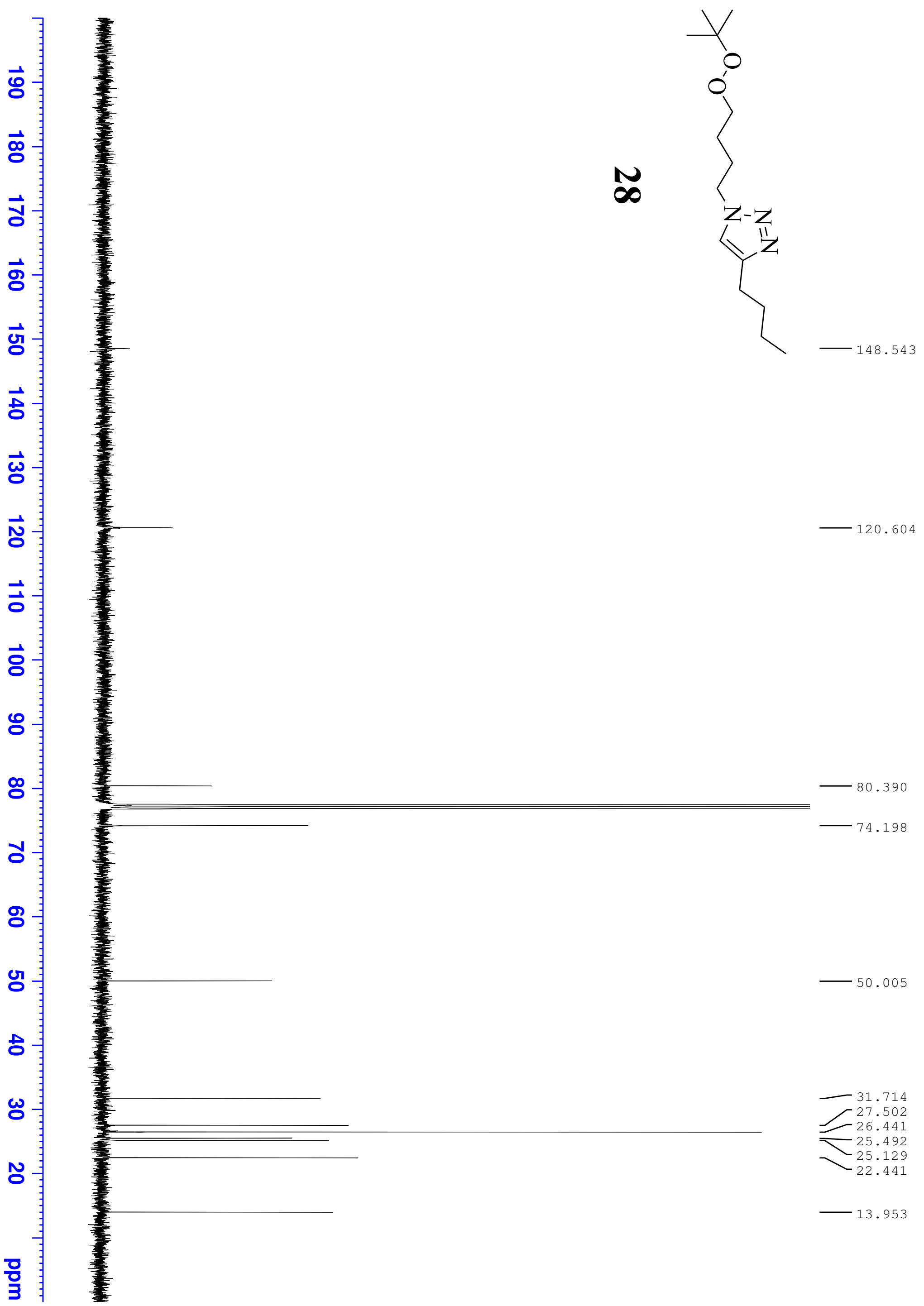
28
148.543
120.604
80.390
74.198
50.005
31.714
27.502
26.441
25.492
25.129
22.441
13.953
ppm
190
180
170
160
150
140
130
120
110
100
90
80
70
60
50
40
30
20

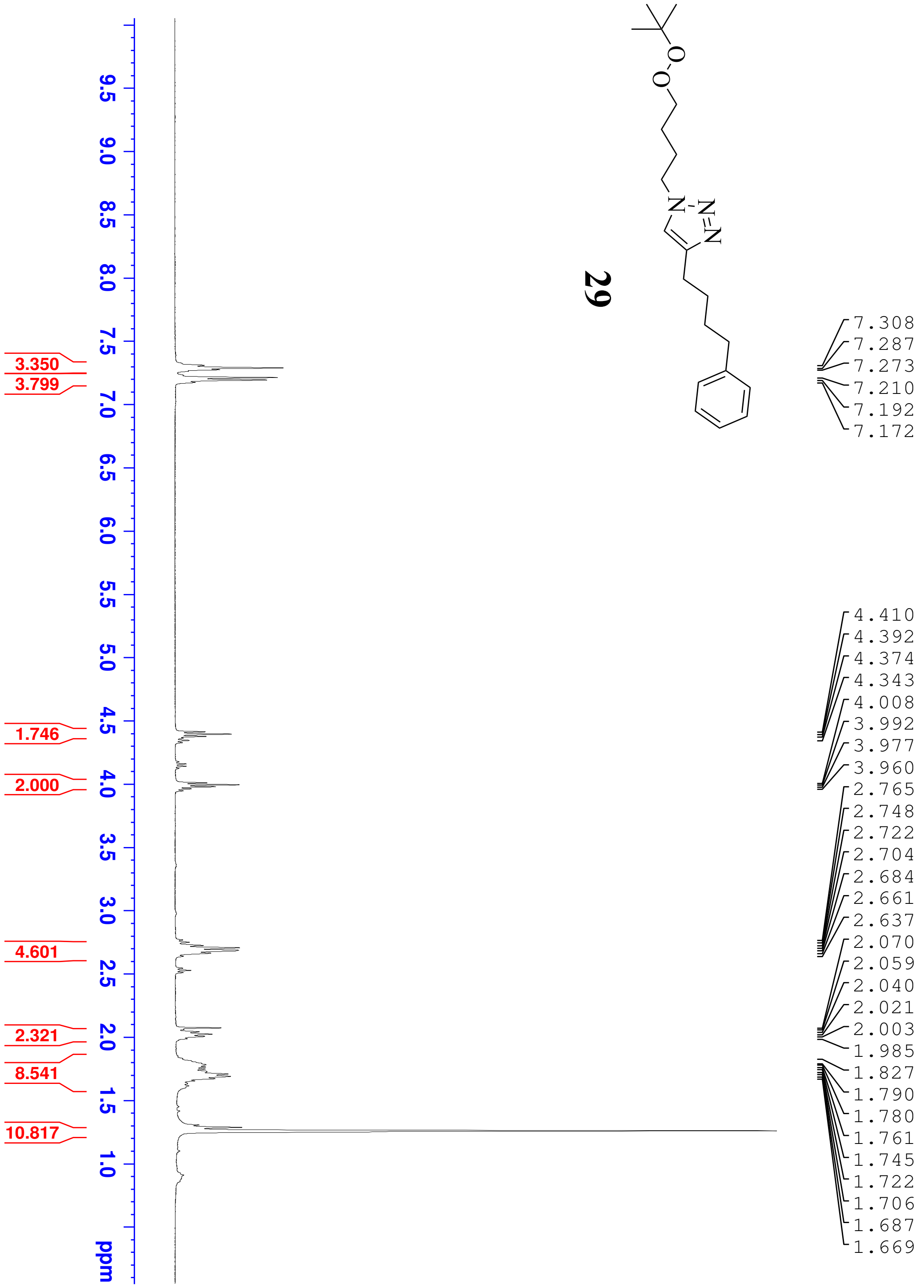
29
7.308
7.287
7.273
7.210
7.192
7.172
4.410
4.392
4.374
4.343
4.008
3.992
3.977
3.960
2.765
2.748
2.722
2.704
2.684
2.661
2.637
2.070
2.059
2.040
2.021
2.003
1.985
1.827
1.790
1.780
1.761
1.745
1.722
1.706
1.687
1.669
3.350
3.799
1.746
2.000
4.601
2.321
8.541
10.817
9.5
9.0
8.5
8.0
7.5
7.0
6.5
6.0
5.5
5.0
4.5
4.0
3.5
3.0
2.5
2.0
1.5
1.0
ppm

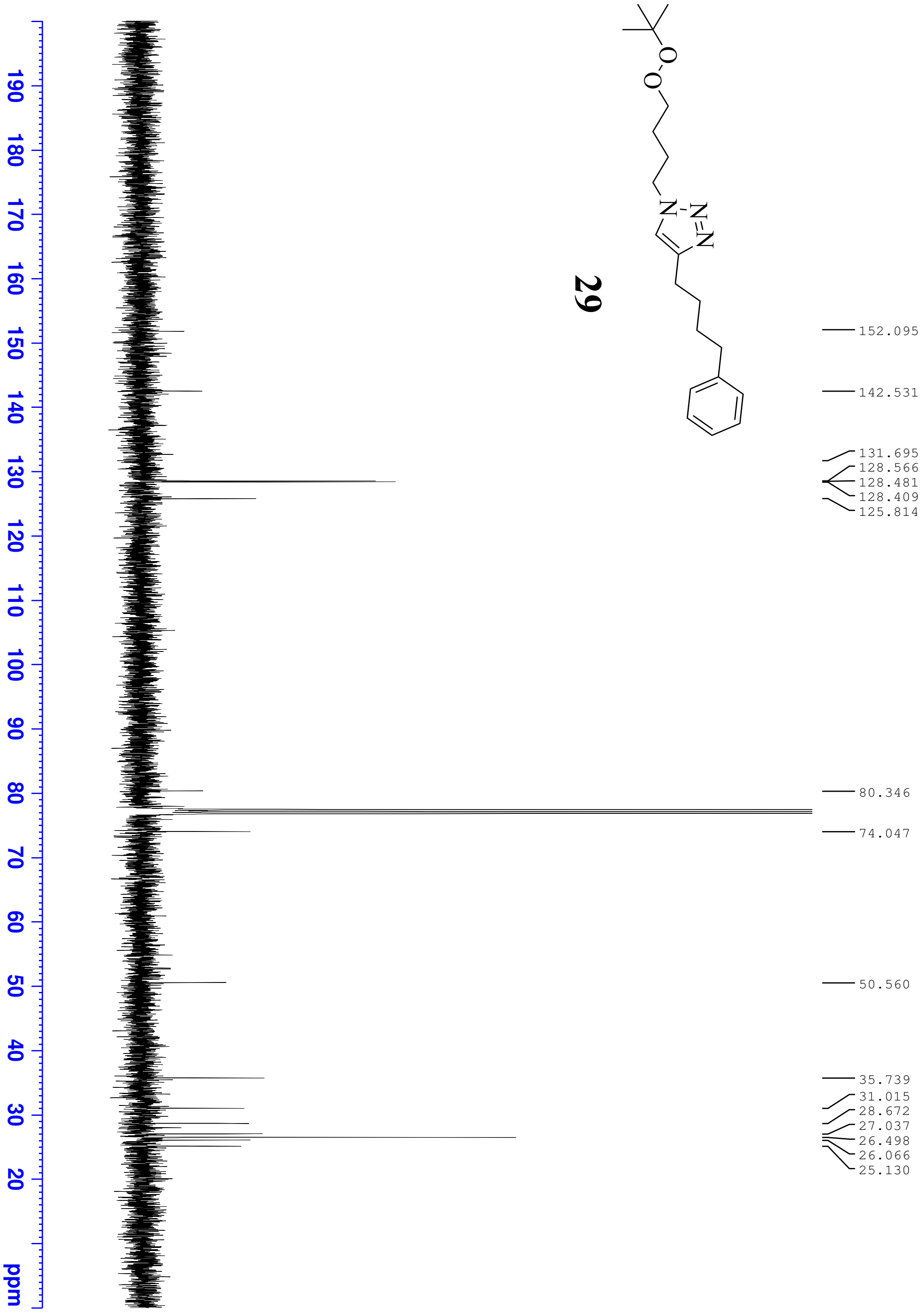
29
152.095
142.531
131.695
128.566
128.481
128.409
125.814
80.346
74.047
50.560
35.739
31.015
28.672
27.037
26.498
26.066
25.130
ppm

Figure 1: Pentanethiolate Functionalized Au NPs

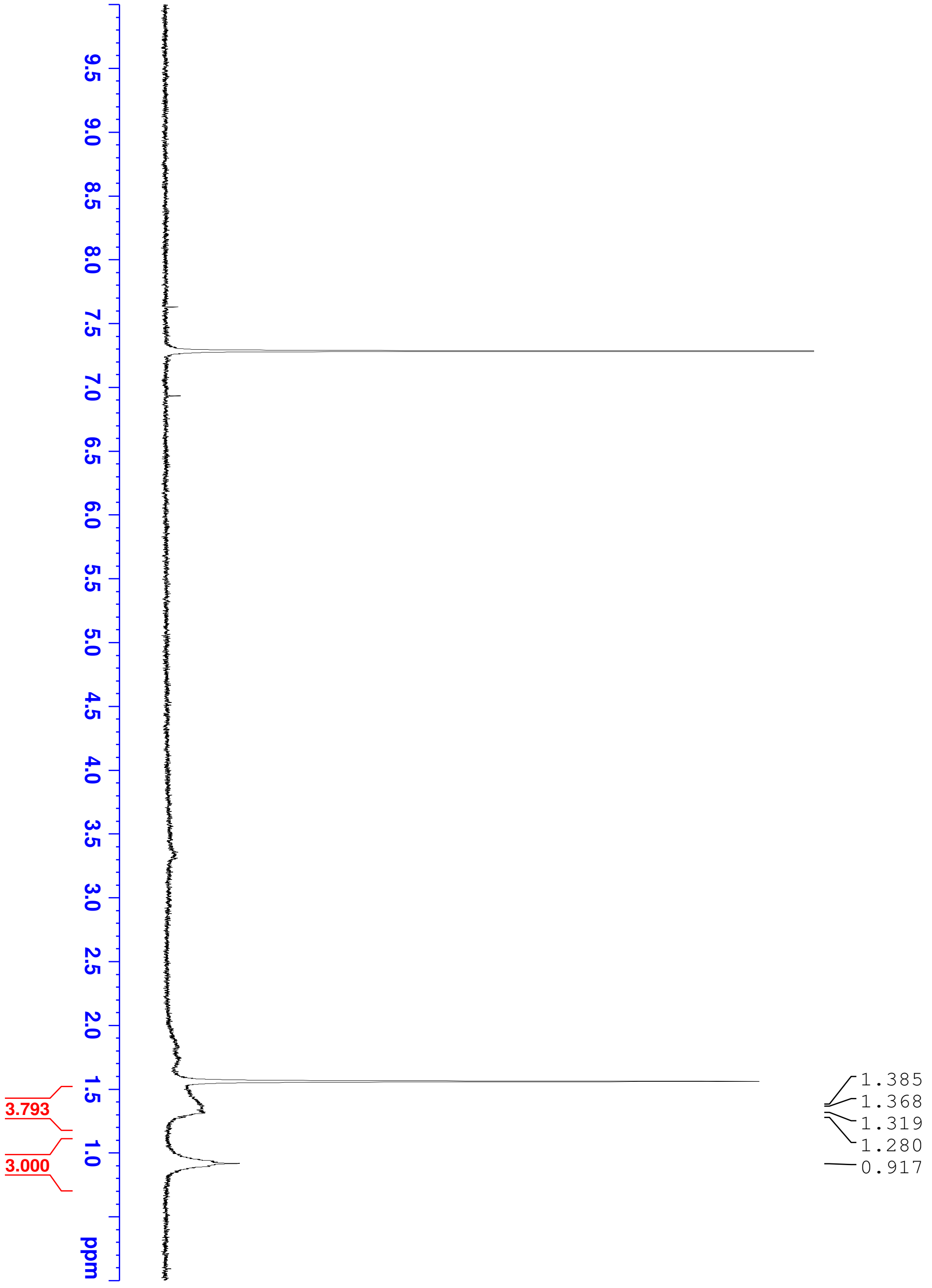

Figure 2: XPS Au Scan of Pentanethiolate Functionalized Au NPs

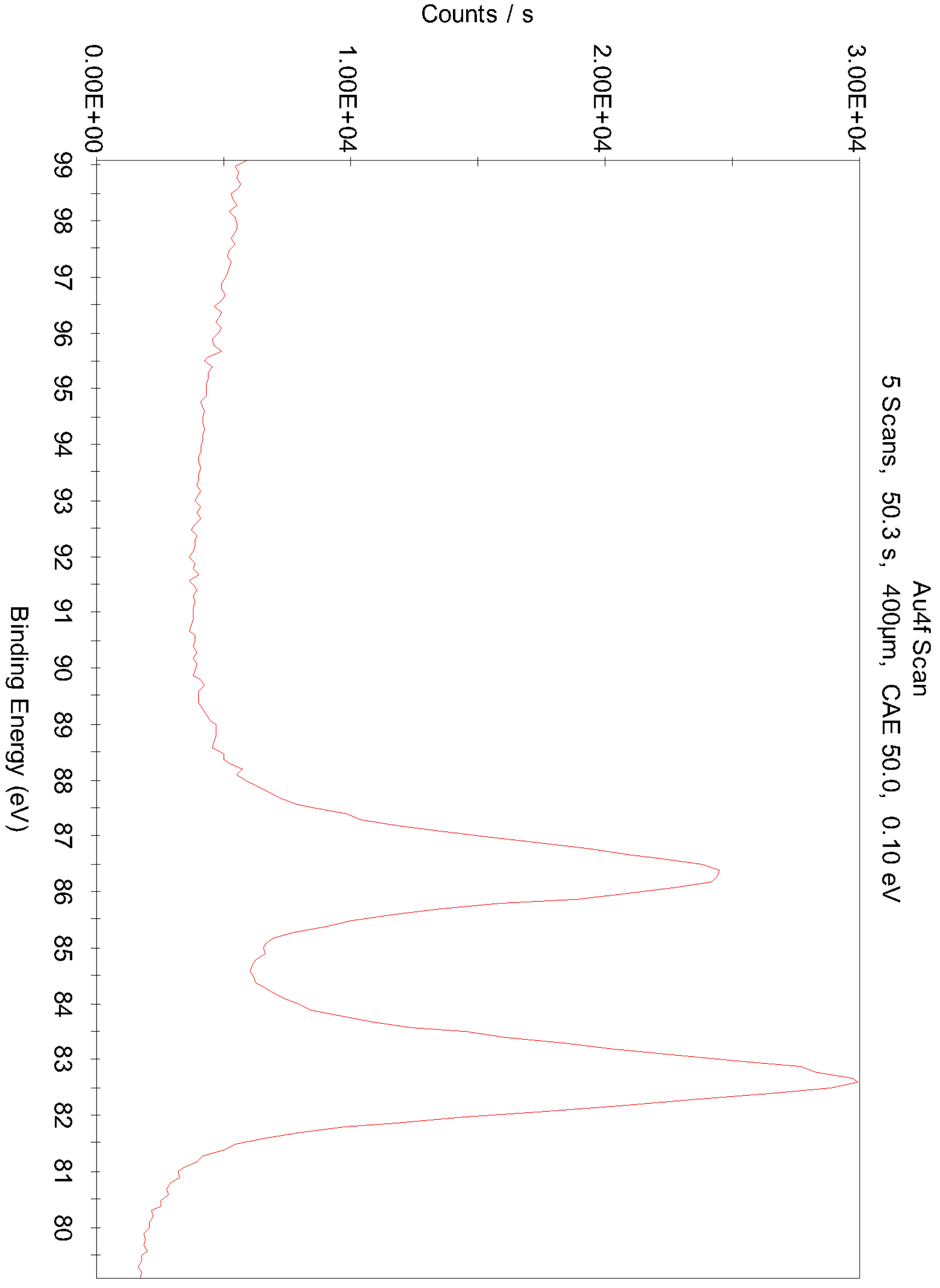

Figure 3: XPS S Scan of Pentanethiolate Functionalized Au NPs

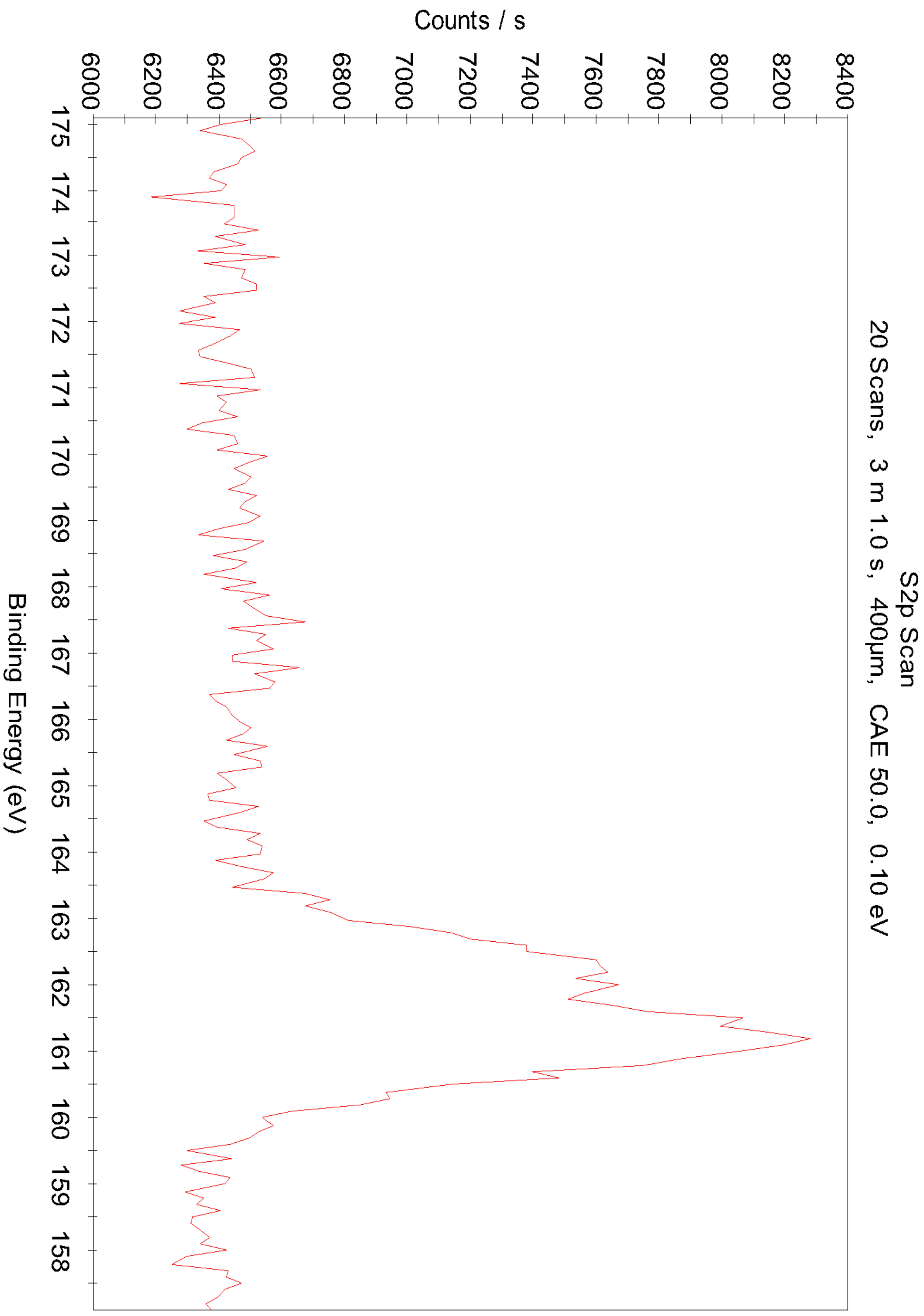

Figure 4: XPS C Scan of Pentanethiolate Functionalized Au NPs

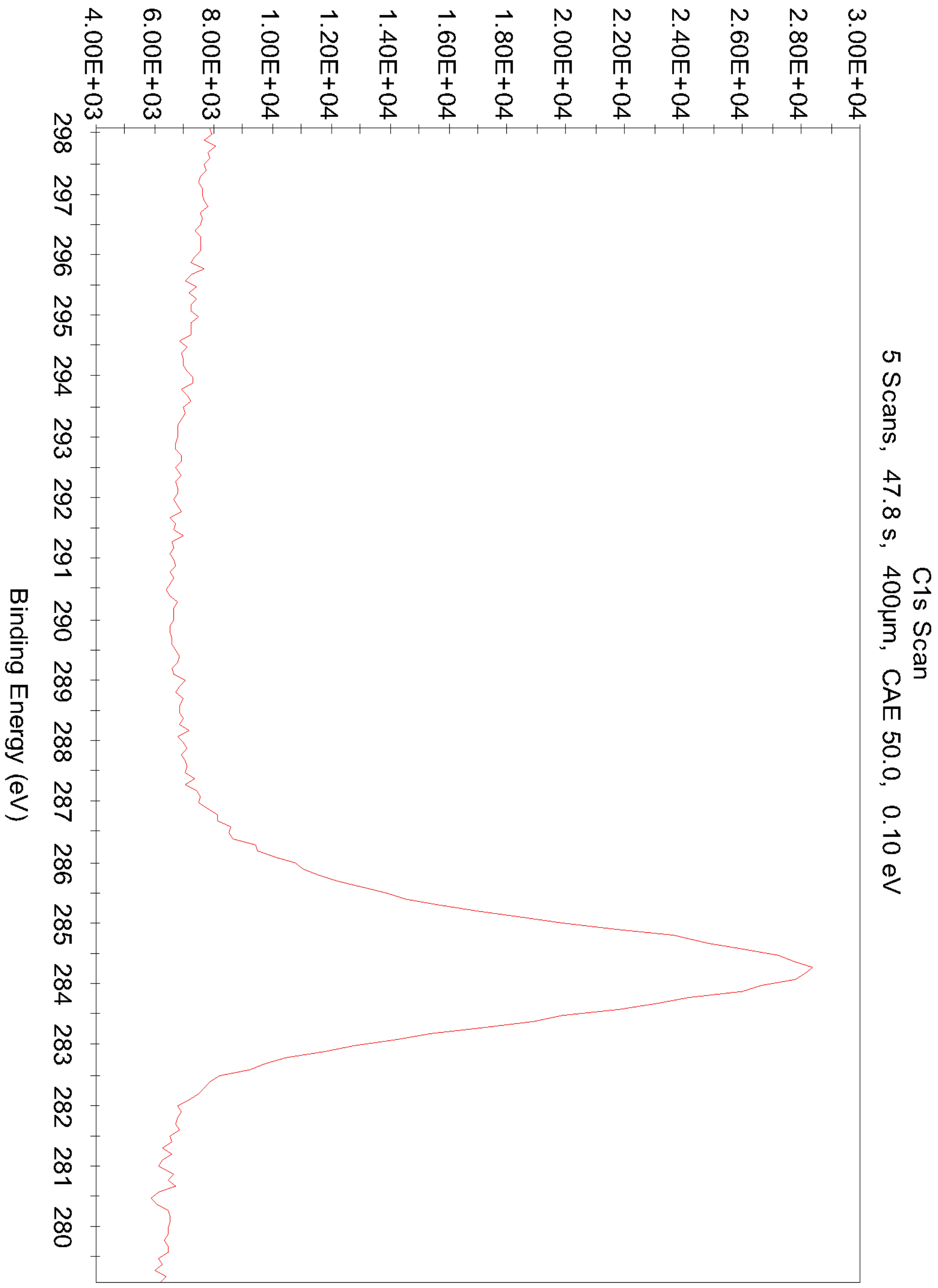

Figure 5: Dodecanethiolate Functionalized Au NPs

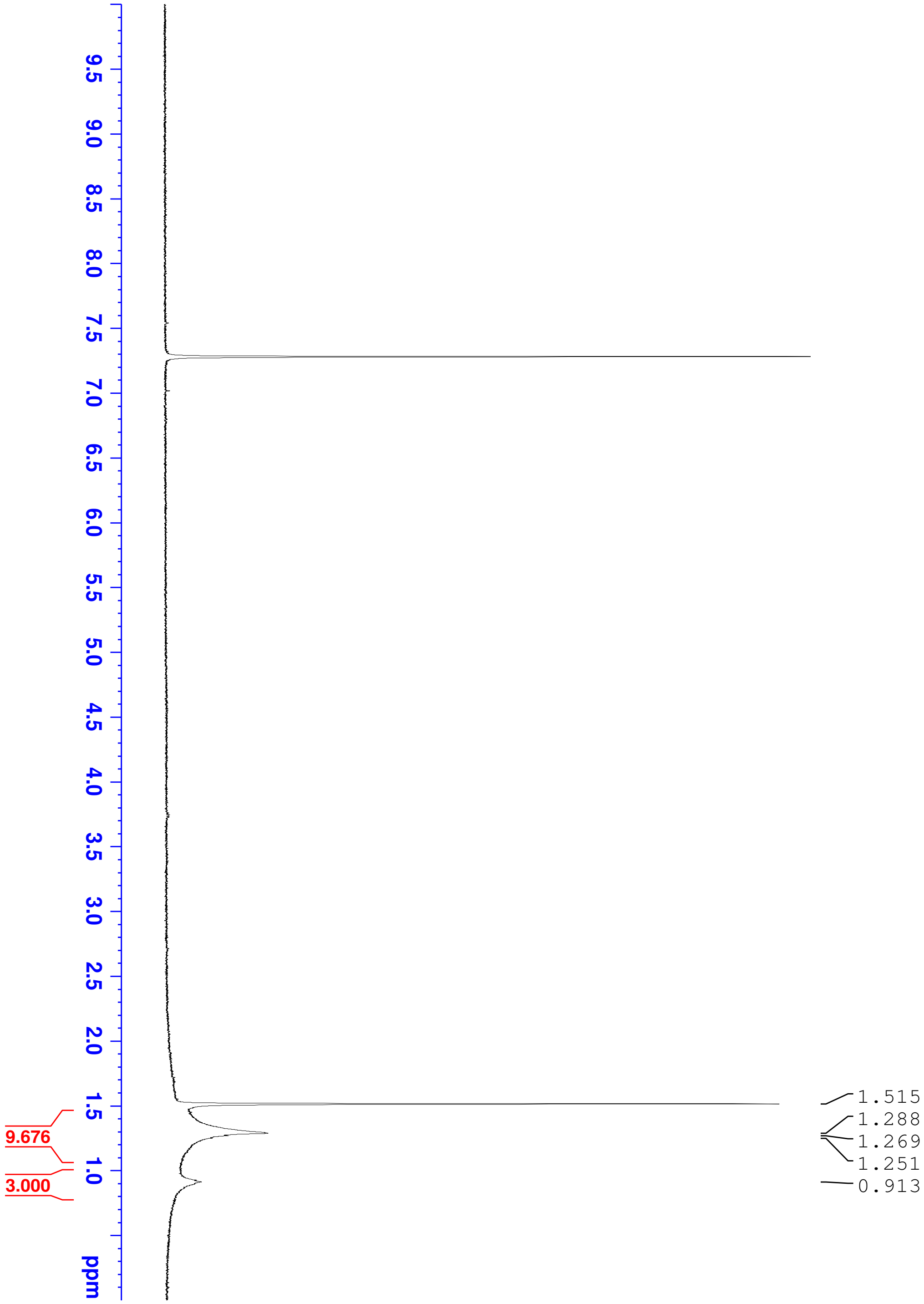

Figure 6: XPS Au Scan of Dodecanethiolate Functionalized Au NPs

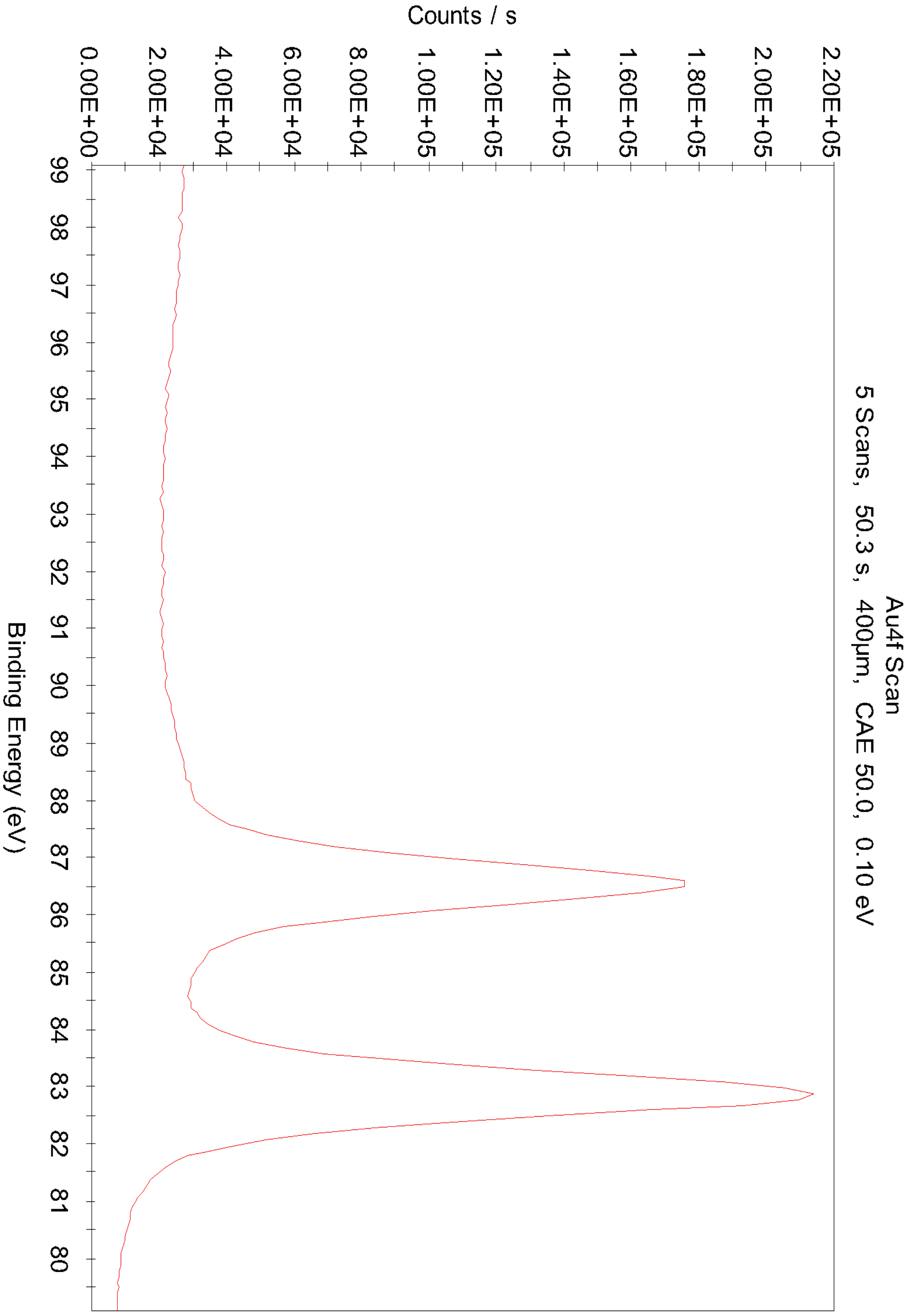

Figure 7: XPS S Scan of Dodecanethiolate Functionalized Au NPs

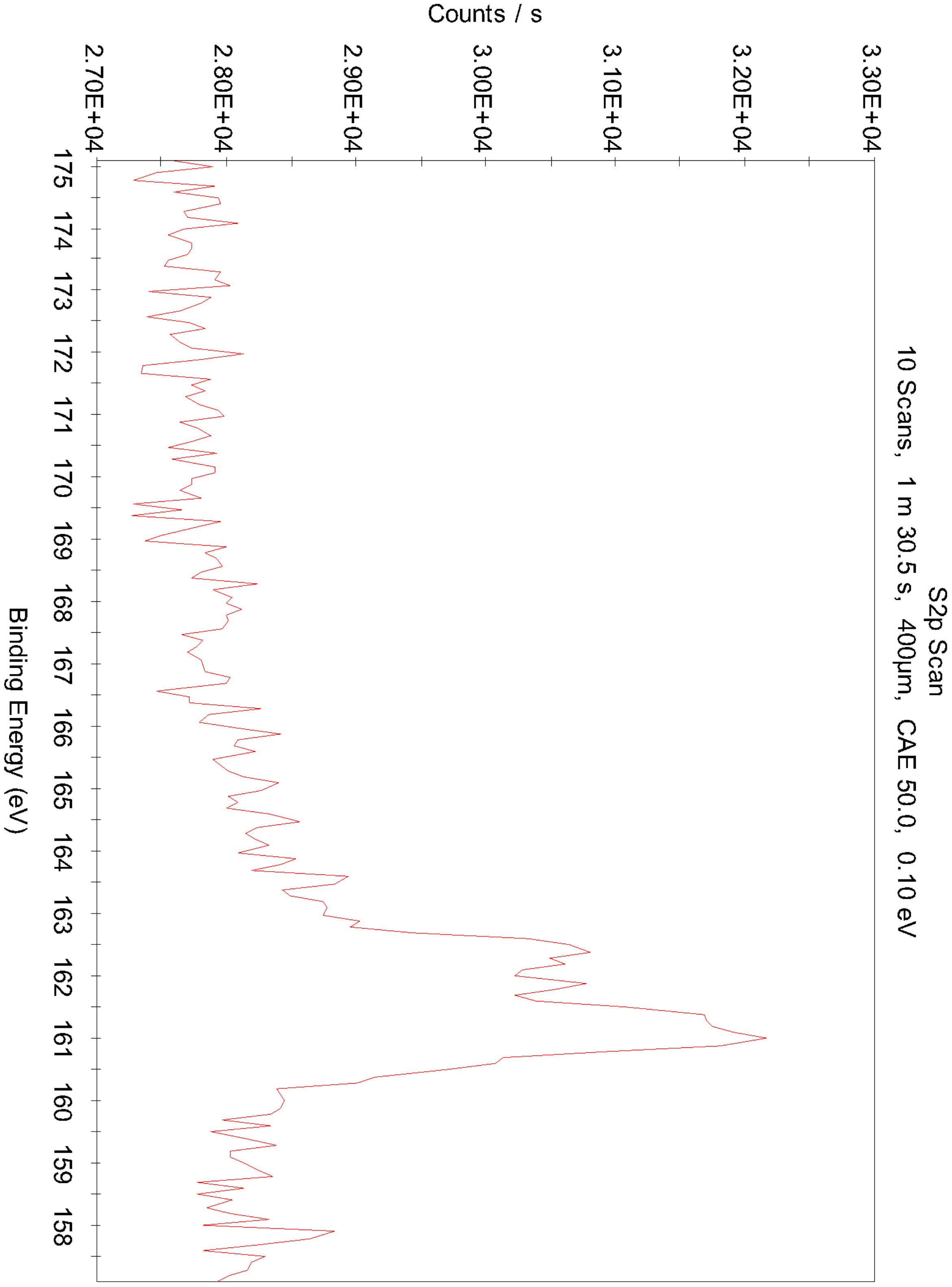

Figure 8: XPS C Scan of Dodecanethiolate Functionalized Au NPs

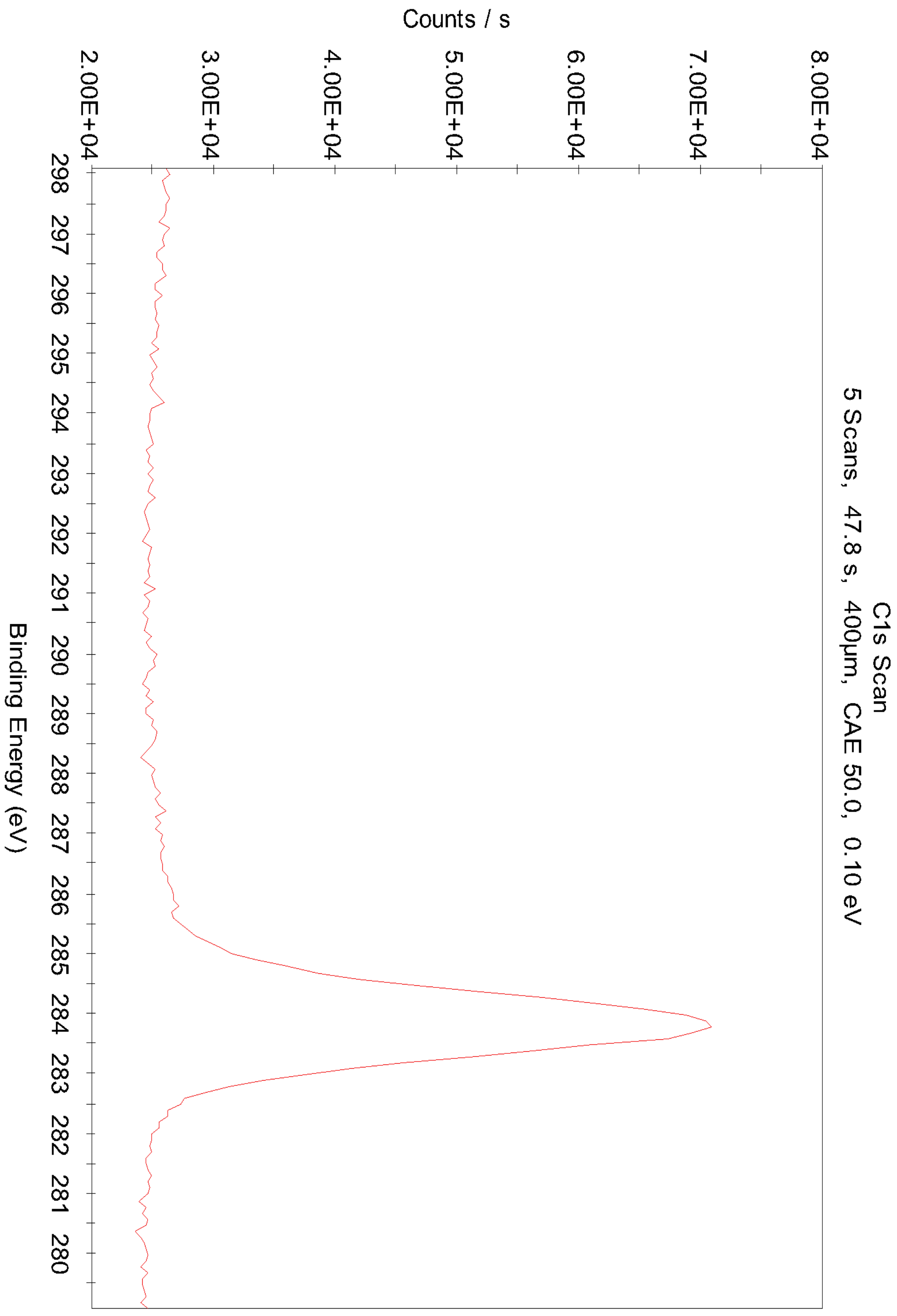

Figure 9: Azide Functionalized Au NPs

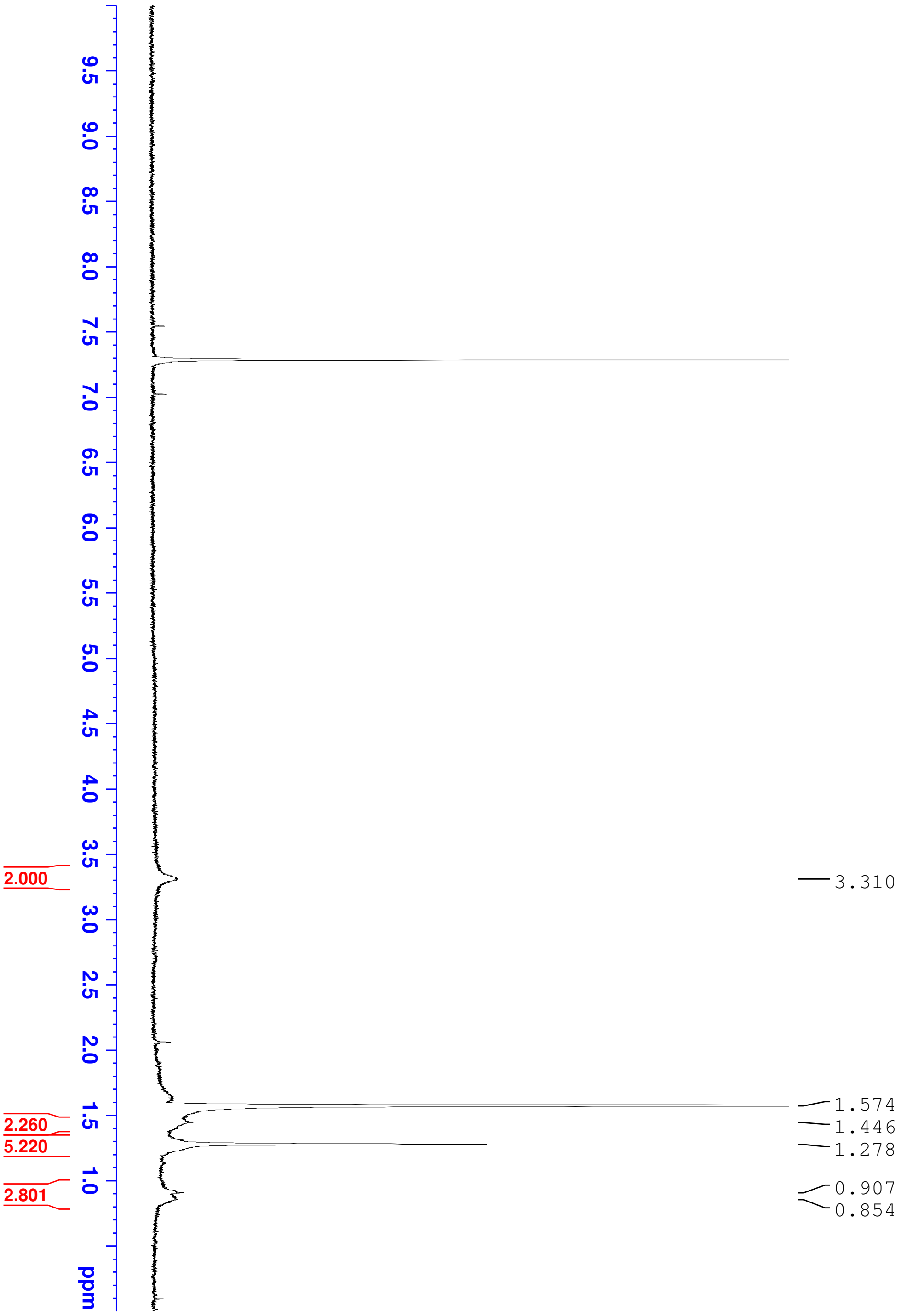

Figure 10: XPS Au Scan of Azide Functionalized Au NPs

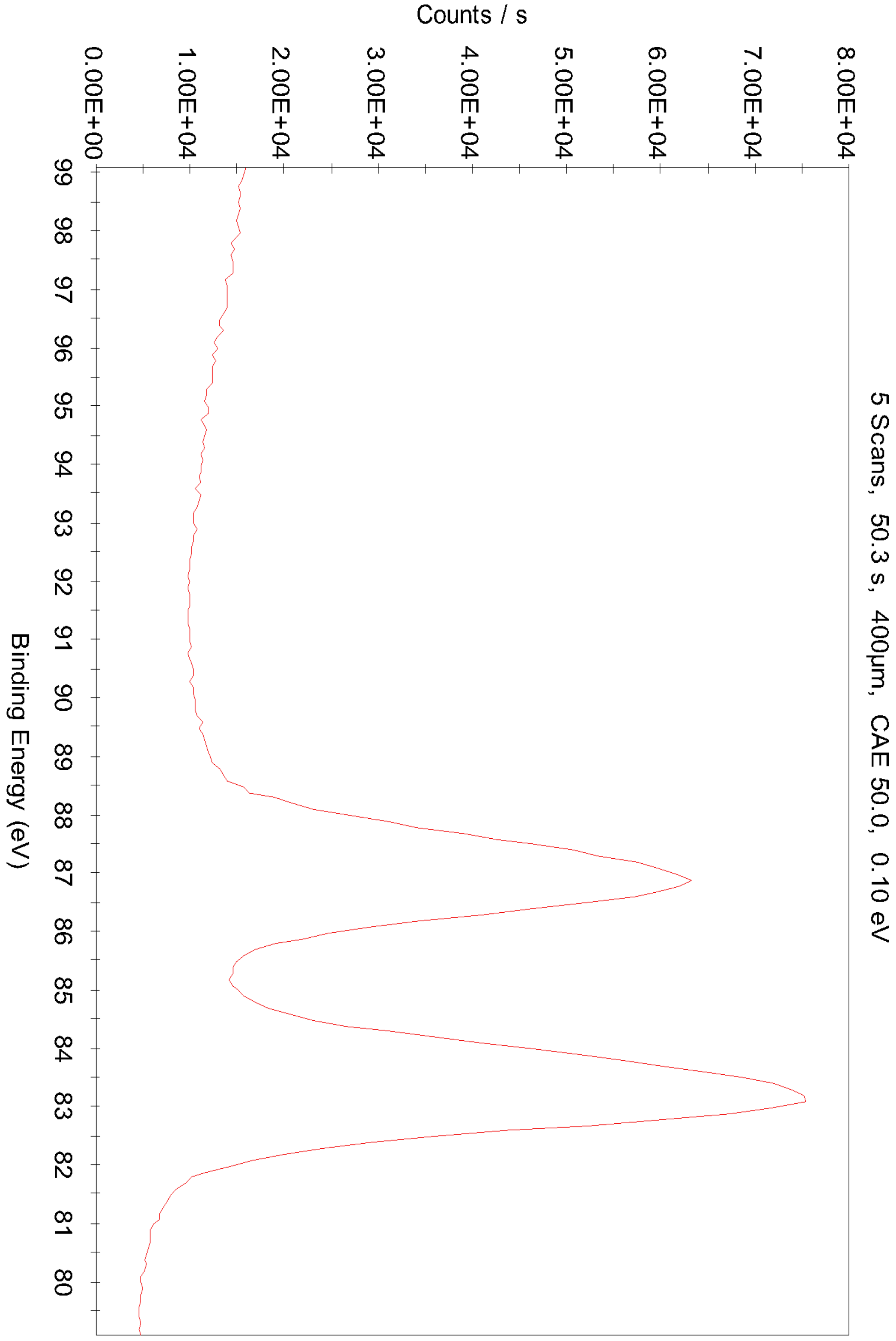

Figure 11: XPS S Scan of Azide Functionalized Au NPs

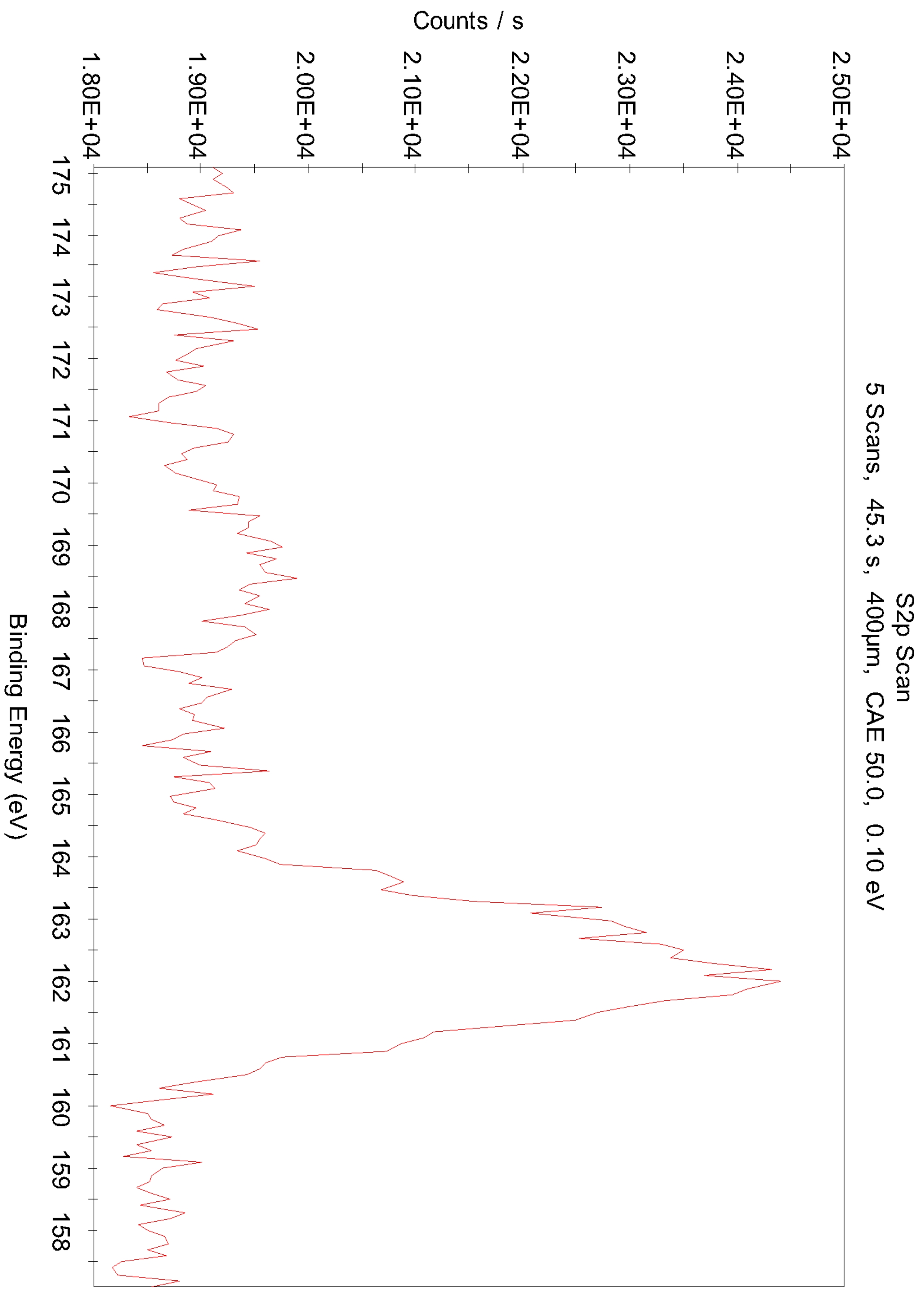

Figure 12: XPS C Scan of Azide Functionalized Au NPs

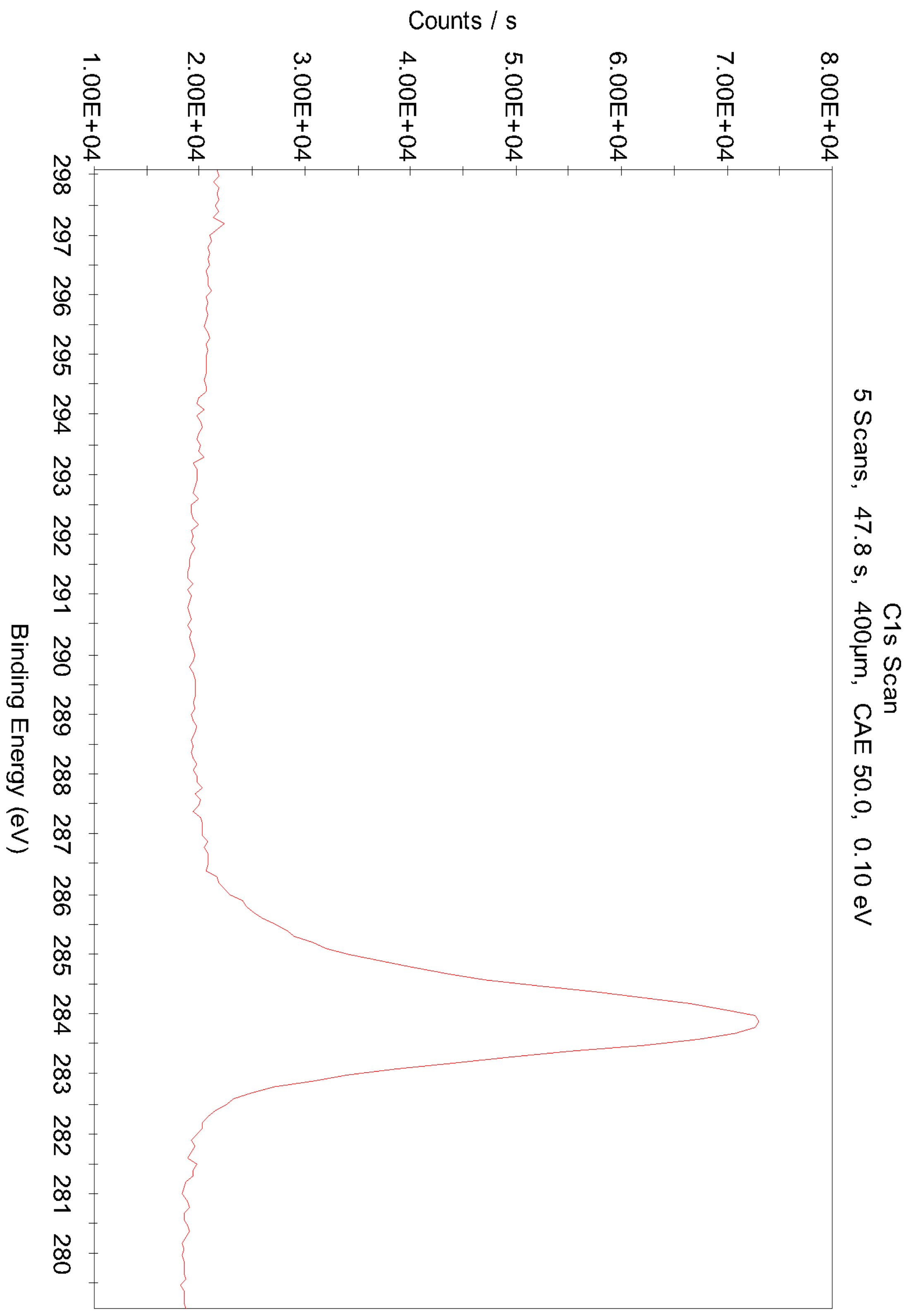

Figure 13: XPS N Scan of Azide Functionalized Au NPs

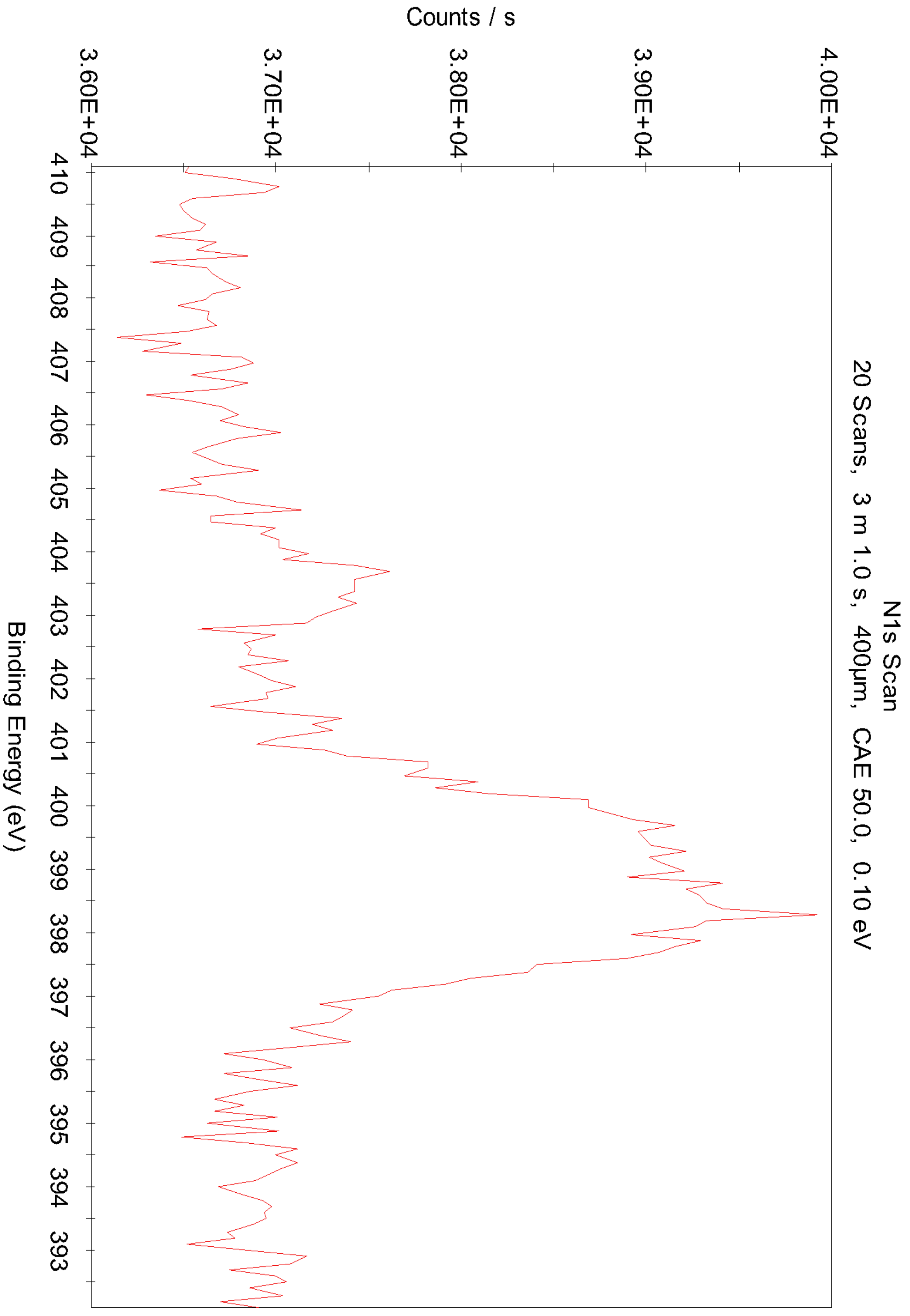

Figure 14: Peroxide Functionalized Au NPs

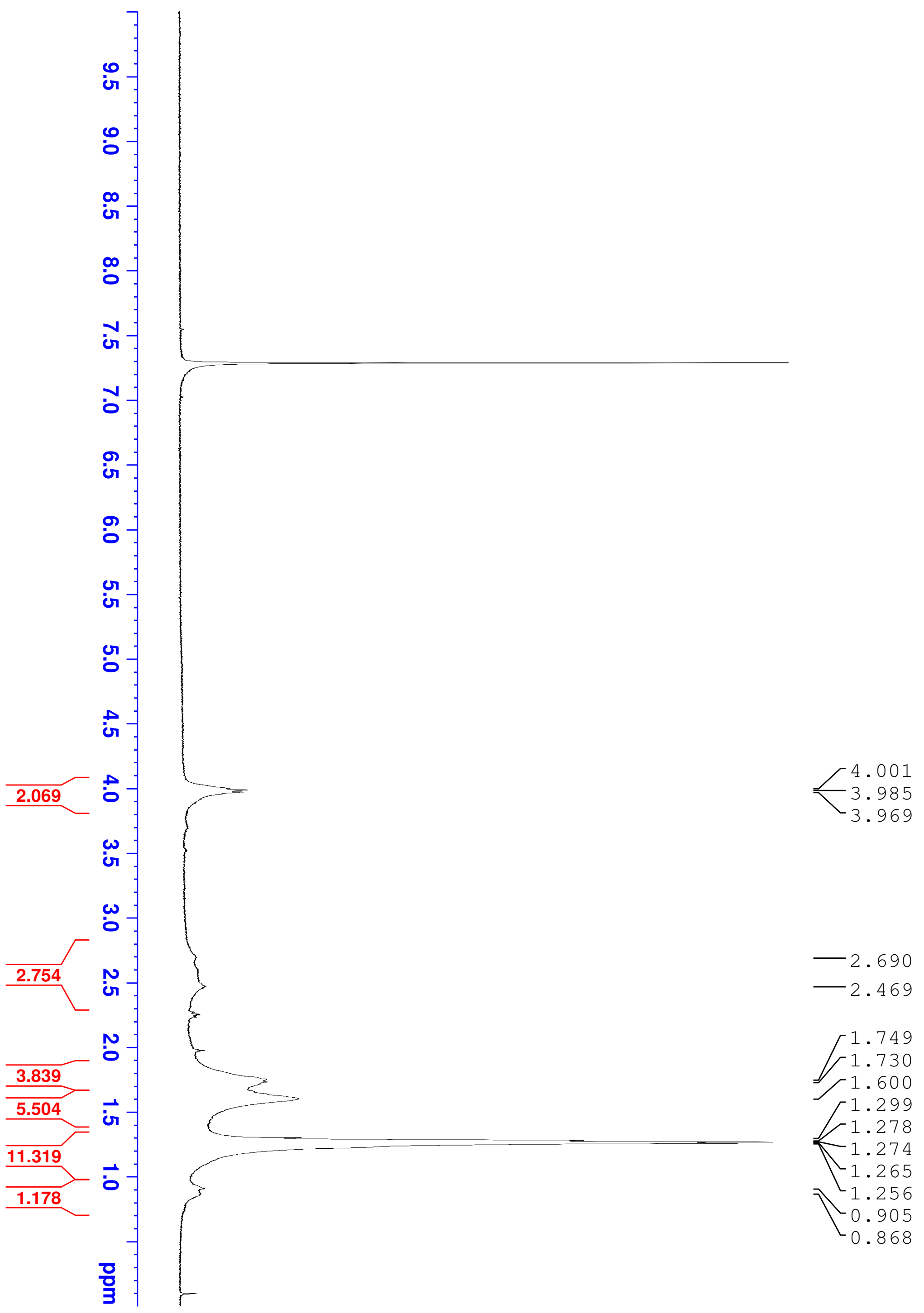

Figure 15: XPS Au Scan of Peroxide Functionalized Au NPs

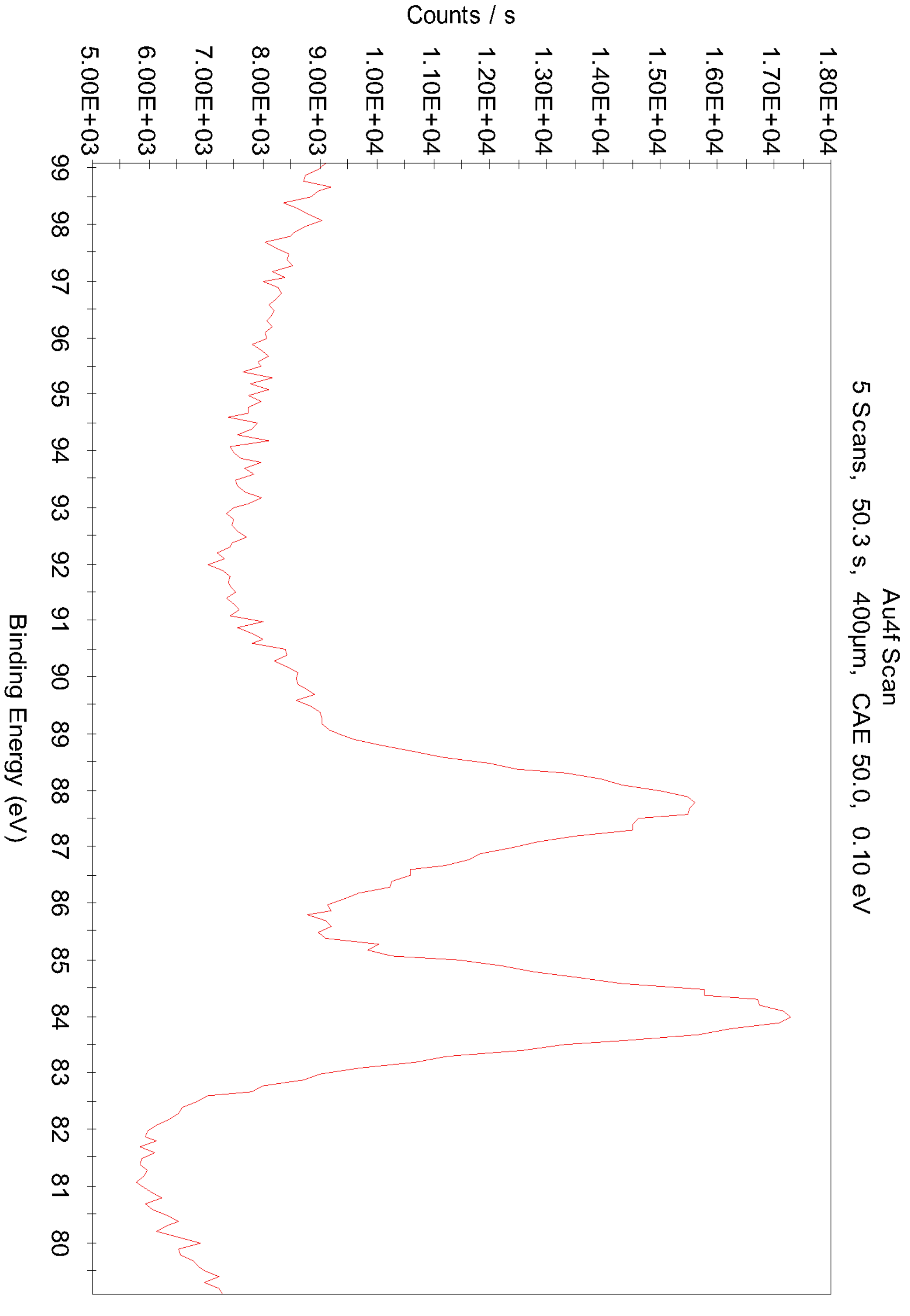

Figure 16: XPS S Scan of Peroxide Functionalized Au NPs

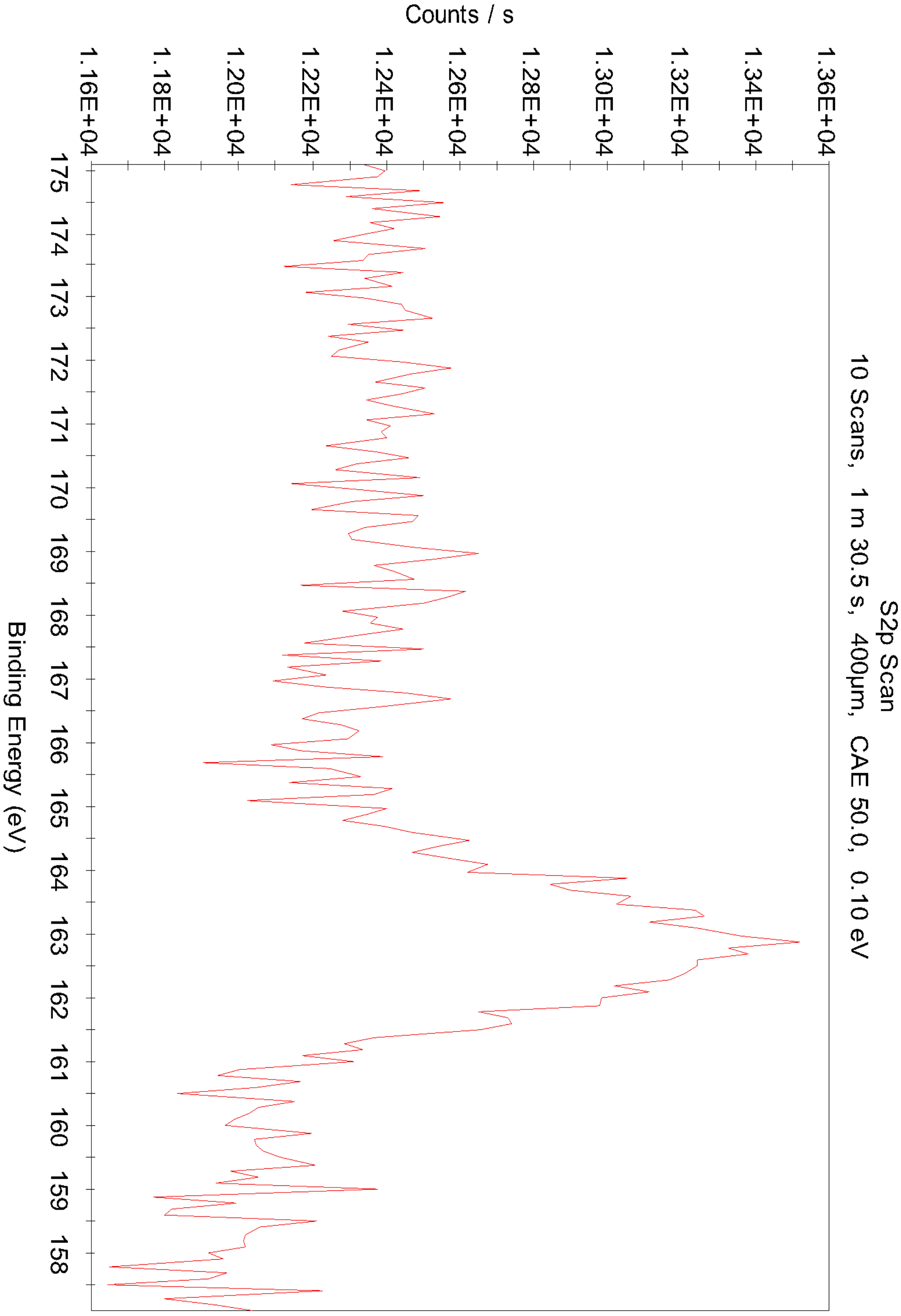

Figure 17: XPS C Scan of Peroxide Functionalized Au NPs

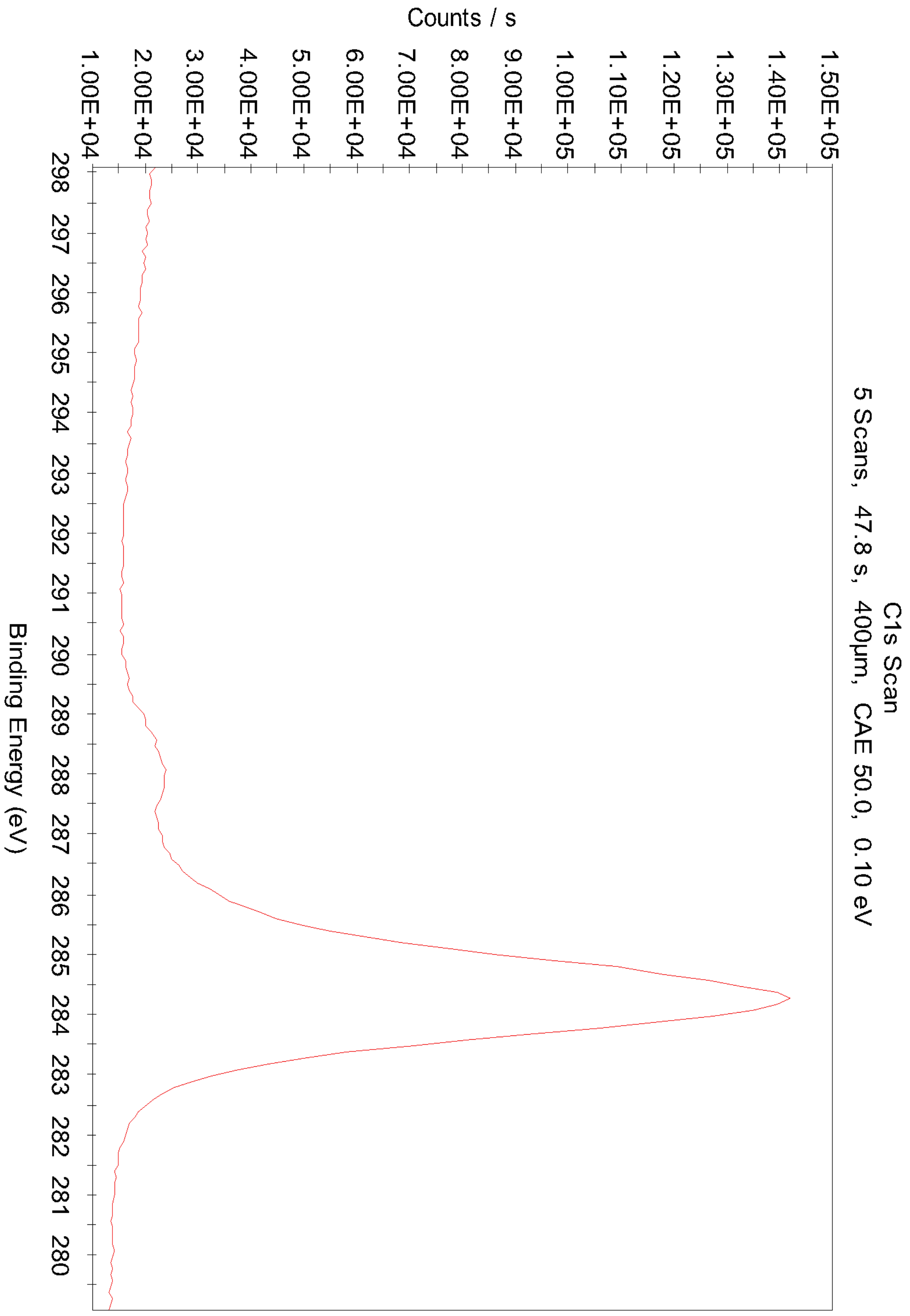

Figure 18: XPS N Scan of Peroxide Functionalized Au NPs

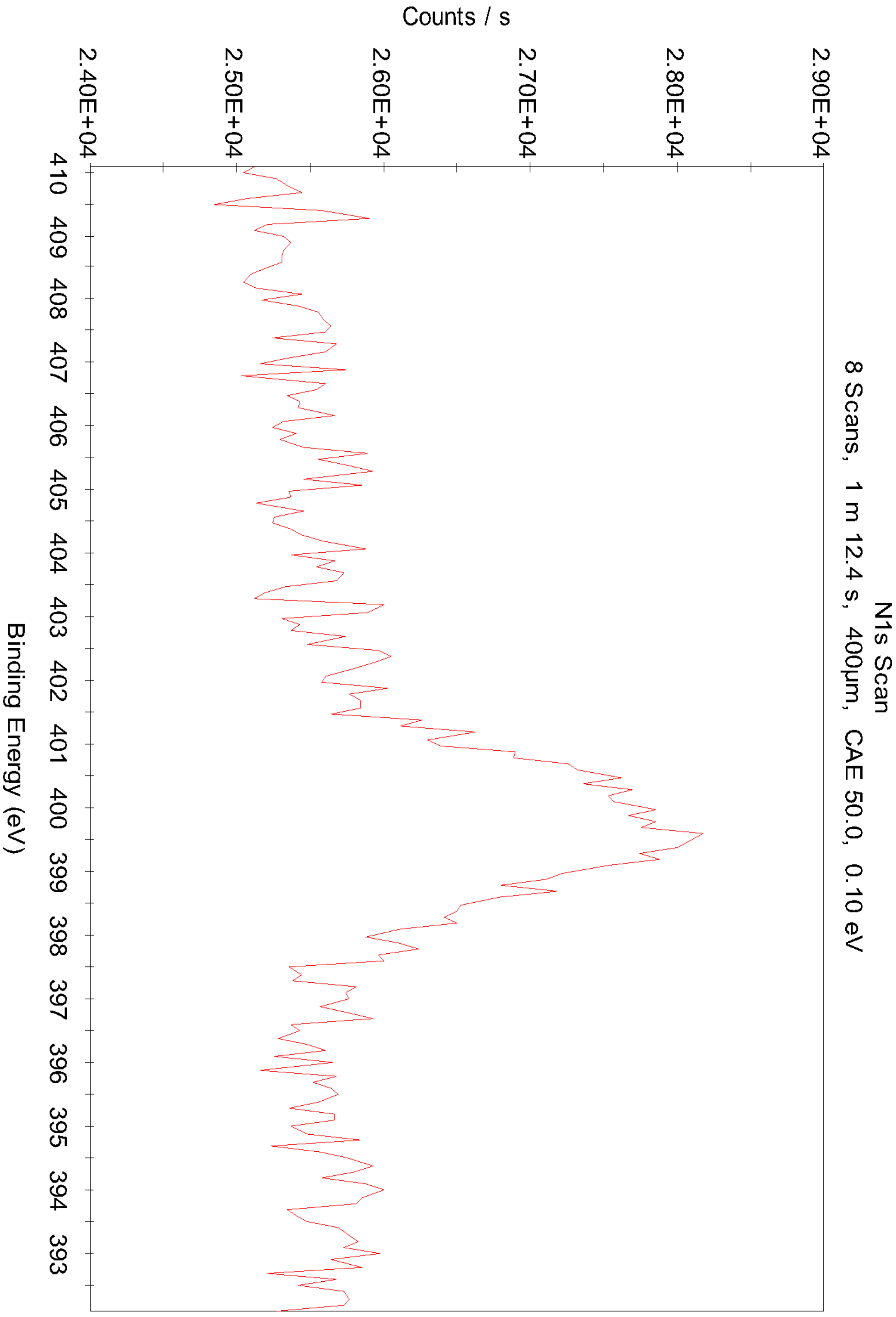

Figure 19: XPS O Scan of Peroxide Functionalized Au NPs

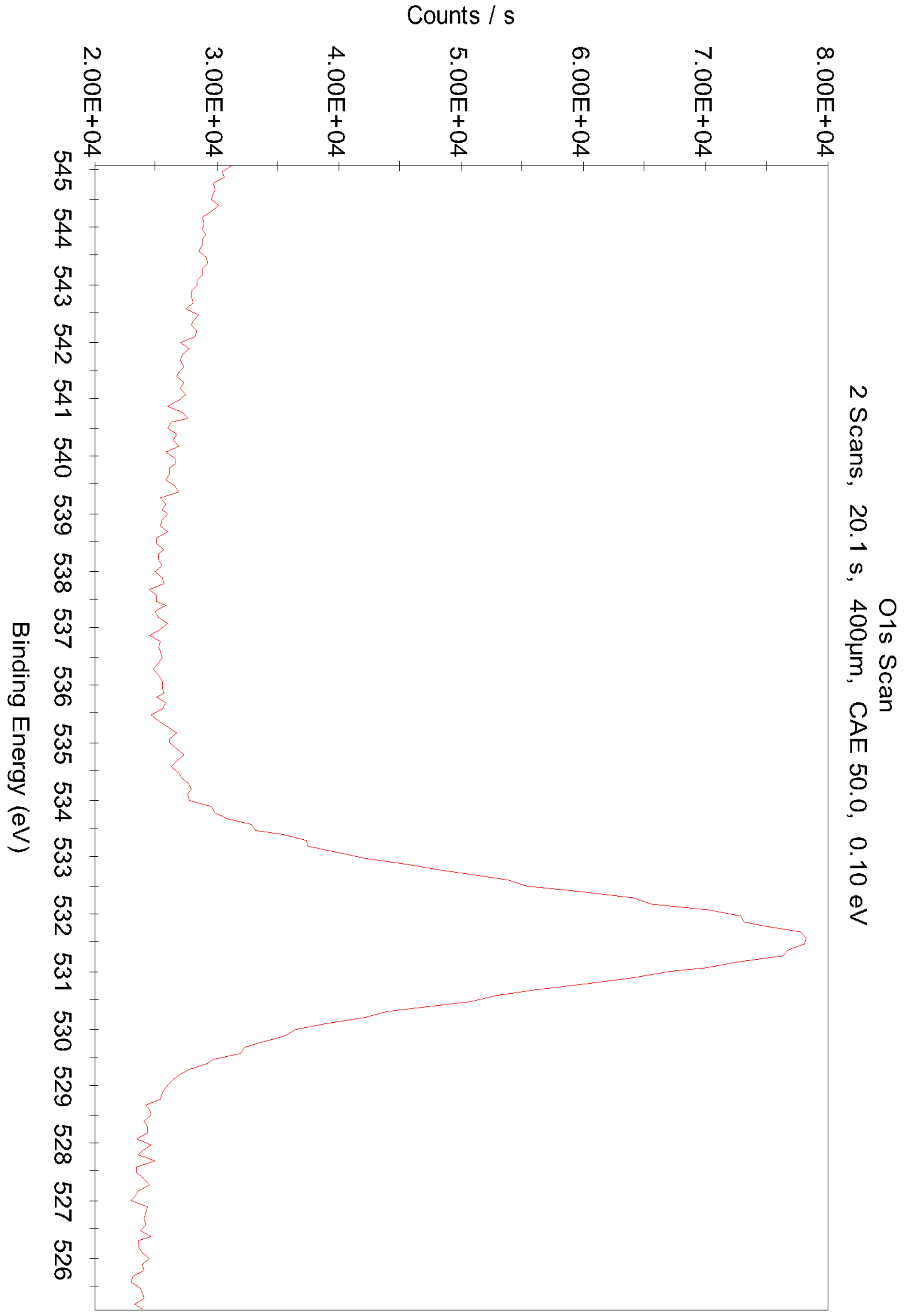

Figure 20: Peroxyacetal Functionalized Au NPs

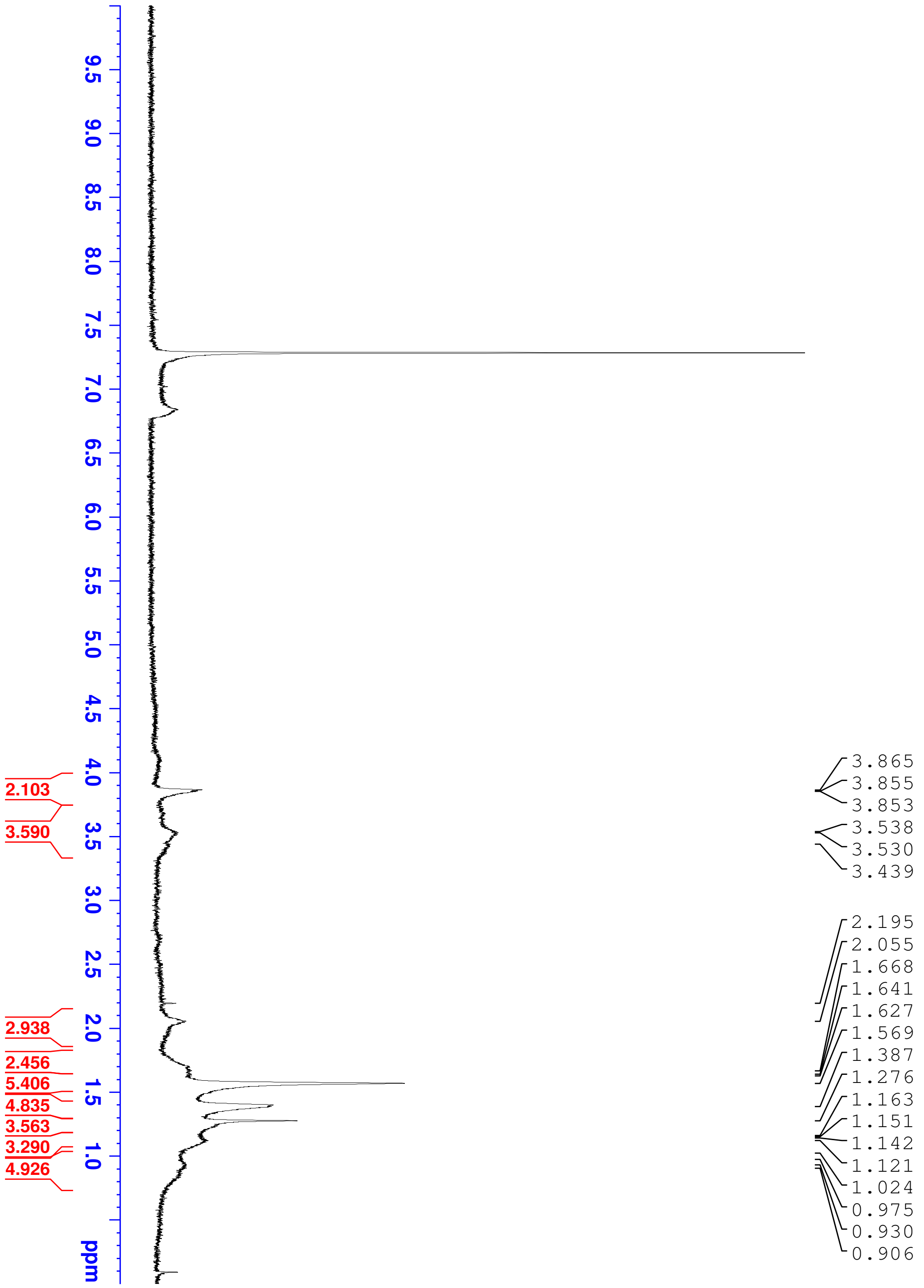

Figure 21: XPS Au Scan of Peroxyacetal Functionalized Au NPs

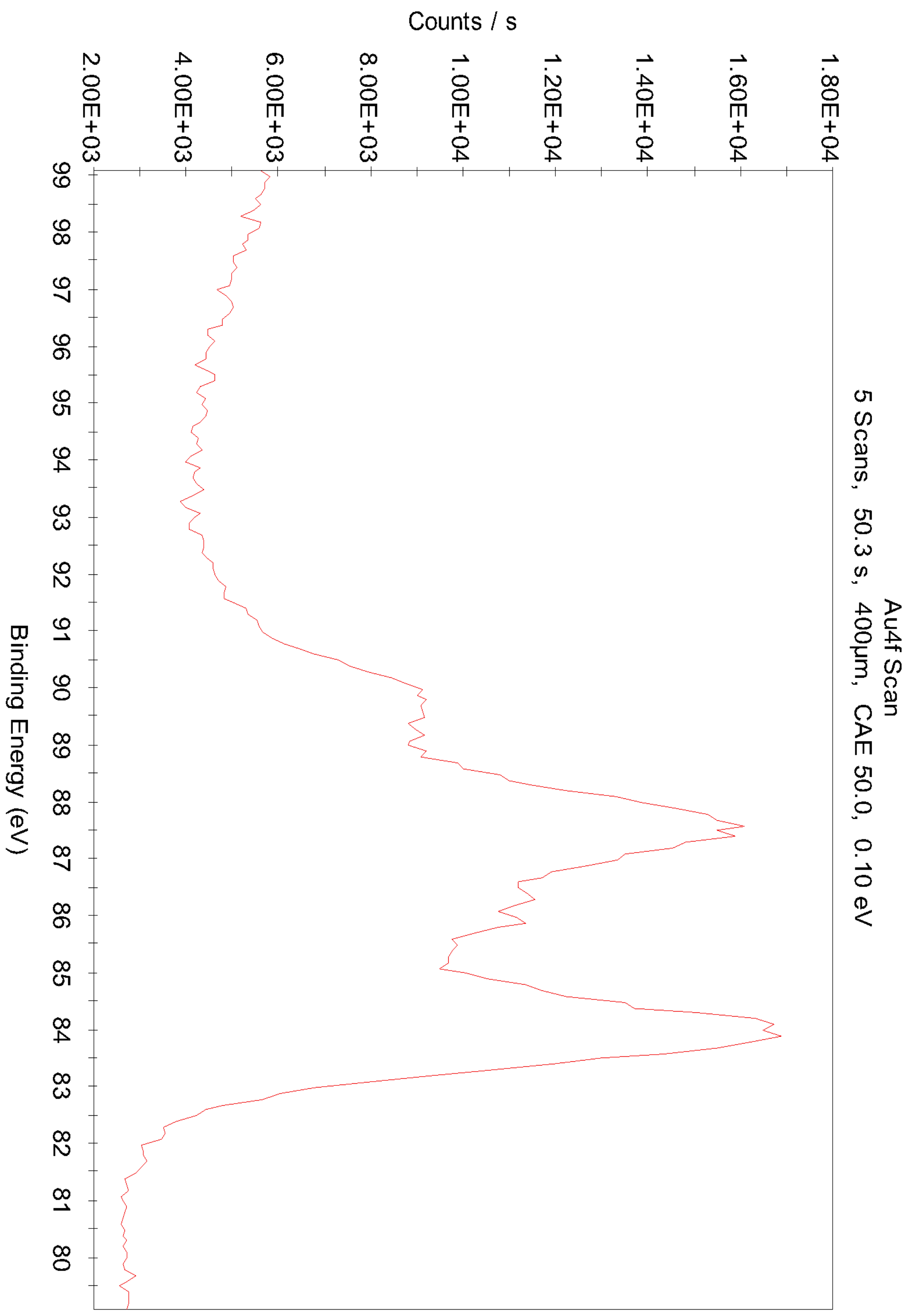

Figure 22: XPS S Scan of Peroxyacetal Functionalized Au NPs

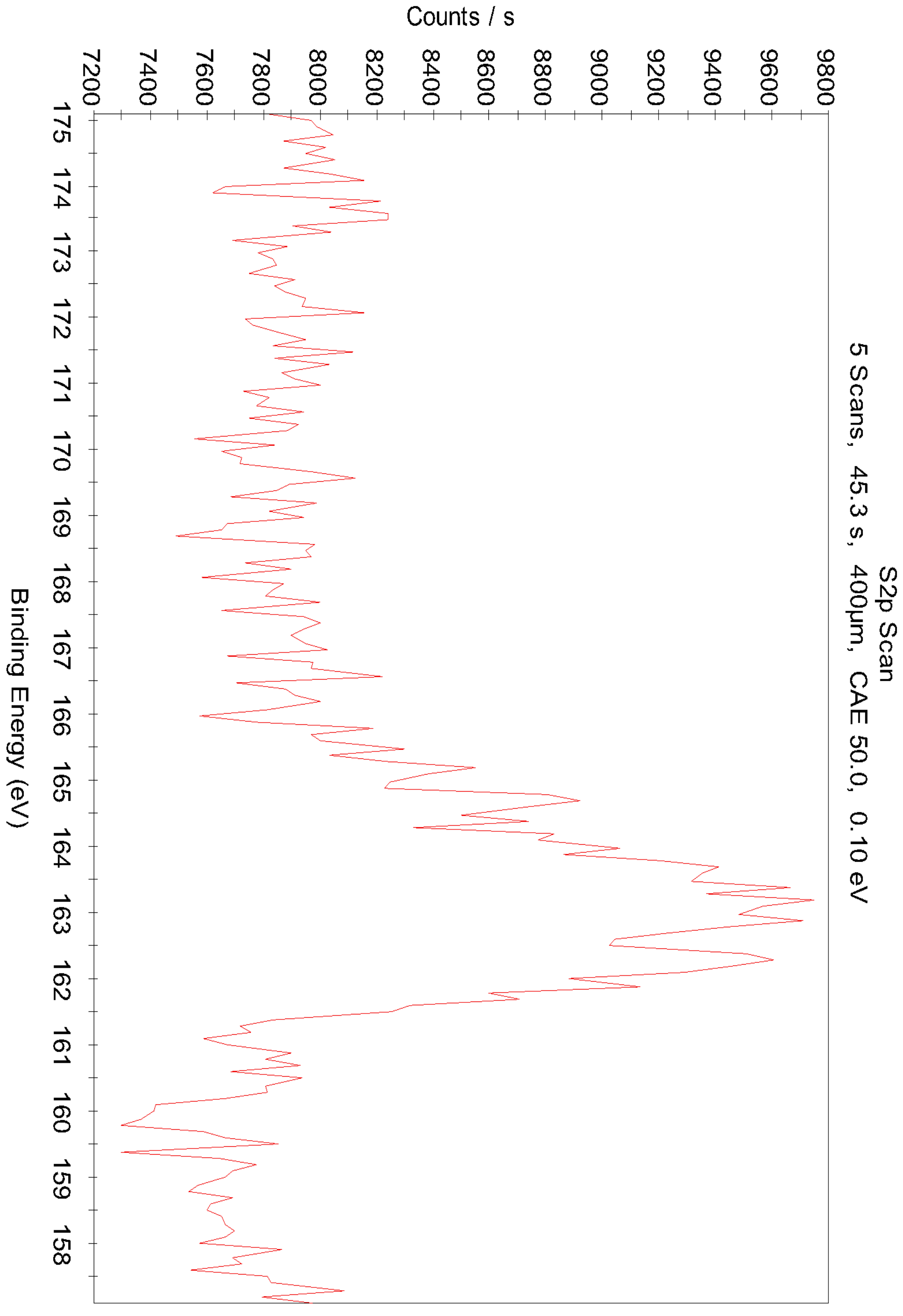

Figure 23: XPS C Scan of Peroxyacetal Functionalized Au NPs

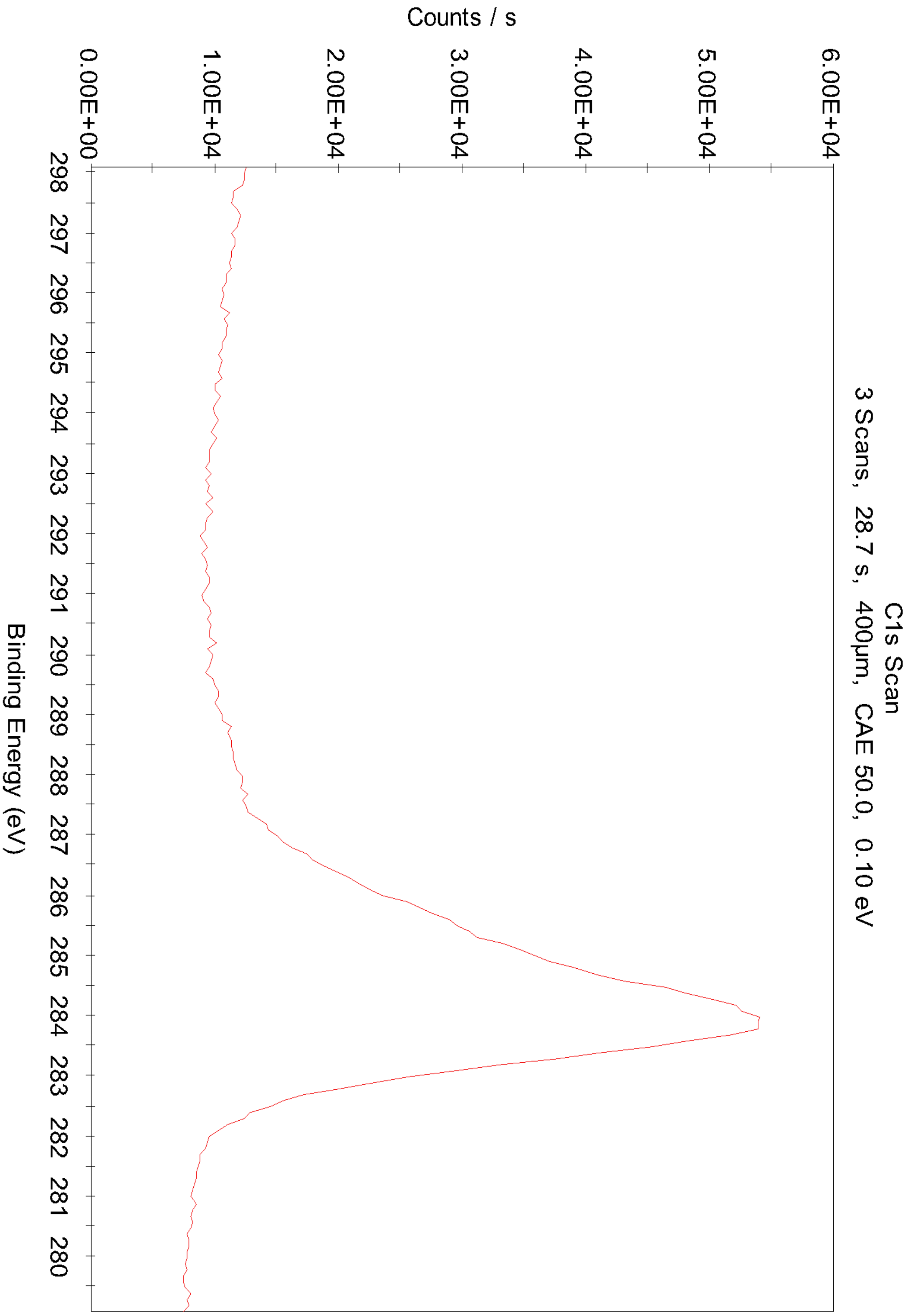

Figure 24: XPS N Scan of Peroxyacetal Functionalized Au NPs

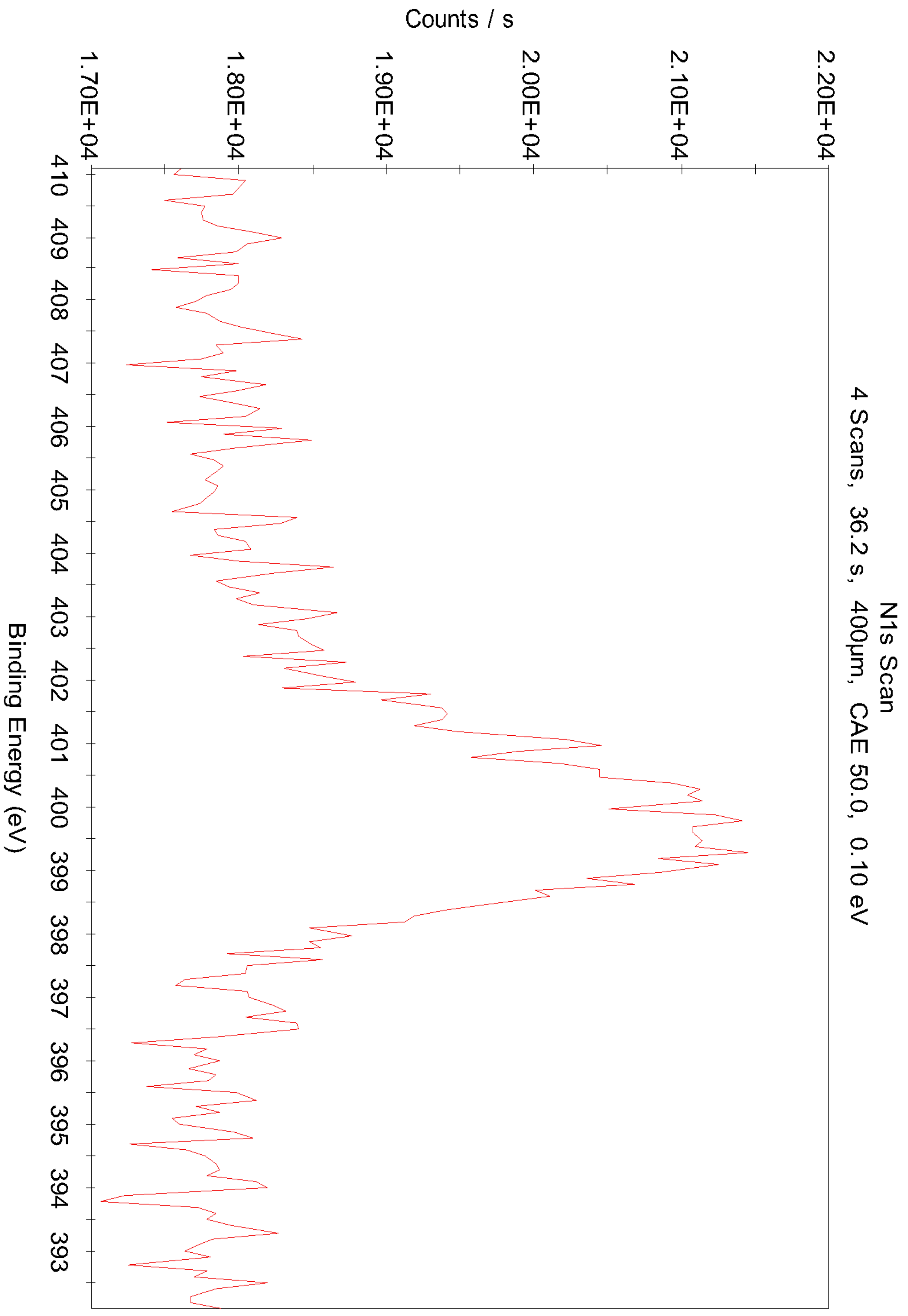

Figure 25: XPS O Scan of Peroxyacetal Functionalized Au NPs

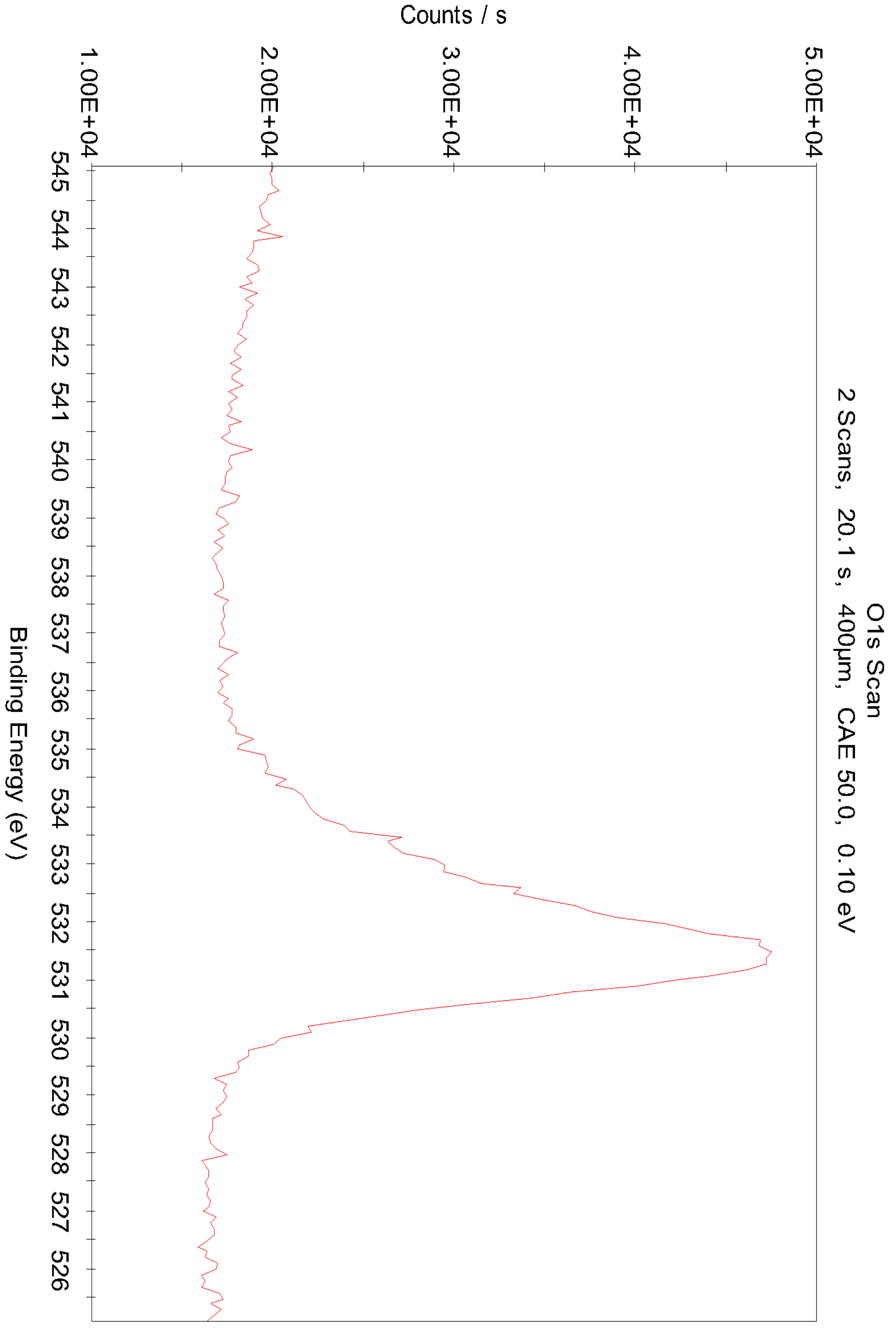

Supplement: RA-010-D0RA09088C-s001 [file RA-010-D0RA09088C-s001.pdf]
